# The Interplay Between Component Denticity and Flexibility Promotes the Formation of [Ag^I^⋅⋅⋅Ag^I^]‐stabilised Links and Knots

**DOI:** 10.1002/anie.202423962

**Published:** 2025-01-12

**Authors:** Aleksandra Sarwa, Andrei Khmara, Krzysztof A. Konieczny, Dagmara Kulesza, Eugeniusz Zych, Bartosz Trzaskowski, Bartosz Szyszko

**Affiliations:** ^1^ Faculty of Chemistry University of Wrocław 14 F. Joliot-Curie St. 50-383 Wrocław Poland; ^2^ Department of Chemistry and Biochemistry University of California, Los Angeles California 90024-1569 USA; ^3^ Faculty of Chemistry Wrocław University of Science and Technology Wybrzeże Wyspiańskiego 27 Wrocław 50-370 Poland; ^4^ Centre of New Technologies University of Warsaw 2c Banach St. 02-097 Warsaw Poland

**Keywords:** supramolecular chemistry, self-assembly, catenanes, knots, silver

## Abstract

A subtle interplay between the flexibility of the 2,2’‐bipyridyl‐based diamine and the denticity of the coordination domain formed upon self‐assembly enabled the formation of four distinct topologies stabilised by [Ag⋅⋅⋅Ag]^2+^ pairs. The reactions utilising 2,6‐diformylpyridine resulted in the formation of silver(I)‐stabilised molecular tweezer, trefoil knot, and Solomon link. The 1,8‐naphthyridine‐based dialdehyde promoted the formation of [2]catenanes and trefoil knot, demonstrating very close Ag^I^⋅⋅⋅Ag^I^ distances. Two of the studied assemblies demonstrated interesting luminescent properties in the solid state.

## Graphical abstract

The interplay between the diamine‘s flexibility and the coordination domain‘s denticity governed the self‐assembly of four distinct [Ag^I^⋅⋅⋅Ag^I^]‐stabilised architectures. Reactions with 2,6‐diformylpyridine produced molecular tweezer, trefoil knot, and Solomon link, while 1,8‐naphthyridine‐based dialdehyde promoted the formation of [2]catenanes and trefoil knot. Selected assemblies showed interesting luminescent properties.

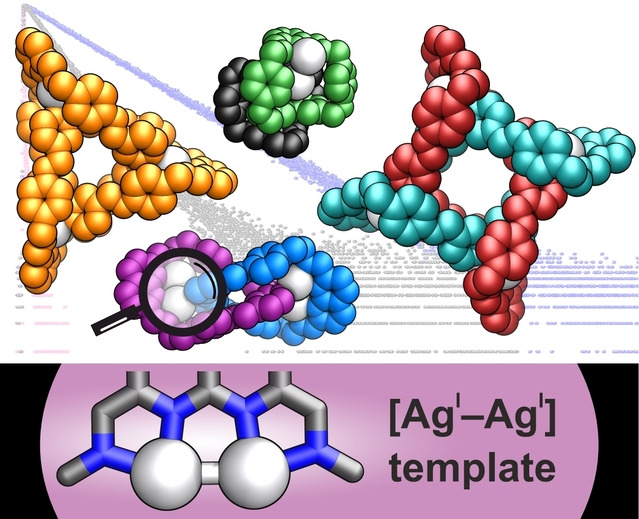

The self‐assembly approach constitutes one of the most powerful synthetic methods for constructing intricate architectures.^[1]^ Among several variants, the subcomponent self‐assembly,^[2]^ being a type of metal coordination‐driven self‐organisation process,^[3]^ relies on the in situ ligand formation accompanied by its organisation around metal template into the supramolecular architecture. The method was effective for constructing macrocycles,[^4^, ^5^] helicates,[^6^, ^7^] and molecular links and knots of complex topologies.[^8^, ^9^, ^10^, ^11^, ^12^]

The careful selection of metal, amine, and aldehyde subcomponents plays a crucial role in the creation of an assembly of desired topology, size, and shape, allowing to preprogramme its reactivity, guest binding, luminescence, and magnetic properties.[^13^, ^14^, ^15^] The design of the components incorporating carefully tailored coordination domains separated through the linkers of suitable flexibility is of fundamental importance for targeting the specific topology of the interlocked architecture.[^10^, ^11^, ^12^, ^16^, ^17^, ^18^, ^19^, ^20^, ^21^] The metal‐binding moiety's structure was shown to significantly affect the reactivity and dynamics of the assembly.[^22^, ^23^]

The choice of a metal template remains of fundamental importance. Due to the significant tolerance of the Ag(I) to the type, number, and geometric arrangements of coordination donors and its ability to form π‐complexes, silver(I) was often employed to create systems of considerable complexity.[^12^, ^24^, ^25^, ^26^, ^27^, ^28^] However, the propensity of Ag(I) to form clusters through argentophilic interactions^[29]^ was only seldom employed in the design of supramolecular architectures.[^30^, ^31^, ^32^, ^33^, ^34^, ^35^]

Here, we demonstrate how a subtle interplay between the flexibility of the diamine component and the design of the coordination domain enabled the formation of four distinct topologies, each stabilised by [Ag⋅⋅⋅Ag]^2+^ moieties. Extending the template concept from a single cation to a cluster of metal atoms opens new opportunities for constructing original topologies and architectures showing intriguing features, such as magnetism, luminescence and catalysis.[^31^, ^32^, ^33^, ^36^, ^37^]

The reaction of diamine **1** ^[11]^ with diformylpyridine **2** was carried out with excess silver(I) acetate (10 equiv.) in *iso*‐propanol at 70 °C for 20 hours (Scheme 1). Although the starting materials reacted, forming majorly an insoluble precipitate that could not be identified, a careful post‐synthetic work‐up allowed for the isolation of the molecular tweezer **3–2Ag_2_** with a 2 % yield. The elemental composition of the product was determined by ESI MS (electrospray ionisation mass spectrometry), which indicated the incorporation of four silver(I) cations in **3–2Ag_2_** (Figure S2). Interestingly, using a smaller excess of AgOAc or using a different Ag(I) source (triflate, hexafluorophosphate, nitrate, and fluoride) resulted in the formation of none or only traces of imine assemblies.

**Scheme 1:**
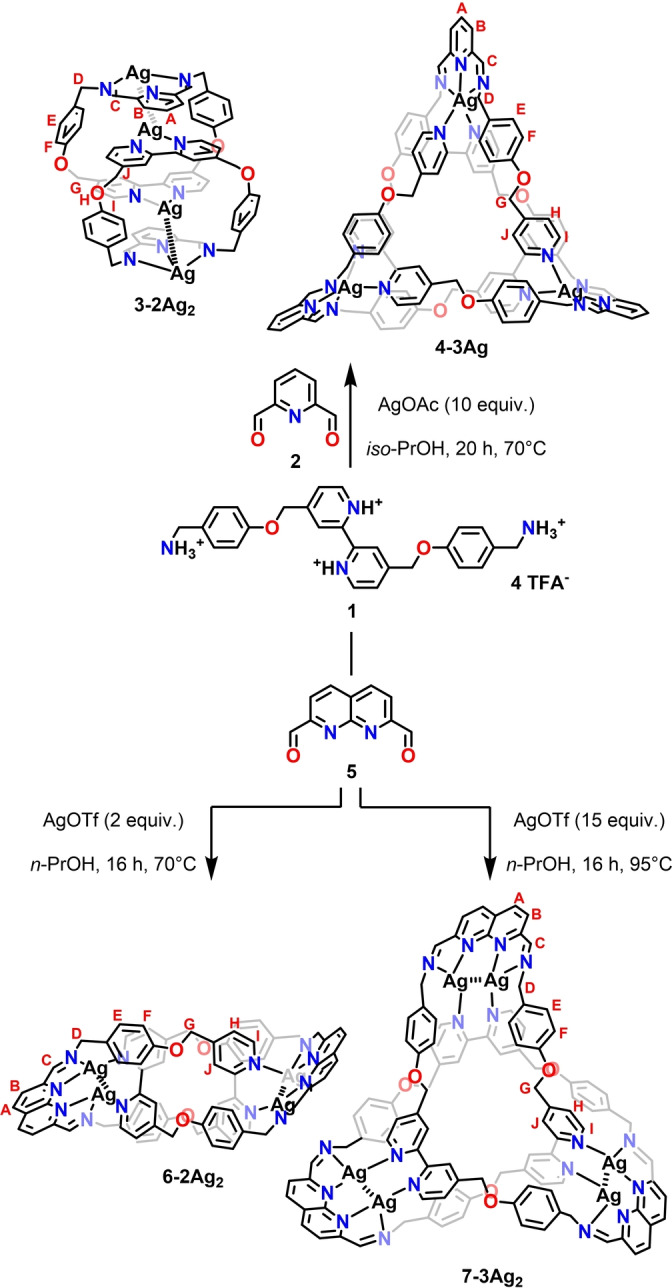
Synthesis of **3–2Ag_2_**, **4–3Ag**, **6–2Ag_2_** and **7–3Ag_2_**. Anions and charge of the assemblies were omitted for clarity.

The ^1^H, ^13^C, and ^19^F NMR spectroscopy, in combination with homo‐ and heteronuclear 2D NMR techniques, allowed for the structure elucidation (Figures S31–40). The ^1^H NMR spectrum of **3–2Ag_2_** showed ten signals correspondingly to the *C*_2v_ effective symmetry of the assembly (Figure 1A). The imine resonance at 8.83 ppm was accompanied by three bipyridine lines at 8.61, 8.26, and 7.49 ppm, two signals of pyridine at 8.37 and 8.02 ppm and two *p*‐phenylene doublets at 6.66 and 6.03 ppm. Two methylene groups resonated at 4.96 and 4.70 ppm, respectively.

**Figure 1:**
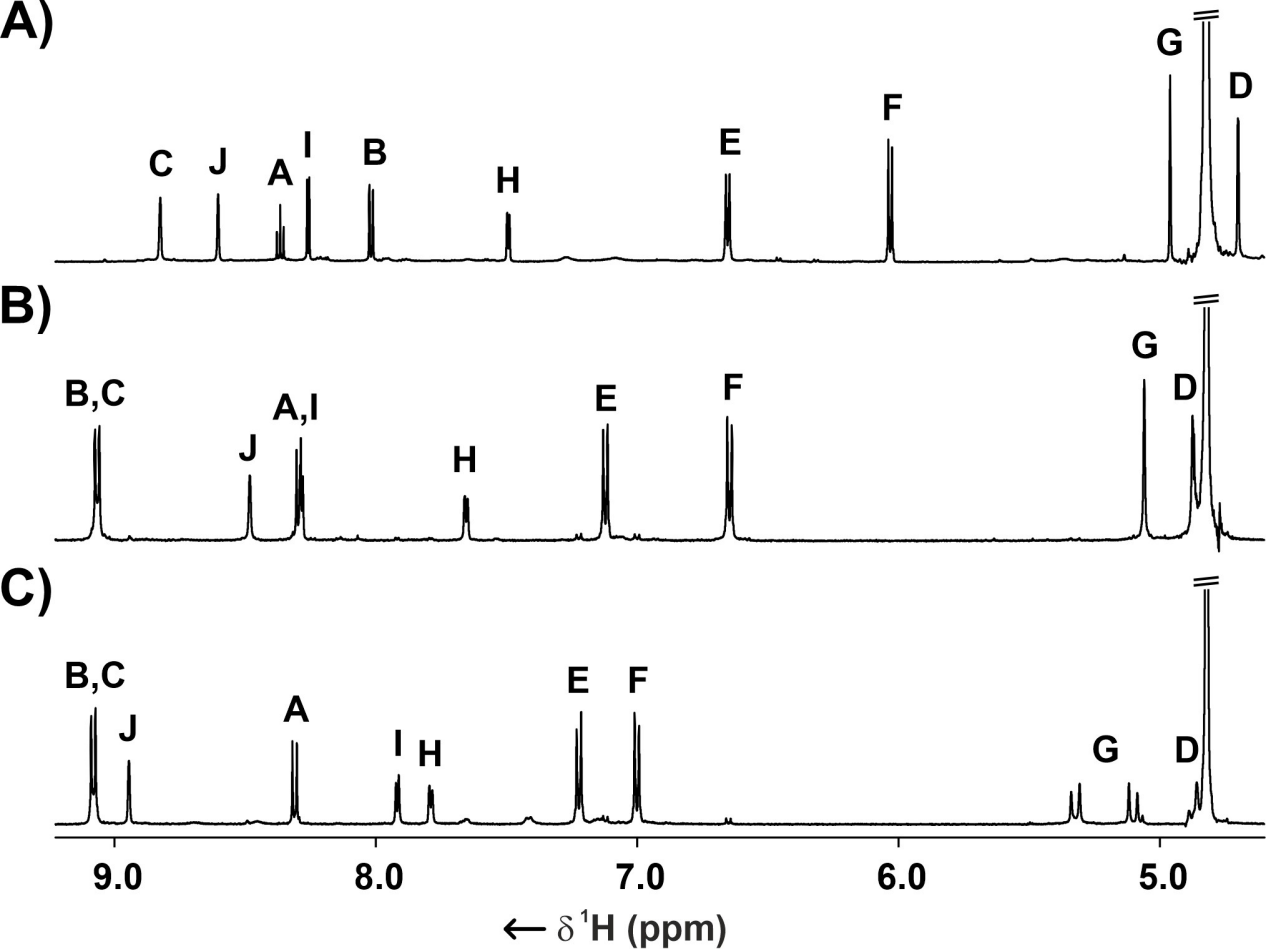
The ^1^H NMR (300 K, [D_4_]methanol, 600 MHz) of A) **3–2Ag_2_**, B) **6–2Ag_2_**, and C) **7–3Ag_2_**. Resonance labelling is shown in Scheme 1.

The identity of **3–2Ag_2_** was confirmed by XRD studies.^[38]^ The molecular structure revealed a dimeric assembly composed of two macrocycles formed upon the condensation of diamine **1** with 2,6‐diformylpyridine **2** (Figure 2A). The two C‐shaped macrocyclic components were arranged in a zig‐zag fashion in a way where the diiminopyridine of the first one interacted through the π⋅⋅⋅π stacking (ca. 3.4 Å) with bipyridyl of the other. The diiminopyridine and bipyridyl on both ends were engaged in the coordination of two separate silver(I) cations with the N_py_−Ag distance of 2.277(9) Å, N_im_−Ag bond length of 2.594(10)/2.65(1) Å, and N_bpy_−Ag equal to 2.297(9)/2.310(10) Å. Two Ag(I) centres differed in geometry, with the first one showing trigonal coordination originating from the binding to two nitrogens of bipyridyl and oxygen of trifluoroacetate and the second one demonstrating a distorted square pyramidal arrangement of three nitrogens of diiminopyridine, and two oxygens of two separate trifluoroacetates (O−Ag: 2.297(8), 2.454(7) Å). The carboxylate‐bridged silver cations in the [Ag⋅⋅⋅Ag]^2+^ units were separated by 2.976 Å moiety—a distance remaining in the range expected for argentophilic interactions (Figure S1).^[29]^ The molecular tweezer components interacted with neighbouring dimers through rhomboidal Ag−O−Ag−O units, propagating into a linear coordination polymer (Figure 2B).

**Figure 2:**
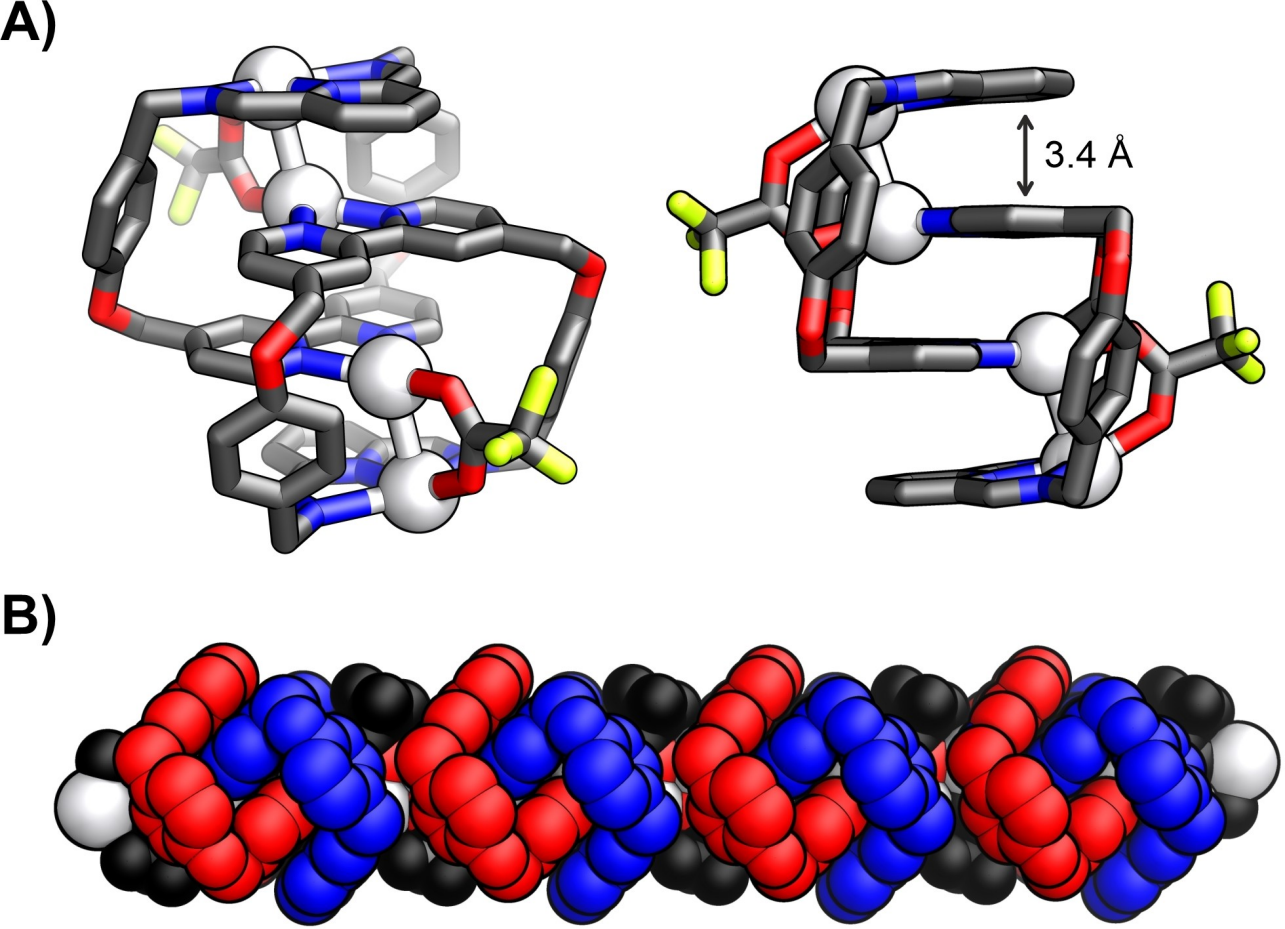
The X‐ray molecular structure of **3–2Ag_2_**. A) Front‐side (left) and side view (right); colour code: carbon—dark grey, oxygen—red, nitrogen—blue, fluorine—lime, silver—light grey. B) The coordination polymer with macrocycles in red and blue, Ag(I) in light grey and CF_3_COO^−^ in black. Protons, non‐coordinating anions and solvents were omitted for clarity.

The evaluation of ^1^H NMR and MS spectra recorded for mixtures incorporating **3–2Ag_2_** revealed the trace amounts of additional assemblies, i.e., tetranuclear Solomon link and trefoil knot **4–3Ag** (Figures S4, S41). Interestingly, the amount of the latter could be increased by carrying out the reaction at lower concentrations, but the selective formation of **4–3Ag** proved challenging (Figure S51). The confirmation of trefoil knot topology was based on the XRD studies. Unfortunately, due to the low quality of the X‐ray data, a thorough analysis could not be carried out, and the structural model provided only general, qualitative information regarding atom connectivity in **4–3Ag** (Figure S132). The model revealed the similarity of **4–3Ag** to the earlier reported Zn(II)/Cd(II),[^11^, ^39^] and Cu(I)^[40]^ assemblies. In particular, the knot architecture was stabilised by three distorted trigonal bipyramidal Ag(I) cations coordinating three diiminopyridine and two bipyridyl nitrogens each.

Intrigued by the **3–2Ag_2_** architecture, where the peculiar [Ag⋅⋅⋅Ag]^2+^ moieties were involved in the formation of the assembly, the use of silver(I) clusters in the construction of interlocked architecture was addressed. It was envisaged that the exploitation of such an unusual template would require finding a suitable ligand motif capable of stabilising the M⋅⋅⋅M units. The diimino‐1,8‐naphthyridine encompassing four nitrogen donors, with the N→M coordination vectors orientation promoting bis‐bidentate binding, was identified as a promising synthon.[^31^, ^32^, ^41^] Hence, the reaction of **1** was carried out with 2,7‐diformyl‐1,8‐naphthyridine **5** and silver(I) in *n*‐propanol at 70–95 °C (Scheme 1). The formation of Ag(I)‐incorporating imine architectures was confirmed by ESI MS for the reactions employing silver(I) triflate, acetate, trifluoroacetate and fluoride, with the ratio of products depending on the metal source. When excess AgOAc was used, the [2]catenane **6–2Ag_2_** (72 %) was formed as the sole product (see Supporting Information for details). In contrast, the use of AgOTf yielded mixtures containing, depending on the conditions, [2]catenane **6–2Ag_2_** (25 %) or trefoil knot **7–3Ag_2_** (8 %) as a major product (Scheme 1).

The elemental composition of **6–2Ag_2_** was established through MS (Figure S8). The ^1^H NMR signals assignment employed 2D NMR spectroscopy (Figures S52–62). The number of resonance lines suggested a high effective symmetry of the [2]catenane with the imine resonance at 9.06 ppm supplemented by two doublets of 1,8‐naphthyridine at 9.10 and 8.32 ppm (Figures 1B, S53). The bipyridyl signals were located at 8.41, 8.24 and 7.64 ppm, whereas *p*‐phenylene protons gave rise to two doublets at 7.13 and 6.64 ppm. Two singlets of methylene groups arose at 5.04 and 4.89 ppm.

The X‐ray crystallography confirmed the identity of **6–2Ag_2_** (Figure 3A).^[38]^ The catenane comprised two macrocyclic imines formed in the condensation of **5** and **1**. The diimino‐1,8‐naphthyridine acted as the bis‐bidentate ligand, incorporating the [Ag⋅⋅⋅Ag]^2+^ moieties at the poles of the assembly. The incorporation of the silver(I) pair required the rotation of pyridine rings of bipyridyl by 37.3/41.2°, enforcing the bis‐monodentate binding of Ag(I). Each Ag(I) cation was bound to three nitrogen atoms—one of imine, 1,8‐naphthyridine, and a twisted bipyridyl. The additional coordination of trifluoroacetates and methanol molecules resulted in silver cations adopting a slightly different geometry—from deformed trigonal to distorted tetrahedral and trigonal pyramidal. The 2.841/2.884 Å interatomic distances in the [Ag⋅⋅⋅Ag]^2+^ were in the limit of ligand‐supported argentophilic interactions.[^27^, ^29^, ^41^]

**Figure 3:**
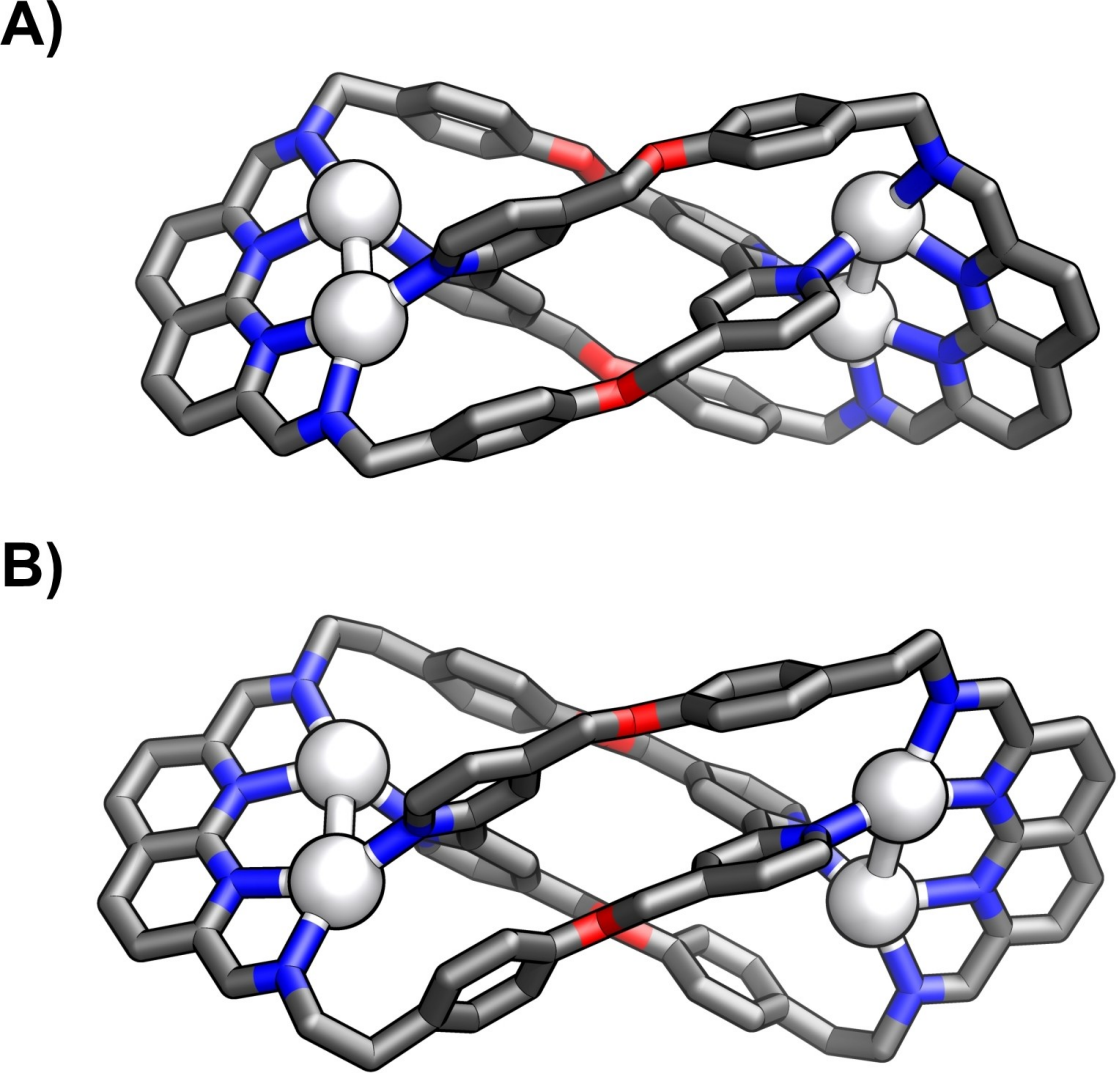
The X‐ray molecular structures of A) **6–2Ag_2_** and B) **9–2Ag_2_**. Protons, anions, and solvents were omitted for clarity.

The crystallisation of **7–3Ag_2_** proved challenging, and its identification was based on mass spectrometry and NMR spectroscopy (Figures S14, S71–81). The ESI MS confirmed the incorporation of six Ag(I) cations. The ^1^H NMR spectrum showed imine resonance at 9.08 ppm, overlapping with one of the naphthyridine doublets coupled to the other at 8.31 ppm. The bipyridyl resonances at 8.95, 7.92 and 7.79 ppm were accompanied by two *p*‐phenylene doublets at 7.22 and 7.00 ppm. The diastereotopically split methylene protons resonated in the 5.32–4.83 ppm region.

Perplexed by the variety of architectures formed from aldehydes **2** and **5** and diamine **1** and recognising that the topology of self‐assembled structures is often influenced by subtle factors such as the flexibility of their components, more pliable diamine **8** was designed and put to use for the Ag(I)‐templated self‐assembly (Scheme 2). The reaction of **8** with **5** in the presence of excess silver(I) triflate in *iso*‐propanol at 70 °C yielded the catenane **9–2Ag_2_** (35 %), analogous to **6–2Ag_2_**, as confirmed by ESI MS and NMR spectroscopy (Figures S16, S82–92). The crystallographic analysis confirmed that **9–2Ag_2_** retained most of the structural features of **6–2Ag_2_** (Figure 3B).^[38]^ Each [Ag⋅⋅⋅Ag]^2+^ unit was bound by bis‐bidentate diimino‐1,8‐naphthyridine and bis‐monodentate bipyridyl, but they differed in the coordination of additional trifluoromethanosulfonate and methanol/water. The Ag⋅⋅⋅Ag distances were slightly elongated, in comparison to **6–2Ag_2_**, reaching 3.015/3.008 Å.

**Scheme 2:**
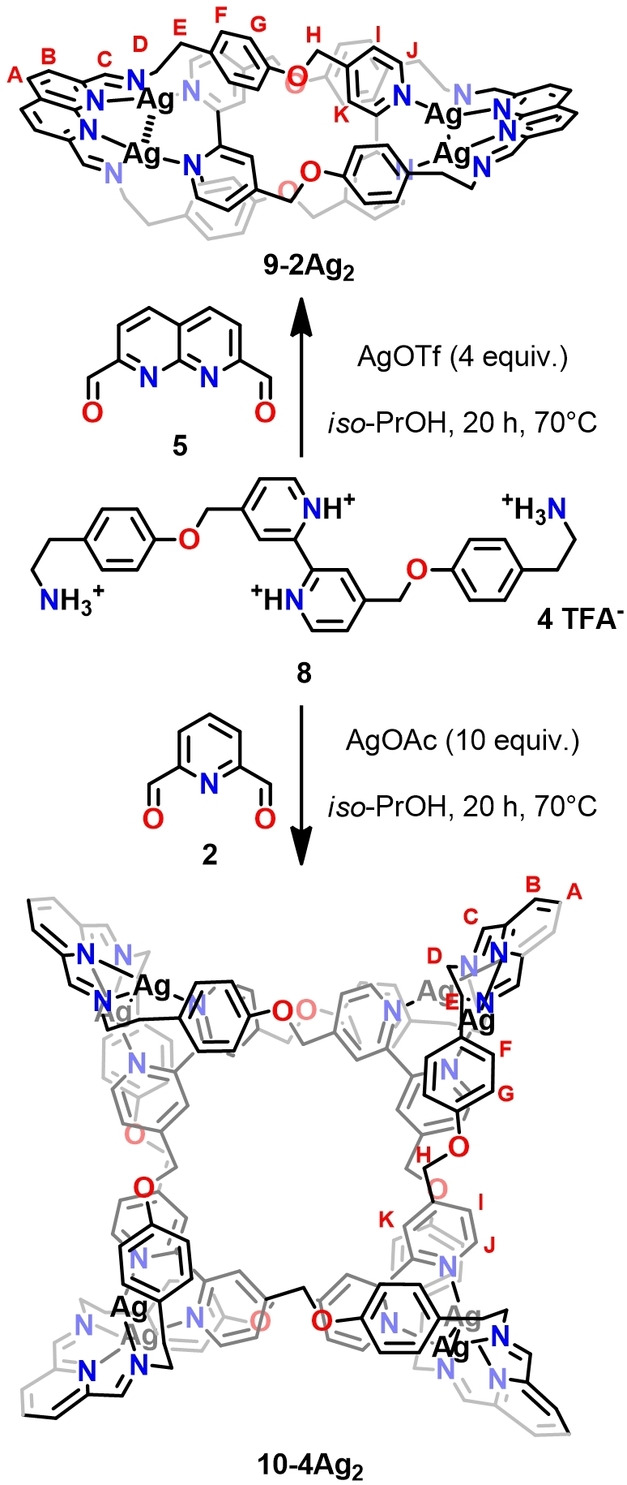
Synthesis of **9–2Ag_2_** and **10–4Ag_2_**. Anions and charge of the assemblies were omitted for clarity.

Surprisingly, when **8** was reacted with **2** and silver(I) acetate under conditions that had previously yielded the molecular tweezer **3–2Ag_2_**, a completely different architecture—Solomon link **10–4Ag_2_**—was obtained (18 %) (Scheme 2). The formation of a doubly interlocked [2]catenane[^42^, ^43^, ^44^] indicated that the increased flexibility of the subcomponent governed the self‐assembly towards the specific topology. Furthermore, once the reaction was carried out in the presence of potassium hexafluorophosphate or tetrafluoroborate, the clean formation of **X_n_⊂10–4Ag_2_** complexes (X=PF_6_^−^ (8 %), BF_4_^−^ (30 %)) was observed, indicating the role of anion templation in the self‐assembly of Solomon link.

The incorporation of eight Ag(I) cations into the Solomon link architecture was confirmed by ESI MS (Figure S20), and the structure elucidation was carried out based on the combination of ^1^H, ^13^C, ^19^F and 2D NMR (Figures S93–104). The ^1^H NMR spectrum of **10–4Ag_2_** showed a single imine resonance at 8.89 ppm accompanied by bipyridyl lines at 8.65, 8.35, and 7.99 ppm, pyridine ones at 8.35 and 8.08 ppm, and this of *p*‐phenylene at 6.89 ppm (Figure S93). The latter separated into two sets of signals at 7.10, 6.99, 6.88 and 6.62 ppm upon lowering the temperature to 190 K, correspondingly with the slower rotation of the phenylene rings (Figure S95). The propensity of Solomon link for anion encapsulation was corroborated employing ^1^H‐^19^F HOESY (heteronuclear Overhauser effect spectroscopy) which demonstrated a series of correlation peaks between the fluorine nuclei of the intracavity PF_6_^−^/BF_4_^−^ and bipyridyl (K), phenylene (F, G), and methylene (H) protons in **X_n_⊂10–4Ag_2_** complexes (Figures 4, S119, S131).

**Figure 4:**
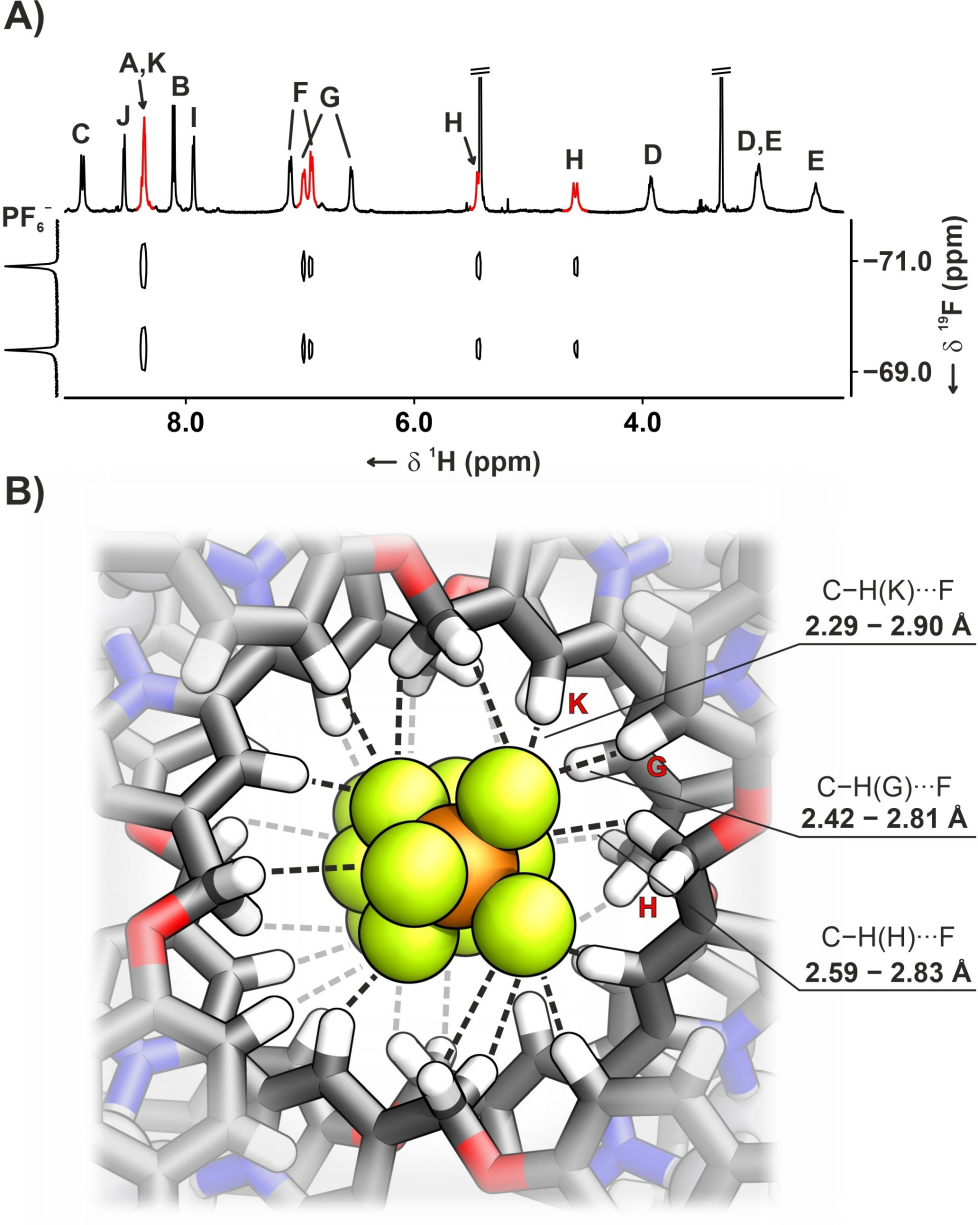
A) The ^1^H‐^19^F HOESY NMR spectrum of **(PF_6_)_2_** ⊂ **10–4Ag_2_** (230 K, [D_4_]methanol, 500 MHz), and B) the cavity‐centred region of the complex with HB shown with dashed lines.

The X‐ray molecular structure of **(PF_6_)_2_** ⊂ **10–4Ag_2_** revealed a doubly interlocked [2]catenane comprising two equivalent, helically twisted, 14×28 Å‐sized tetraimine macrocycles formed from 2 equivalents of **8** and **2** (Figure 5).^[38]^ The macrocycles were held together due to the coordination of four [Ag^I^⋅⋅⋅Ag^I^] moieties, demonstrating an unusual binding mode. Each silver(I) in every pair was coordinated to one imine nitrogen of diiminopyridine (Ag−N_im_: 2.220(6)–2.265(4) Å) and one of bis‐monodentate bipyridyl (Ag−N_bpy_: 2.224(5)–2.304(4) Å). The Ag−N distance to heterocyclic nitrogen of diiminopyridine varied in the 2.545(5)–2.707(6) Å range. Additional ligands, i.e., water and methanol, were found binding to silver(I) centres. The relatively close distance between silver(I) and the nearby aromatic rings (2.70–2.90 Å) suggested a weak *η* ^1^‐Ag−C π‐coordination.[^45^, ^46^] Two hexafluorophosphate anions were found encapsulated in the cavity, stabilised through a network of C−H⋅⋅⋅F hydrogen bonds (CH−F: 2.29–2.90 Å).

**Figure 5:**
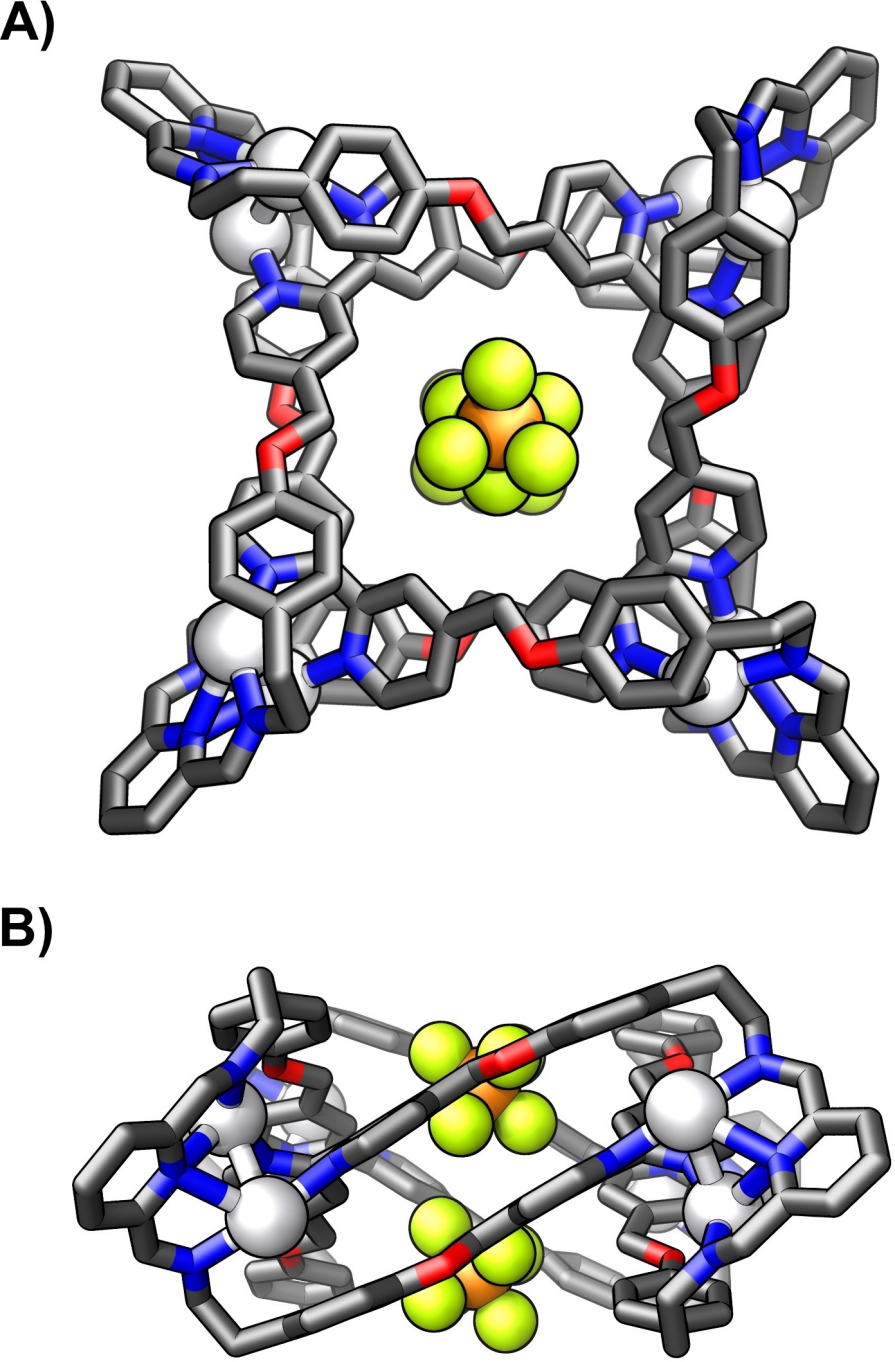
The X‐ray molecular structure of **(PF_6_)_2_** ⊂ **10–4Ag_2_**. Protons, non‐encapsulated anions, and solvents were omitted for clarity.

The ^1^H‐^109^Ag HMBC (heteronuclear multiple bond correlation) spectra for **6–2Ag_2_**, **7–3Ag_2_**, **9–2Ag_2_**, and **10–4Ag_2_** were recorded to assess the chemical shifts of the ^109^Ag nuclei (Figures S70, S79, S90, S102). The ^109^Ag resonances consistently appeared within the 500–550 ppm range, indicating a shared coordination motif across the compounds and revealing only a slight dependence of the silver(I) chemical shift on the topology of the assembly.

Identification of the close Ag⋅⋅⋅Ag contacts in the silver(I)‐stabilised links and knots prompted the evaluation of their photoluminescent properties, as many d^10^‐metal coordination compounds demonstrated ligand‐centered, charge‐transfer, or, in the case of polynuclear compounds, even metal‐centered emission.^[47]^ Considering the synthetic accessibility and stability of the assemblies, the experimental and computational solid‐state luminescence studies were carried out for **9–2Ag_2_** and **(PF_6_)_2_** ⊂ **10–4Ag_2_**, incorporating four and eight silver(I) cations, respectively. The [2]catenane **9–2Ag_2_** exhibited red broadband emission with a maximum at ca. 625 nm upon excitation at the band peaking at about 545 nm (Figure S133). Since this emission showed a very short decay time (Figure S135), it was expected to result from ligand‐to‐metal charge transfer (LMCT) interactions. The time‐dependent density functional theory calculations and the analysis of natural transition orbitals for **9–2Ag_2_** implied that this assignment is indeed relevant, as all three major excitations have an LMCT character (Figure S138). In contrast, **(PF_6_)_2_** ⊂ **10–4Ag_2_** demonstrated three emission bands in the solid state with maxima at 279, 395, and 553 nm (Figure S134) and relevant three absorption bands peaking at about 254, 312, and 480 nm. The high‐energy emission in the UV region showed a decay time of 115.9 ns, while the subsequent two broad emissions′ decay times were 110 μs (395 nm) and 47.5 μs (553 nm) (Figure S136). The simulated UV/Vis spectrum of **(PF_6_)_2_** ⊂ **10–4Ag_2_** also displayed three major absorption bands at similar energies/wavelengths of 230, 324, and 458 nm. Analysis of orbital contributions showed both ligand‐to‐ligand and metal‐to‐ligand charge transfers driving these transitions (Figures S139–141). The most energetic absorption and emission bands primarily involved the S_1_↔S_0_ transitions, which also displayed LMCT character according to computational results (Figure S141).

The self‐assembly of pyridine‐ and 1,8‐naphthyridine‐based aldehydes, bipyridyl‐incorporating diamines and silver(I) resulted in the formation of four distinct topologies, stabilised by the [Ag^I^⋅⋅⋅Ag^I^] moieties. The reactions carried out using 2,6‐diformylpyridine yielded, depending on the flexibility of the diamine component, the molecular tweezer, trefoil knot or Solomon link. The self‐assembly in the presence of 2,7‐diformyl‐1,8‐naphthyridine produced Ag(I)‐stabilised [2]catenane and trefoil knot. The unusual [Ag^I^⋅⋅⋅Ag^I^] moieties stabilising the assemblies constitute an exciting extension of a classic metal template concept where not a single cation, but rather a simple metal cluster acted to organise the organic ligands into the intricate architecture. It is believed that further work on the cluster‐templated systems will generate an exciting class of assemblies demonstrating unique luminescence or magnetic properties.

## Supporting Information

The authors have cited additional references within the Supporting Information.[^48^, ^49^, ^50^, ^51^, ^52^, ^53^, ^54^, ^55^, ^56^, ^57^, ^58^, ^59^, ^60^, ^61^, ^62^]

## Conflict of Interests

The authors declare no conflict of interest.

## Supporting information

As a service to our authors and readers, this journal provides supporting information supplied by the authors. Such materials are peer reviewed and may be re‐organized for online delivery, but are not copy‐edited or typeset. Technical support issues arising from supporting information (other than missing files) should be addressed to the authors.

Supporting Information

Supporting Information

## Data Availability

The data that support the findings of this study are available in the supplementary material of this article.
