# Supplementary material for: The Interplay Between Component Denticity and Flexibility Promotes the Formation of [AgI⋅⋅⋅AgI]‐stabilised Links and Knots
Source: Angew Chem Int Ed Engl. 2025 Jan 12;64(16):e202423962. doi: 10.1002/anie.202423962 (PMC12001158; doi:10.1002/anie.202423962)
Supplement: Supplementary file 2 — Supporting Information [file ANIE-64-e202423962-s001.pdf]

Angewandte Chemie
Eine Zeitschrift der Gesellschaft Deutscher Chemiker

Supporting Information

The Interplay Between Component Denticity and Flexibility Promotes the Formation of [$Ag^{I}\cdots Ag^{I}$]-stabilised Links and Knots

*A. Sarwa, A. Khmara, K. A. Konieczny, D. Kulesza, E. Zych, B. Trzaskowski, B. Szyszko**

Supporting Information
©Wiley-VCH 2021
69451 Weinheim, Germany

The Interplay Between Component Denticity and Flexibility Promotes the Formation of [$Ag^{I}\cdots Ag^{I}$]−stabilised Links and Knots

Aleksandra Sarwa, Andrei Khmara, Krzysztof A. Konieczny, Dagmara Kulesza, Eugeniusz Zych, Bartosz Trzaskowski, Bartosz Szyszko*

Abstract: A subtle interplay between the flexibility of the 2,2'-bipyridyl-based diamine and the denticity of the coordination domain formed upon self-assembly enabled the formation of four distinct topologies stabilised by $[Ag\cdots Ag]^{2+}$ pairs. The reactions utilising 2,6-diformylpyridine resulted in the formation of silver(I)-stabilised molecular tweezer, trefoil knot, and Solomon link. The 1,8-naphthyridine-based dialdehyde promoted the formation of [2]catenanes and trefoil knot, demonstrating very close $Ag^{I}\cdots Ag^{I}$ distances. Two studied assemblies demonstrated interesting luminescent properties in the solid state.

DOI: 10.1002/anie.2021XXXXX

Table of Contents

Table of Contents 2
Experimental Procedures 3
NMR spectroscopy 3
Mass spectrometry 3
Luminescence spectroscopy 3
Computational methods 3
X-ray diffraction data 3
Synthesis 6
Molecular Tweezer 3-2Ag_2 8
Trefoil Knot 4-3Ag 8
[2]Catenane 6-2Ag_2^{OTf} 9
[2]Catenane 6-2Ag_2^{OAc} 9
Trefoil Knot 7-3Ag_2 10
[2]Catenane 9-2Ag_2 10
Solomon Link 10-4Ag_2 11
Solomon Link $(PF_6)_2 \subset$ 10-4Ag_2 11
Solomon Link $(BF_4)_n \subset$ 10-4Ag_2 12
High-resolution Mass Spectra 13
Mass spectra of molecular tweezer 3-2Ag_2 13
Mass spectra of the mixture of molecular tweezer, trefoil knot with traces of Solomon link 14
Mass spectra of [2]catenane 6-2Ag_2^{OTf} 16
Mass spectrum of [2]catenane 6-2Ag_2^{OAc} 19
Mass spectra of trefoil knot 7-3Ag_2 20
Mass spectra of [2]catenane 9-2Ag_2 21
Mass spectra of Solomon link 10-4Ag_2 23
Mass spectra of Solomon link $(PF_6)_2 \subset$ 10-4Ag_2 24
Mass spectra of Solomon link $(BF_4)_n \subset$ 10-4Ag_2 25
NMR spectra 26
NMR spectra of NBoc amine S4 and its salt 8 26
NMR spectra of molecular tweezer 3-2Ag_2 29
NMR spectra of mixtures containing trefoil knot 4-3Ag 35
NMR spectra of [2]catenane 6-2Ag_2^{OTf} (obtained with silver(I) triflate). 41
NMR spectra of [2]catenane 6-2Ag_2^{OAc} (obtained with silver(I) acetate). 47
NMR spectra of trefoil knot 7-3Ag_2 51
NMR spectra of [2]catenane 9-2Ag_2 58
NMR spectra of Solomon link 10-4Ag_2 65
Titration of the Solomon Link 10-4Ag_2 with KPF_6. 72
NMR spectra of Solomon link $(PF_6)_2 \subset$ 10-4Ag_2 74
NMR spectra of Solomon link $(BF_4)_n \subset$ 10-4Ag_2 82
The structural model of 4-3Ag based on poor-quality X-ray data 88
Luminescence studies 89
Computational results 91
Cartesian coordinates 95
References 99

Experimental Procedures

NMR spectroscopy

The NMR spectra were recorded on high-field spectrometers: 600.15 MHz equipped with broadband inverse and observe gradient probes and 500.16 MHz equipped with broadband observe gradient probe. The ^{1}H and ^{13}C NMR spectra were referenced to the residual solvent signal of [D$_4$]methanol (^{1}H NMR: $\delta = 3.31$ ppm, ^{13}C NMR: $\delta = 49.0$ ppm). The ^{109}Ag NMR signals in the ^{1}H-^{109}Ag HMBC spectra were referenced to a 4 M solution of $AgNO_3$ in D_2O (22.00 ppm).[48] The ^{19}F NMR spectra were referenced to hexafluorobenzene C_6F_6 in [D$_4$]methanol ^{19}F NMR: $\delta = -165.37$ ppm).

Mass spectrometry

The mass spectra were recorded on Bruker qTOF compact spectrometer.

Luminescence spectroscopy

Photoluminescence and luminescence excitation spectra were recorded with an FLS 1000 Spectrometer from Edinburgh Instruments Inc. Excitation was executed with an Ozone-free Xe lamp, and the emission was recorded with PMT-980 photomultiplier operating in the 185–900 nm range and thermoelectrically cooled to −20 °C with a Peltier module. The double-grating 2x 325 mm Czerny-Tuners excitation and emission monochromators were utilised in the excitation and emission channels. PLE spectra were corrected for the incident light intensity and PL spectra for the wavelength dependence of the spectral response of the recording channel. Decay curves were recorded using the same Spectrometer upon Xe pulse lamp excitation for microsecond decays and an EPLED-250 (250 nm) pulsed laser for nanosecond decays. A TCSPC technique and a high-speed, low-noise F-G05 detector featuring a Hamamatsu H5773-04 photomultiplier were used for the latter.

Computational methods

In the computational part of this study, the geometry optimisation was performed, followed by frequency calculations and excited state calculations for two systems. For **9-2Ag$_2$**, the time-dependent density functional theory (TD-DFT) employing the B3PW91 functional with the LanL2DZ basis set was used, as in the study of other groups on a silver(I) coordination compounds [46,49-51]. First, the optimisation of the geometry was performed, followed by frequency calculations to assess whether the system is in the minimum. Next, TD-DFT calculations were carried out using the same method, basis set, and the polarisable continuum model with methanol as the solvent. Unfortunately due to the size of **$(PF_6)_2 \subset$ 10-4Ag$_2$**, such an approach was not possible; therefore, the GFN2-xTB semi-empirical extended tight-binding method was obtained to obtain the optimised geometry. In that case, the simplified Tamm-Dancoff Approximation was exploited to study excited states[52,53]. In both cases, the system was modelled in methanol using the generalised Born model with surface area contributions. All molecular orbital visualisations were generated using an isosurface value of 0.03.
The Meyer and Wiberg bord indices between Ag atoms were calculated to quantify the potential argentophilic interactions. The obtained average values were equal to 0.10 (Meyer) and 0.22 (Wiberg) for **9-2Ag$_2$**, and 0.15 (Meyer) and 0.22 (Wiberg) for **$(PF_6)_2$10-4Ag$_2$**. These values are similar to the bond order reported for binuclear silver(I) coordination compounds of diiminonaphthyridine and lower than bond orders obtained for previously synthesized pyrrole cages encapsulating silver(I) clusters.[27,47,54]

X-ray diffraction data

The data was collected for all crystals at 100 K on a Rigaku XtalLAB Synergy R diffractometer equipped with a HyPix-Arc 150 HPAD detector and Cu-Kα rotating anode. The diffraction images were processed in CrysAlisPro ver. 1.171.42.74a.[55] All structures were solved by ShelXT[55] and refined by a ShelXL full matrix least-square method on F^2[56] using the Olex2 software suit.[57] Selected crystallographic data was gathered in Table S1.

9-2Ag$_2$
The Gaussian absorption corrections from crystal shape were applied. All non-hydrogen atoms were refined anisotropically except minor components in certain disordered fragments. Hydrogen atoms were constrained based on their corresponding positions using the riding model. The positive charge of the complex was equalised by the presence of triflate anions for which the disorder was modelled whenever necessary. The position of the Ag4 cation was disordered over two occupation sites followed by disorder in the organic part of the complex (from C55 to N7), which were found to be correlated (of 0.55) and are plausibly a result of a substitutional disorder of the solvents coordinated to Ag4. A set of restraints on bond lengths, valence angles and atomic displacement parameters,

such as DFIX, DANG, SIMU, SADI and RIGU, were applied for disorder modelling purposes. The structure was deposited in CCDC with deposition **#2380730**.

6-2Ag$_2$

Absorption corrections and crystal structure determination procedure were analogous to **9-2Ag$_2$**. The charge of the complex was equalised by triflate anions. The structure was deposited in CCDC with deposition **#2380731**.

3-2Ag$_2$

The data was processed as a two-component non-merohedral twin with a scale factor equal to 0.7028(18) for a major component, and multi-scan absorption corrections were applied. The structure was determined based on the twinned data using the HKLF 5 command. All non-hydrogen atoms were refined anisotropically, and all hydrogen atoms were placed geometrically using the riding model or riding rotating model for methanol molecules. The charge of the complex was equalised by trifluoroacetate and trifluoroacetate/acetate substitutionally disordered anions. The solvent molecules were not refined; however, due to twinning, the solvent masking was not applied since it did not improve the structure quality. The structure was deposited in CCDC with deposition **#2380732**.

$(PF_6)_2 \subset$ 10-4Ag$_2$

The multi-scan absorption corrections were applied. All non-hydrogen atoms were refined anisotropically, and all hydrogen atoms were constrained using the riding model. The masking algorithm (AKA squeeze) by Olex2[58] was applied to handle part of the disorder within the structure, including solvent molecules and some coordination sites of silver cations. The nature of the charge distribution within the crystal remains non-resolved, and positions of a few hexafluorophosphate anions were refined only if their presence was unambiguous and for which SADI, DFIX and RIGU restraints were applied. The structure was deposited in CCDC with deposition **#2380733**.

4-3Ag

The multi-scan absorption corrections were applied. The crystal was at least a 4-component non-merohedral twin. However, the data was reduced to a 2-component twin, taking into account two major components only. The structure was solved based on the single component data deconvoluted from twinned data. The structure was solved in the monoclinic *C*2/c space group. Refinement with the masking algorithm unambiguously confirmed the formation of a trefoil knot. However, the overall data quality does not allow us to determine the crystal structure's correctness on the crystal system's level with certainty. The data was not deposited within CCDC.

Table S1 Selected crystallographic data.

Structure	**9-2Ag$_2$**	**6-2Ag$_2$**	**3-2Ag$_2$**	**$(PF_6)_2 \subset$ 10-4Ag$_2$**
Crystal system	monoclinic	monoclinic	Triclinic	Monoclinic
Space group	$P2_1/c$	$P2_1/c$	P-1	I2/a
Z, Z’	4, 1	4, 1	1, 0.5	8, 1
a [Å]	22.18800(1)	20.73090(16)	12.3784(6)	36.6407(4)
b [Å]	26.5056(2)	27.4146(2)	12.7842(8)	24.46649(19)
c [Å]	15.58070(10)	15.12761(14)	13.1766(3)	42.0712(4)
α [°]	90	90	92.082(4)	90
β [°]	107.2830(1)	93.5950(10)	89.907(3)	99.6149(9)
γ [°]	90	90	96.686(5)	90
Volume [Å^3]	8749.38(11)	8580.55(12)	2069.61(17)	37185.7(6)
D_x [g/cm^3]	1.742	1.769	1.546	SQUEEZE applied
Reflections collected	219948	63396	30077	193956
Reflections independent	17973	16675	30077	37946
Reflections observed	17002	14517	23760	26192
Completeness [%]	99.9	99.7	100	99.8
R_{int}	0.0375	0.0230	0.1132	0.0768

R [$F^2 > 2\sigma(F^2)$], wR, S	0.045, 0.1106, 1.091	0.048, 0.1117, 1.064	0.1155,0.3337, 1.093	0.0662, 0.1939, 1.120
$\Delta\rho_{max}$, $\Delta\rho_{min}$ [eÅ^{-3}]	1.077,-1.102	0.859,-1.293	3.029,-1.658	1.140, -1.053

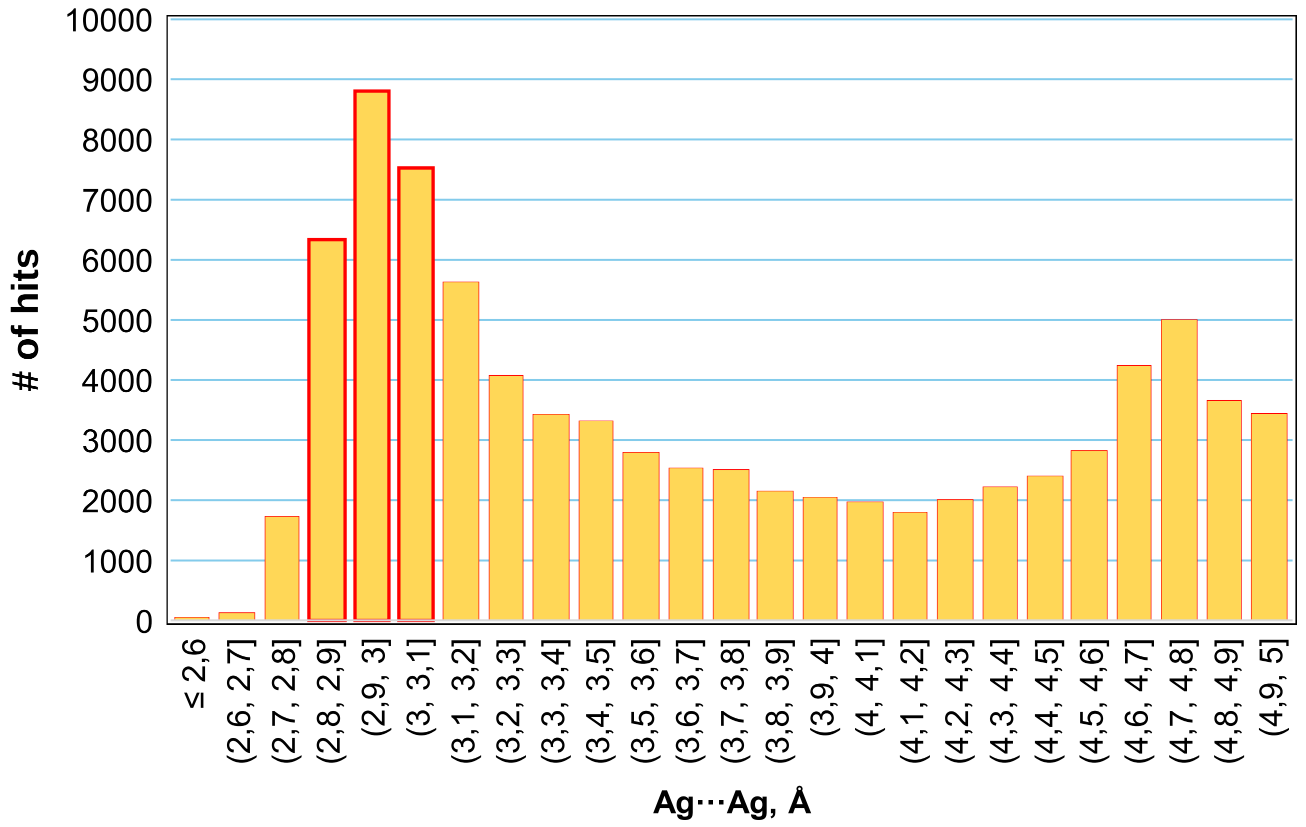

Figure S1. Ag···Ag distance distribution below 5 Å based on a search in Cambridge Structural Database ver. 2023.3.0. From all 23373 structures containing Ag, 9907 structures contain contact between two silver entities lower than 5 Å. All structures published within this work belong to ranges marked with thicker red lines.

Synthesis

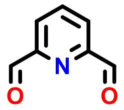

2

2,6-Diformylpyridine 2 was synthesised as described in the literature.[59]

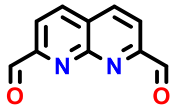

5

2,7-Diformylnaphthyridine 5 was synthesised as described in the literature, starting from 2-methylnaphthyridine.[60]

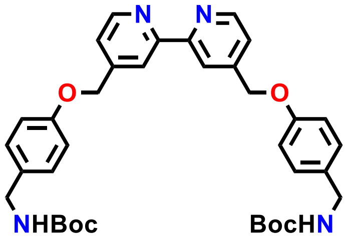

S1

NBoc-protected amine S1 was synthesised as described in the literature.[11]

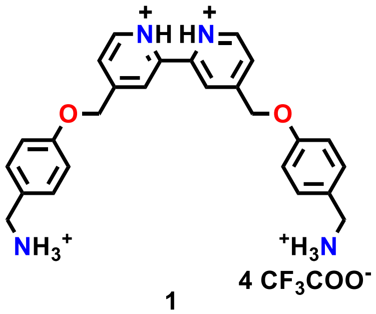

1

Trifluoroacetic salt of amine **1** was synthesised as described in the literature.[11]

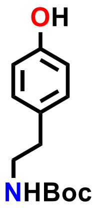

S2

N-Boc-2-(4-hydroxyphenyl)ethylamine S2 was synthesized as described in the literature.[61]

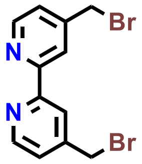

S3

4,4'-Bis(bromomethyl)-2,2'-bipyridine S3 was synthesized as described in the literature.[62]

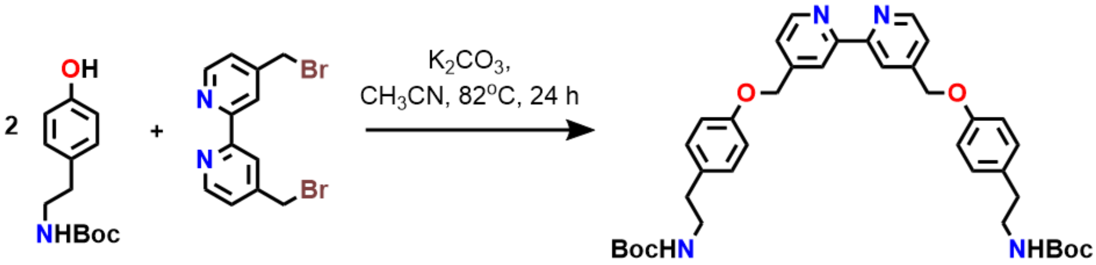

S2 **S3** **S4**

NBoc-protected amine S4 was synthesised via the modified procedure described in the literature.[11]

4,4'-Bis(bromomethyl)-2,2'-bipyridine **S3** (265 mg, 0.77 mmol) was added to a solution of **S2** (373 mg, 1.67 mmol) and anhydrous K_2CO_3 (450 mg, 3.3 mmol) in anhydrous acetonitrile (10 mL) under nitrogen atmosphere in a 25 mL two-neck round bottom flask. The reaction mixture was heated at 82°C for 24 hours. After that, the solvent was removed under reduced pressure, 50 mL of ethyl acetate was added, and the suspension after sonication was transferred to the separatory funnel and washed with water (3 × 50 mL) and brine (50 mL). The organic layer was dried over magnesium sulfate and evaporated to dryness. Then, 20 mL of cold ethyl acetate was added and sonicated. The solid was separated *via* filtration and dried under vacuum to afford **S4** as a light-yellow powder (310 mg, 62%).

^{1}H NMR (600 MHz, [D]chloroform, 300 K) δ (ppm): 8.67 (d, ^3J = 5.0 Hz, 2H, bpy), 8.44 (s, 2H, bpy), 7.41 (d, ^3J = 5.0 Hz, 2H, bpy), 7.10 (d, ^3J = 8.1 Hz, 4H, phen), 6.91 (d, ^3J = 8.2 Hz, 4H, phen), 5.15 (s, 4H, OCH_2), 4.50 (br, 2H, NH), 3.36 – 3.26 (m, 4H, CH_2**CH_2**N), 2.72 (t, ^3J = 7.0 Hz, 4H, C**H_2**CH_2N), 1.41 (s, 18H, Boc).
^{13}C NMR (151 MHz, [D]chloroform, 300 K) δ (ppm): 156.9, 156.2, 155.9, 149.6, 147.5, 131.9, 129.9, 121.7, 119.0, 115.0, 79.2, 68.5, 41.9, 35.3, 28.4.

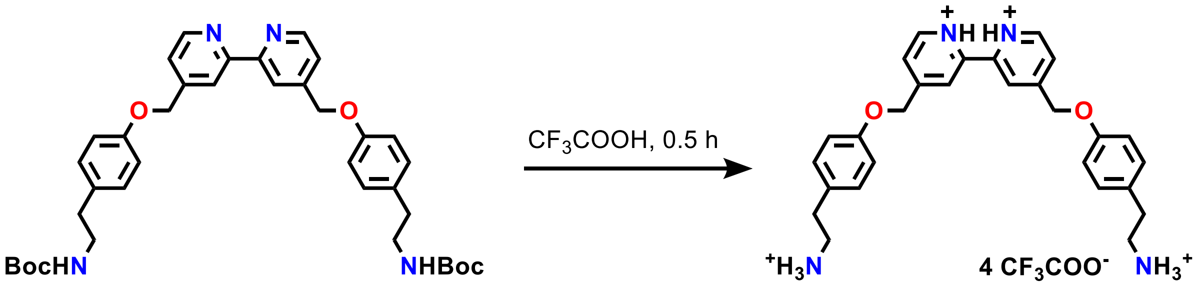

S4 **8**

Trifluoroacetic salt of 8 was obtained using trifluoroacetic acid to remove the Boc protecting group from **S4**.

Boc-protected diamine S4 (15 mg, 0.023 mmol) was dissolved in 1.5 mL of trifluoroacetic acid (TFA) in a 25 mL round-bottom flask and stirred at room temperature for 30 minutes. Excess of TFA was removed by evaporation under reduced pressure, followed by subsequent additions and removals of MeOH (5 × 10 mL). Compound **8** was obtained as pink oil.

^{1}H NMR (500 MHz, [D$_4$]methanol, 300 K) δ (ppm): 8.79 (d, ^3J = 5.3 Hz, 2H, bpy), 8.60 (s, 2H, bpy), 7.82 (d, ^3J = 5.3 Hz, 2H, bpy), 7.25 (d, ^3J = 8.5 Hz, 4H, phen), 7.08 (d, ^3J = 8.5 Hz, 4H, phen), 5.37 (s, 4H, OC**H_2**), 3.14 (t, ^3J = 7.5 Hz, 4H, CH_2C**H_2**N), 2.91 (t, ^3J = 7.5 Hz, 4H, C**H_2**CH_2N).
^{13}C NMR (151 MHz, [D$_4$]methanol, 300 K) δ (ppm): 161.8 (q, $^3J_{C,F}$ = 35.0 Hz), 158.6, 155.5, 150.2, 147.9, 131.1, 125.4, 121.9, 117.5 (q, $^2J_{C,F}$ = 291.0 Hz), 116.5, 68.9, 42.0, 33.7.
^{19}F NMR (565 MHz, [D$_4$]methanol, 300 K) δ (ppm): –77.3.

Molecular Tweezer 3-2Ag$_2$

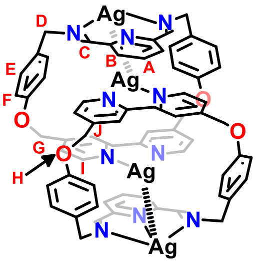

Deprotection of Boc-protected amine **S1** was performed directly before carrying self-assembly. After removing the excess of TFA with methanol, **1** was washed with diethyl ether. Afterwards, **1** (19 mg, 0.022) mmol) was dissolved in *iso*-PrOH and sonicated for 5 minutes. Then AgOAc (36 mg, 0.22 mmol) and 2,6-diformylpyridine **2** (4.4 mg, 0.024 mmol) were added, and the mixture was sonicated for another 5 minutes. The mixture was then stirred at 70ºC for 20 hours. The orange precipitate was separated using a centrifuge, and the filtrate was evaporated to dryness. Then, 2 mL of methanol was added, followed by ca. 10 mL diethyl ether to precipitate the **3-2Ag$_2$**. The product was separated and dried *in vacuo*. Yield: 0.5 mg (2%).

^{1}H NMR (600 MHz, [D$_4$]methanol, 300 K) δ (ppm): 8.83 (t, ^3J = 1.8 Hz, 4H, C), 8.61 (s, 4H, J), 8.37 (t, ^3J = 7.7 Hz, 2H, A), 8.26 (d, ^3J = 5 Hz, 4H, I), 8.02 (d, ^3J = 7.7 Hz, 4H, B), 7.49 (d, ^3J = 5 Hz, 4H, H), 6.67 – 6.64 (m, 8H, E), 6.05 – 6.02 (m, 8H, F), 4.96 (s, 8H, G), 4.70 (d, J = 1.6 Hz, 8H, D).
^{13}C NMR (151 MHz, [D$_4$]methanol, 300 K) δ (ppm): 160.3, 158.0, 153.0, 151.6, 151.0, 149.7, 141.7, 132.6, 131.0, 130.3, 124.6, 122.6, 114.9, 68.0, 64.1. *Not all the signals in the ^{13}C NMR spectrum were assigned due to high broadening.*
^{19}F NMR (471 MHz, [D$_4$]methanol, 300 K) δ (ppm): –76.7.
HR-MS (ESI+, TOF): *m/z* 1821.0127 calcd. for $[C_{66}H_{54}N_{10}O_4Ag_4(CF_3COO)_3]^+$ 1821.0086.

Trefoil Knot 4-3Ag

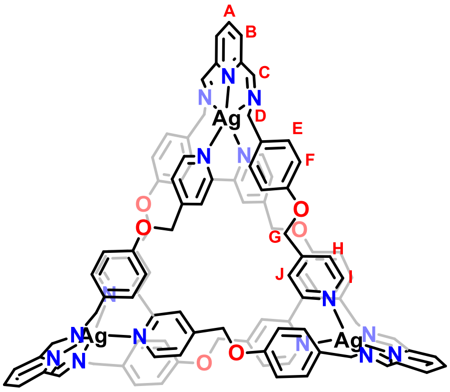

The traces of trefoil knot **4-3Ag** were observed in the product mixture from the synthesis of **3-2Ag$_2$**, which was carried out in 10-100 mL (2.4 – 0.2 mM amine concentration) of *iso*-propanol.

HR-MS (ESI+, TOF): *m/z* 1006.6764 calcd. for $[C_{99}H_{81}N_{15}O_6Ag_3(CF_3COO)]^{2+}$ 1006.6743.

[2]Catenane 6-2Ag$_2^{OTf}$

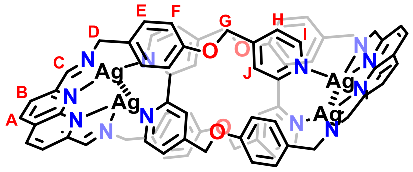

Deprotection of Boc-protected amine S1 was performed directly before carrying self-assembly. After removing the excess of TFA with methanol, the oily residue 1 (15 mg, 0.017 mmol) was dissolved in 15 mL of n-propanol, and after complete dissolution of the salt 1, AgOTf (9 mg, 0.034 mmol) and 2,7-diformyl-1,8-naphthyridine 5 (3.2 mg, 0.017 mmol) were added. After the complete dissolution of reagents, the mixture was stirred at 70ºC under a reflux condenser for 16 hours. The yellow precipitate was separated using a centrifuge, washed with 10 mL of *iso*-propanol, and dried *in vacuo*. Then 10 mL of methanol was added to the solid, and the yellow solution was separated and concentrated to ca. 2 mL before diethyl ether was added to precipitate the product. The solid was then filtered and dried *in vacuo*. Yield: 4 mg (25%).

^{1}H NMR (600 MHz, [D$_4$]methanol, 240 K) δ (ppm): 9.10 (d, ^3J = 8.3, 4H, B), 9.06 (d, ^3J = 8.3, 4H, C), 8.41 (s, 4H, J), 8.32 (d, ^3J = 8.5 Hz, 4H, A), 8.24 (d, ^3J = 5.2 Hz, 4H, I), 7.64 (d, ^3J = 5.2 Hz, 4H, H), 7.13 (d, ^3J = 8.3 Hz, 8H, E), 6.64 (d, ^3J = 8.3 Hz, 8H, F), 5.04 (s, 8H, G), 4.89 (s, 8H, D).
^{13}C NMR (151 MHz, [D$_4$]methanol, 300 K) δ (ppm): 161.2, 158.2, 156.5, 156.2, 153.6, 151.8, 143.6, 134.6, 131.5, 129.4, 127.6, 124.5, 123.3, 121.9, 116.1, 67.4, 62.2. *Not all the signals in the ^{13}C NMR spectrum were assigned due to high broadening.*
^{19}F NMR (565 MHz, [D$_4$]methanol, 300 K) δ (ppm): –80.0.
HR-MS (ESI+, TOF): *m/z* 577.6739 calcd. for $[C_{72}H_{56}N_{12}O_4Ag_4(CF_3SO_3)]^{3+}$ 577.6754; 905.0185 calcd. for $[C_{72}H_{56}N_{12}O_4Ag_4(CF_3CO_2)_2]^{2+}$ 905.0224; 923.0013 calcd. for $[C_{72}H_{56}N_{12}O_4Ag_4(CF_3SO_3)(CF_3CO_2)]^{2+}$ 923.0059; 940.9854 calcd. for $[C_{72}H_{56}N_{12}O_4Ag_4(CF_3SO_3)_2]^{2+}$ 940.9893.

[2]Catenane 6-2Ag$_2^{OAc}$

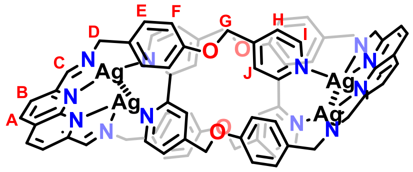

*Due to the low solubility and stability of **6-2Ag$_2^{OTf}$**, the analogous reaction was performed using AgOAc in modified conditions.*

Deprotection of Boc-protected amine **S1** was performed directly before carrying self-assembly. After removing the excess of TFA with methanol and washing with diethyl ether, the oily residue **1** (21.1 mg, 0.024 mmol) was dissolved in 10 mL of *iso*-PrOH. Next, AgOAc (16 mg, 0.096 mmol) and 2,7-diformyl-1,8-naphthyridine **5** (4.7 mg, 0.018 mmol) were added. The suspension was sonicated for 5 minutes, and the mixture was stirred at 70 ºC under a reflux condenser for 16 hours. The mixture was evaporated to dryness, and the residue was washed with methanol (10 mL) and acetonitrile (10 mL). The combined extracts were concentrated to ca. 3 mL before 10 mL of diethyl ether was added to precipitate the product. The solution was then removed, and the solid was dried *in vacuo*. Yield: 15.7 mg (72%).

^{1}H NMR (600 MHz, [D$_4$]methanol, 300 K) δ (ppm): 9.02 (b, 4H, C), 9.00 (d, ^3J = 8.3 Hz, 4H, B), 8.39 (s, 4H, J), 8.25 (d, ^3J = 8.3 Hz, 4H, A), 8.05 (d, ^3J = 5.5 Hz, 4H, I), 7.50 (d, ^3J = 5.5 Hz, 4H, H), 7.11 (d, ^3J = 8.5 Hz, 8H, E), 6.60 (d,^3J = 8.5 Hz, 8H, F), 5.00 (s, 8H, G), 4.89 (s, 8H, D).
^{13}C NMR (151 MHz, [D$_4$]methanol, 300 K) δ (ppm): 160.7, 158.1, 156.8, 156.2, 152.9, 152.3, 151.2, 143.1, 133.7, 131.5, 129.0, 127.4, 123.8, 122.7, 115.8, 67.3, 63.4. *Not all the signals in the ^{13}C NMR spectrum were assigned due to high broadening.*
HR-MS (ESI+, TOF): *m/z* 905.0244 calcd. for $[C_{72}H_{56}N_{12}O_4Ag_4(CF_3CO_2)_2]^{2+}$ 905.0224.

Trefoil Knot 7-3Ag₂

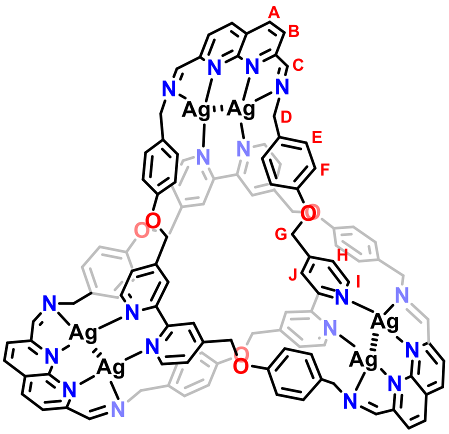

Deprotection of Boc-protected amine **S1** was performed directly before carrying self-assembly. After removing the excess of TFA with methanol, the oily residue **1** (21 mg, 0.024 mmol) was dissolved in 10 mL of *n*-PrOH and placed in a 25 mL round bottom flask. AgOTf (98 mg, 0.38 mmol) was then added, and the solution was stirred for 10 minutes before adding 2,7-diformyl-1,8-naphthyridine **5** (5 mg, 0.026 mmol). After the complete dissolution of reagents, the mixture was stirred at 95ºC under a reflux condenser for 16 hours. The yellow precipitate was separated using a centrifuge and dried before 5 mL of methanol was added and separated from the insoluble residue in a centrifuge. The solution was concentrated on a rotary evaporator to 2 mL before diethyl ether was added to precipitate the product. **7-3Ag₂** was filtered and dried *in vacuo*. Yield 2.0 mg (8%).

^{1}H NMR (600 MHz, [D$_4$]methanol, 300 K) δ (ppm): 9.11 – 9.06 (m, 12H, B, C), 8.95 (s, 6H, J), 8.31 (d, ^3J = 8.4 Hz, 6H, A), 7.92 (d, ^3J = 5.4 Hz, 6H, I), 7.79 (d, ^3J = 5.4 Hz, 6H, H), 7.22 (d, ^3J = 8.7 Hz, 12H, E), 7.00 (d, ^3J = 8.7 Hz, 12H, F), 5.32 (d, ^2J = 16.0 Hz, 6H, G), 5.10 (d, ^2J = 16.0 Hz, 6H, G), 4.89 – 4.80 (2 × m, D). The integration for D (CH$_2$) signals could not be precisely determined due to the overlap with solvent signals.
^{13}C NMR (151 MHz, [D$_4$]methanol, 300 K) δ (ppm): 160.8, 159.1, 156.3, 155.6, 154.1, 152.7, 151.6, 143.7, 132.9, 131.9, 129.5, 127.8, 124.0, 123.0, 122.0 (CF_3SO_3, $^1J_{C,F}$ = 319.0 Hz), 116.1, 68.5, 63.3.
^{19}F NMR (565 MHz, [D$_4$]methanol, 300 K) δ (ppm): –79.9.
HR-MS (ESI+, TOF): *m/z* 1486.9656 calcd. for $[C_{108}H_{84}N_{18}O_6Ag_6(CF_3COO)_4]^{2+}$ 1486.9608.

[2]Catenane 9-2Ag₂

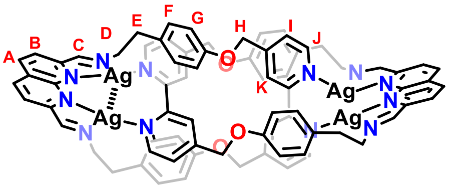

Deprotection of Boc-protected amine **S4** was performed directly before carrying self-assembly. After removing the excess of TFA with methanol, the oily residue **8** (14.3 mg, 0.016 mmol) was dissolved in 10 mL of *iso*-PrOH and placed in a 25 mL round bottom flask. Then AgOTf (17 mg, 0.063 mmol) and 2,7-diformyl-1,8-naphthyridine **5** (3.1 mg, 0.017 mmol) were added. The mixture was then stirred for 20 hours at 70 ºC under a reflux condenser. The yellow precipitate was separated on the centrifuge, washed with 5 mL of methanol and acetonitrile, filtered from the insoluble residue, evaporated on a rotary evaporator, and dried *in vacuo*. Yield 6.2 mg (35 %).

^{1}H NMR (500 MHz, [D$_4$]methanol, 300 K) δ (ppm): 9.06 (d, ^3J = 8.2 Hz, 4H, B), 9.04 (s, 4H, C), 8.55 (s, 4H, K), 8.33 (d, ^3J = 5.4 Hz, 4H, J), 8.29 (d, ^3J = 8.2 Hz, 4H, A), 7.60 (d, ^3J = 5.4 Hz, ^4J = 1.2 Hz, 4H, I), 7.09 (d, ^3J = 8.4 Hz, 8H, F), 6.72 (d, ^3J = 8.4 Hz, 8H, G), 5.20 (s, 8H, H), 4.35 (m, 8H, D), 3.00 (m, 8H, E).
^{13}C NMR (151 MHz, [D$_4$]methanol, 300 K) δ (ppm): 164.7, 157.1, 156.8, 156.0, 153.5, 152.2, 143.7, 133.7, 130.2, 129.5, 128.2, 124.2, 124.1, 121.8 (CF_3SO_3, $^1J_{C,F}$ = 319.0 Hz), 116.6, 68.4, 62.0, 36.1.
^{19}F NMR (565 MHz, [D$_4$]methanol, 300 K) δ (ppm): –80.0.
HR-MS (ESI+, TOF): *m/z* 596.3638 calcd. for $[C_{76}H_{64}N_{12}O_4Ag_4(CF_3SO_3)]^{3+}$ 596.3629; 951.0400 calcd. for $[C_{76}H_{64}N_{12}O_4Ag_4(CF_3CO_2)_2]^{2+}$ 951.0372; 969.0235 calcd. for $[C_{76}H_{64}N_{12}O_4Ag_4(CF_3SO_3)_2]^{2+}$ 969.0206.

Solomon Link 10-4Ag$_2$

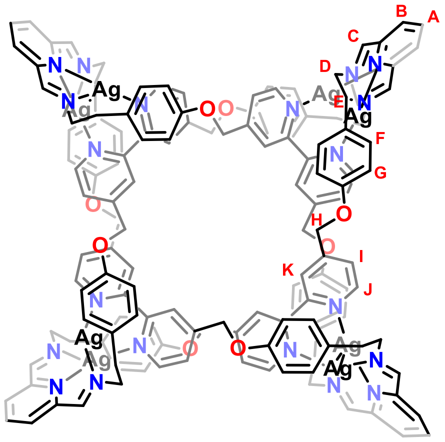

Deprotection of Boc-protected amine **S4** was performed directly before carrying self-assembly. After removing the excess of TFA with methanol, the oily residue **8** (21 mg, 0.023 mmol) was dissolved in 10 mL of *iso*-PrOH and placed in 25 mL round bottom flask. After complete dissolution of the salt **8**, AgOAc (38 mg, 0.23 mmol) and 2,6-diformylpyridine **2** (3.2 mg, 0.024 mmol) were placed in the flask. The mixture was stirred for 20 hours at 70ºC under a reflux condenser. The yellow precipitate was separated on a centrifuge. The solution was then evaporated to dryness and dissolved in ca. 3 mL of methanol before diethyl ether was added to precipitate the product. The solid was then separated from the solution and dried *in vacuo*. Yield: 8 mg (18%).

^{1}H NMR (600 MHz, [D$_4$]methanol, 330 K) δ (ppm): 8.87 (d, *J* = 2.1 Hz, 8H, C), 8.67 (d, ^3J = 5.4 Hz, 8H, J), 8.35 (t, ^3J = 7.8 Hz, 4H, A), 8.34 (s, 8H, K), 8.06 (d, ^3J = 8.0 Hz, 8H, B), 7.98 (d, ^3J = 5.4 Hz, ^4J = 1.0 Hz, 8H, I), 6.96 – 6.81 (m, 32H, F, G), 5.49 (d, ^2J = 15.0 Hz, 8H, H), 4.64 (d, ^2J = 15.0 Hz, 8H, H), 3.98 – 3.92 (m, 8H, D), 3.12 – 3.04 (m, 8H, D), 3.03 – 2.95 (m, 8H, E), 2.57 – 2.48 (m, 8H, E).
^{13}C NMR (151 MHz, [D$_4$]methanol, 330 K) δ (ppm): 165.0, 163.0 (m, TFA), 157.5, 156.1, 154.1, 152.6, 152.2, 141.5, 132.7, 131.5, 129.8, 124.2, 123.7, 116.2, 67.6, 64.4, 36.7. *Not all the signals in the ^{13}C NMR spectrum were assigned due to their broadening.*
^{19}F NMR (565 MHz, [D$_4$]methanol, 300 K) δ (ppm): −76.0.
HR-MS (ESI+, TOF): *m/z* 1877.5779 calcd. for $[C_{140}H_{124}N_{20}O_8Ag_8(CF_3CO_2)_6]^{2+}$ 1877.5725.

Solomon Link (PF$_6$)$_2$ ⊂ 10-4Ag$_2$

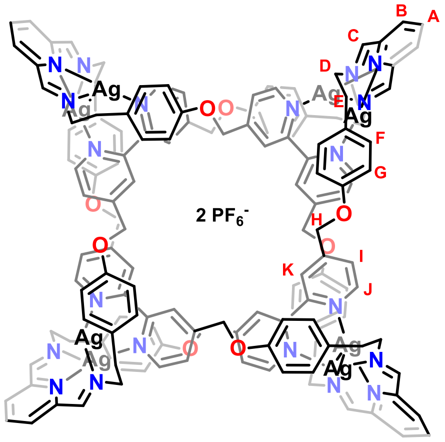

Deprotection of Boc-protected amine **S4** was performed directly before carrying self-assembly. After removing the excess of TFA with methanol, the oily residue **8** (19.7 mg, 0.022 mmol) was dissolved in 10 mL of *iso*-PrOH and placed in 25 mL round bottom flask. After complete dissolution of **8** salt, AgOAc (36 mg, 0.22 mmol) and 2,6-diformylpyridine **2** (3.0 mg, 0.023 mmol), KPF$_6$ (2 mg, 0.011 mmol) were placed in the flask. The mixture was stirred for 20 hours at 70ºC under a reflux condenser. The yellow precipitate was separated, dissolved in 5 mL of methanol, and concentrated at 2 mL before 10 mL of diethyl ether was added to precipitate the product. The solution was then removed, and the solid was dried *in vacuo*. Yield 3.7 mg (8 %).
^{1}H NMR (500 MHz, [D$_4$]methanol, 300 K) δ (ppm): 8.90 (s, 8H, C), 8.57 (d, ^3J = 5.5 Hz, 8H, J), 8.40 – 8.35 (m, 12H, A, K), 8.10 (d, ^3J = 8.0 Hz, 8H, B), 7.94 (dd, ^3J = 5.5 Hz, ^4J = 0.7 Hz, 8H, I), 6.94 (d, ^3J = 7.8 Hz, 16H, F), 6.83 (s, 16H, G), 5.45 (d, ^2J = 15.8 Hz, 8H, H), 4.62 (d, ^2J = 15.8 Hz, 8H), 3.98 – 3.90 (m, 8H, D), 3.09 – 2.97 (m, 16H, D, E), 2.51 (td, ^2J = 14.5, ^3J = 3.3 Hz, 8H, E).

^{13}C NMR (151 MHz, [D$_4$]methanol, 330 K) δ (ppm): 165.1, 163.0 (q, $^3J_{C,F}$ = 34.5 Hz, TFA), 157.7, 156.3, 153.8, 152.6, 152.5, 141.4, 132.6, 131.5, 129.7, 124.0, 118.5 (q, $^2J_{C,F}$ = 295.0 Hz, TFA), 116.3, 67.7, 64.6, 36.8. *Not all the signals in the ^{13}C NMR spectrum were assigned due to broadening.*
^{19}F NMR (471 MHz, [D$_4$]methanol, 230 K) δ (ppm): –69.6 (d, $^1J_{P,F}$ = 711 Hz, PF_6^-), –76.4 (s, CF_3COO^-).
HR-MS (ESI+, TOF): *m/z* 1910.0514 calcd. for $[C_{140}H_{124}N_{20}O_8Ag_8(PF_6)_2(CF_3COO)_4]^{2+}$ 1910.0515.

Solomon Link $(BF_4)_n$ ⊂ 10-4Ag$_2$

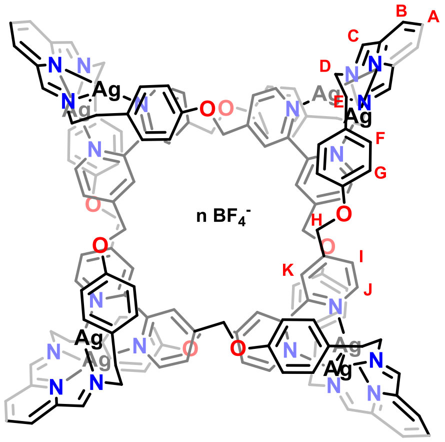

Deprotection of Boc-protected amine **S4** was performed directly before carrying self-assembly. After removing the excess of TFA with methanol, the oily residue **8** (20.3 mg, 0.022 mmol) was dissolved in 10 mL of *iso*-PrOH and placed in 25 mL round bottom flask. After complete dissolution of **8** salt 2,6-diformylpyridine **2** (3.1 mg, 0.023 mmol) and AgOAc (38 mg, 0.22 mmol), KBF_4 (1.5 mg, 0.011 mmol) were placed in the flask. The mixture was stirred for 20 hours at 70ºC under a reflux condenser. The yellow precipitate was separated on a centrifuge. The solution was then evaporated to dryness and dissolved in ca. 3 mL of methanol before diethyl ether was added to precipitate the product. The solid was then separated from the solution and dried *in vacuo*. Yield 6.5 mg (30 %).

^{1}H NMR (600 MHz, [D$_4$]methanol, 330 K) δ (ppm): 8.86 (d, $^3J_{H,Ag}$ = 2.0 Hz, 8H, C), 8.66 (d, ^3J = 5.4 Hz, J), 8.33 (t, ^3J = 7.8, 4H, A), 8.32 (s, 8H, K), 8.06 (d, ^3J = 8.0 Hz, 8H, B), 7.97 (d, ^3J = 5.4 Hz, 8H, I), 6.87 (m, 32H, F, G), 5.48 (d, ^2J = 14.9 Hz, 8H, H), 4.67 (d, ^2J = 14.9 Hz, 8H, H), 3.96 (m, 8H, D), 3.09 (m, 8H, D), 3.00 (m, 8H, E), 2.54 (m, 8H, E).
^{13}C NMR (151 MHz, [D$_4$]methanol, 330 K) δ (ppm): 165.0, 163.0 (q, $^3J_{C,F}$ = 34.5 Hz, TFA), 157.5, 156.0, 154.0, 152.6, 152.2, 141.4, 132.7, 131.5, 129.7, 124.1, 123.7, 118.5 (q, $^2J_{C,F}$ = 294.0 Hz), 116.2, 67.6, 64.4, 36.7.
^{19}F NMR (471 MHz, [D$_4$]methanol, 230 K) δ (ppm): –76.3, –148.4.
HR-MS (ESI+, TOF): *m/z* 1864.5751 calcd. for $[C_{140}H_{124}N_{20}Ag_8(BF_4)(CF_3COO)_5]^{2+}$ 1864.5818.

High-resolution Mass Spectra

Mass spectra of molecular tweezer 3-2Ag$_2$

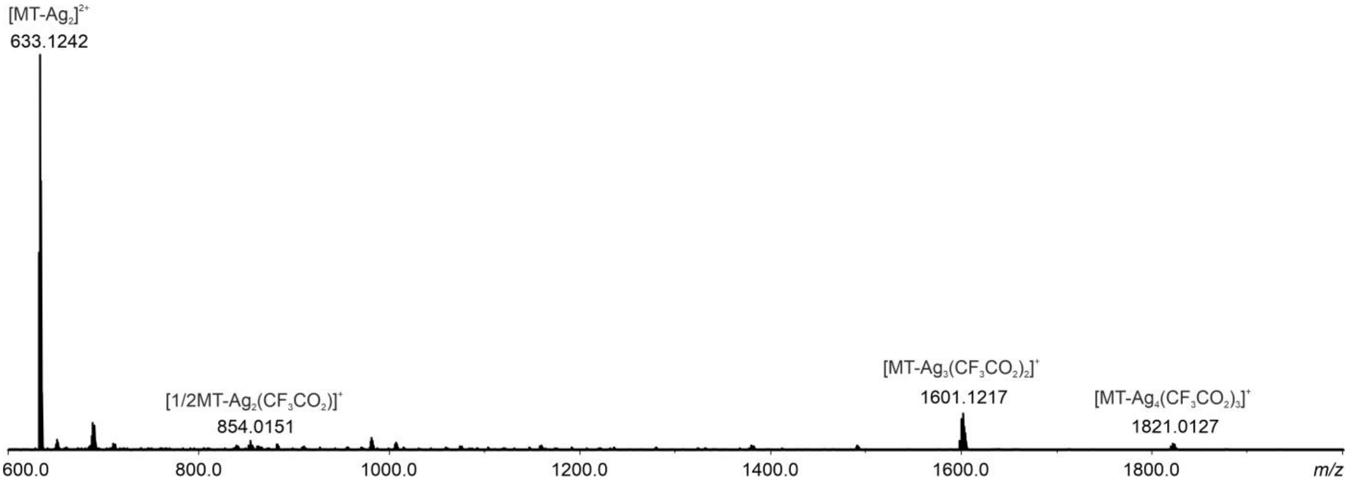

Figure S2. The 600-2000 *m/z* range of the high-resolution mass spectrum of **3-2Ag$_2$** (ESI+, TOF).

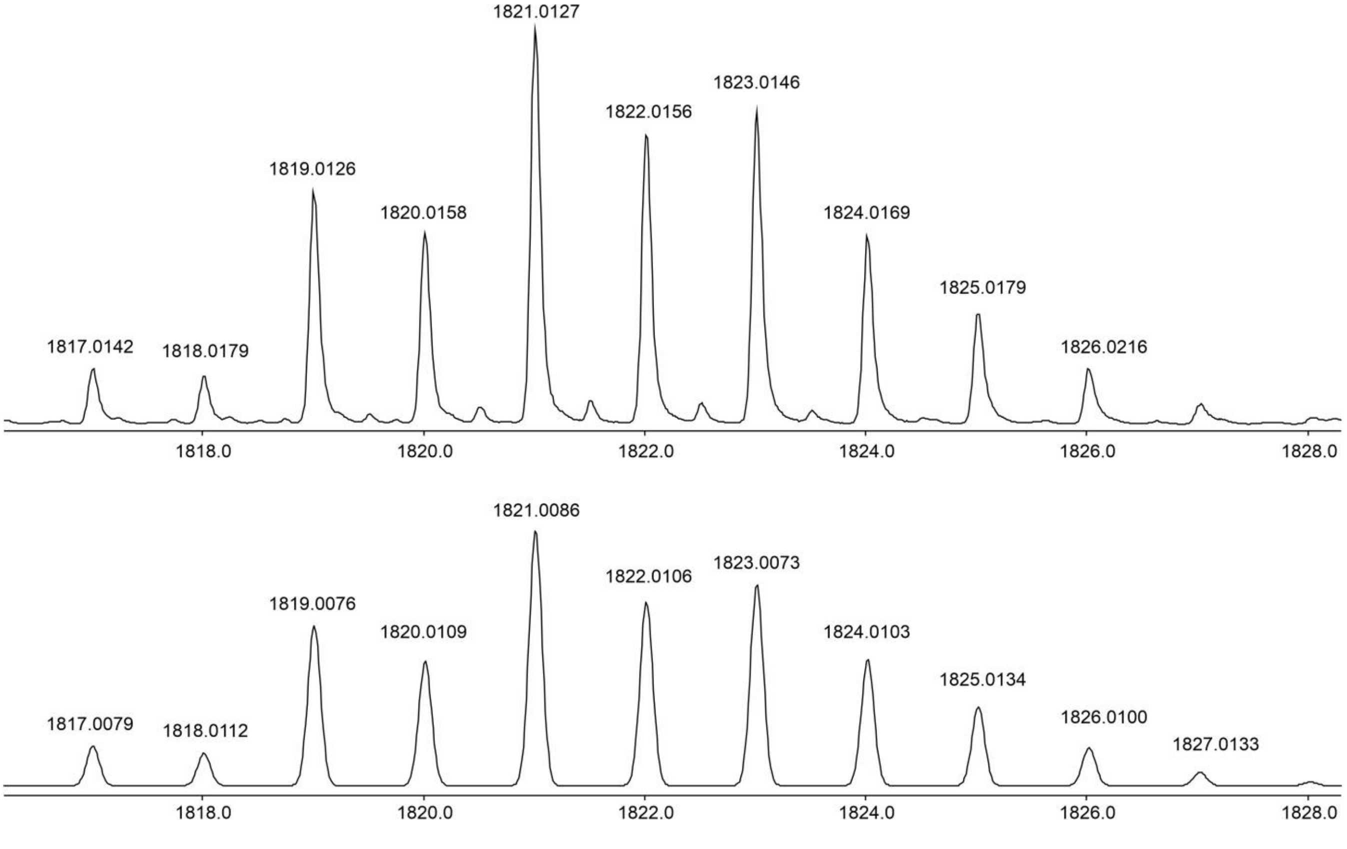

Figure S3. The high-resolution mass spectrum of **3-2Ag$_2$** (ESI+, TOF, [$C_{66}H_{54}N_{10}O_4Ag_4(CF_3COO)_3]^+$). Top: experimental spectrum, bottom: simulated pattern.

Mass spectra of the mixture of molecular tweezer, trefoil knot with traces of Solomon link

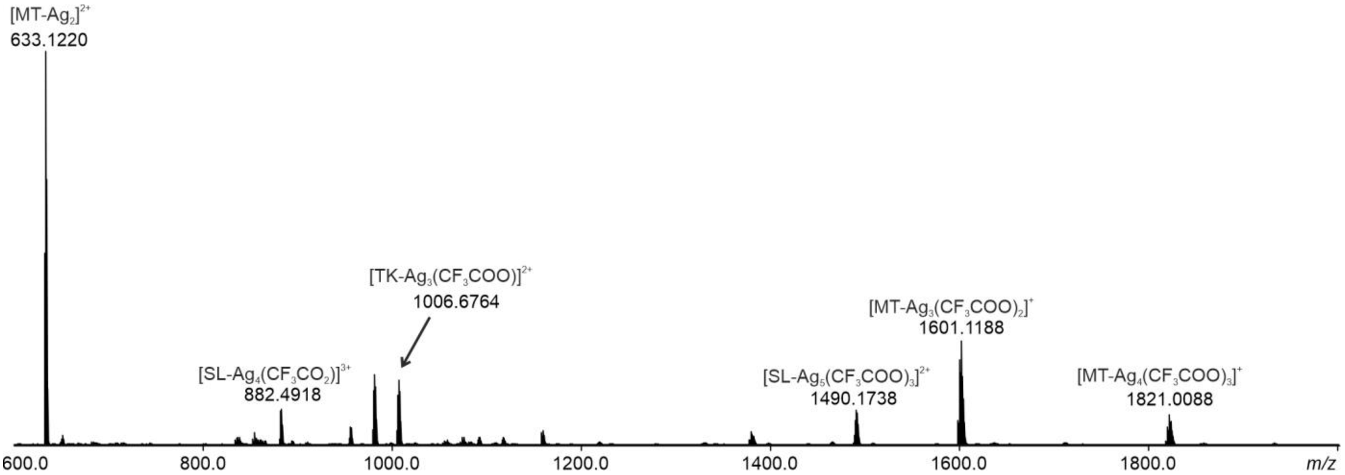

Figure S4. The 600-2000 *m/z* range of the high-resolution mass spectrum of a mixture of molecular tweezer **3-2Ag$_2$**, trefoil knot **4-3Ag**, and tetranuclear Solomon link.

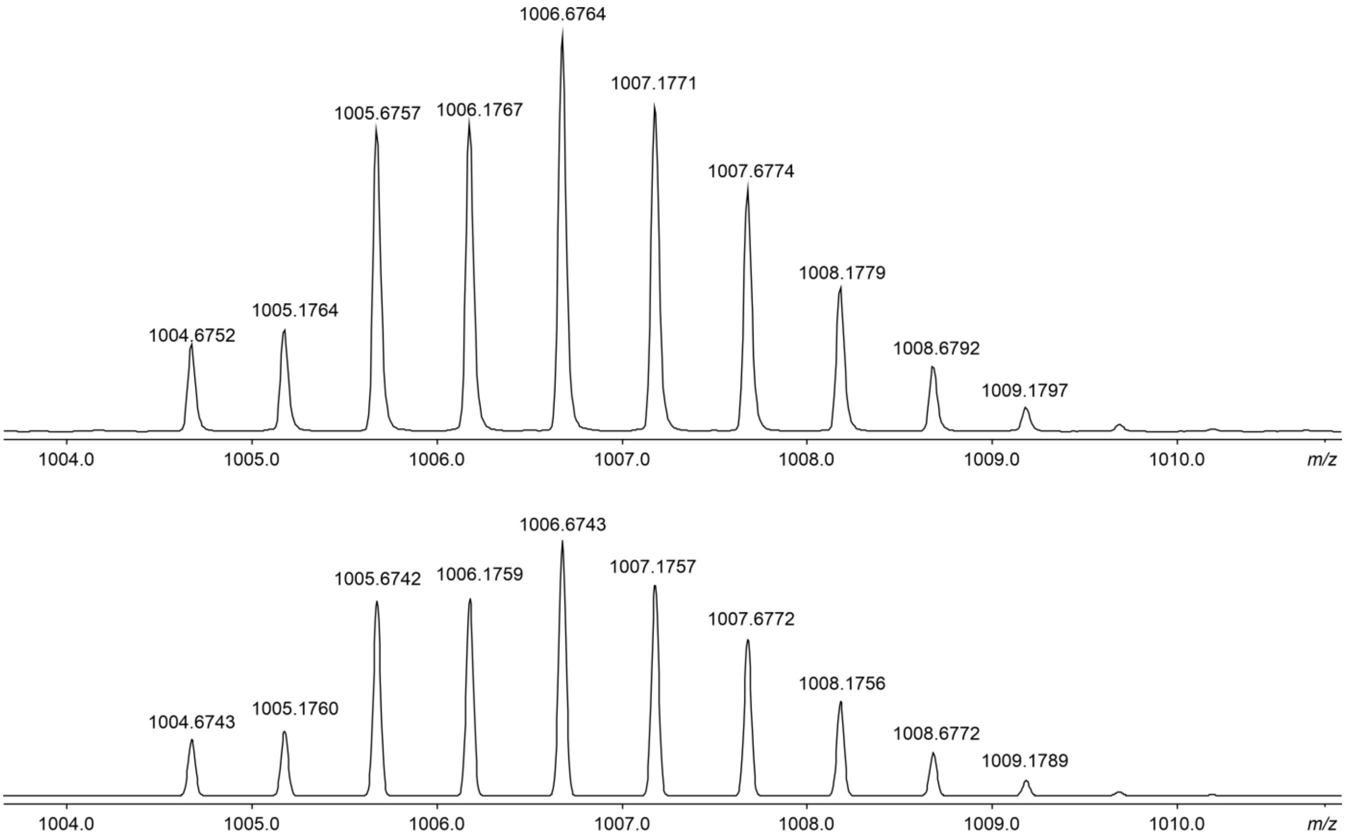

Figure S5. The high-resolution mass spectrum of **4-3Ag** (ESI+, TOF, $[C_{99}H_{81}N_{15}O_6Ag_4(CF_3COO)]^{2+}$). Top: experimental spectrum, bottom: simulated pattern.

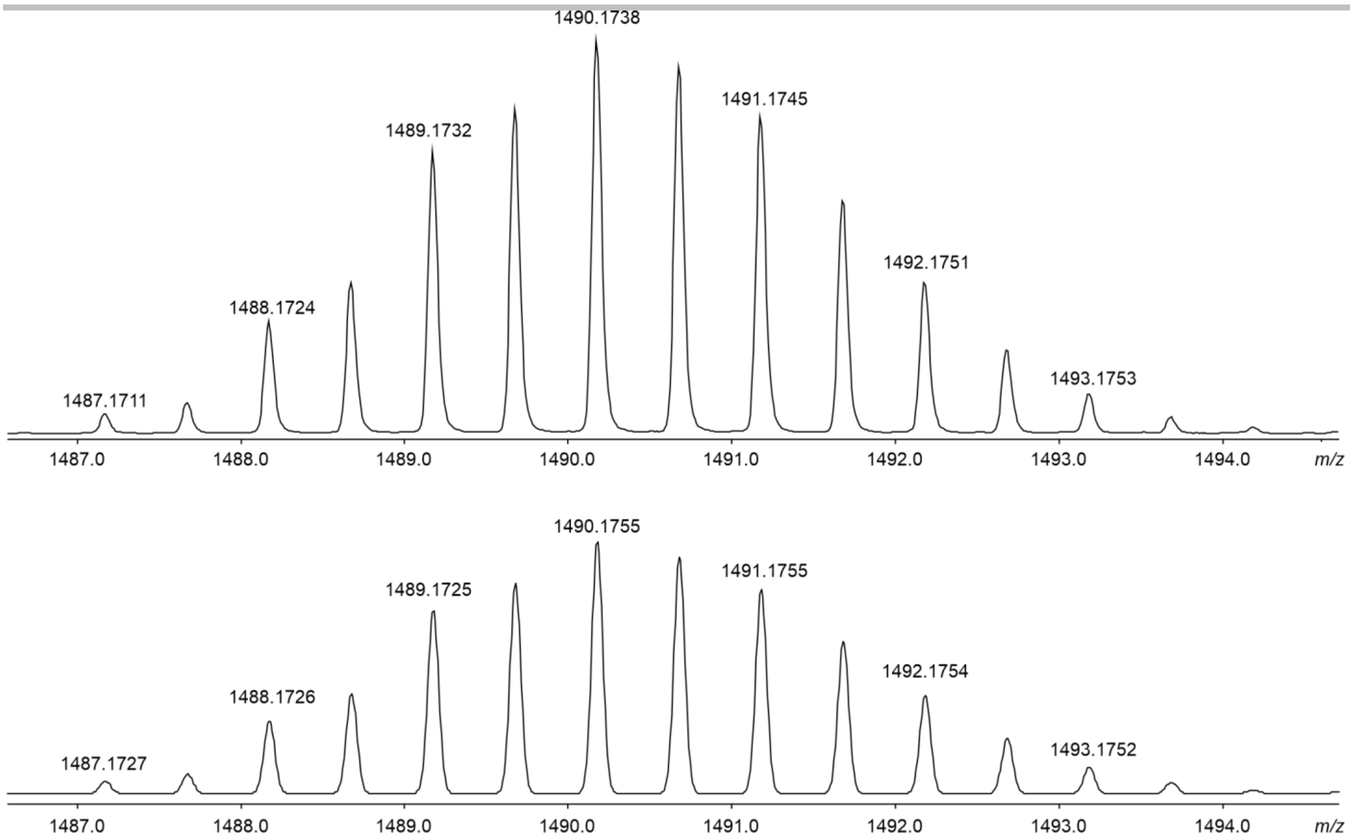

Figure S6. The high-resolution mass spectrum of an additional compound found in the mixture, with elemental composition corresponding to tetranuclear Solomon link (ESI+, TOF, $[C_{132}H_{108}N_{20}O_8Ag_5(CF_3COO)_3]^{2+}$). Top: experimental spectrum, bottom: simulated pattern.

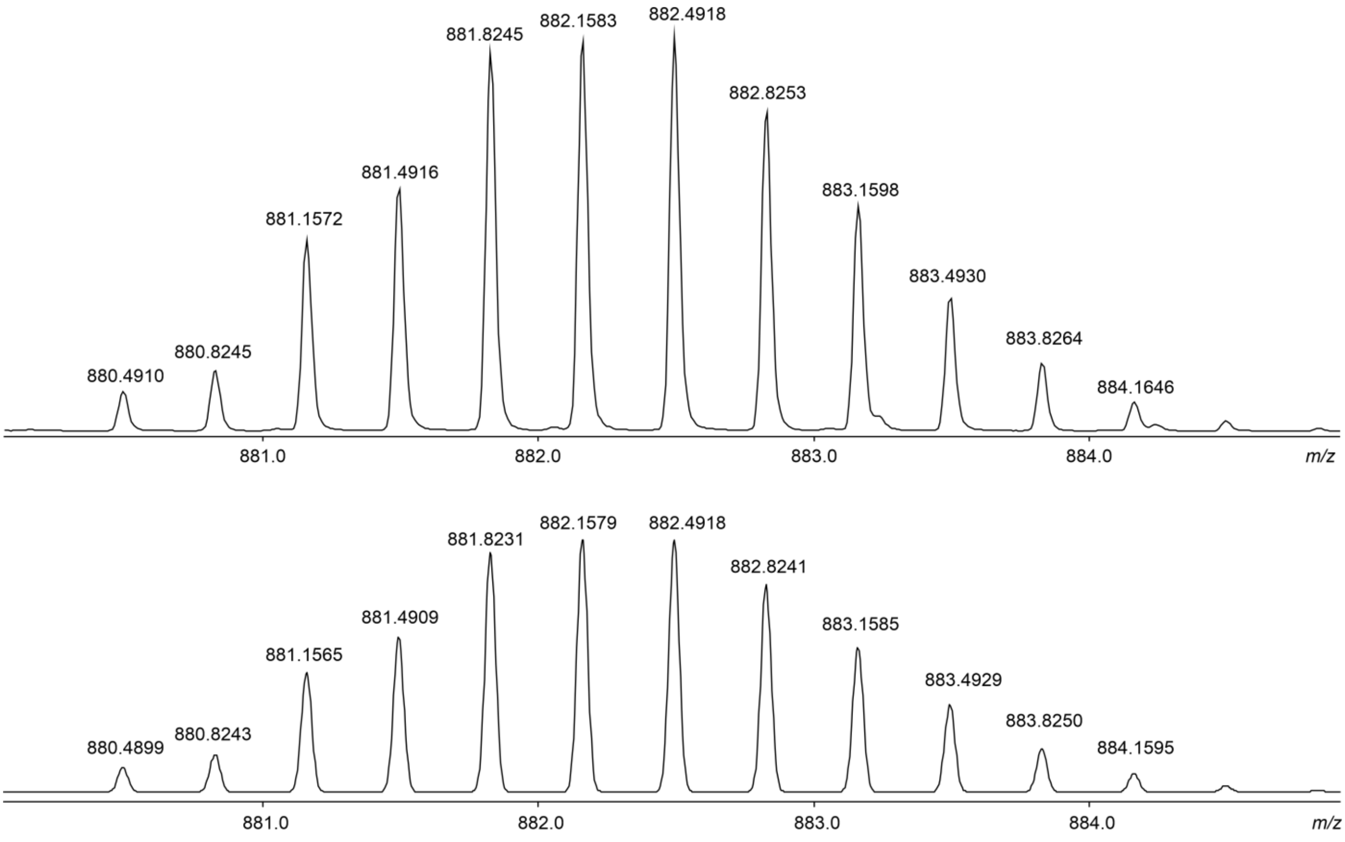

Figure S7. The high-resolution mass spectrum of additional compound found in the mixture with elemental composition corresponding to tetranuclear Solomon link (ESI+, TOF, $[C_{132}H_{108}N_{20}O_8Ag_4(CF_3COO)]^{3+}$). Top: experimental spectrum, bottom: simulated pattern.

Mass spectra of [2]catenane 6-2Ag$_2^{OTf}$

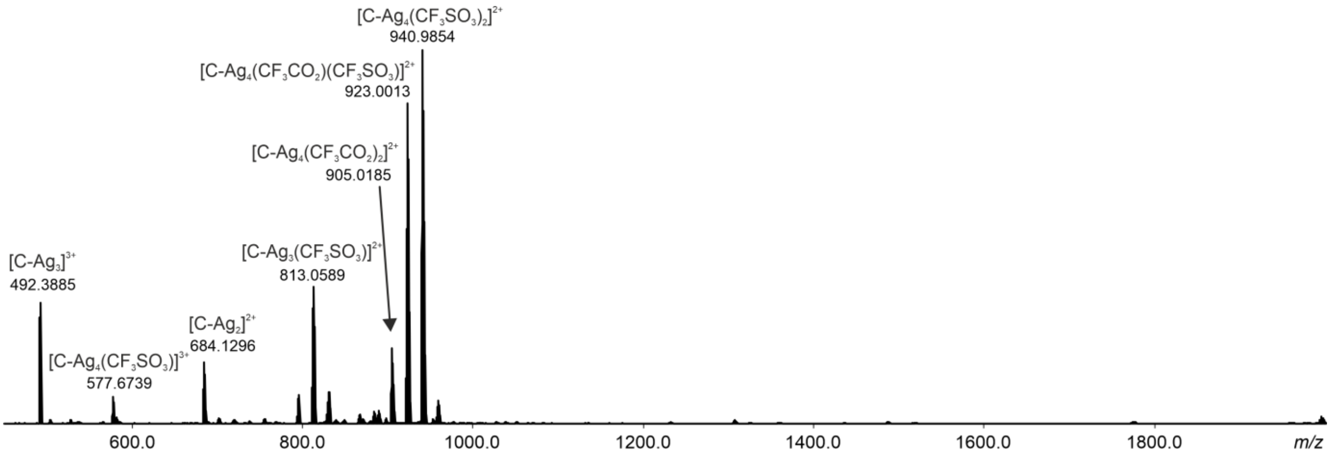

Figure S8. The 450-2000 *m/z* range of the high-resolution mass spectrum of **6-2Ag$_2^{OTf}$** (ESI+, TOF).

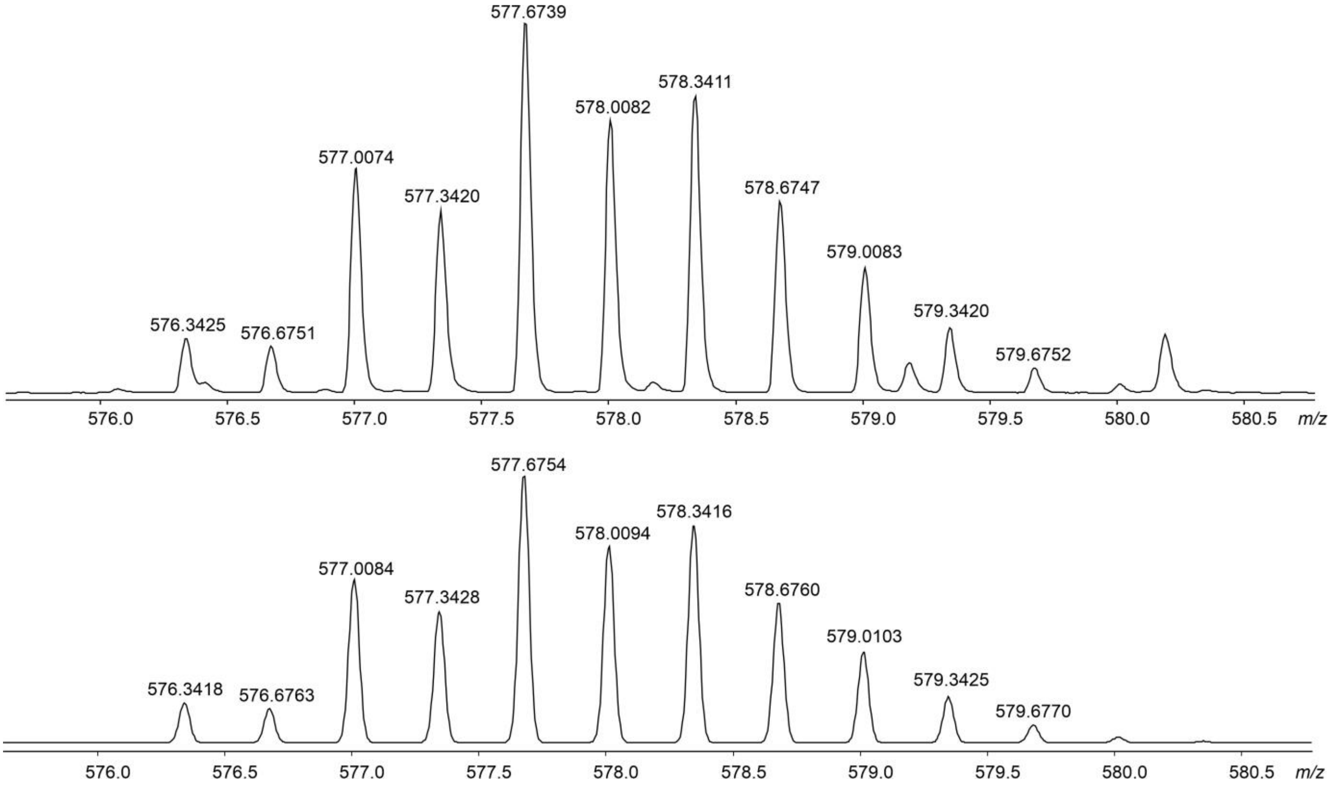

Figure S9. The high-resolution mass spectrum of **6-2Ag$_2^{OTf}$** (ESI+, TOF, [$C_{72}H_{56}N_{12}O_4Ag_4(CF_3SO_3)$]$^{3+}$). Top: experimental spectrum, bottom: simulated pattern.

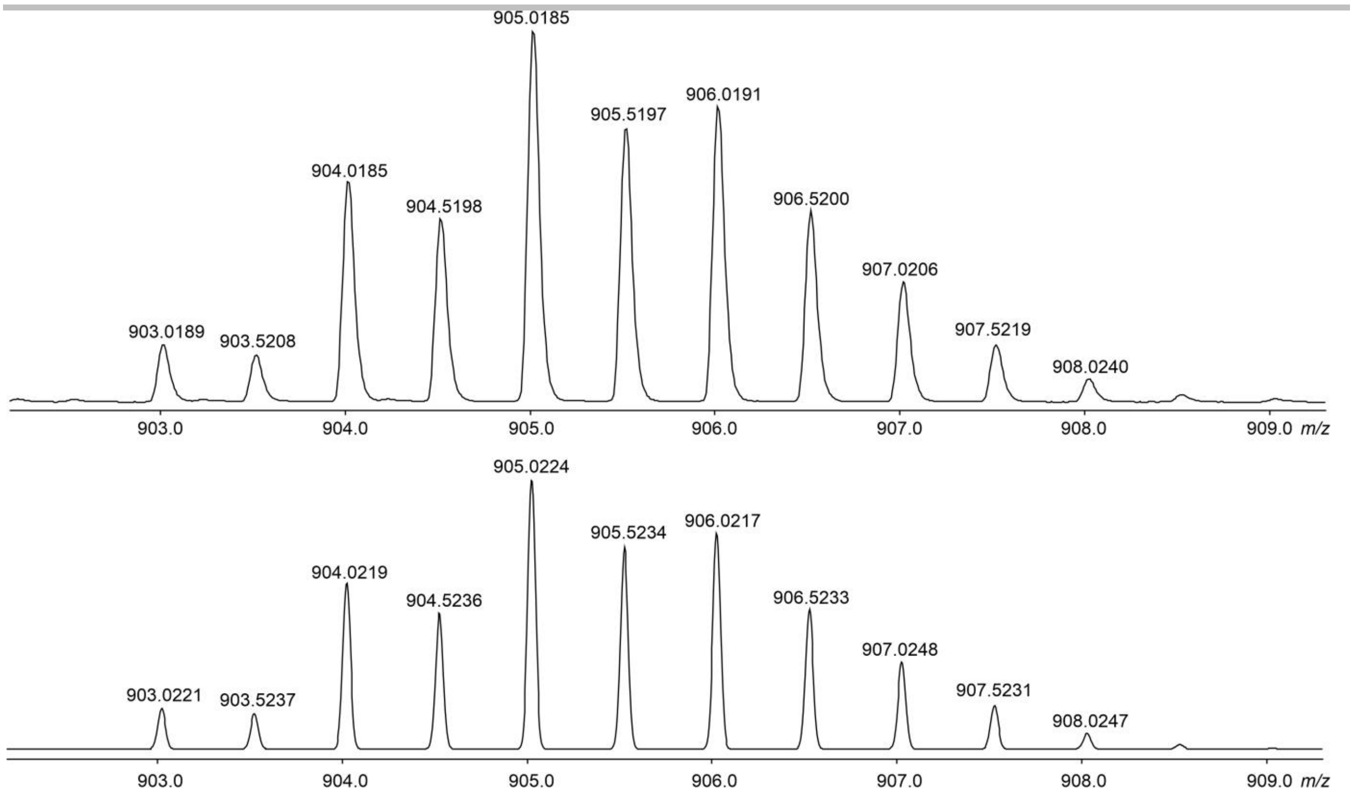

Figure S10. The high-resolution mass spectrum of **6-2Ag$_2^{OTf}$** (ESI+, TOF, $[C_{72}H_{56}N_{12}O_4Ag_4(CF_3CO_2)_2]^{2+}$). Top: experimental spectrum, bottom: simulated pattern.

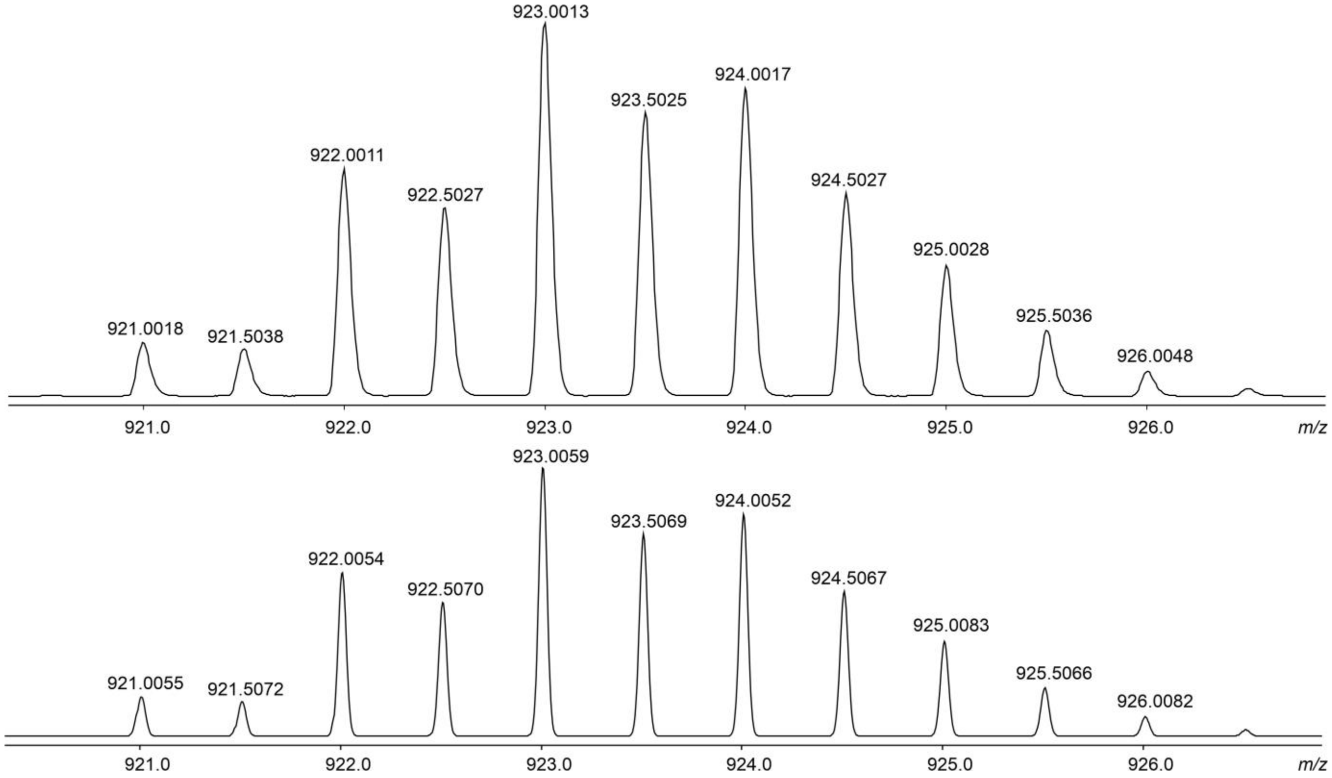

Figure S11. The high-resolution mass spectrum of **6-2Ag$_2^{OTf}$** (ESI+, TOF, $[C_{72}H_{56}N_{12}O_4Ag_4(CF_3CO_2)(CF_3SO_3)]^{2+}$). Top: experimental spectrum, bottom: simulated pattern.

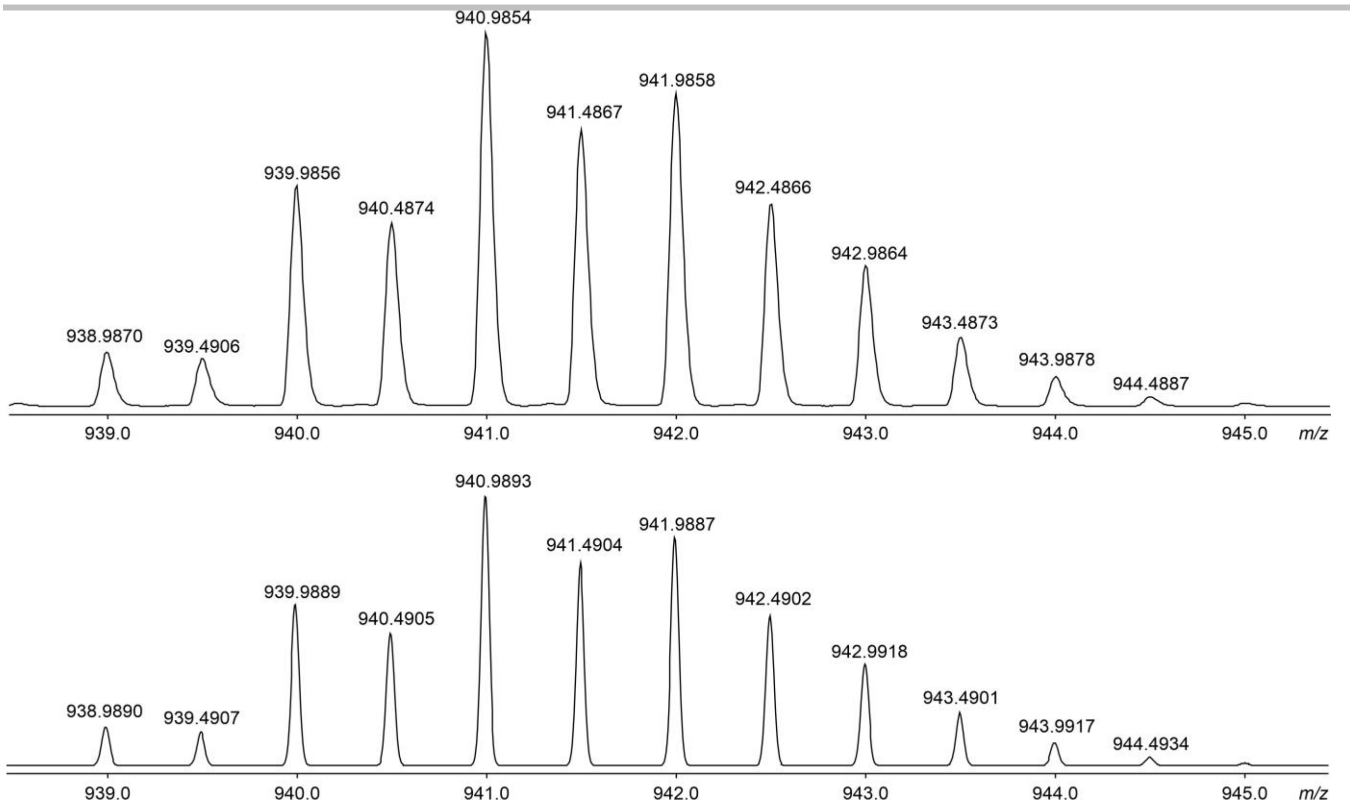

Figure S12. The high-resolution mass spectrum of **6-2Ag$_2$^{OTf}** (ESI+, TOF, $[C_{72}H_{56}N_{12}O_4Ag_4(CF_3SO_3)_2]^{2+}$). Top: experimental spectrum, bottom: simulated pattern.

Mass spectrum of [2]catenane 6-2Ag$_2^{OAc}$

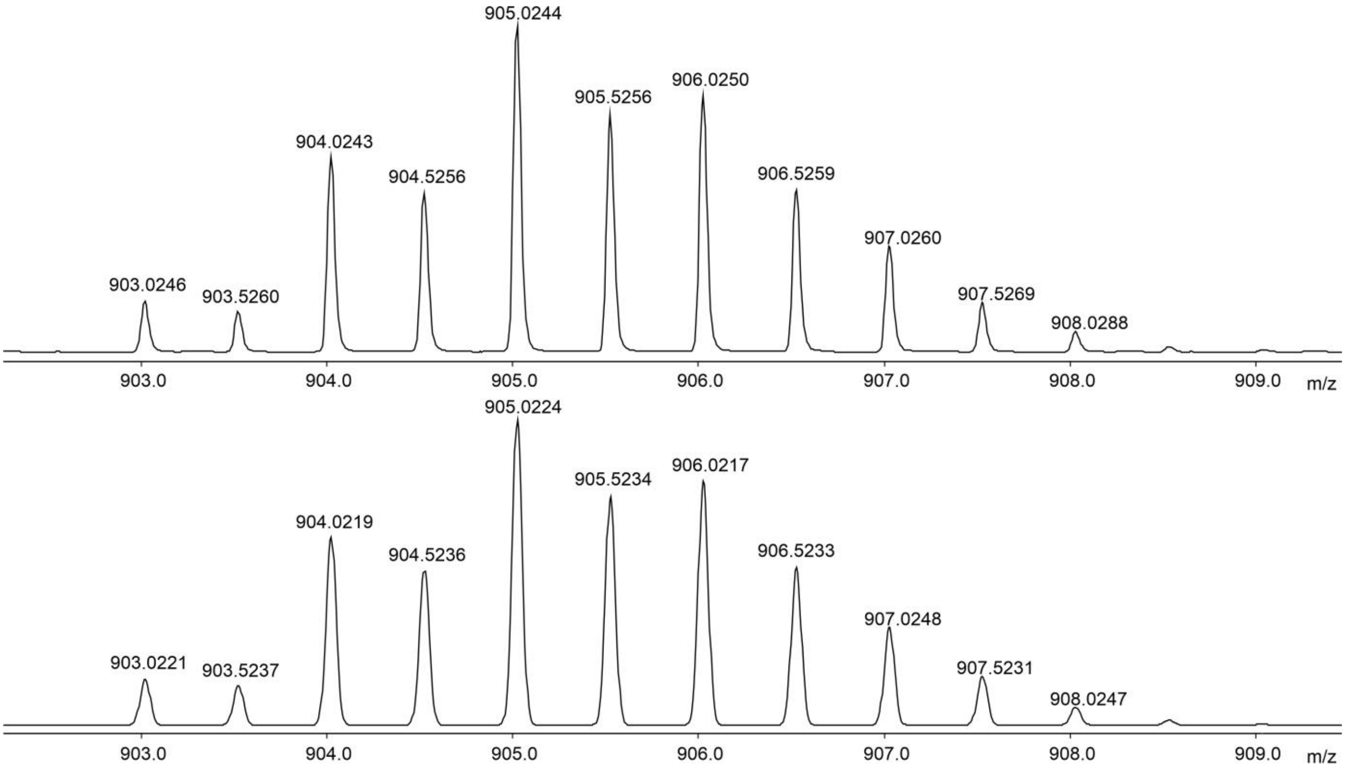

Figure S13. The high-resolution mass spectrum of **6-2Ag$_2^{OAc}$** (ESI+, TOF, $[C_{72}H_{56}N_{12}O_4Ag_4(CF_3CO_2)_2]^{2+}$). Top: experimental spectrum, bottom: simulated pattern.

Mass spectra of trefoil knot 7-3Ag₂

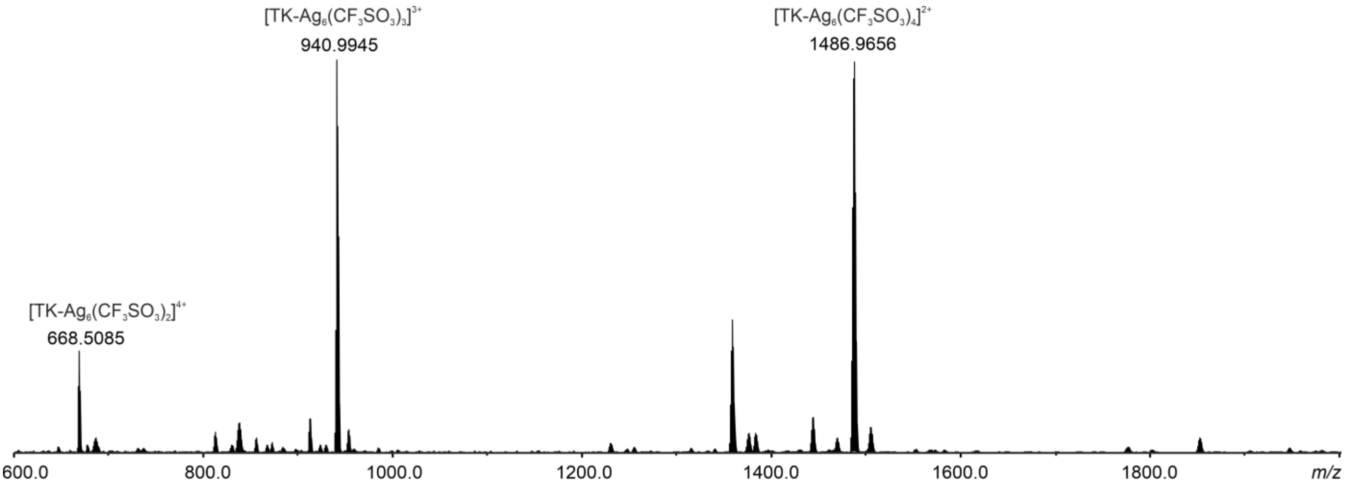

Figure S14. The 600-2000 *m/z* range of the high-resolution mass spectrum of **7-3Ag$_2$** (ESI+, TOF).

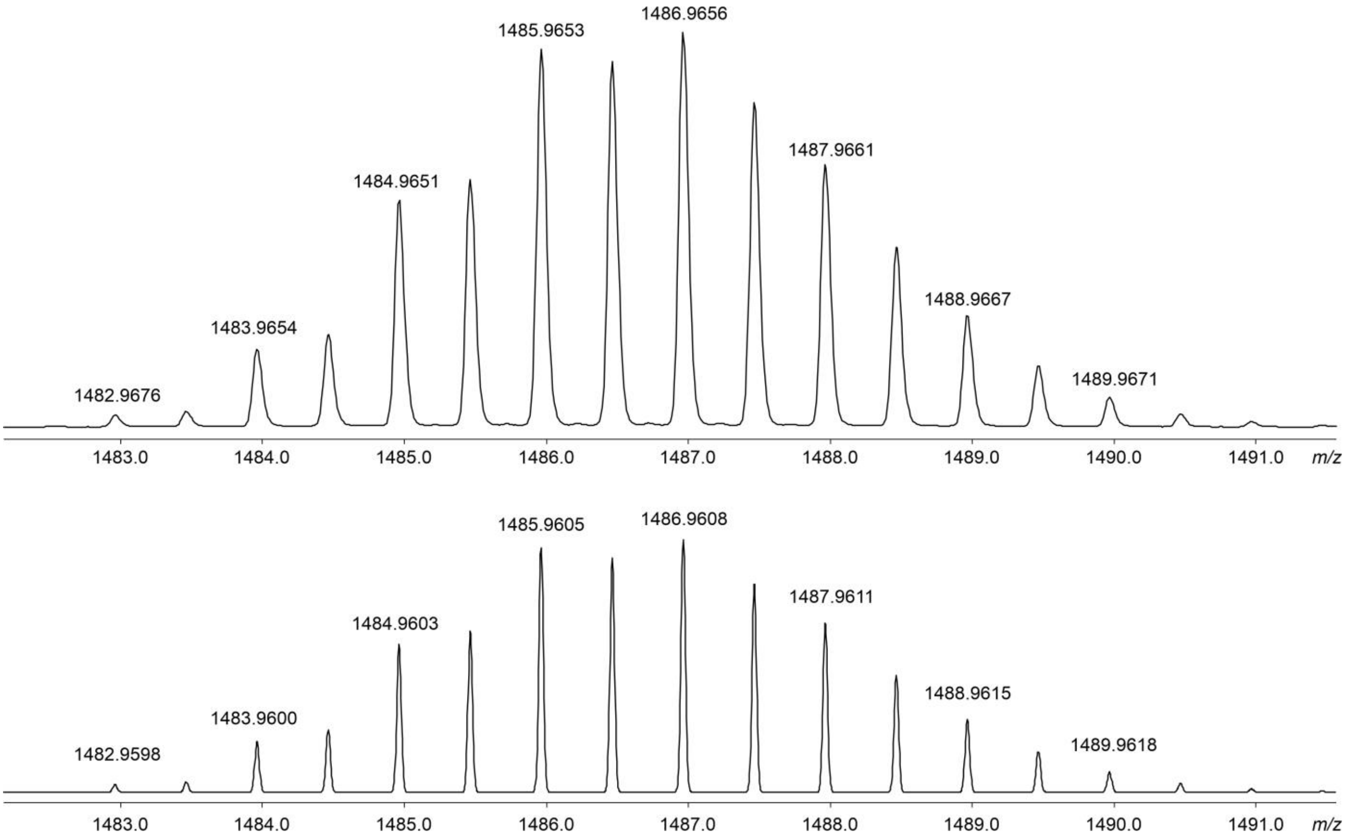

Figure S15. The high-resolution mass spectrum of **7-3Ag$_2$** (ESI+, TOF, $[C_{108}H_{84}N_{18}O_6Ag_6(CF_3COO)_4]^{2+}$). Top: experimental spectrum, bottom: simulated pattern.

Mass spectra of [2]catenane 9-2Ag$_2$

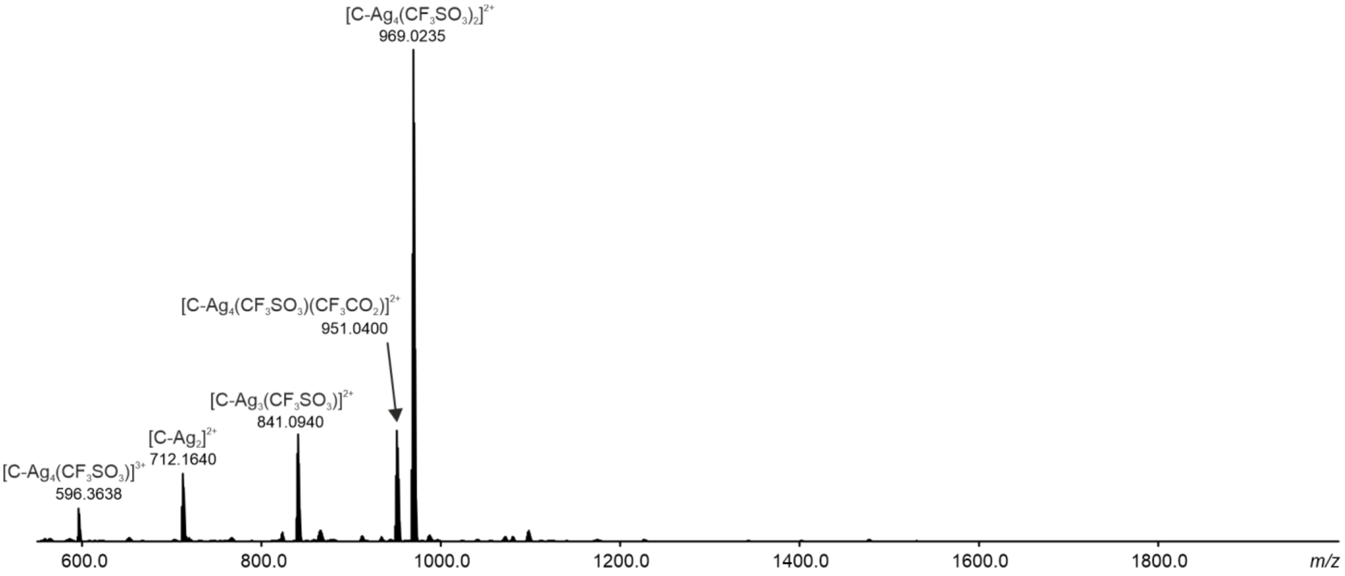

Figure S16. The 550-2000 *m/z* range of the high-resolution mass spectrum of **9-2Ag$_2$** (ESI+, TOF).

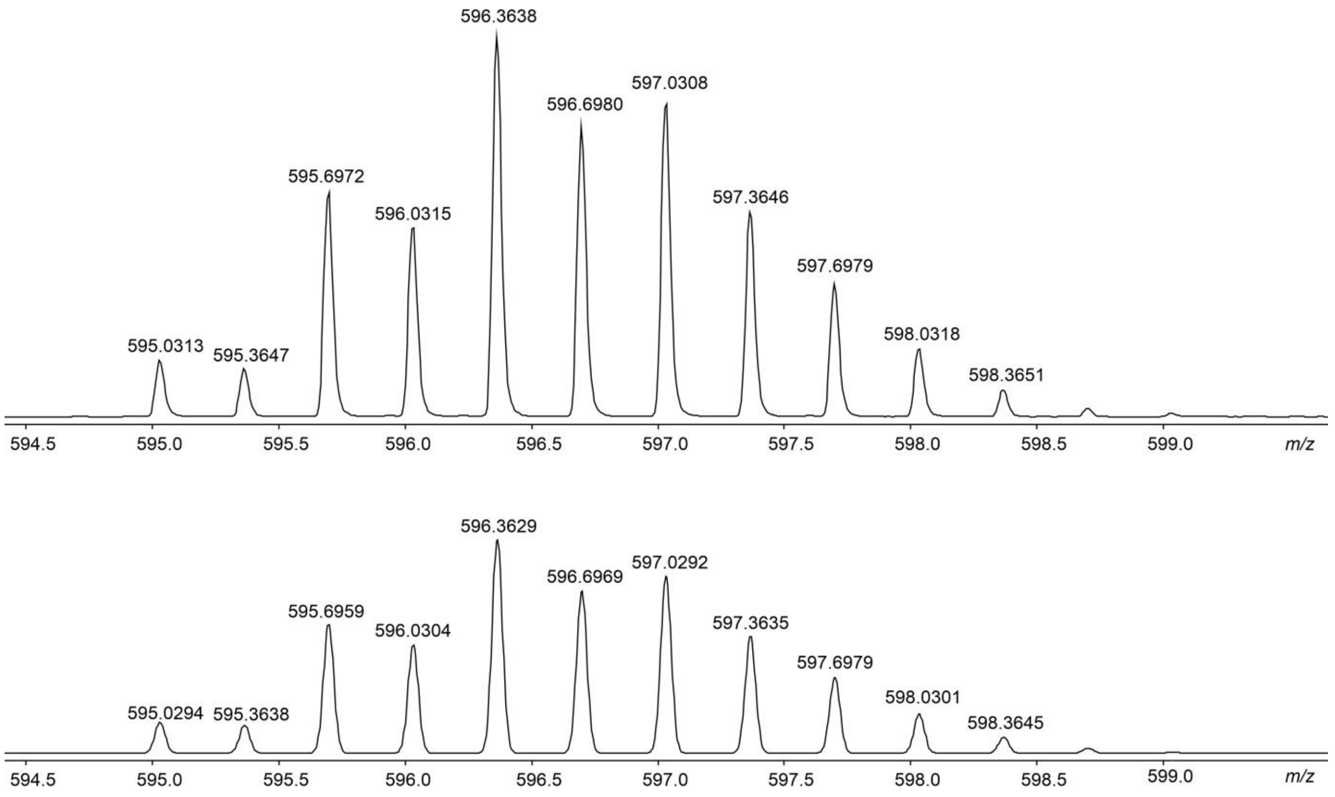

Figure S17. The high-resolution mass spectrum of **9-2Ag$_2$** (ESI+, TOF, $[C_{76}H_{64}N_{12}O_4Ag_4(CF_3SO_3)]^{3+}$). Top: experimental spectrum, bottom: simulated pattern.

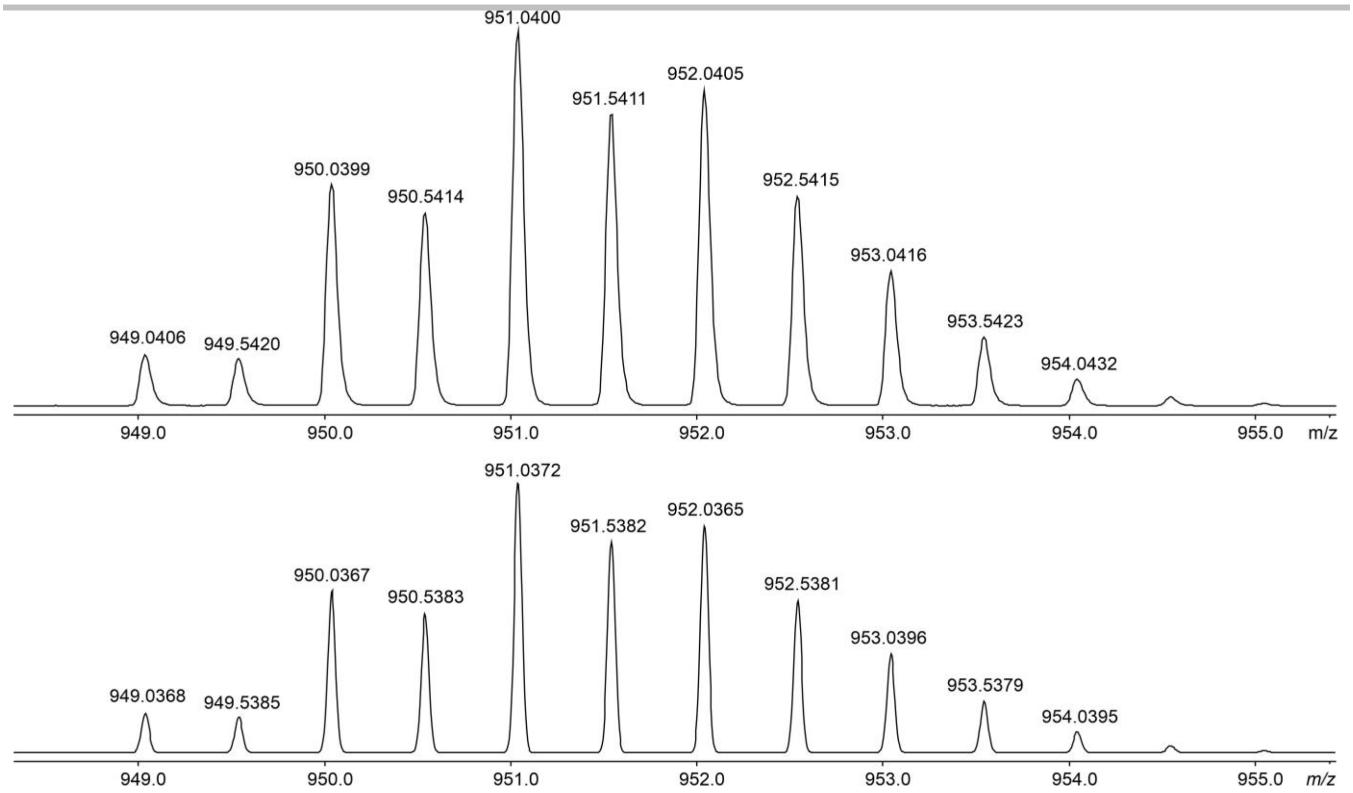

Figure S18. The high-resolution mass spectrum of **9-2Ag₂** (ESI+, TOF, $[C_{76}H_{64}N_{12}O_4Ag_4(CF_3SO_3)(CF_3CO_2)]^{2+}$). Top: experimental spectrum, bottom: simulated pattern.

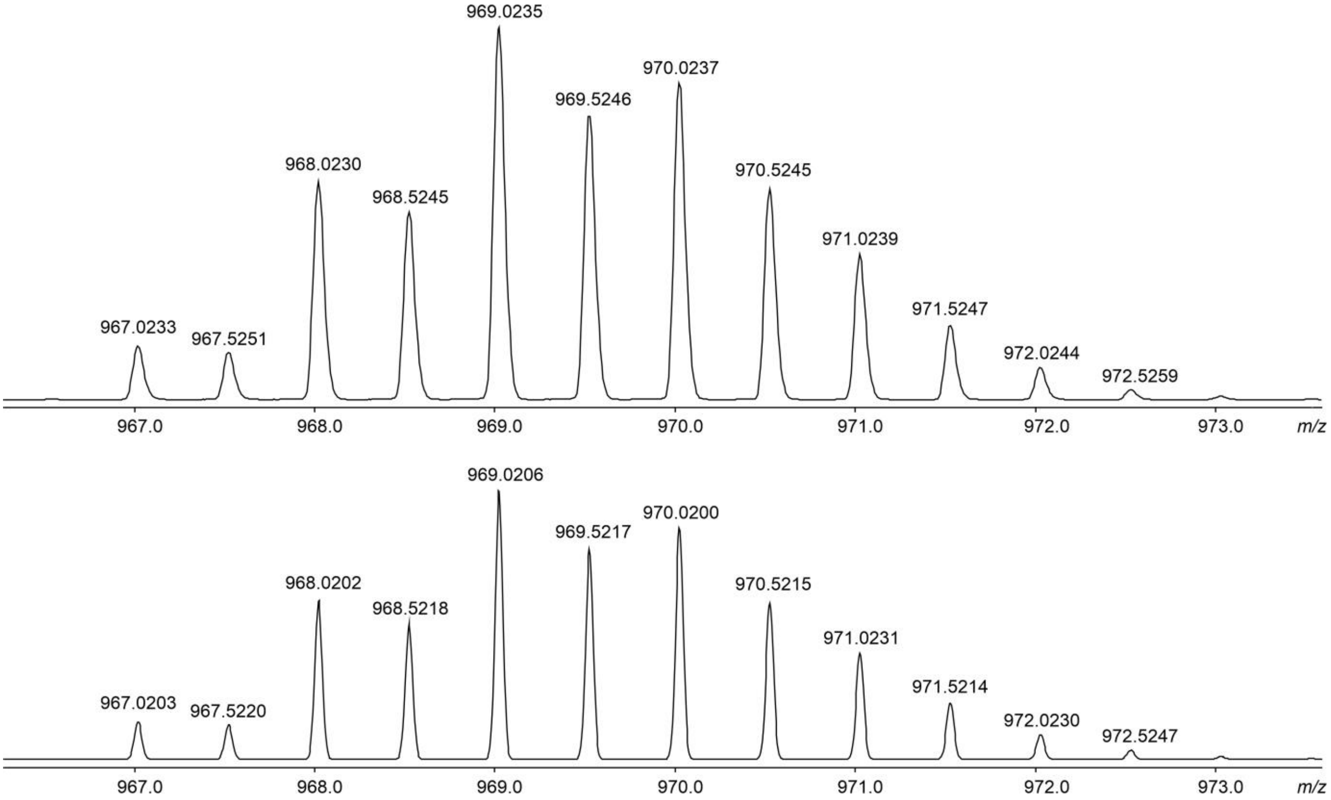

Figure S19. The high-resolution mass spectrum of **9-2Ag₂** (ESI+, TOF, $[C_{76}H_{64}N_{12}O_4Ag_4(CF_3SO_3)_2]^{2+}$). Top: experimental spectrum, bottom: simulated pattern.

Mass spectra of Solomon link 10-4Ag$_2$

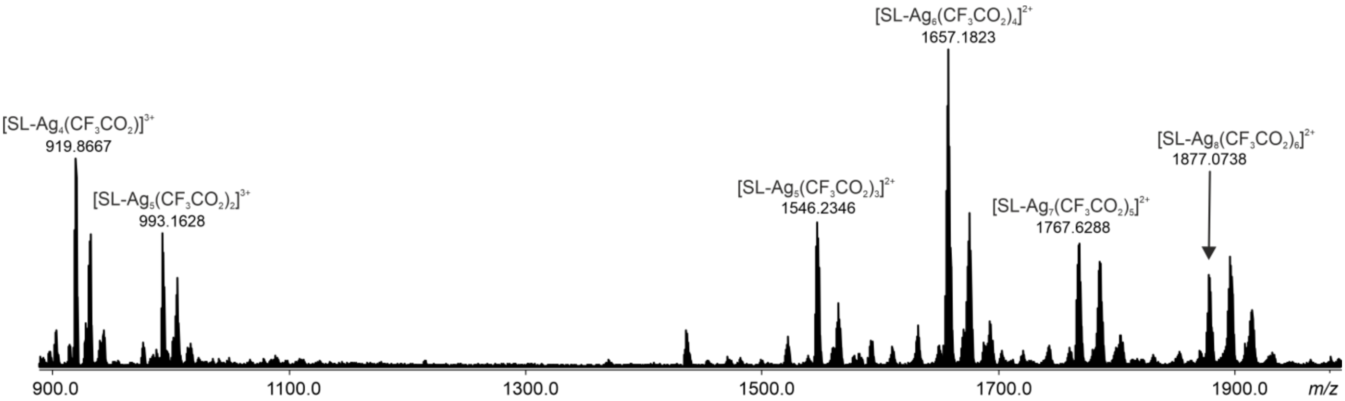

Figure S20. The 900-2000 *m/z* range of the high-resolution mass spectrum of **10-4Ag$_2$** (ESI+, TOF).

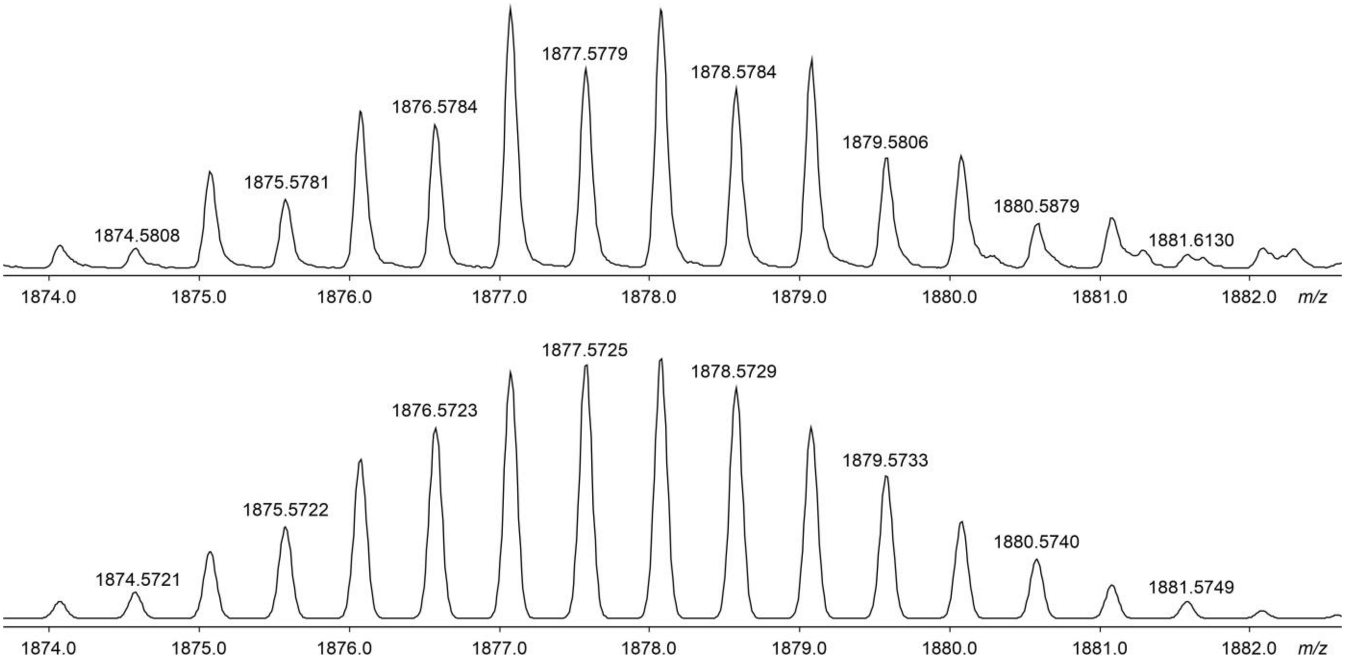

Figure S21. The high-resolution mass spectrum of **10-4Ag$_2$** (ESI+, TOF, $[C_{140}H_{124}N_{20}O_8Ag_8(CF_3CO_2)_6]^{2+}$). Top: experimental spectrum, bottom: simulated pattern.

Mass spectra of Solomon link $(PF_6)_2 \subset$ 10-4Ag$_2$

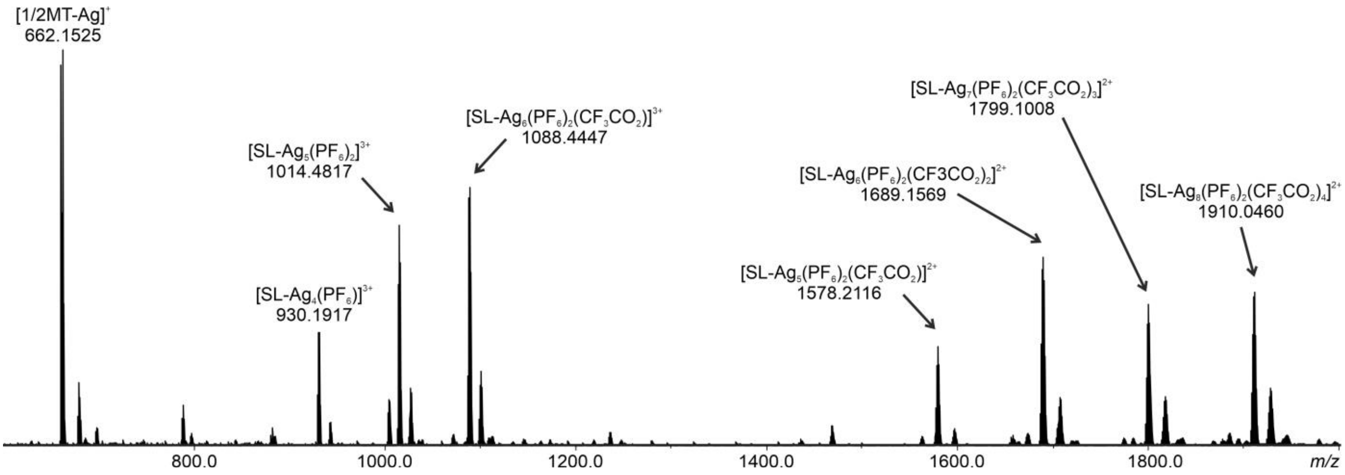

Figure S22. The 600-2000 *m/z* range of the high-resolution mass spectrum of **$(PF_6)_2 \subset$ 10-4Ag$_2$** (ESI+, TOF).

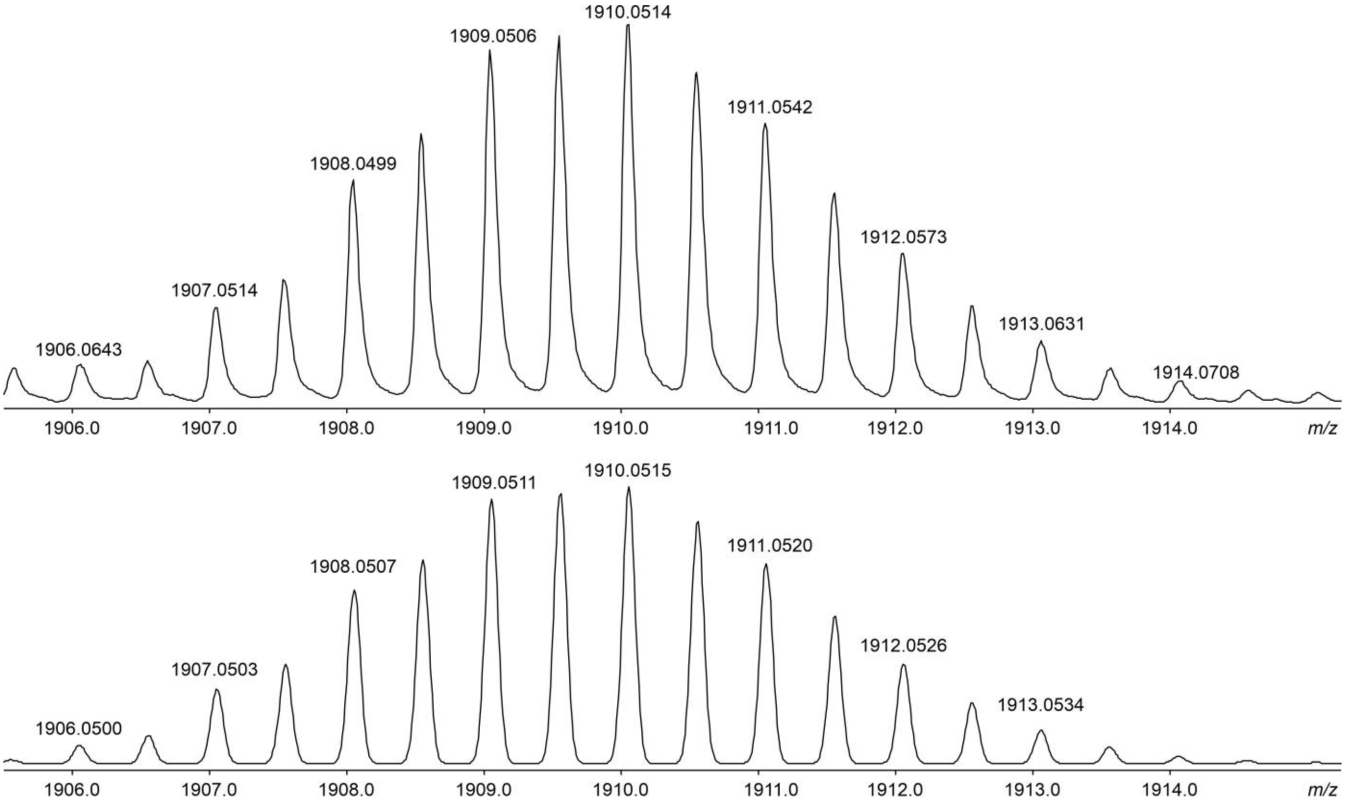

Figure S23. The high-resolution mass spectrum of **$(PF_6)_2 \subset$ 10-4Ag$_2$** (ESI+, TOF, $[C_{140}H_{124}N_{20}O_8Ag_8(PF_6)_2(CF_3COO)_4]^{2+}$). Top: experimental spectrum, bottom: simulated pattern.

Mass spectra of Solomon link $(BF_4)_n \subset$ 10-4Ag$_2$

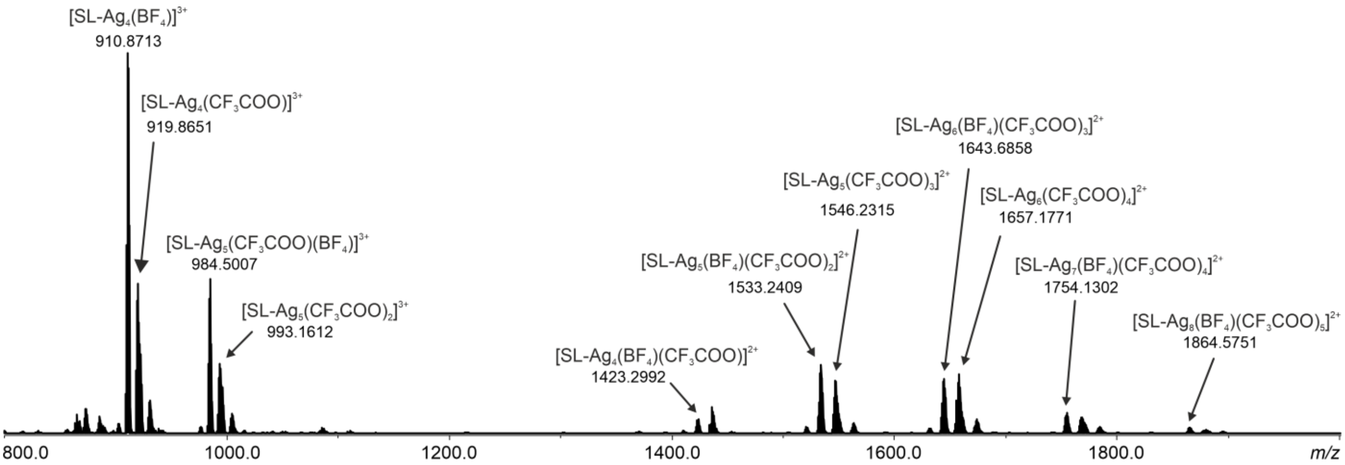

Figure S24. The 800-2000 *m/z* range of the high-resolution mass spectrum of **$(BF_4)_n \subset$ 10-4Ag$_2$** (ESI+, TOF).

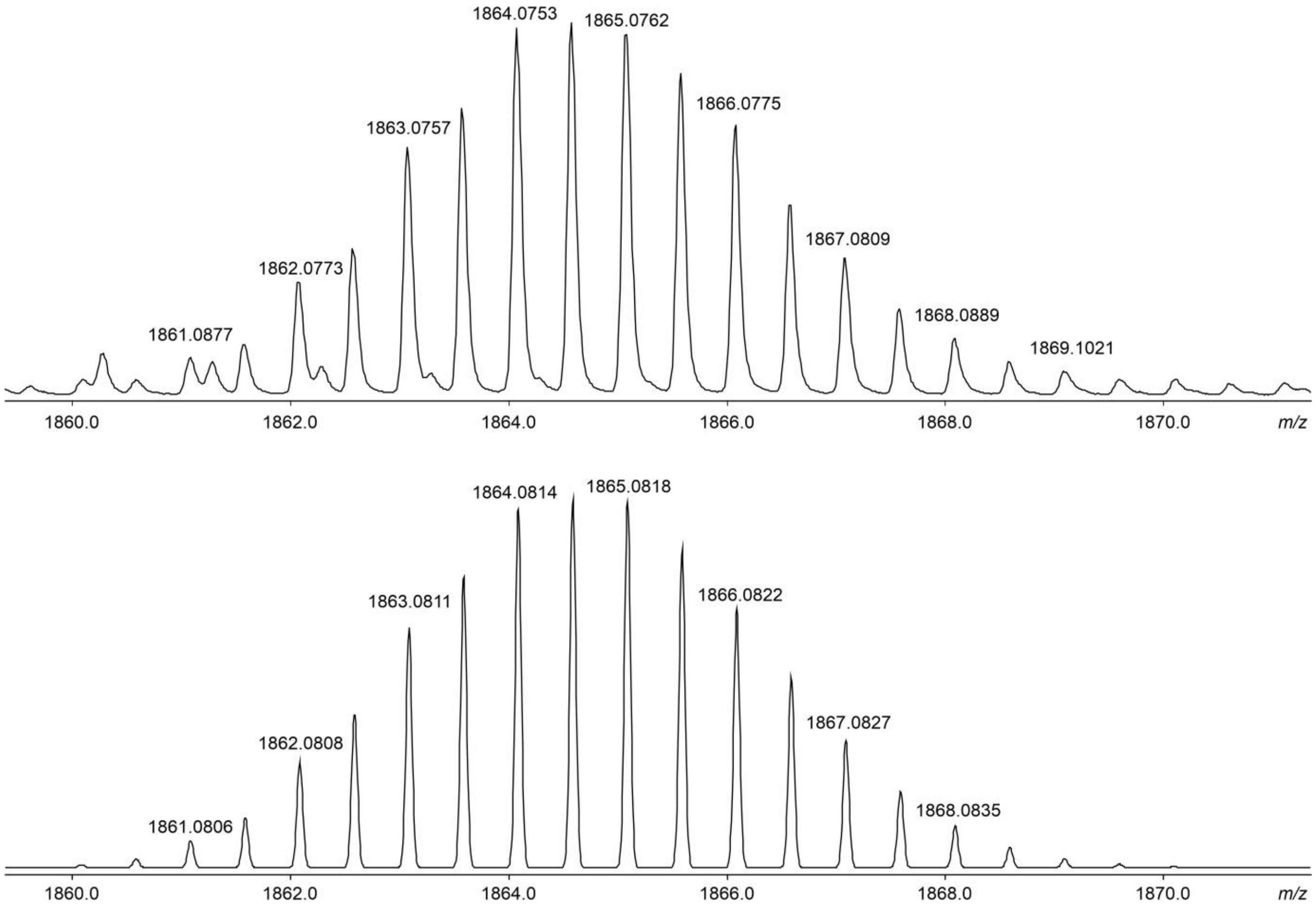

Figure S25. The high-resolution mass spectrum of **$(BF_4)_n \subset$ 10-4Ag$_2$** (ESI+, TOF, $[C_{140}H_{124}N_{20}O_8Ag_8(BF_4)(CF_3COO)_5]^{2+}$). Top: experimental spectrum, bottom: simulated pattern.

NMR spectra

NMR spectra of NBoc amine S4 and its salt 8

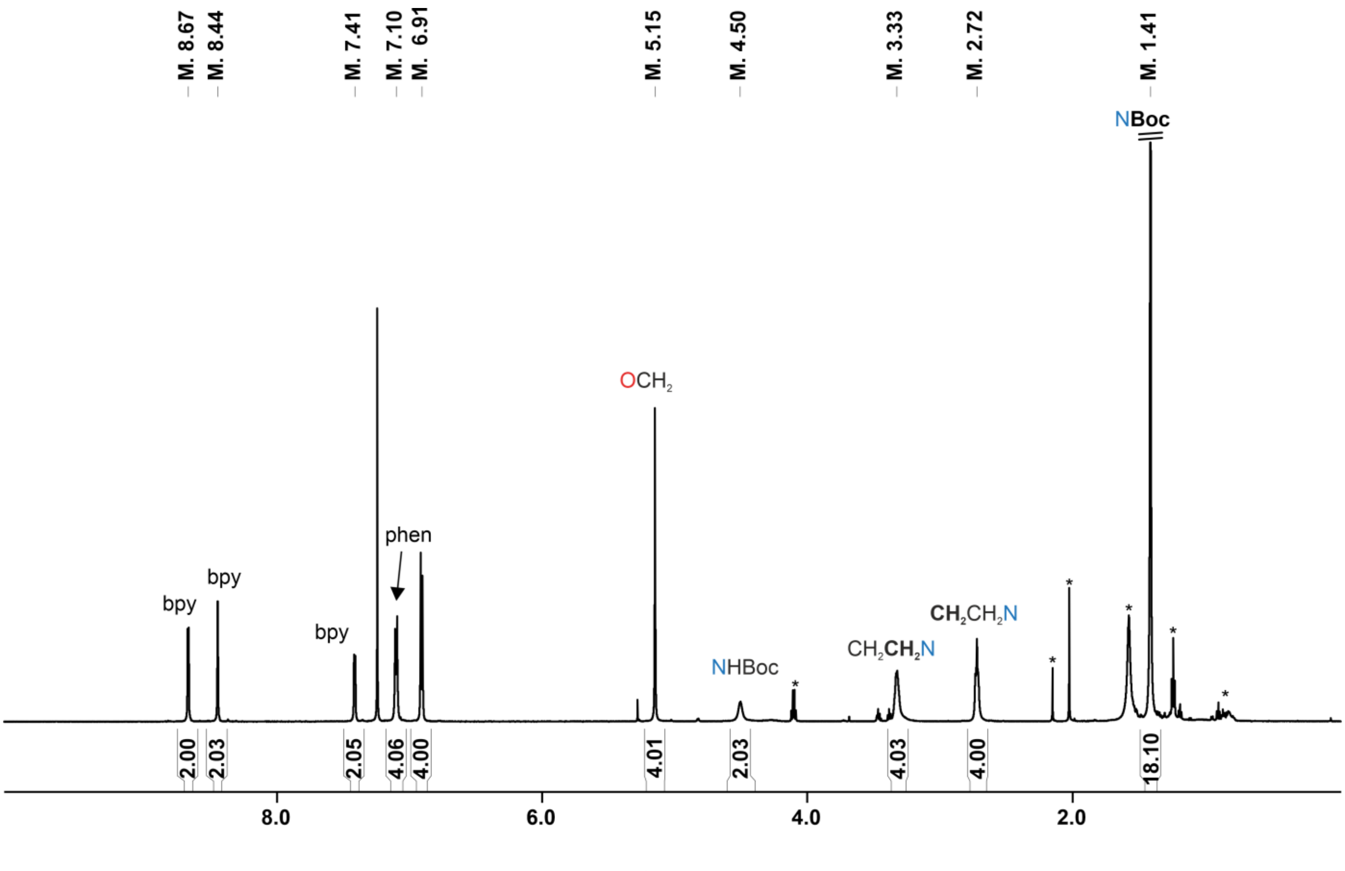

Figure S26. The ^{1}H NMR spectrum of **S4** ([D]chloroform, 300 K, 600 MHz). Signals corresponding to impurities were marked with asterisks.

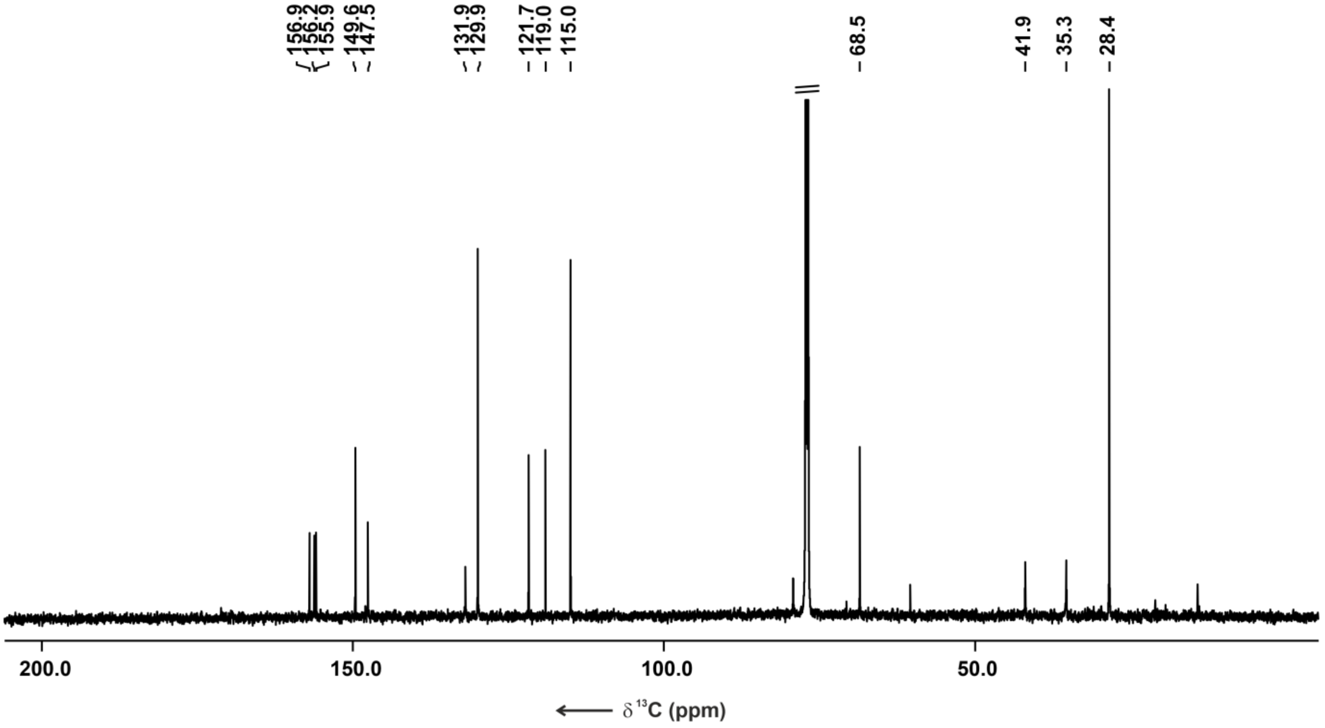

Figure S27. The ^{13}C NMR spectrum of **S4** ([D]chloroform, 300 K, 151 MHz).

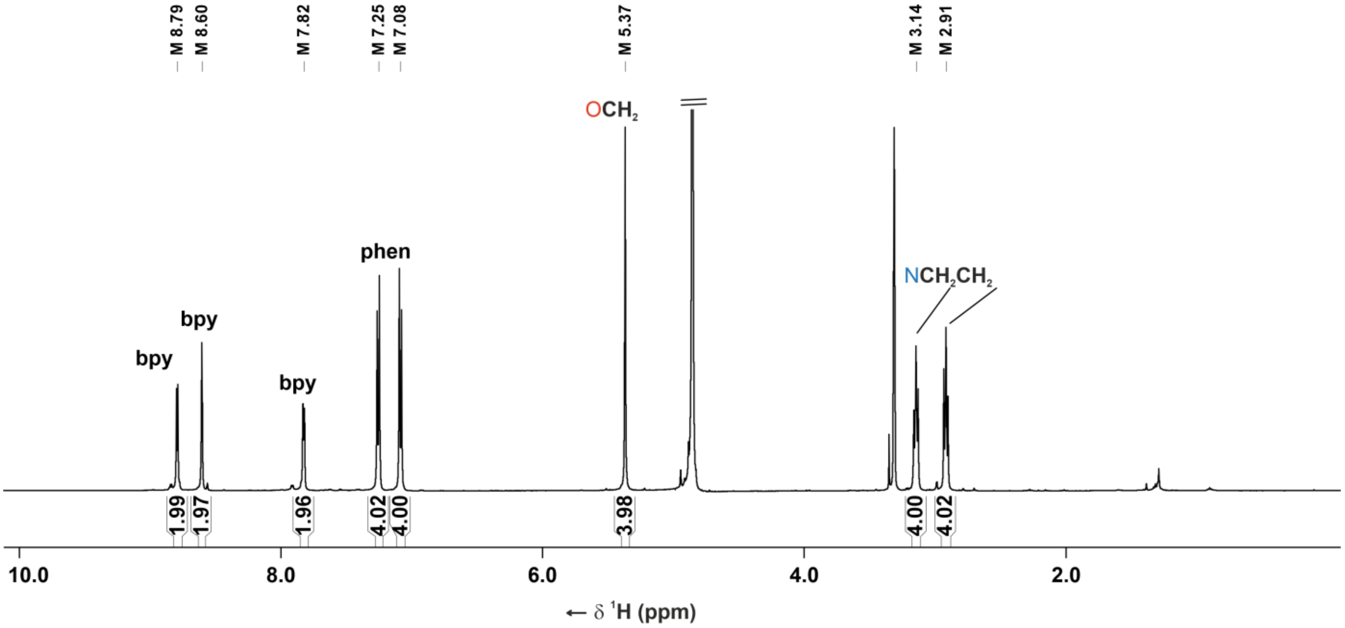

Figure S28. The ^{1}H NMR spectrum of **8** ([D4]methanol, 300 K, 500 MHz).

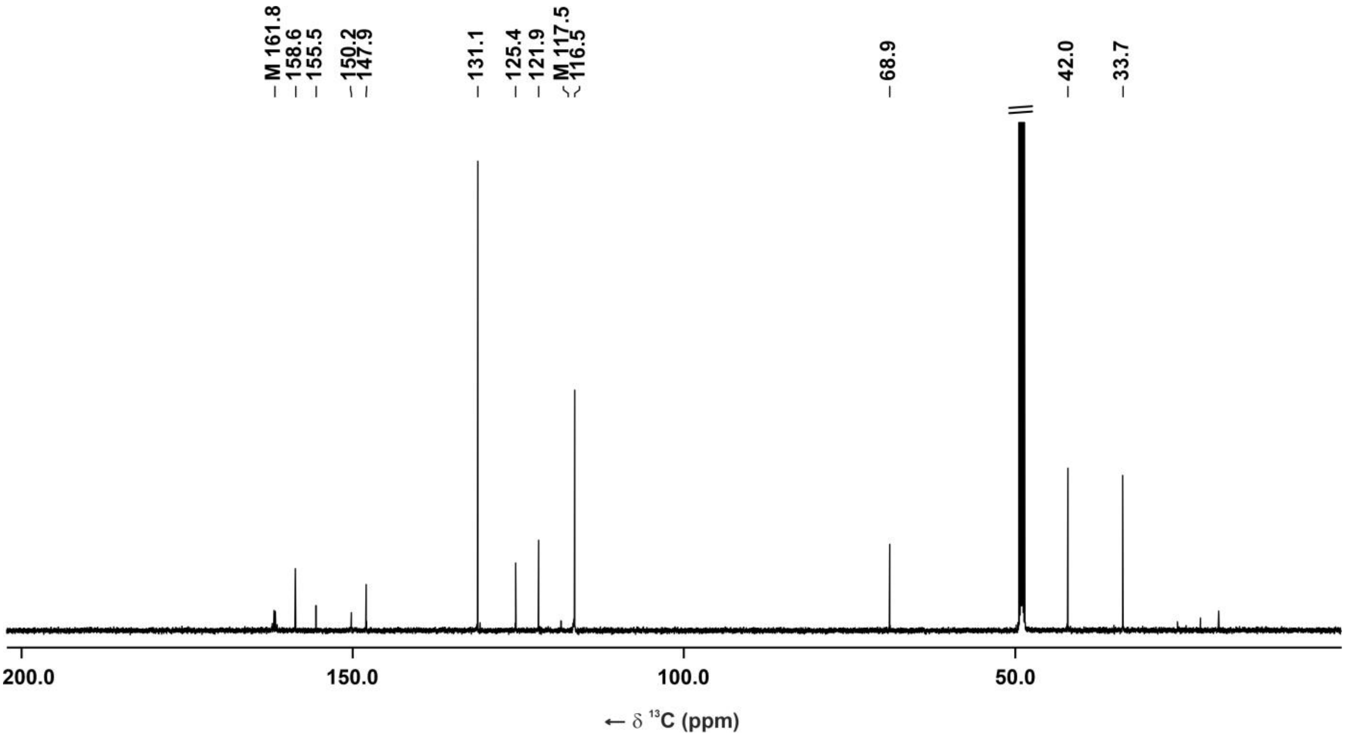

Figure S29. The ^{13}C NMR spectrum of **8** ([D4]methanol, 300 K, 151 MHz).

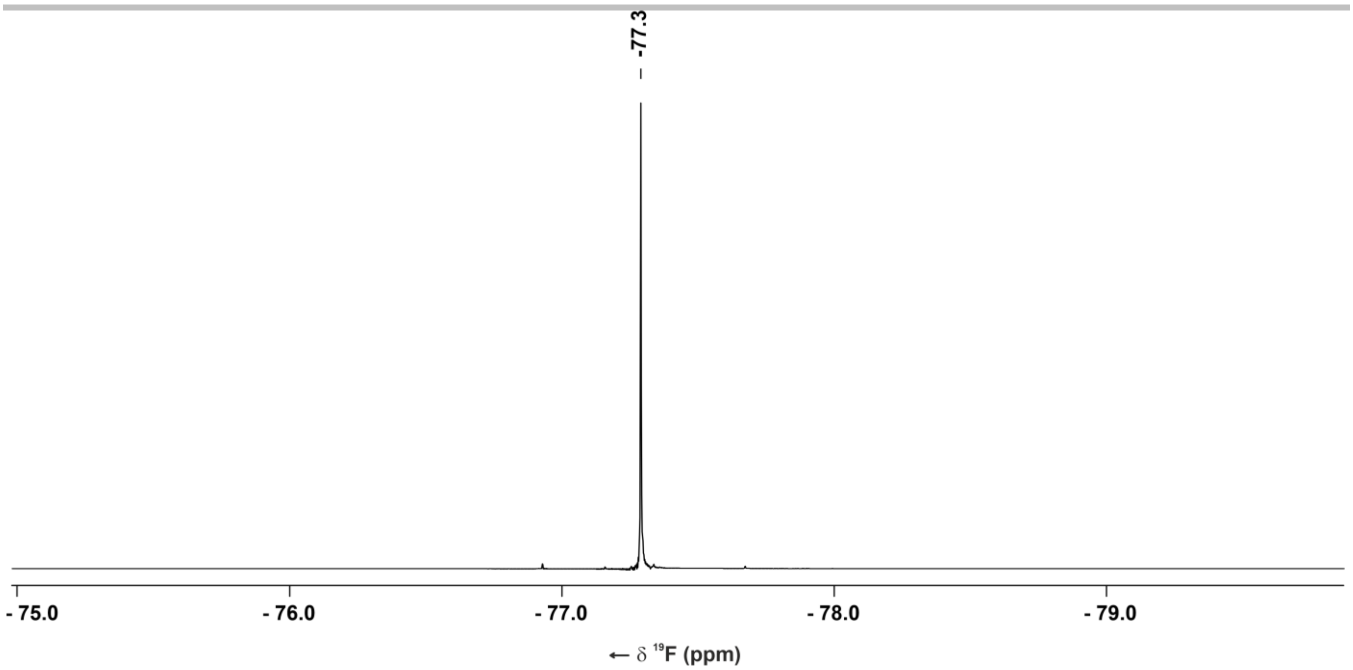

Figure S30. The ^{19}F NMR spectrum of **8** ([D_4]methanol, 300 K, 565 MHz).

NMR spectra of molecular tweezer 3-2Ag₂

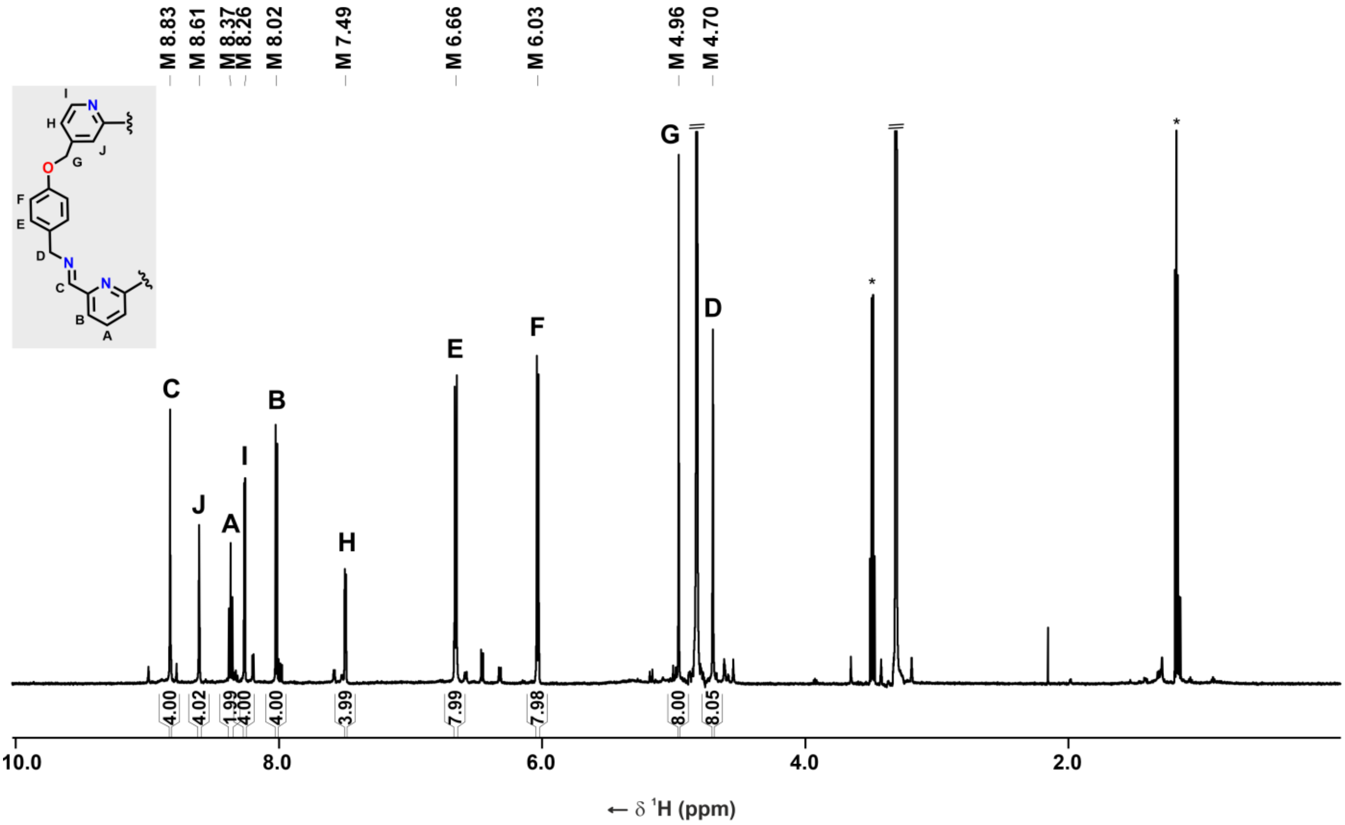

Figure S31. The ^{1}H NMR spectrum of **3-2Ag$_2$** ([D$_4$]methanol, 300 K, 600 MHz). Signals corresponding to impurities were marked with asterisks.

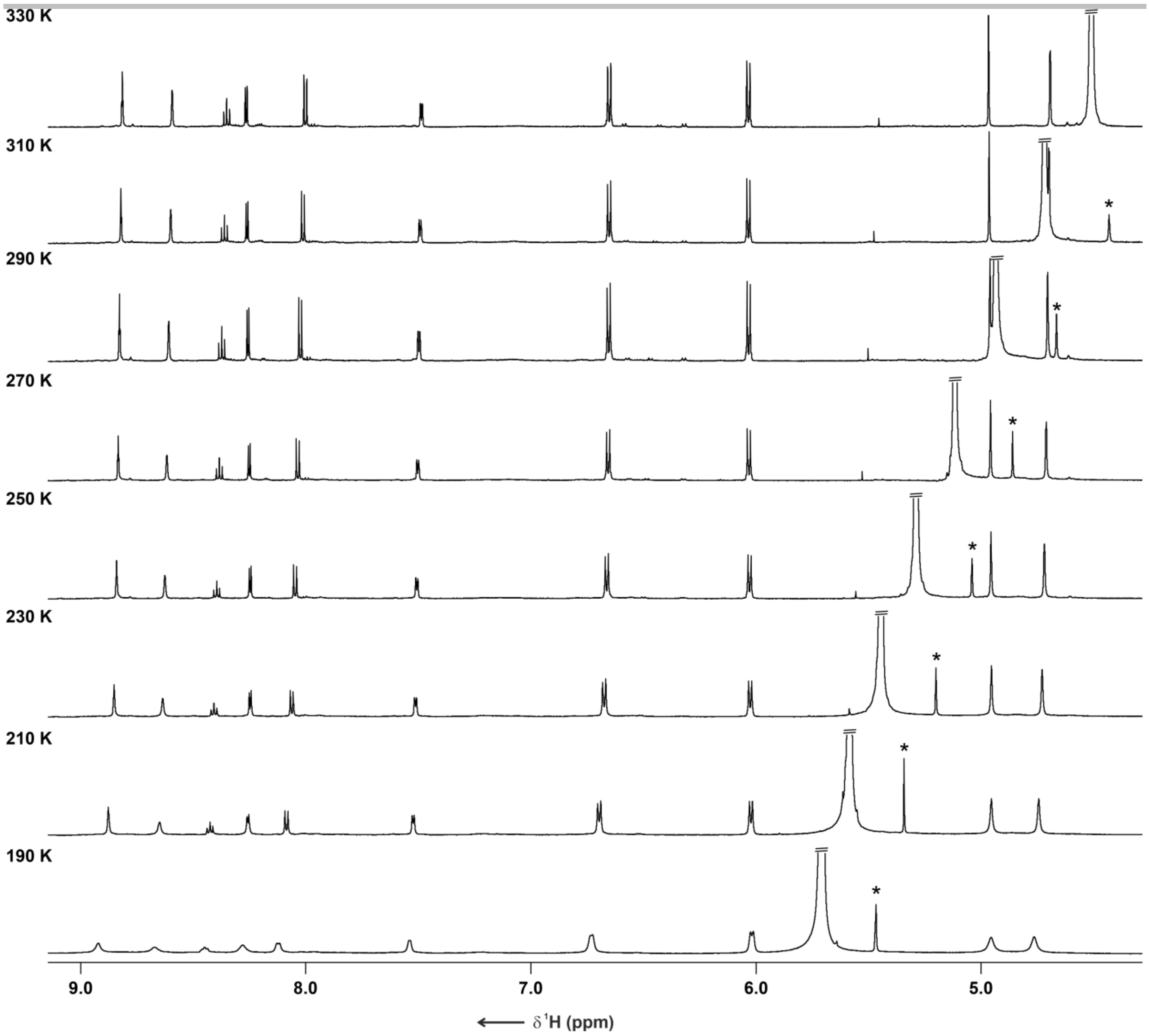

Figure S32. The ^{1}H NMR spectra of **3-2Ag₂** recorded in the 330 K – 190 K temperature range ([D₄]methanol, 600 MHz). Signals corresponding to impurities were marked with asterisks.

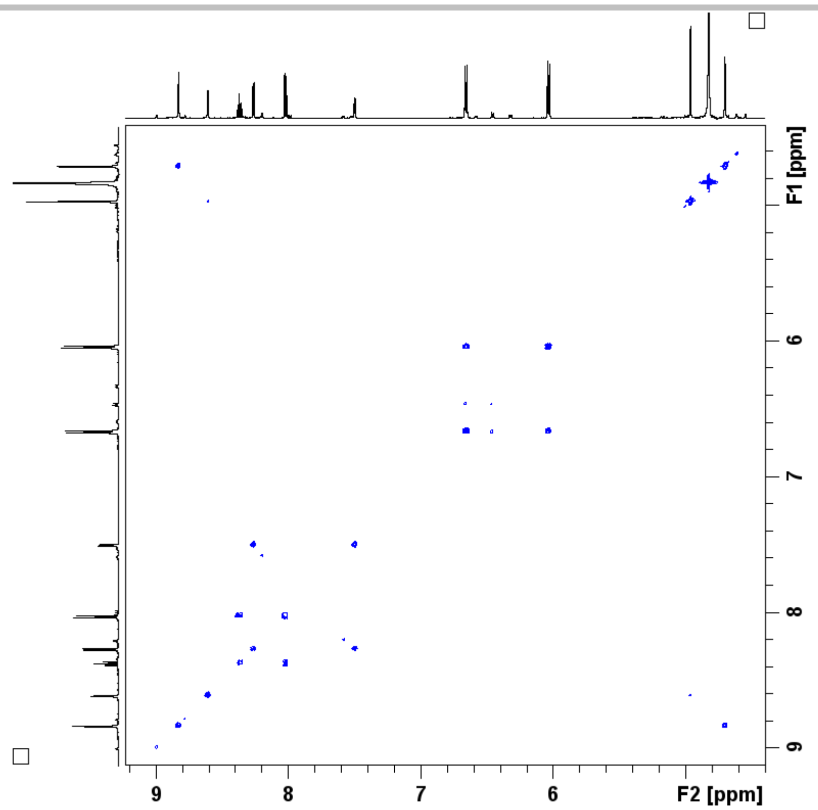

Figure S33. The ^{1}H-^{1}H COSY spectrum of **3-2Ag$_2$** ([D$_4$]methanol, 300 K, 600 MHz).

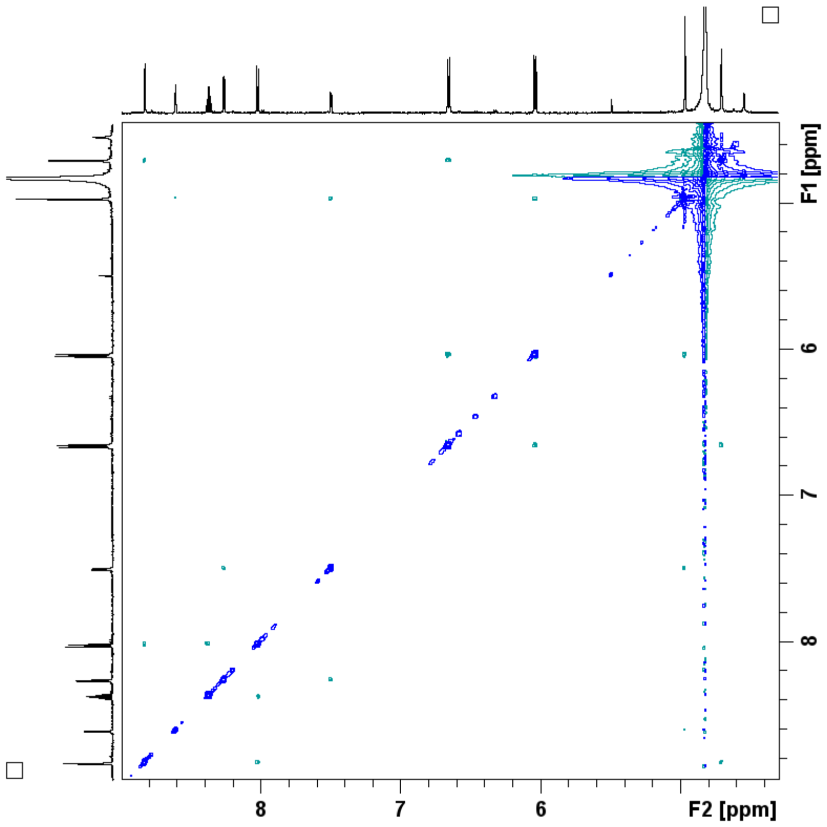

Figure S34. The ^{1}H-^{1}H ROESY spectrum of **3-2Ag$_2$** ([D$_4$]methanol, 300 K, 600 MHz).

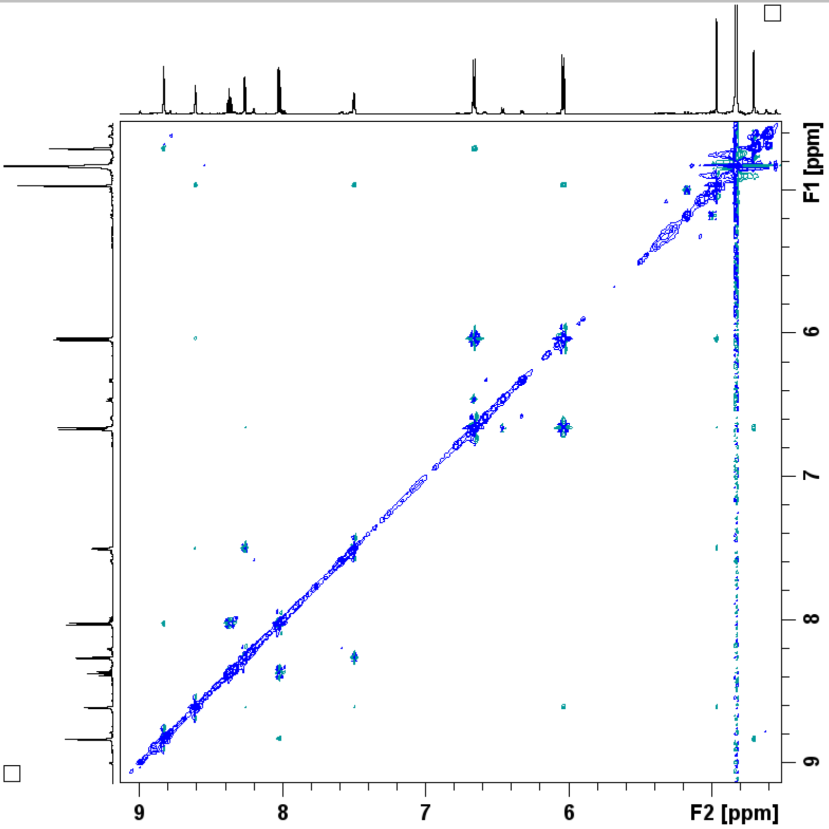

Figure S35. The [1]H-^{1}H NOESY spectrum of **3-2Ag₂** ([D₄]methanol, 300 K, 600 MHz).

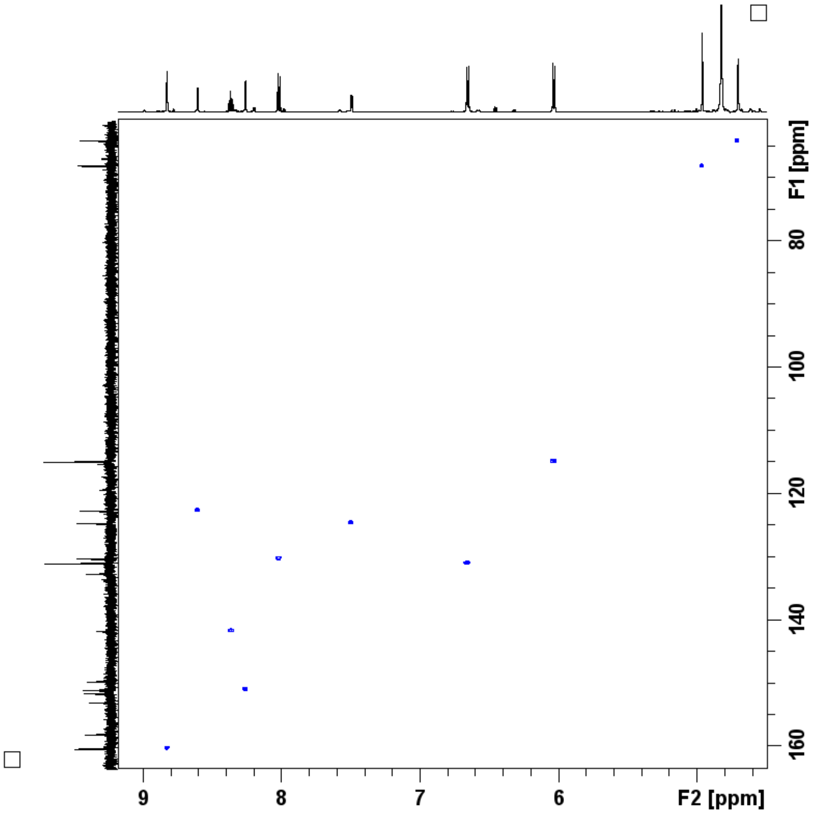

Figure S36. The ^{1}H-^{13}C HSQC spectrum of **3-2Ag₂** ([D₄]methanol, 300 K, 600 MHz).

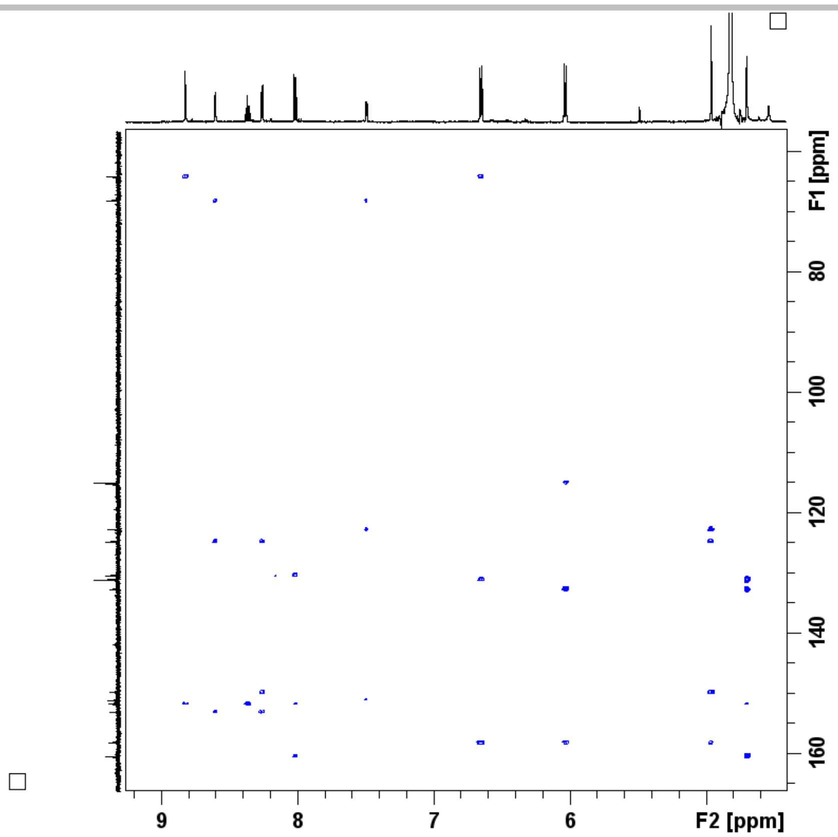

Figure S37. The ^{1}H-^{13}C HMBC spectrum of **3-2Ag$_2$** ([D$_4$]methanol, 300 K, 600 MHz).

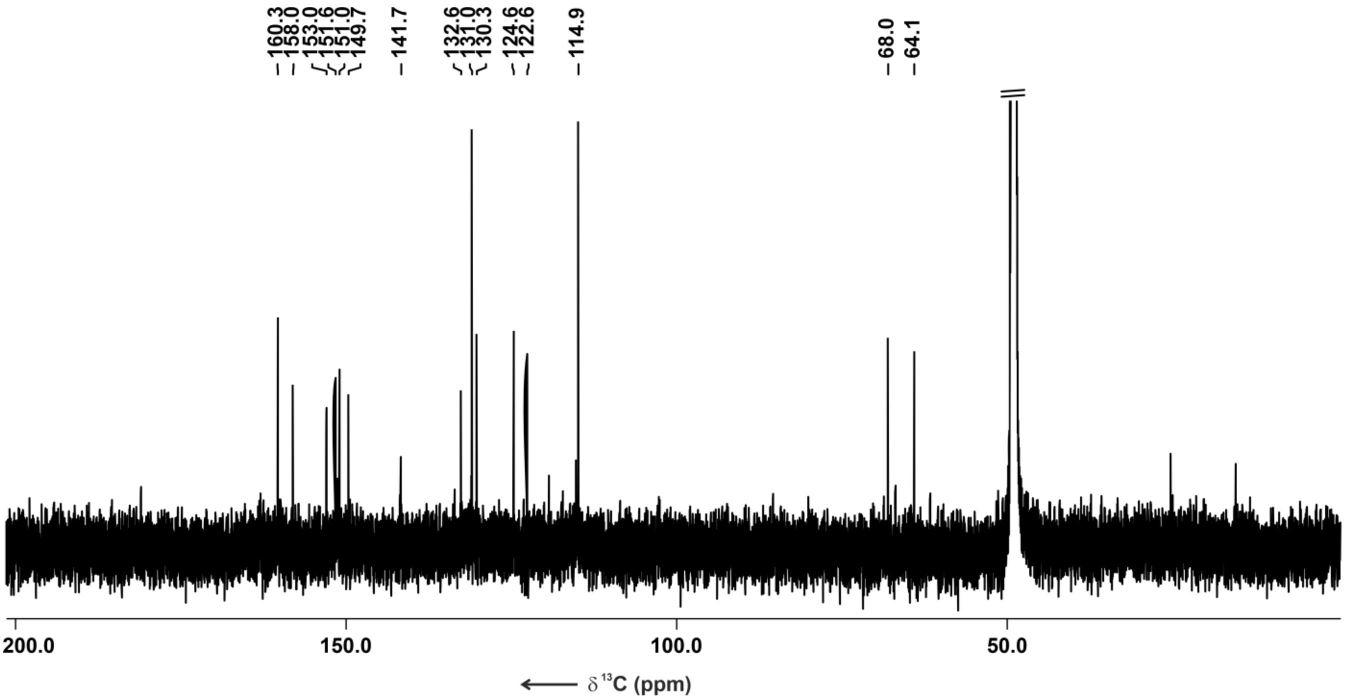

Figure S38. The ^{13}C NMR spectrum of **3-2Ag$_2$** ([D$_4$]methanol, 300 K, 151 MHz).

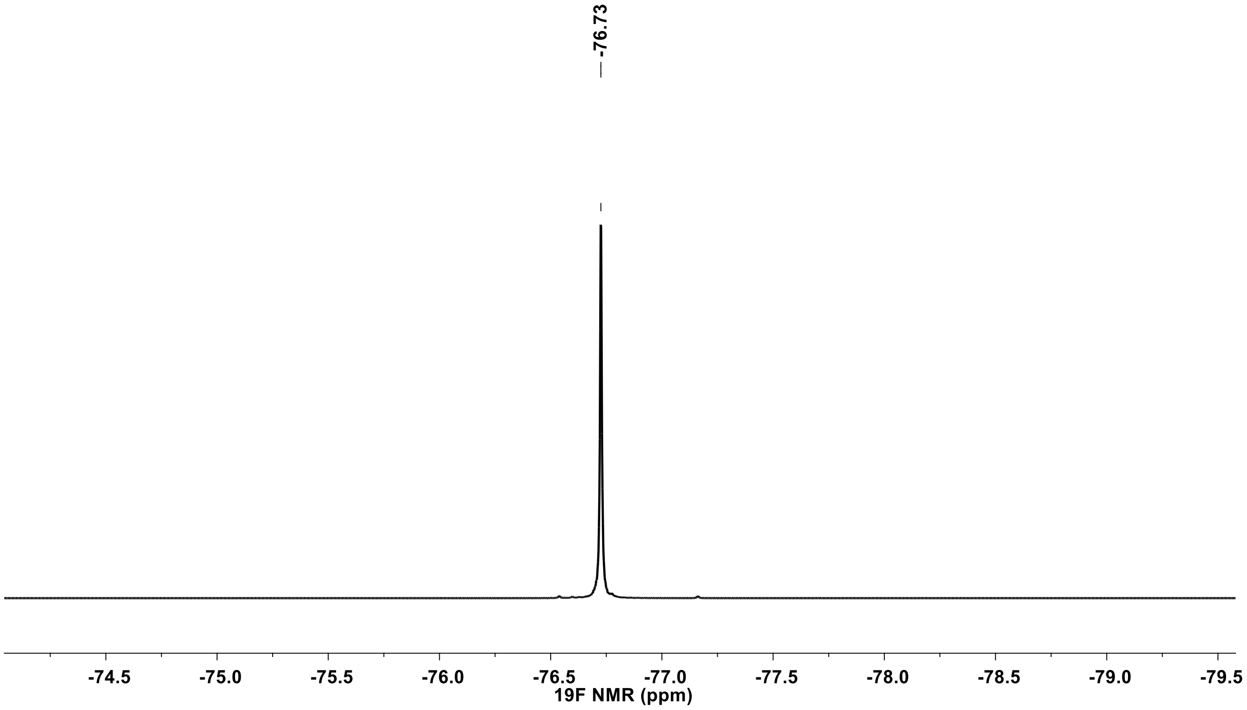

Figure S39. The ^{19}F NMR spectrum of **3-2Ag₂** ([D₄]methanol, 300 K, 471 MHz).

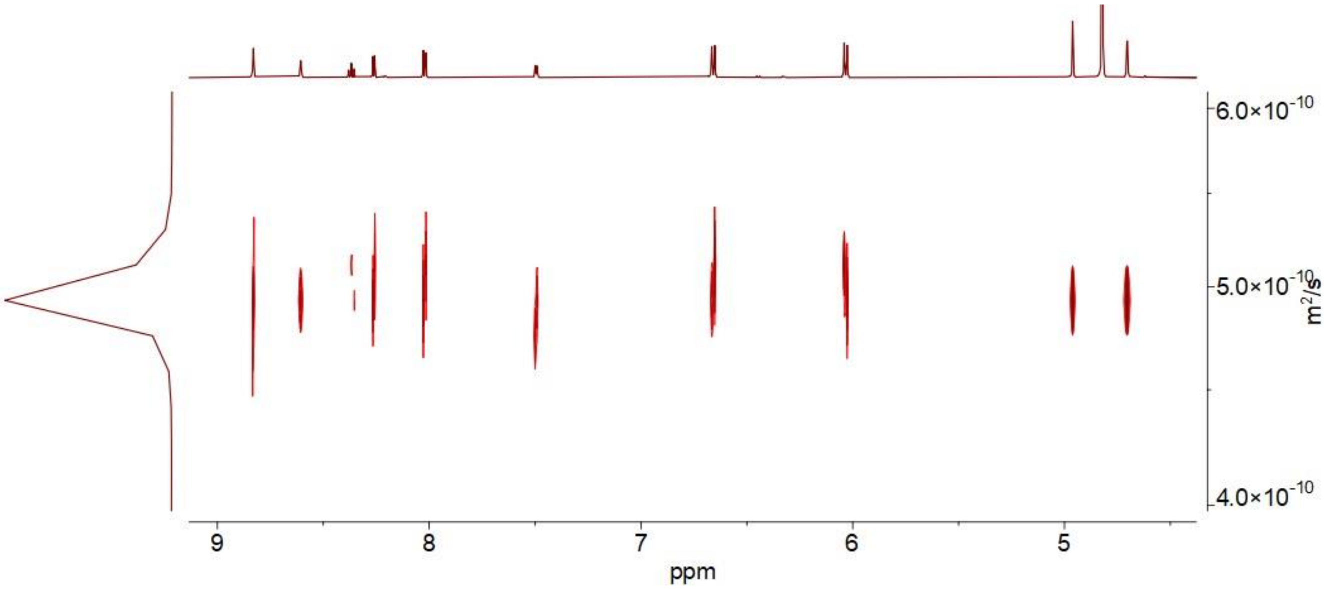

Figure S40. The DOSY NMR spectrum of molecular tweezer **3-2Ag₂** ([D₄]methanol, 300 K, 600 MHz).

NMR spectra of mixtures containing trefoil knot 4-3Ag

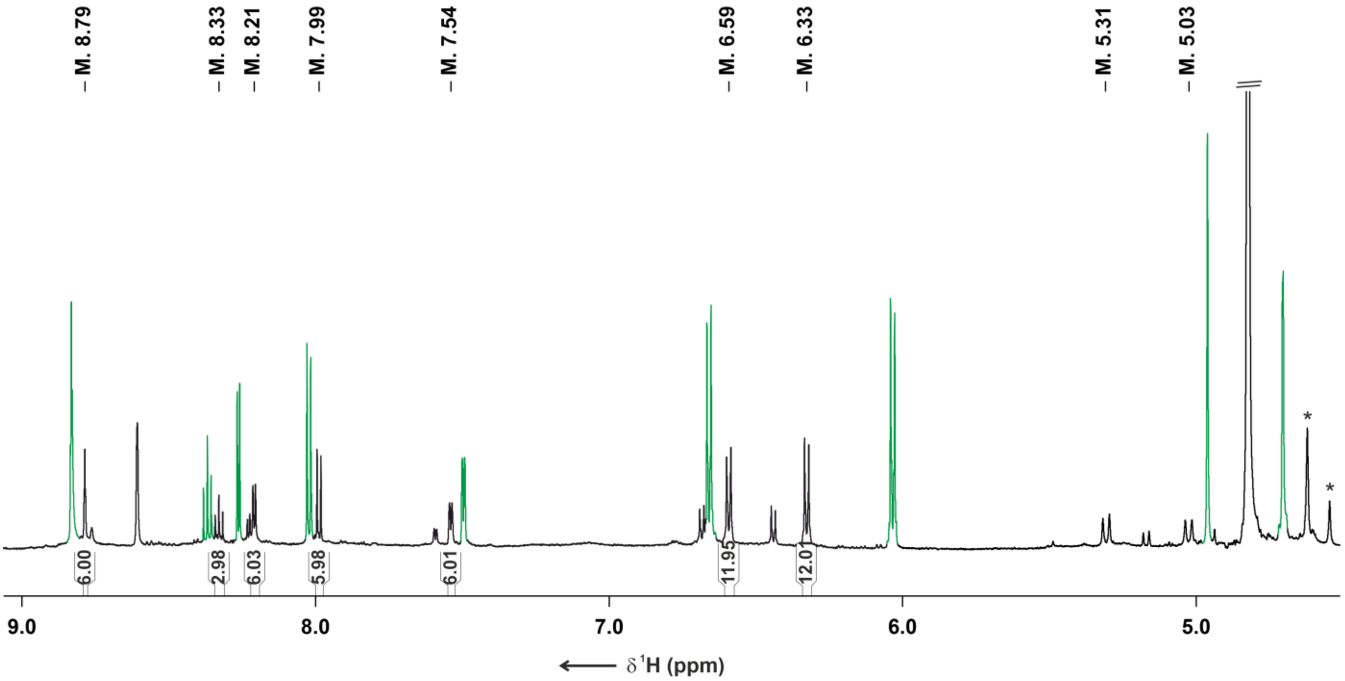

Figure S41. The ^{1}H NMR spectrum of a mixture of **3-2Ag$_2$** (green), **4-3Ag** and tetranuclear Solomon link ([D$_4$]methanol, 300 K, 600 MHz). Signals corresponding to impurities were marked with asterisks.

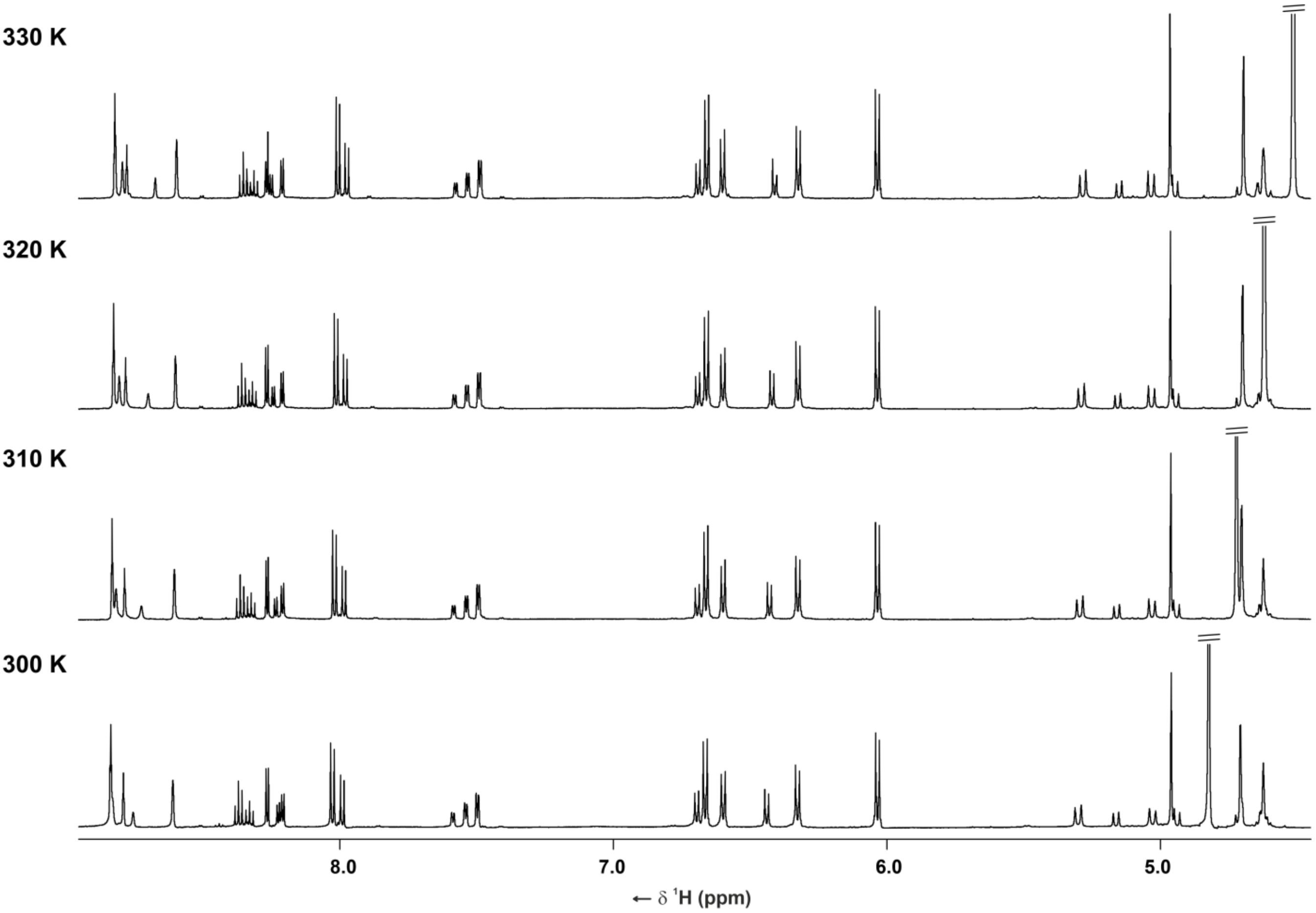

Figure S42. The ^{1}H NMR spectra of the mixture of **3-2Ag$_2$**, **4-3Ag** and tetranuclear Solomon link recorded in the 330 K – 300 K temperature range ([D$_4$]methanol, 600 MHz).

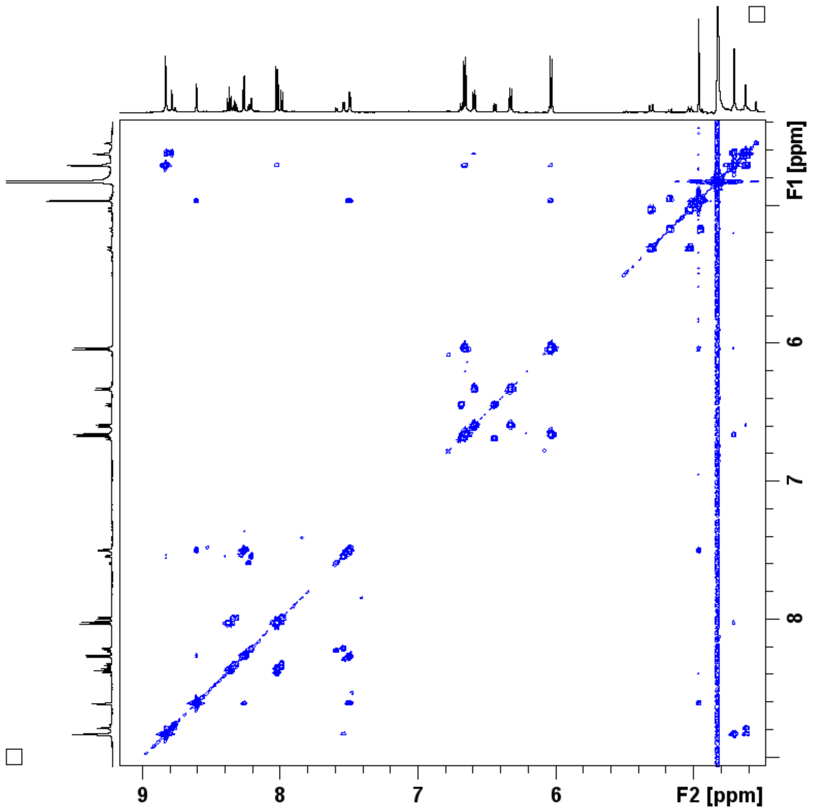

Figure S43. The ^{1}H-^{1}H COSY spectrum of **4-3Ag** ([D$_4$]methanol, 300 K, 600 MHz).

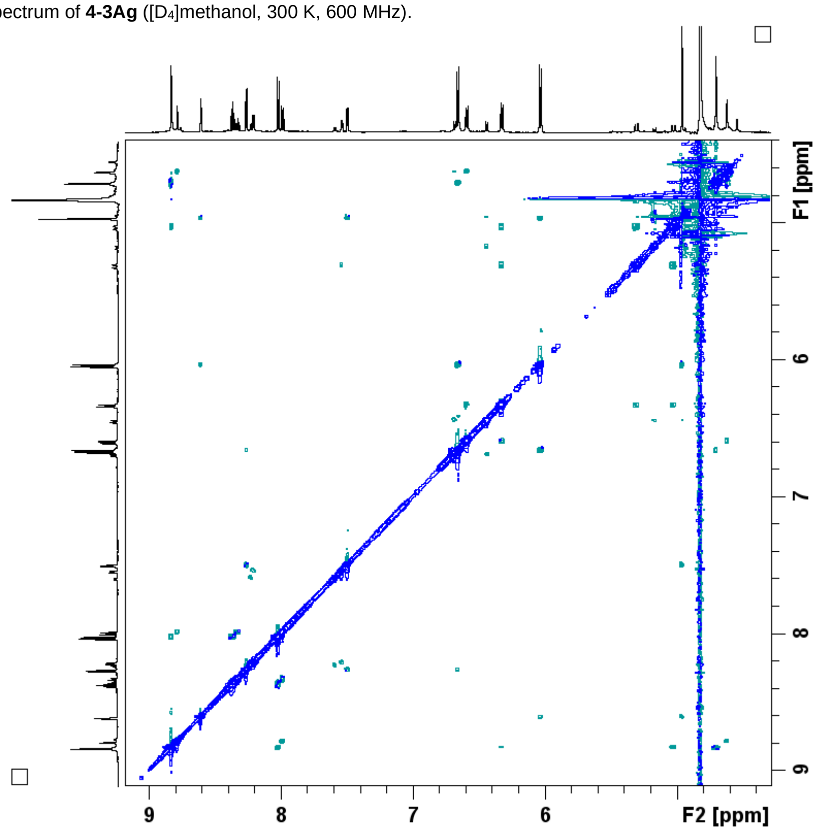

Figure S44. The ^{1}H-^{1}H ROESY spectrum of **4-3Ag** ([D$_4$]methanol, 300 K, 600 MHz).

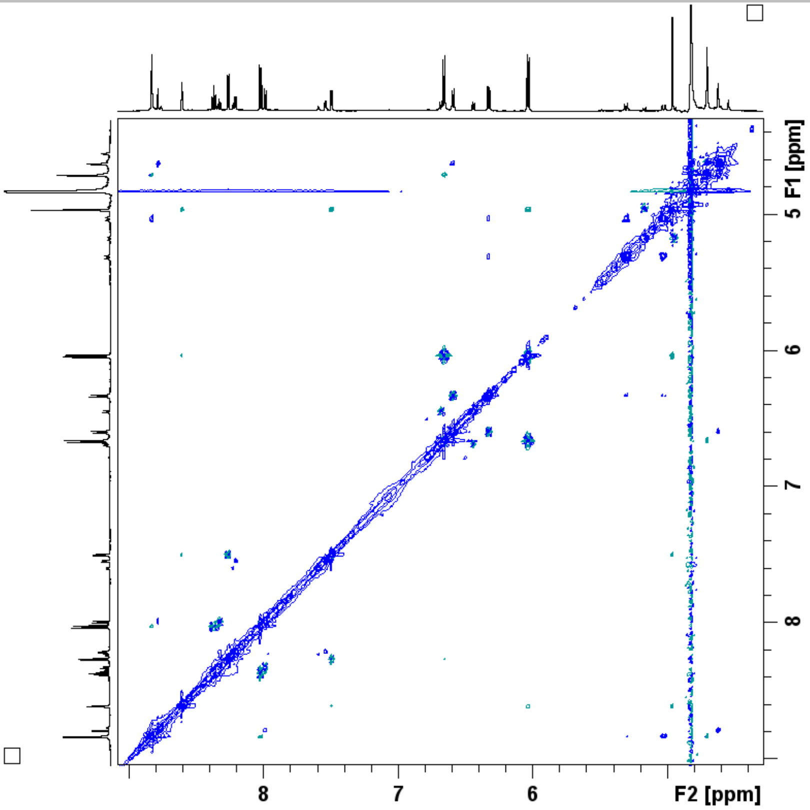

Figure S45. The ^{1}H-^{1}H NOESY spectrum of **4-3Ag** ([D$_4$]methanol, 300 K, 600 MHz).

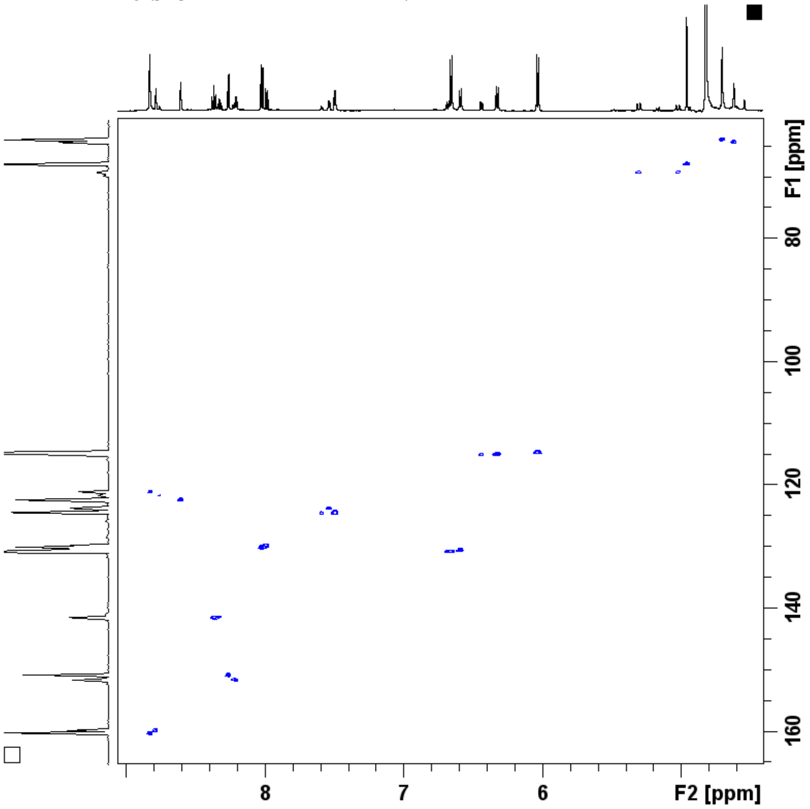

Figure S46. The ^{1}H-^{13}C HSQC spectrum of **4-3Ag** ([D$_4$]methanol, 300 K, 600 MHz).

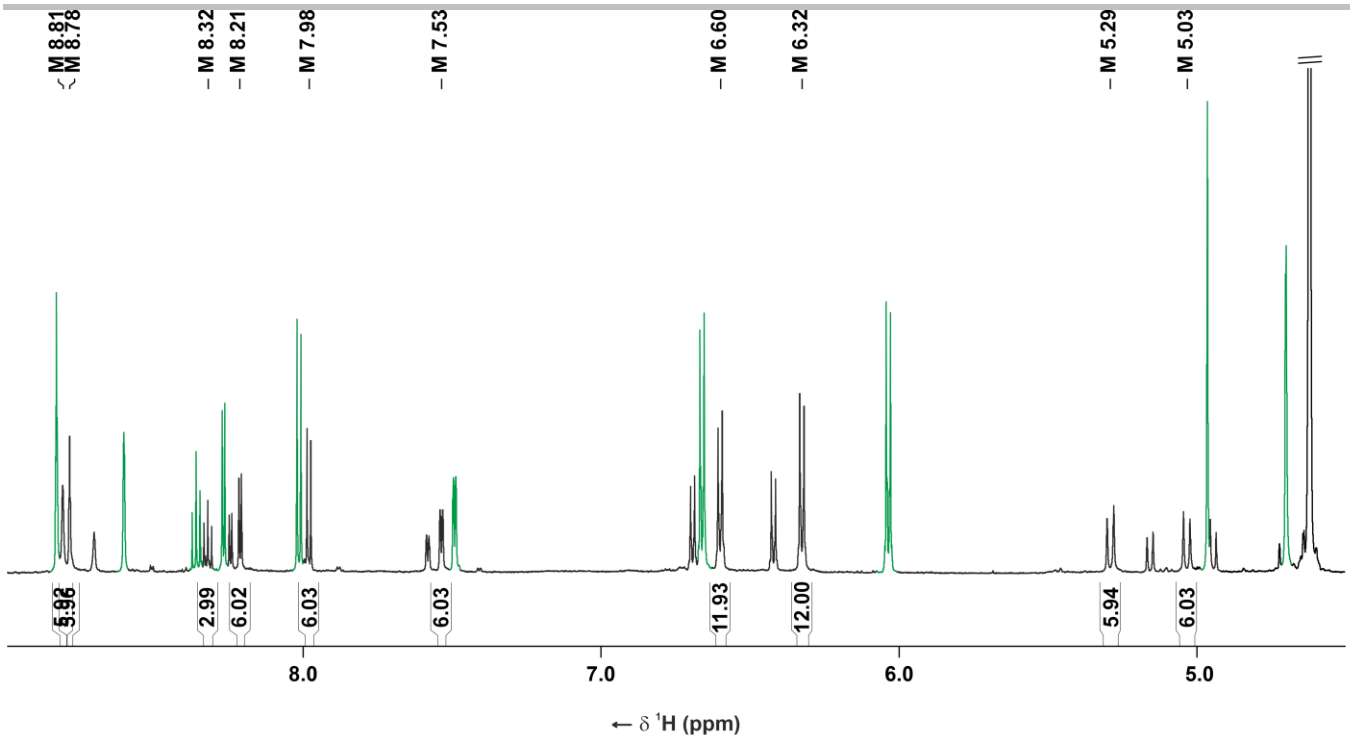

Figure S47. The ^{1}H NMR spectrum of a mixture of **3-2Ag$_2$** (green), **4-3Ag**, and tetranuclear Solomon link ([D$_4$]methanol, 320 K, 600 MHz).

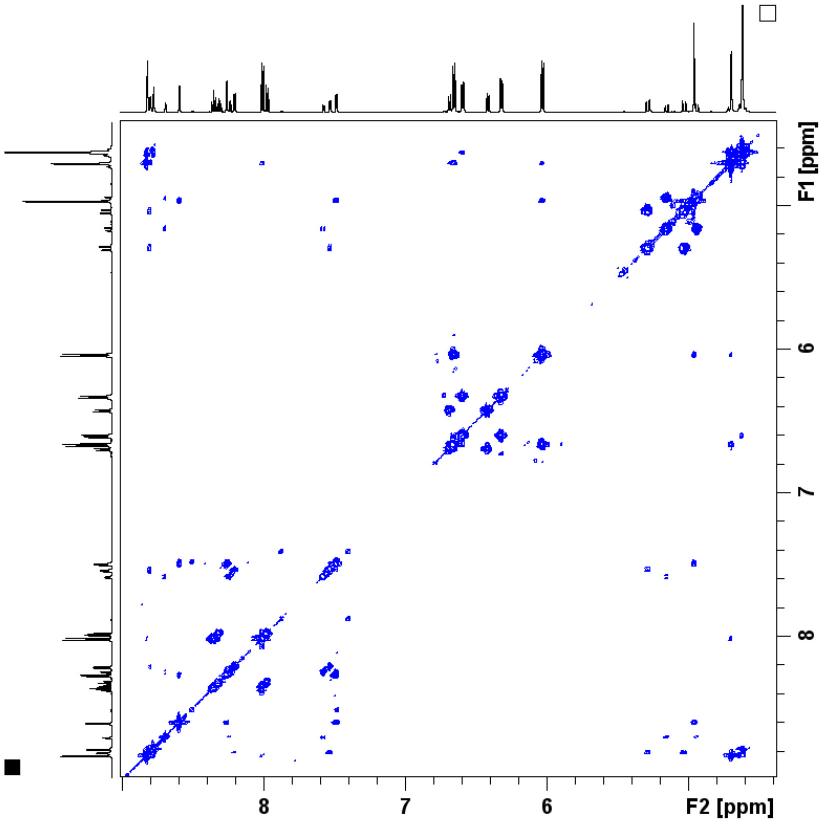

Figure S48. The ^{1}H-^{1}H COSY spectrum of **4-3Ag** ([D$_4$]methanol, 320 K, 600 MHz).

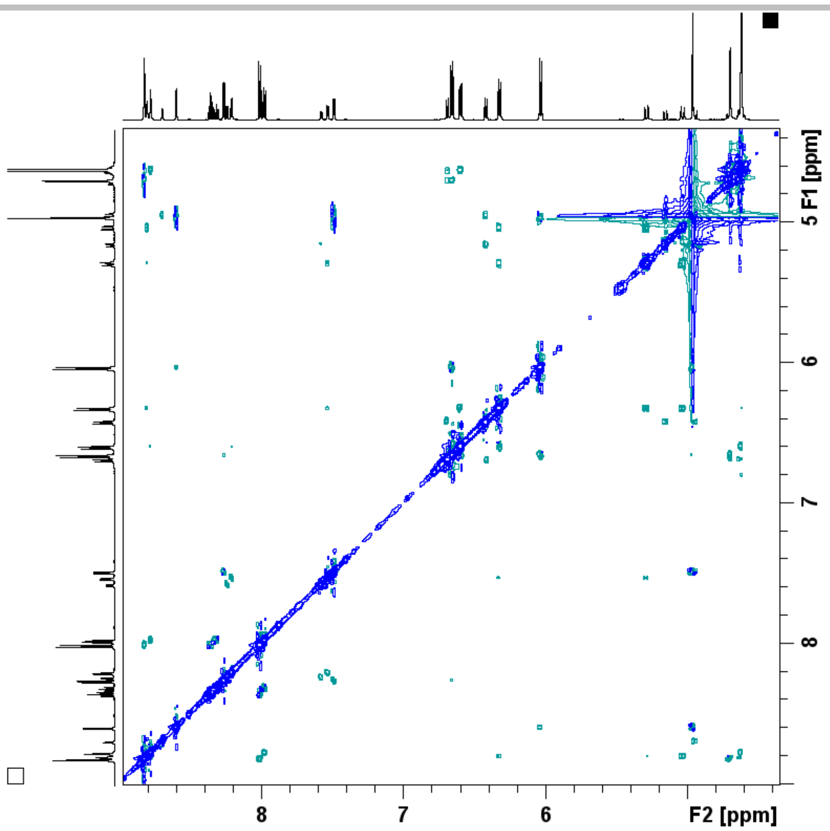

Figure S49. The ^{1}H-^{1}H ROESY spectrum of **4-3Ag** ([D$_4$]methanol, 320 K, 600 MHz).

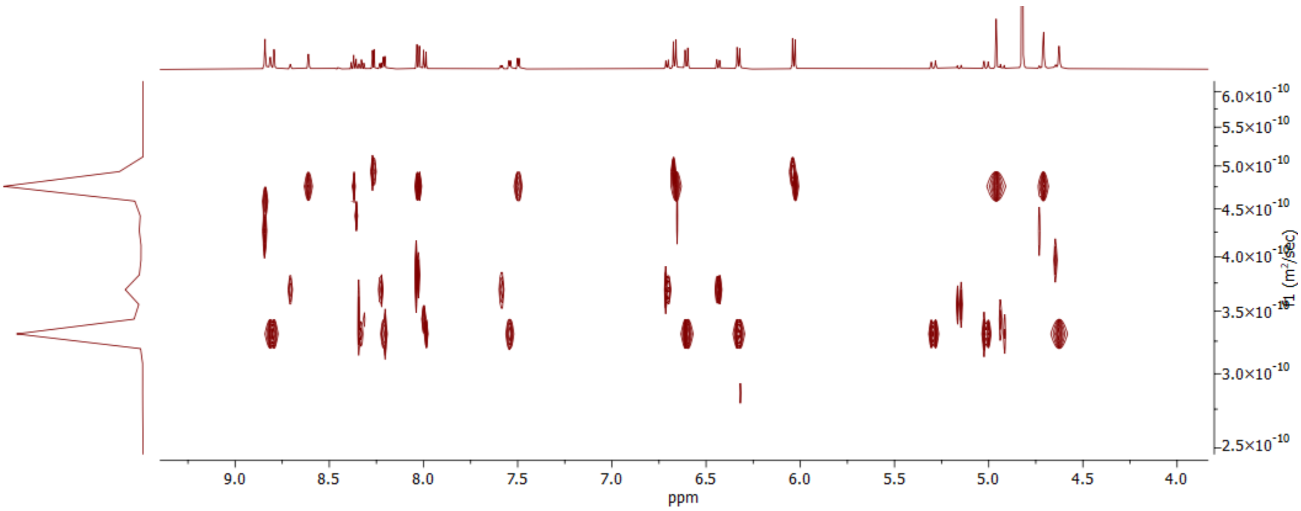

Figure S50. The DOSY NMR spectrum of molecular tweezer **3-2Ag$_2$**, trefoil knot **4-3Ag**, and tetranuclear Solomon link ([D$_4$]methanol, 300 K, 600 MHz).

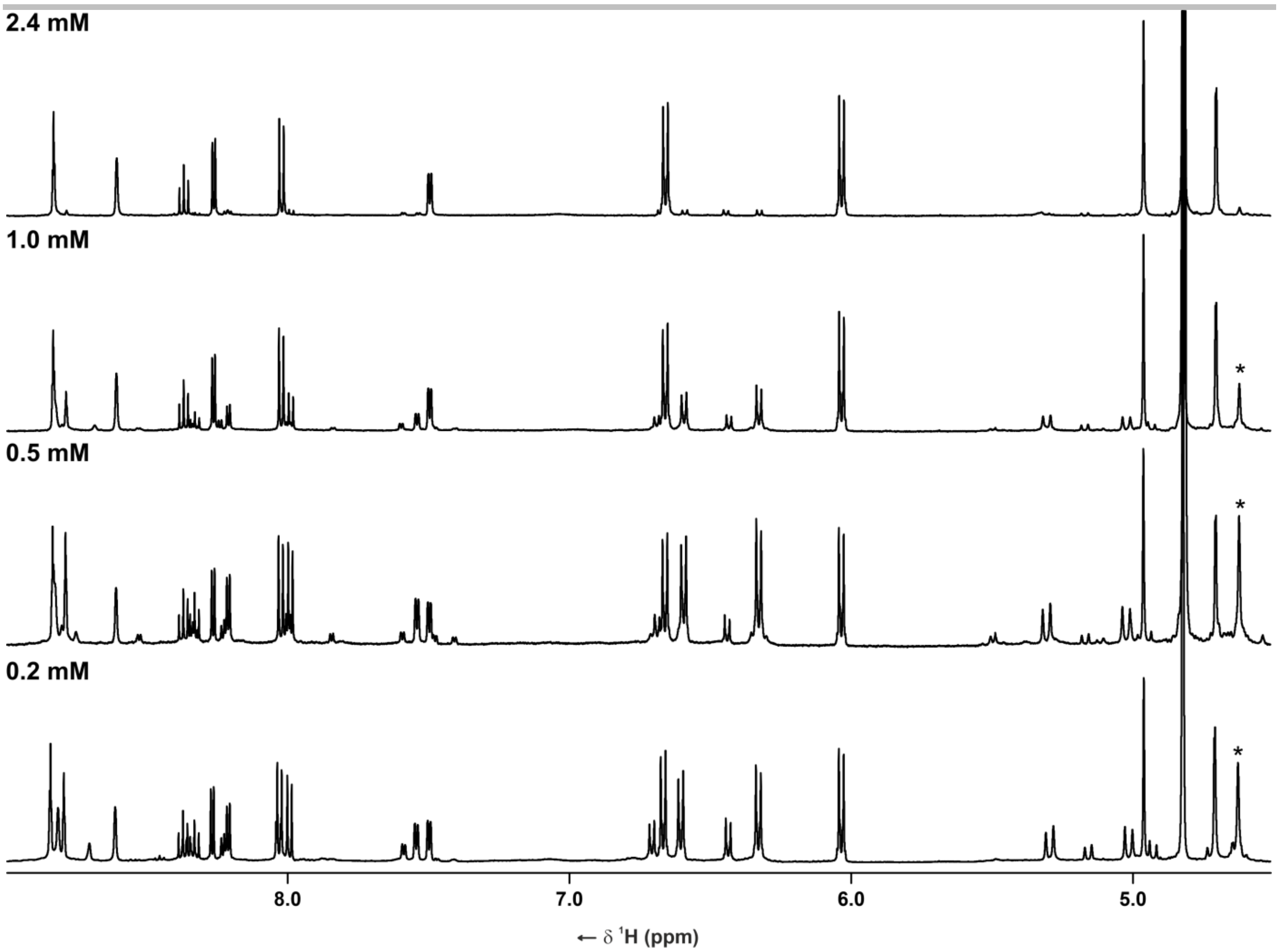

Figure S51. The ^{1}H NMR spectra of a mixture of **3-2Ag₂**, **4-3Ag**, and Solomon link recorded for the reactions carried out at the 0.2 – 2.4 mM concentration (relative to **1**) ([D4]methanol, 300 K, 500 MHz). Signals corresponding to impurities were marked with asterisks.

NMR spectra of [2]catenane 6-2Ag$_2^{OTf}$ (obtained with silver(I) triflate).

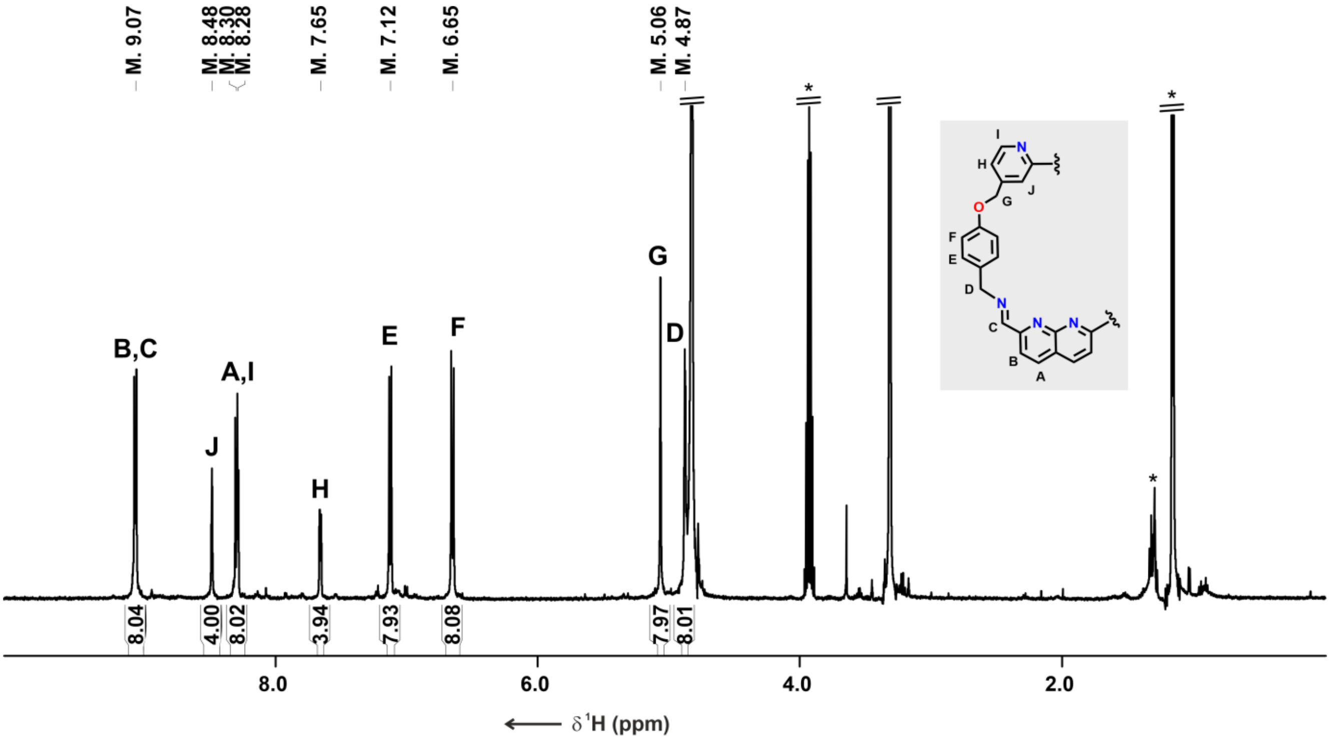

Figure S52. The ^{1}H NMR spectrum of **6-2Ag$_2^{OTf}$** ([D$_4$]methanol, 300 K, 600 MHz). Signals corresponding to impurities were marked with asterisks.

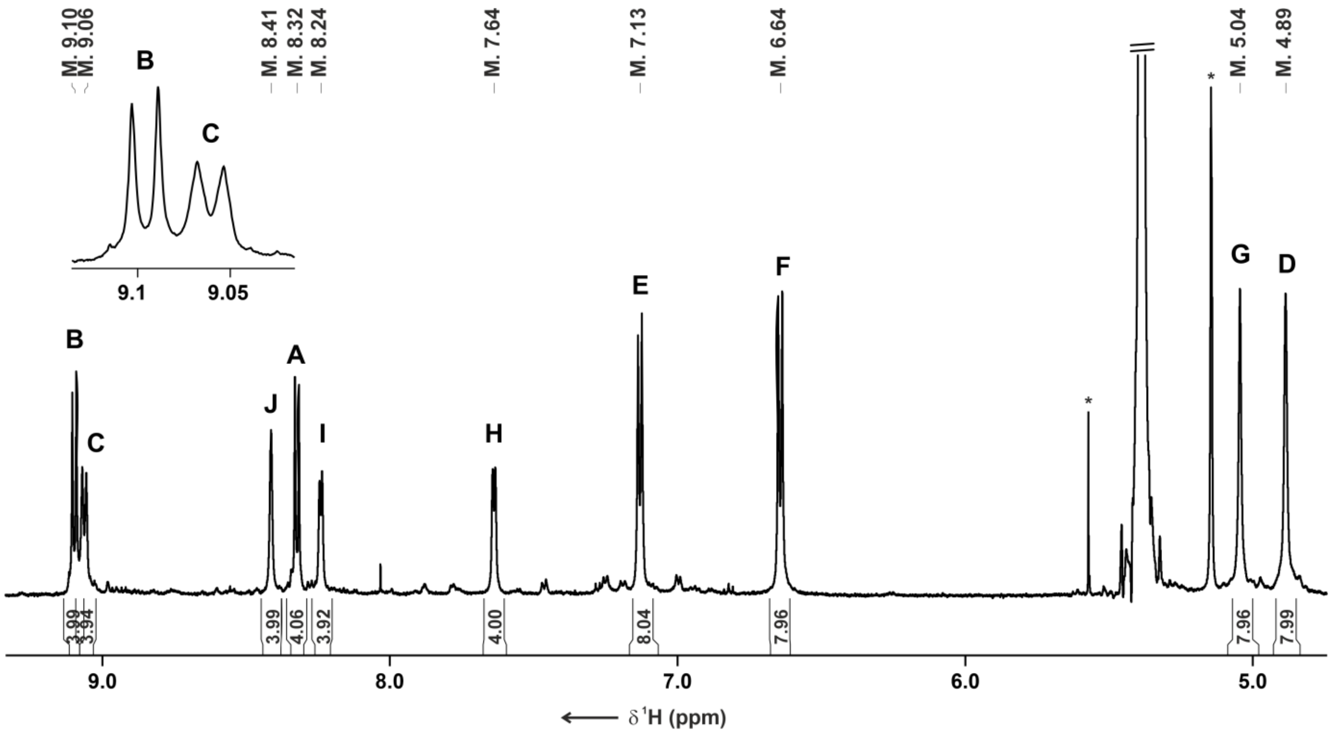

Figure S53. The ^{1}H NMR spectrum of **6-2Ag$_2^{OTf}$** ([D$_4$]methanol, 240 K, 600 MHz). Signals corresponding to impurities were marked with asterisks.

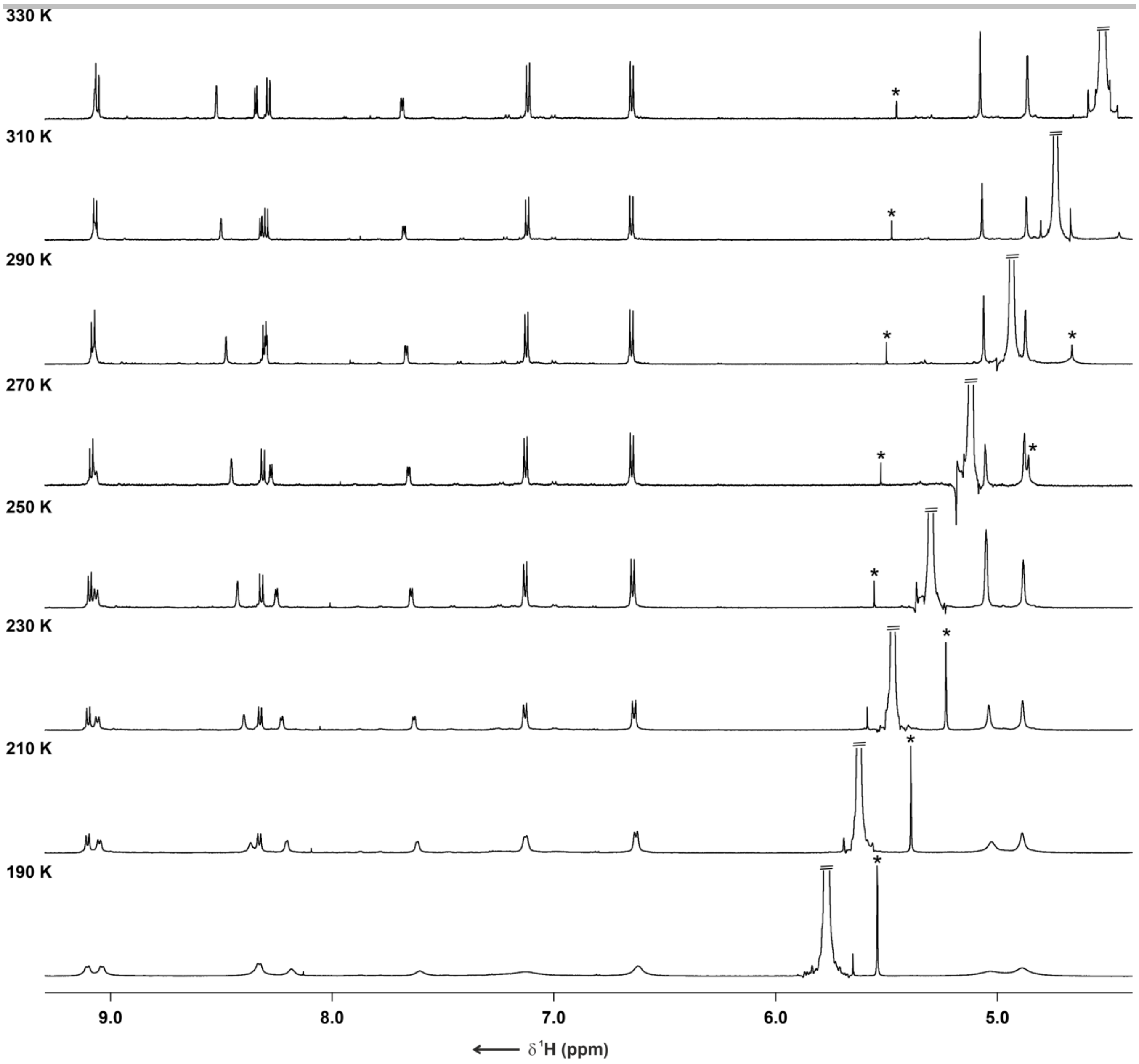

Figure S54. The ^{1}H NMR spectra of **6-2Ag$_2$^{OTf}** recorded in the 330 K – 190 K temperature range ([D$_4$]methanol, 600 MHz).

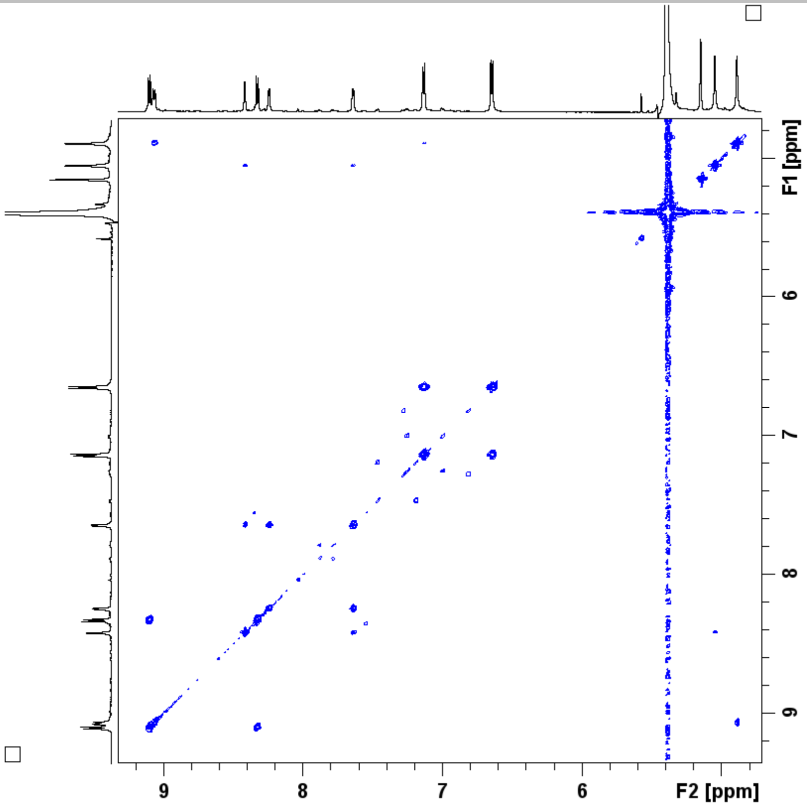

Figure S55. The ^{1}H-^{1}H COSY spectrum of **6-2Ag$_2^{OTf}$** ([D_4]methanol, 240 K, 600 MHz).

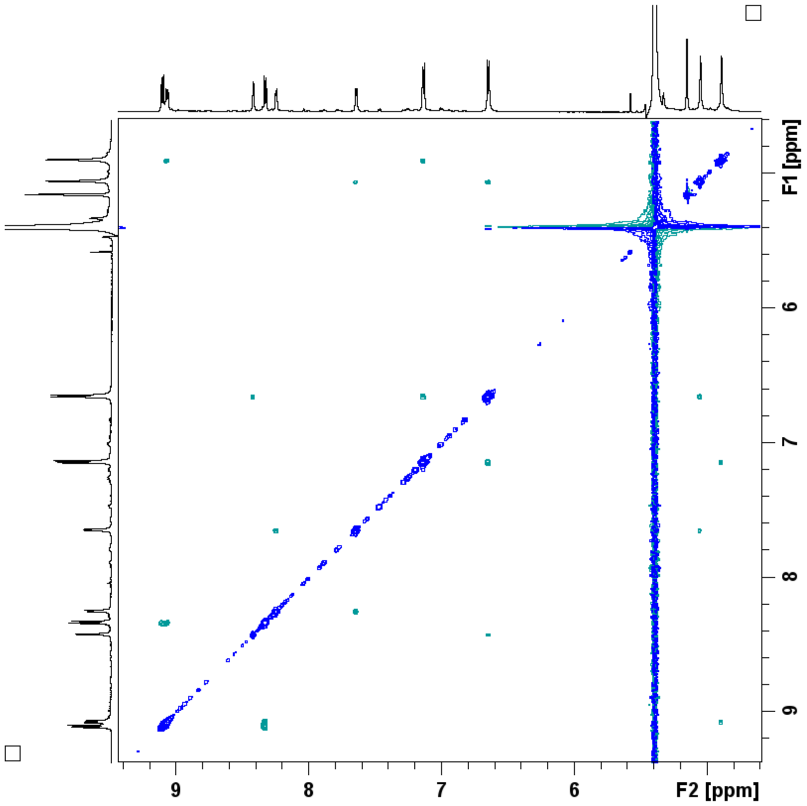

Figure S56. The ^{1}H-^{1}H ROESY spectrum of **6-2Ag$_2^{OTf}$** ([D_4]methanol, 240 K, 600 MHz).

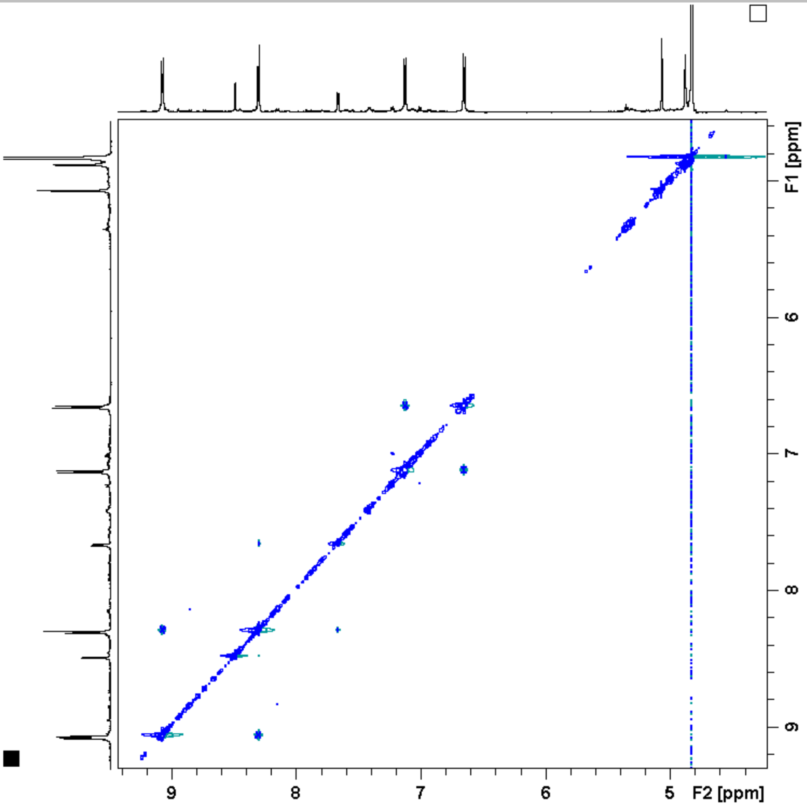

Figure S57. The ^{1}H-^{1}H NOESY spectrum of **6-2Ag$_2$^{OTf}** ([D$_4$]methanol, 300 K, 600 MHz).

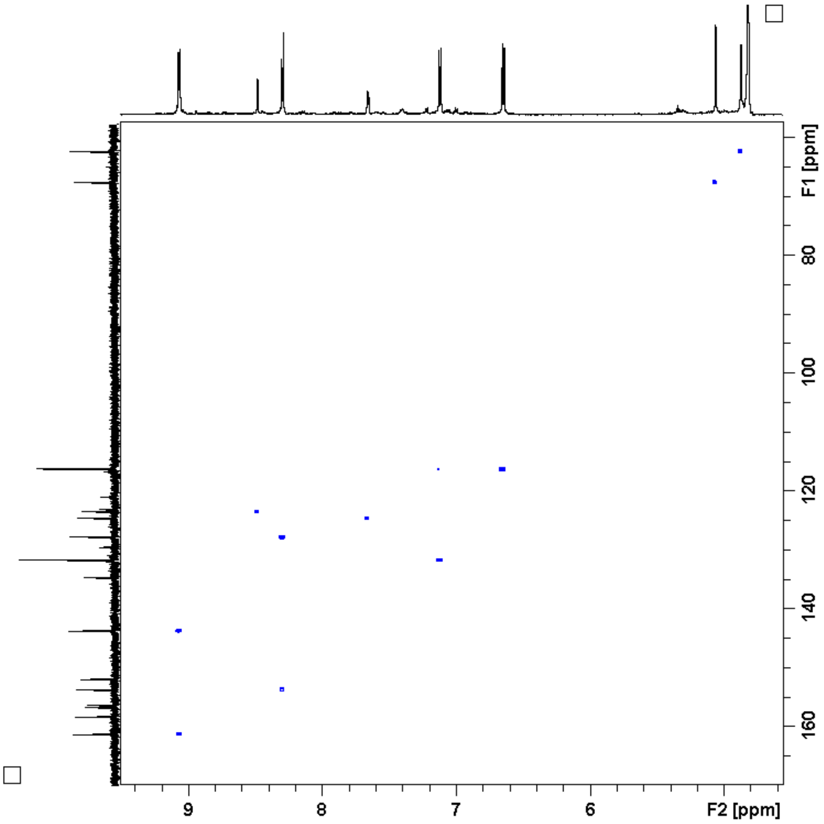

Figure S58. The ^{1}H-^{13}C HSQC spectrum of **6-2Ag$_2$^{OTf}** ([D$_4$]methanol, 300 K, 600 MHz).

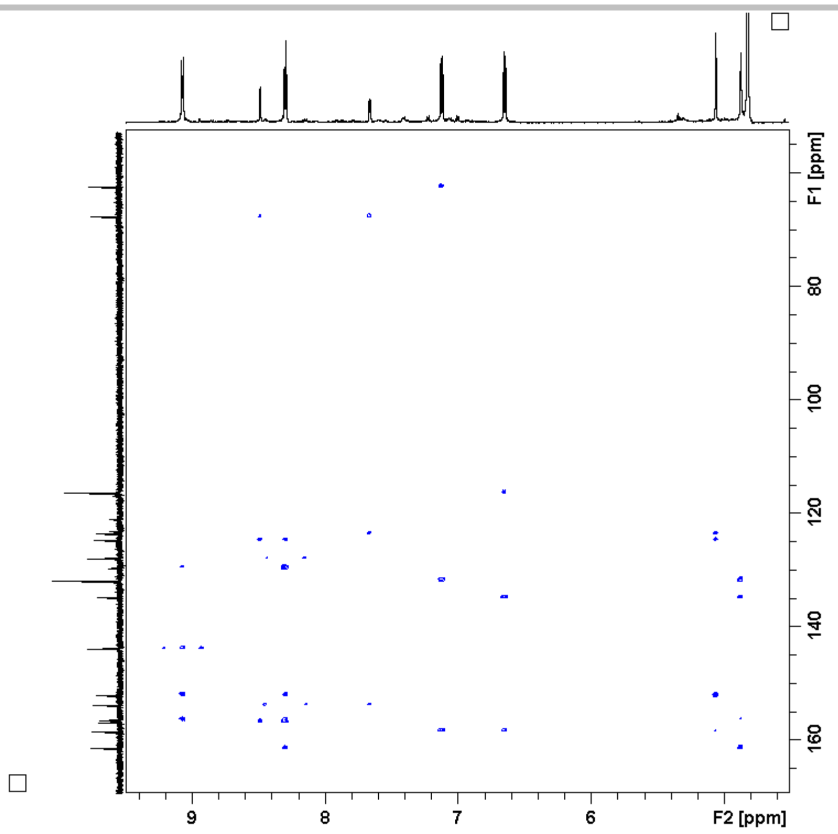

Figure S59. The ^{1}H-^{13}C HMBC spectrum of **6-2Ag$_2$^{OTf}** ([D$_4$]methanol, 300 K, 600 MHz).

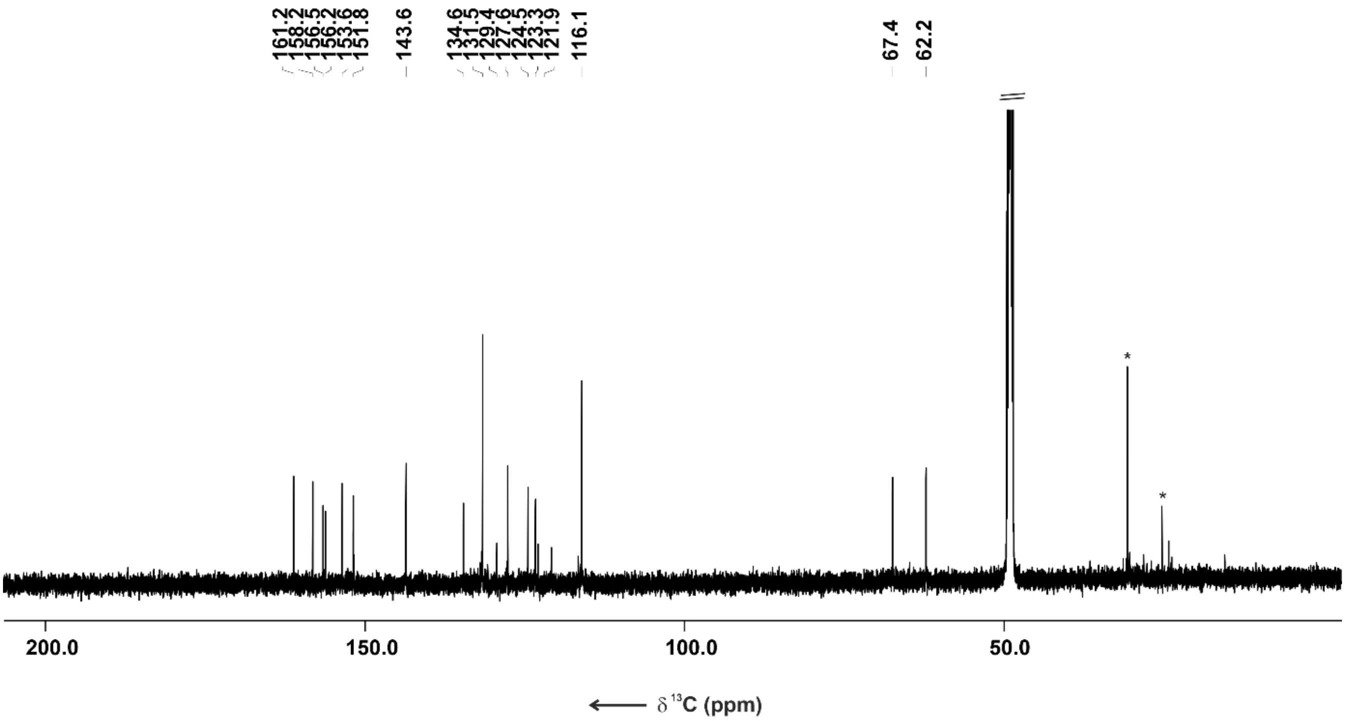

Figure S60. The ^{13}C NMR spectrum of [2]catenane **6-2Ag$_2$^{OTf}** ([D$_4$]methanol, 300 K, 151 MHz).

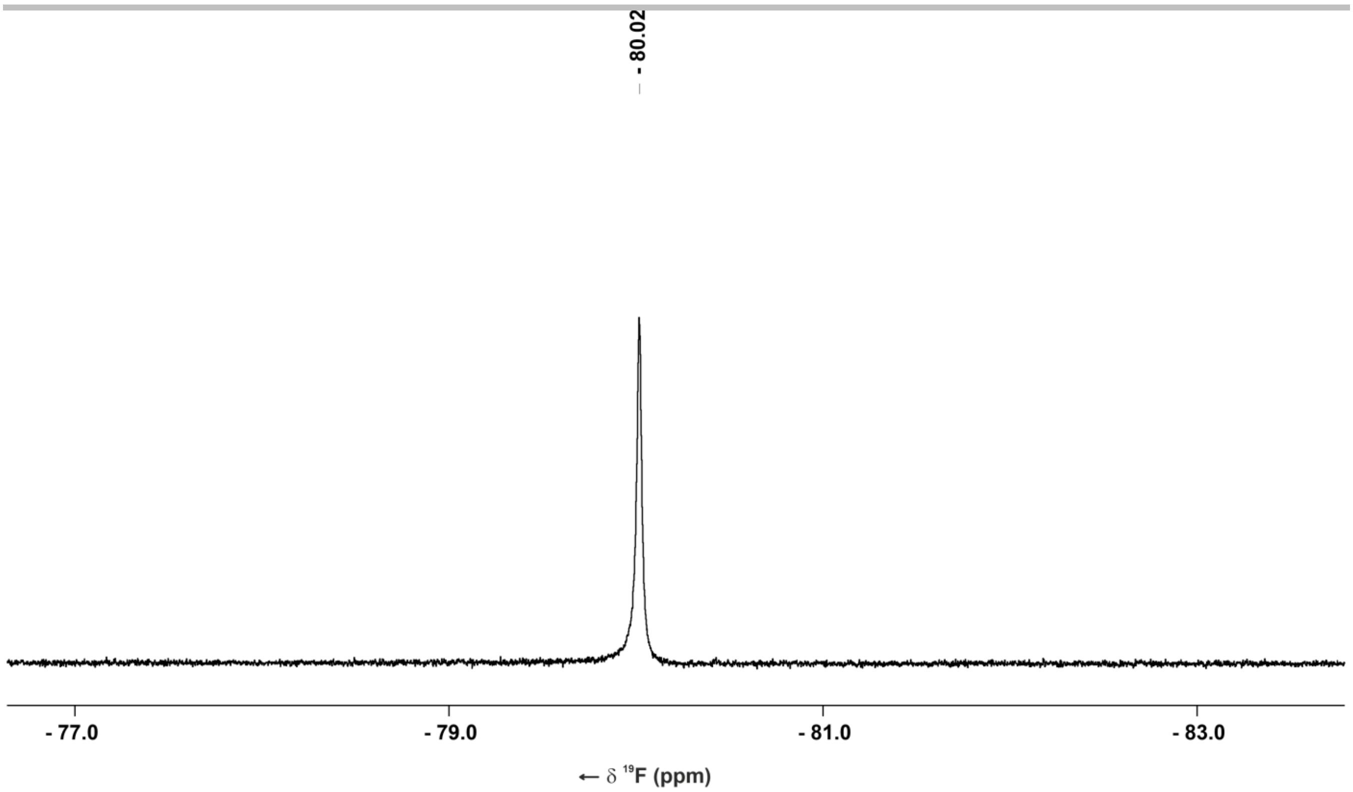

Figure S61. The ^{19}F NMR spectrum of **6-2Ag$_2$^{OTf}** ([D$_4$]methanol, 300 K, 565 MHz).

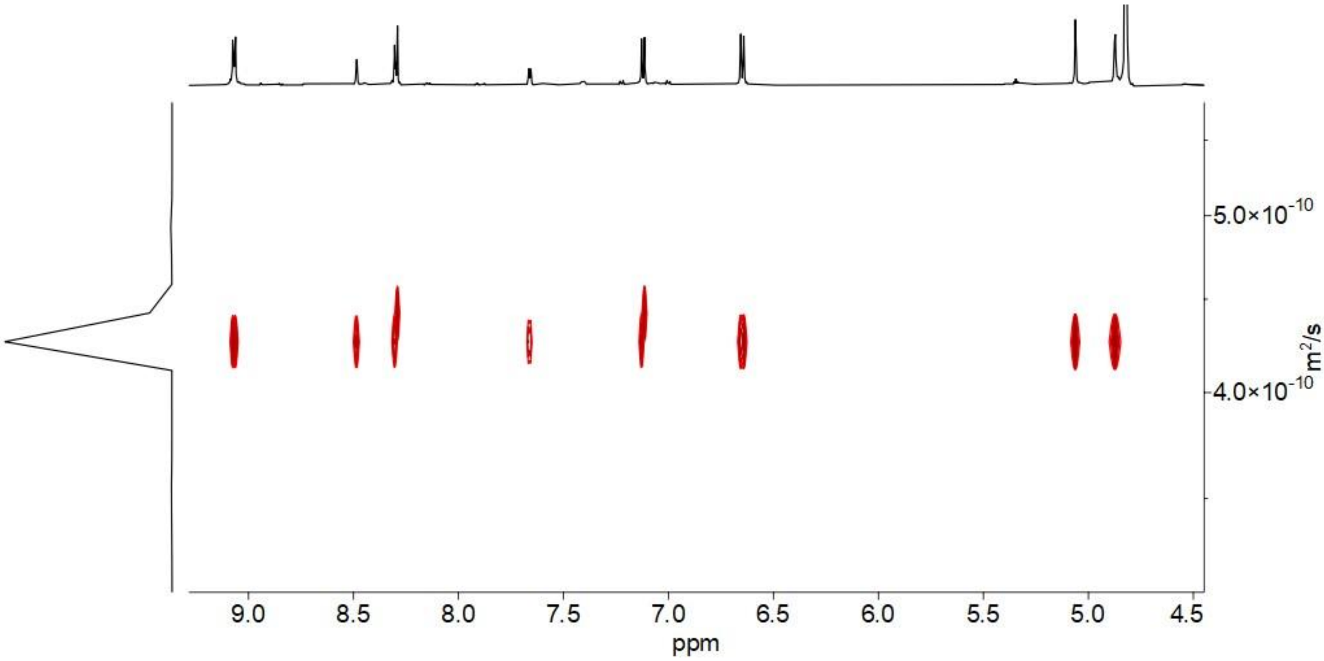

Figure S62. The DOSY NMR spectrum of [2]catenane **6-2Ag$_2$^{OTf}** ([D$_4$]methanol, 300 K, 600 MHz).

NMR spectra of [2]catenane 6-2Ag$_2^{OAc}$ (obtained with silver(I) acetate).

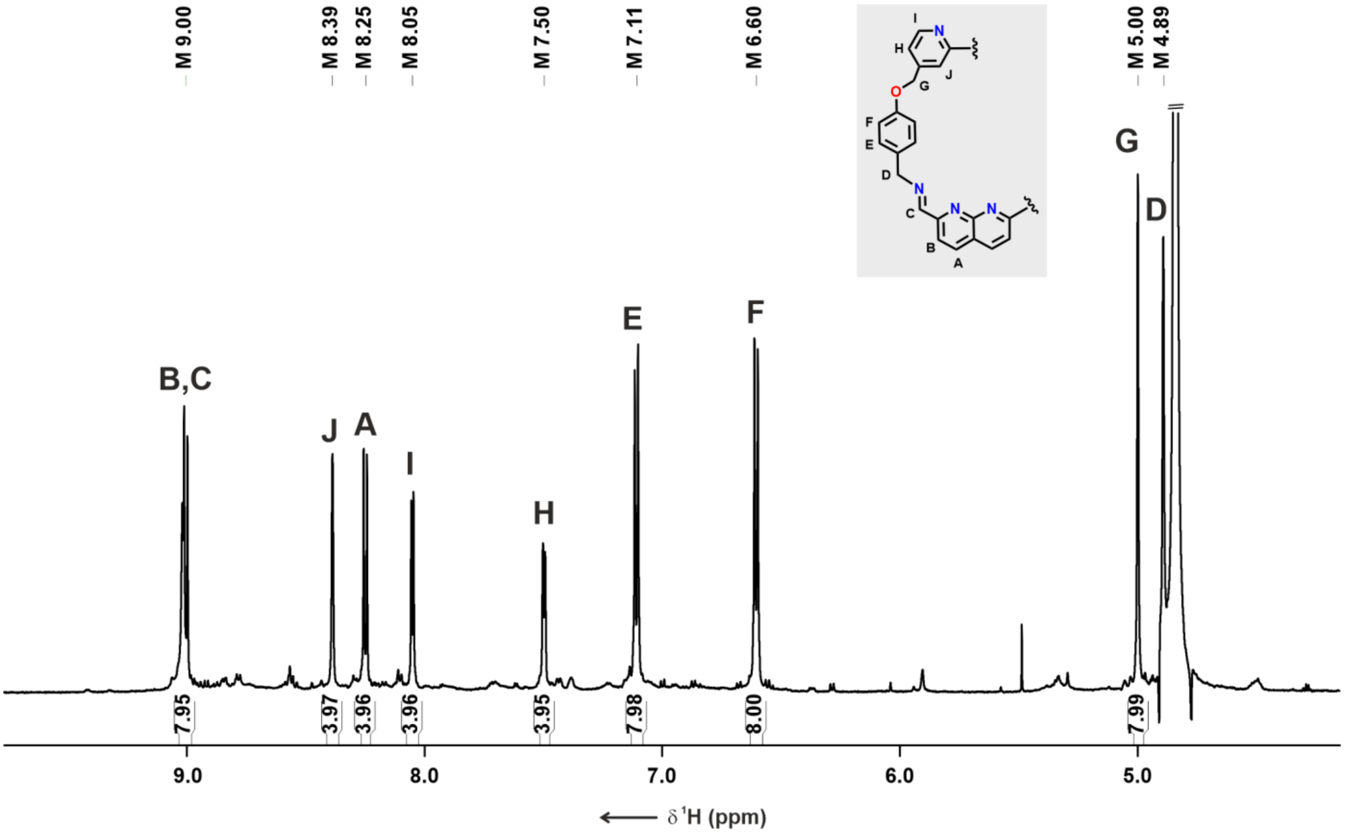

Figure S63. The ^{1}H NMR spectrum of **6-2Ag$_2^{OAc}$** ([D$_4$]methanol, 300 K, 600 MHz).

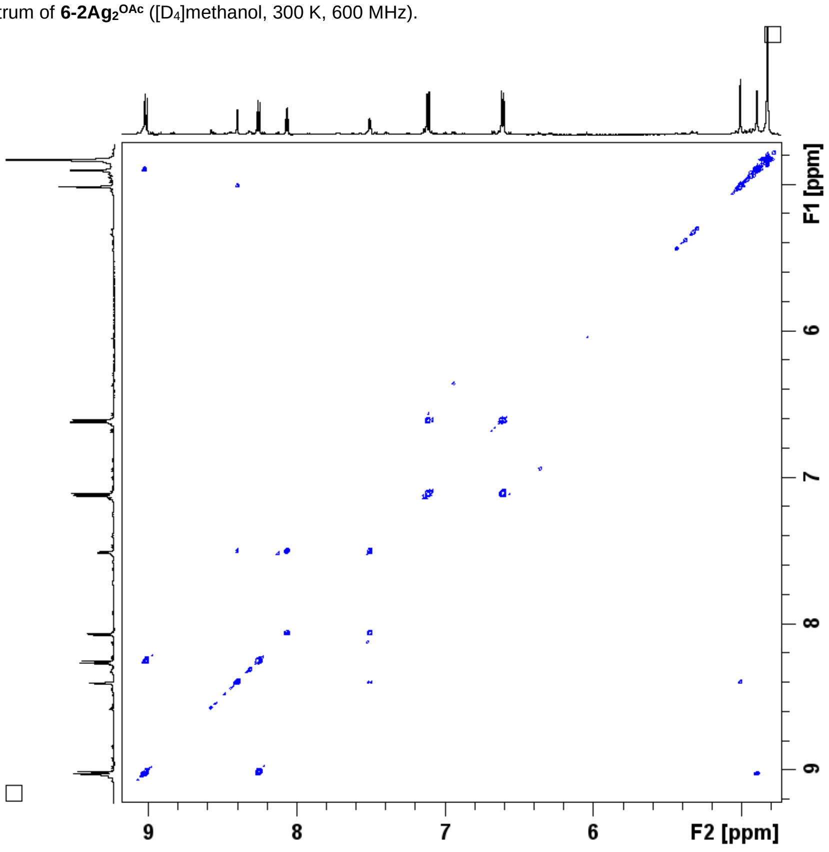

Figure S64. The ^{1}H-^{1}H COSY spectrum of **6-2Ag$_2^{OAc}$** ([D$_4$]methanol, 300 K, 600 MHz).

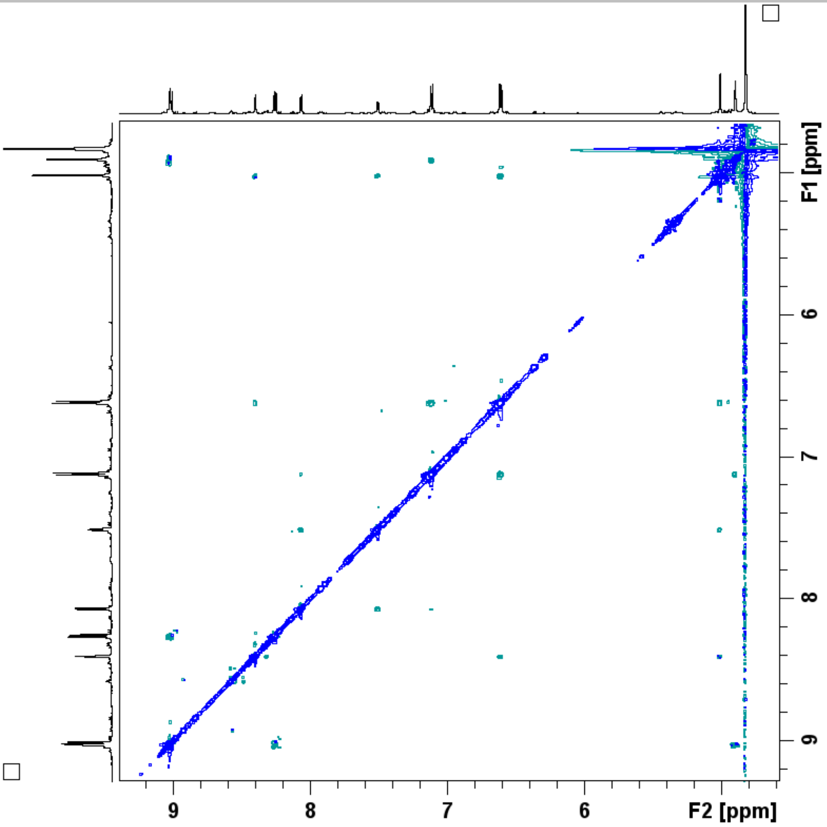

Figure S65. The ^{1}H-^{1}H ROESY spectrum of **6-2Ag$_2$^{OAc}** ([D$_4$]methanol, 300 K, 600 MHz).

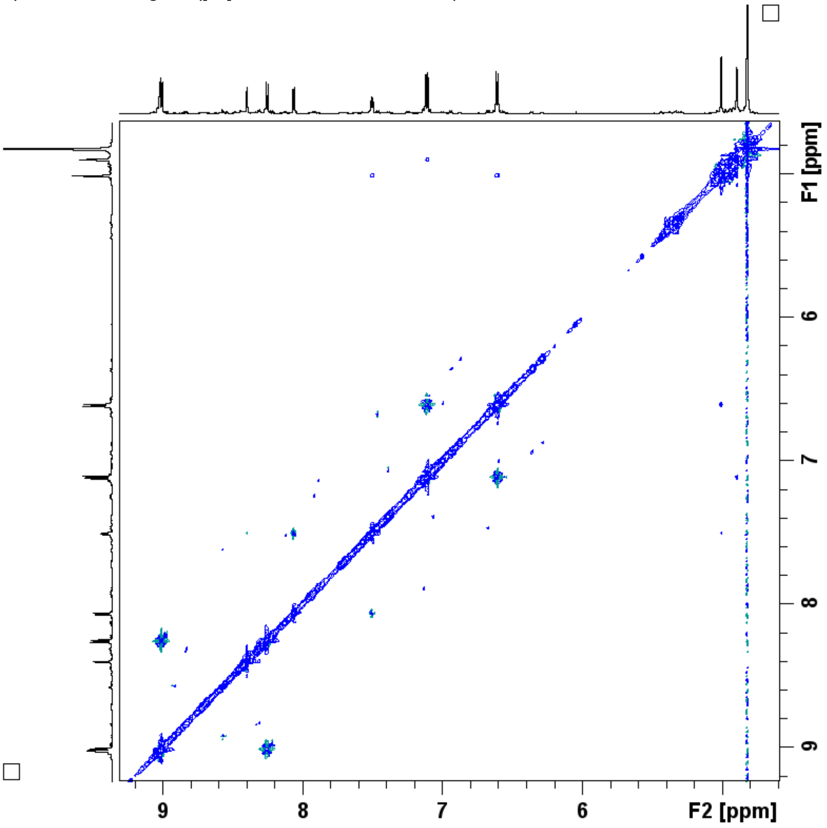

Figure S66. The ^{1}H-^{1}H NOESY spectrum of **6-2Ag$_2$^{OAc}** ([D$_4$]methanol, 300 K, 600 MHz).

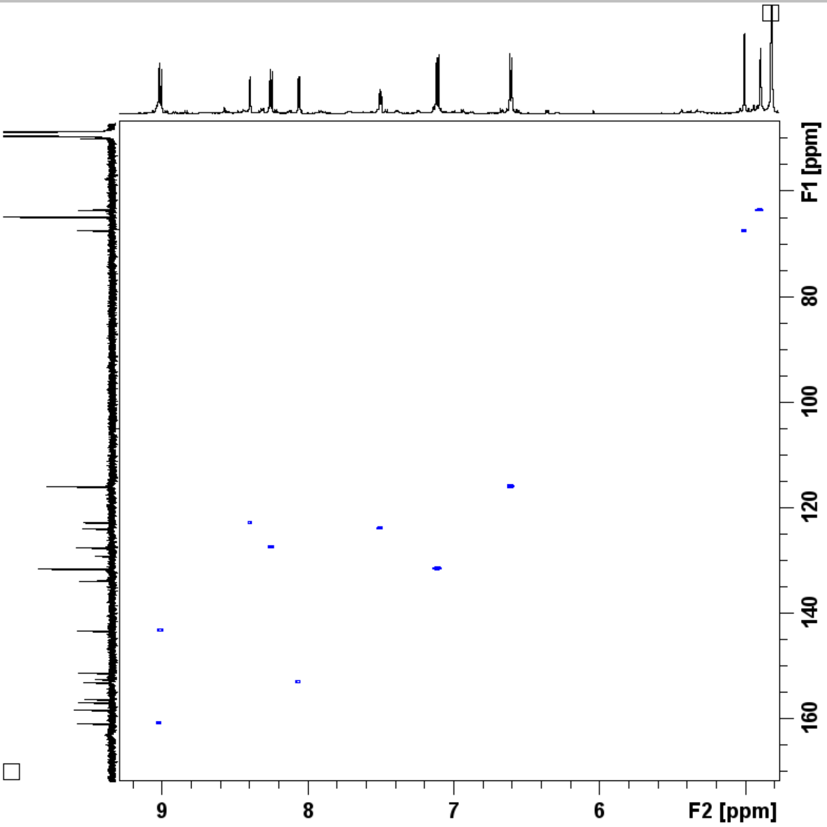

Figure S67. The ^{1}H-^{13}C HSQC spectrum of **6-2Ag$_2^{OAc}$** ([D$_4$]methanol, 300 K, 600 MHz).

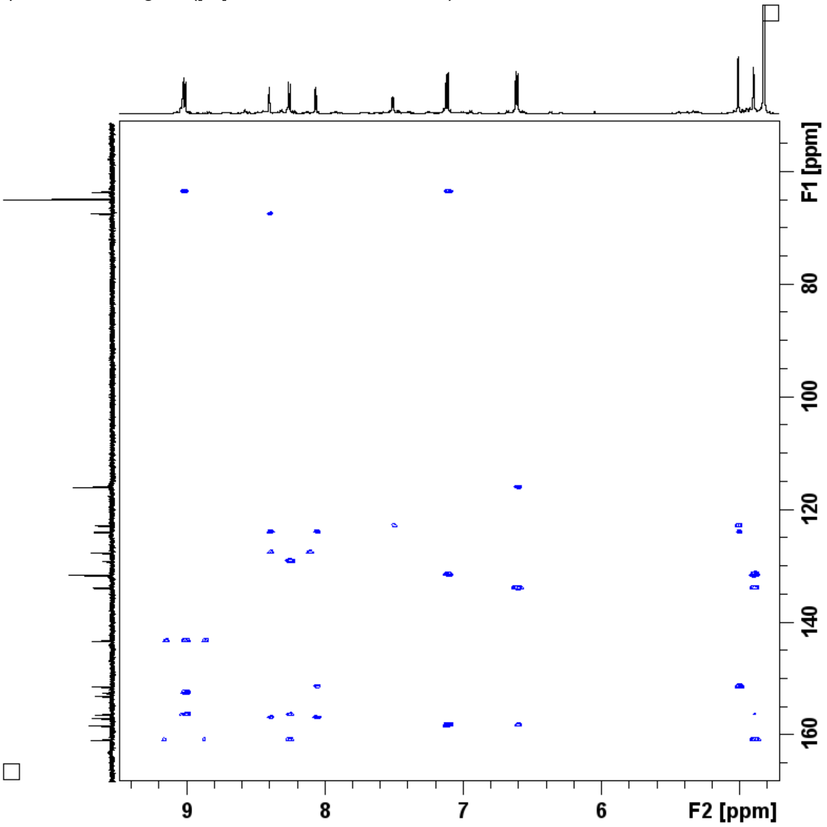

Figure S68. The ^{1}H-^{13}C HMBC spectrum of **6-2Ag$_2^{OAc}$** ([D$_4$]methanol, 300 K, 600 MHz).

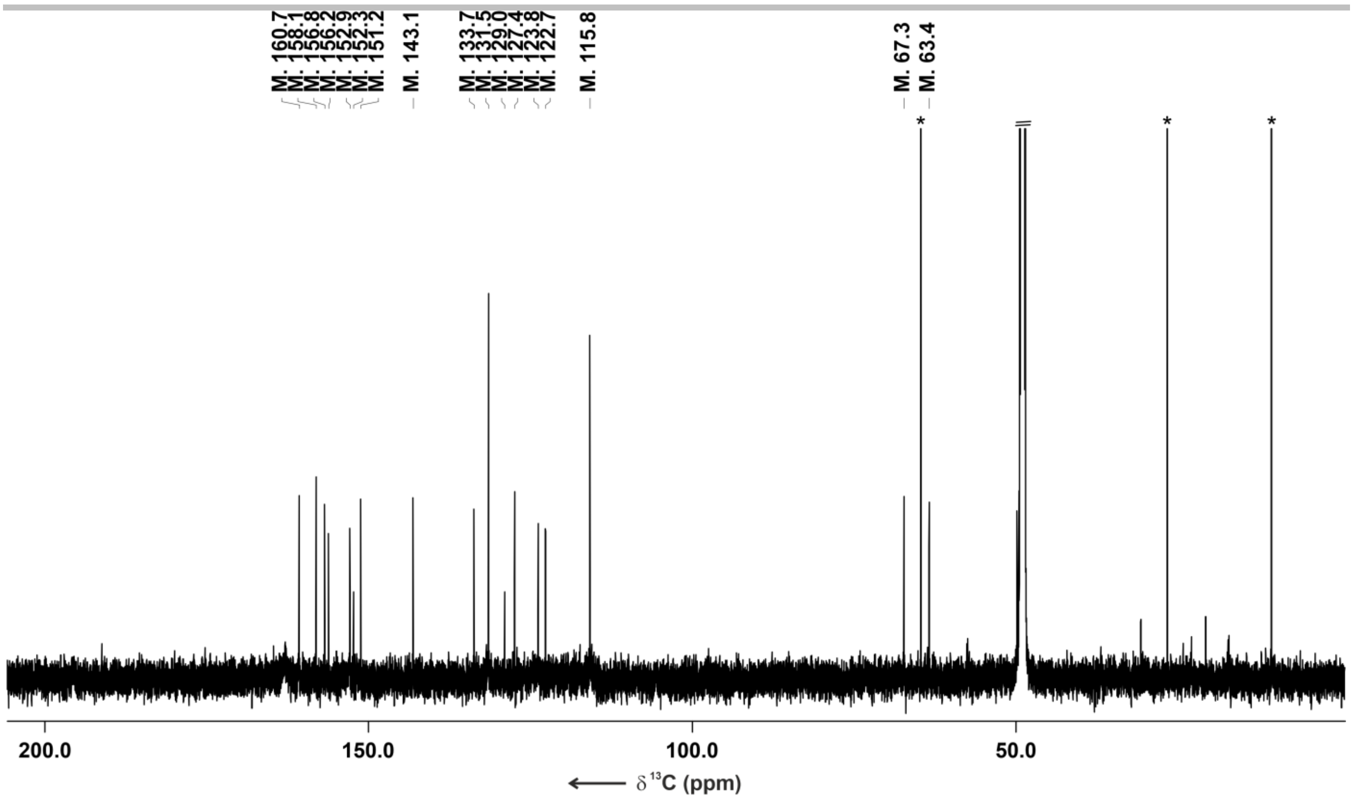

Figure S69. The ^{13}C NMR spectrum of **6-2Ag$_2^{OAc}$** ([D$_4$]methanol, 300 K, 151 MHz). The signals corresponding to impurities were marked with asterisks.

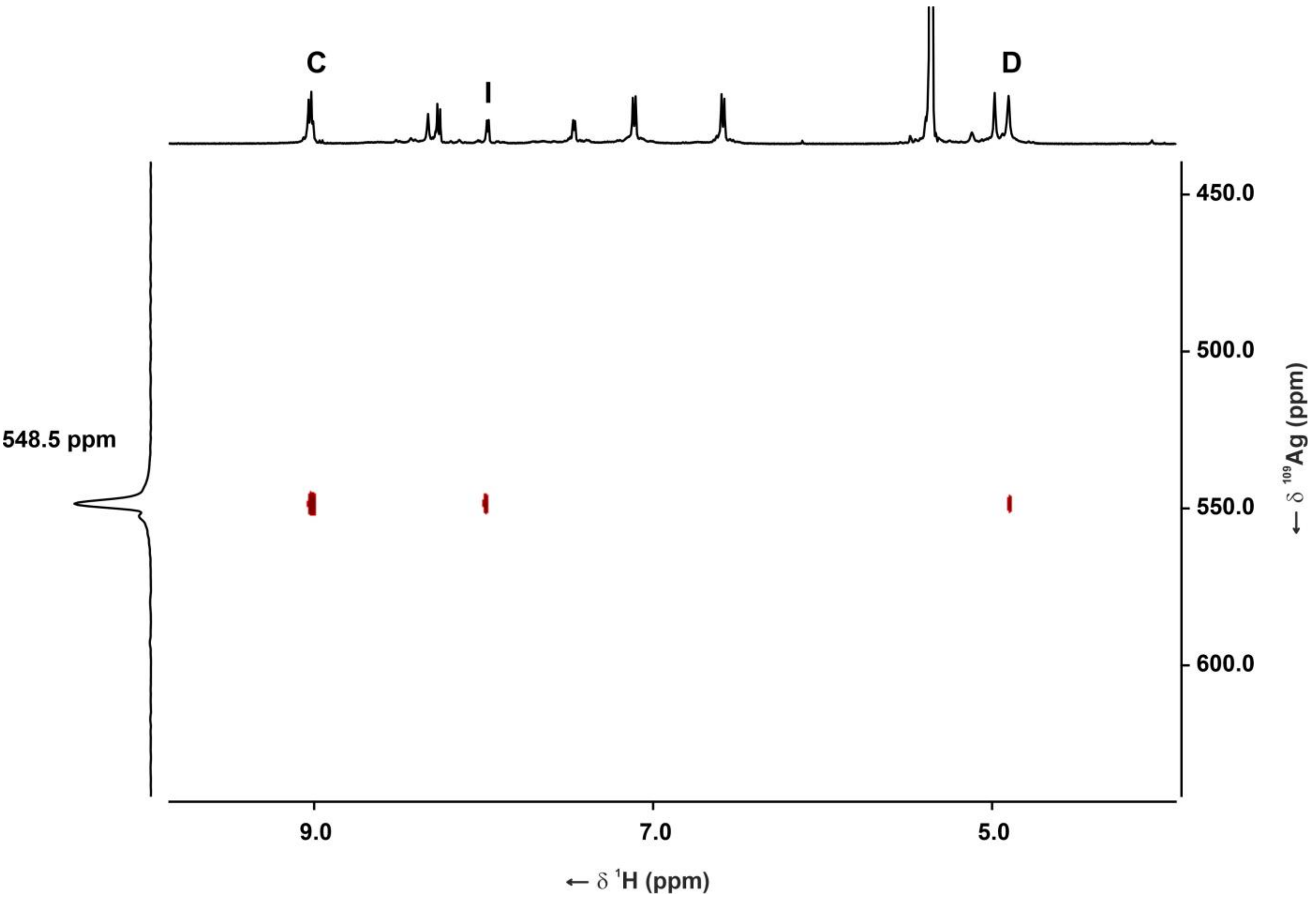

Figure S70. The ^{1}H-^{109}Ag HMBC spectrum of **6-2Ag$_2^{OAc}$** ([D$_4$]methanol, 250 K, 500 MHz).

NMR spectra of trefoil knot 7-3Ag2

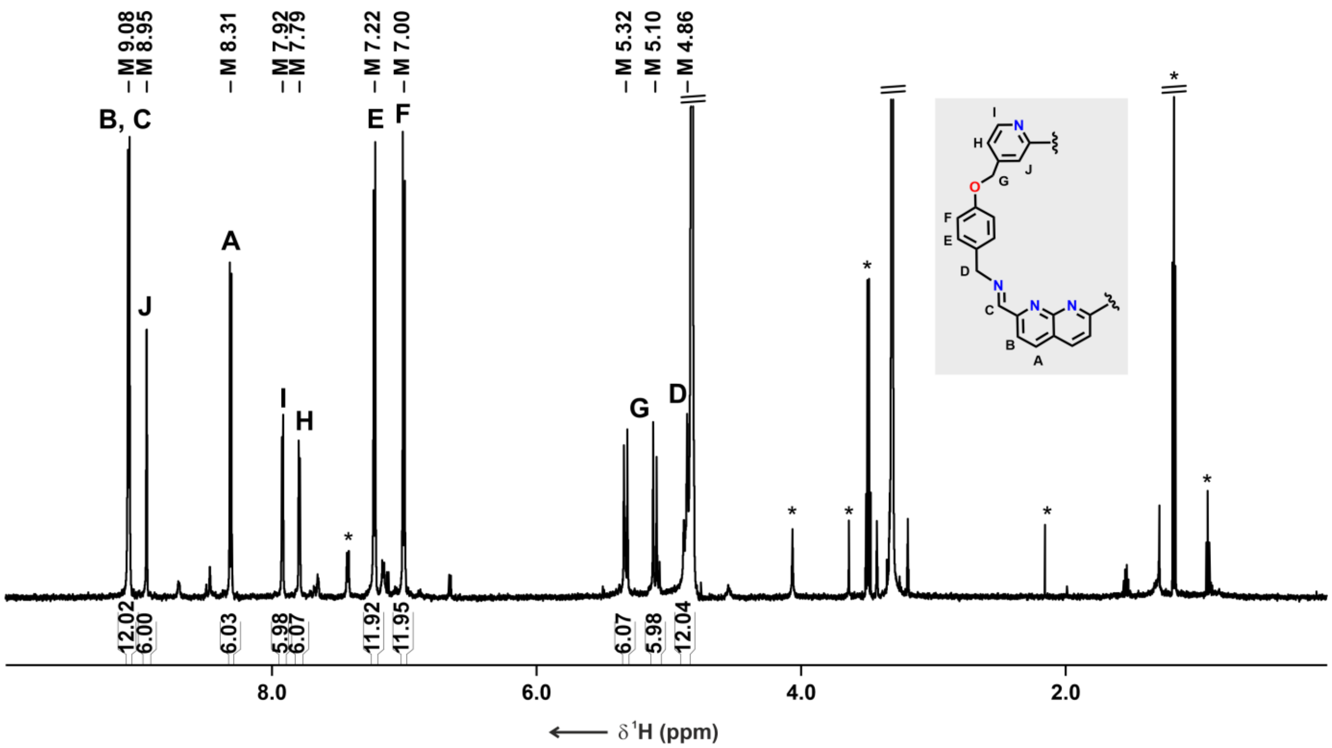

Figure S71. The ^{1}H NMR spectrum of **7-3Ag$_2$** ([D$_4$]methanol, 300 K, 600 MHz). The signals corresponding to impurities were marked with asterisks.

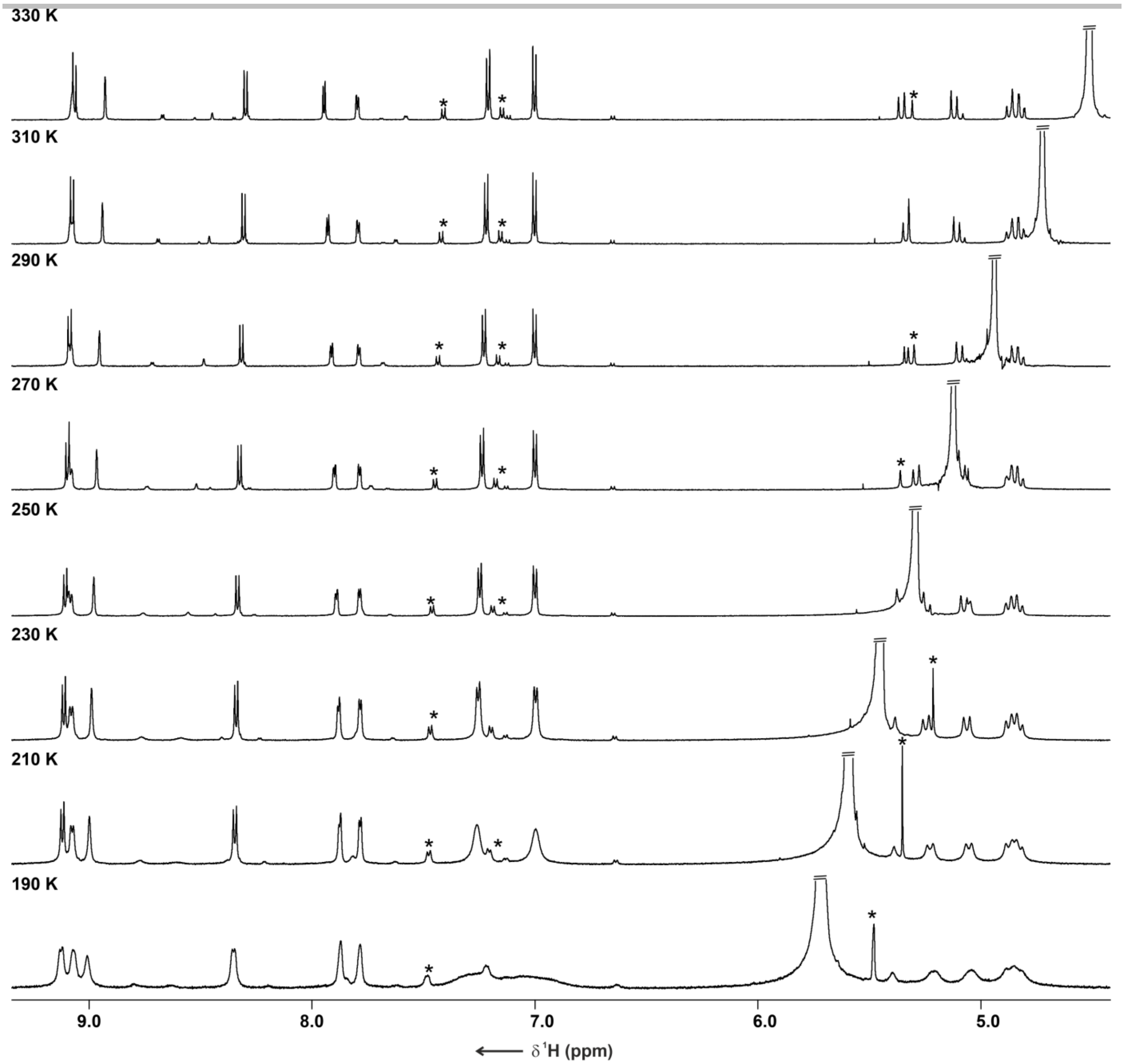

Figure S72. The ^{1}H NMR spectra of **7-3Ag$_2$** recorded in the 330 K – 190 K temperature range ([D$_4$]methanol, 600 MHz). The signals corresponding to impurities were marked with asterisks.

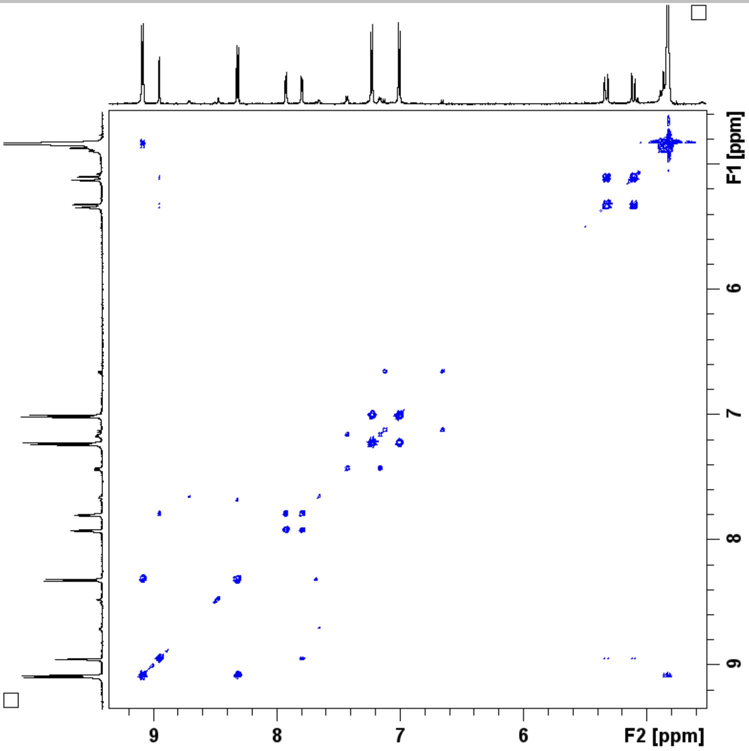

Figure S73. The ^{1}H-^{1}H COSY spectrum of **7-3Ag₂** ([D₄]methanol, 300 K, 600 MHz).

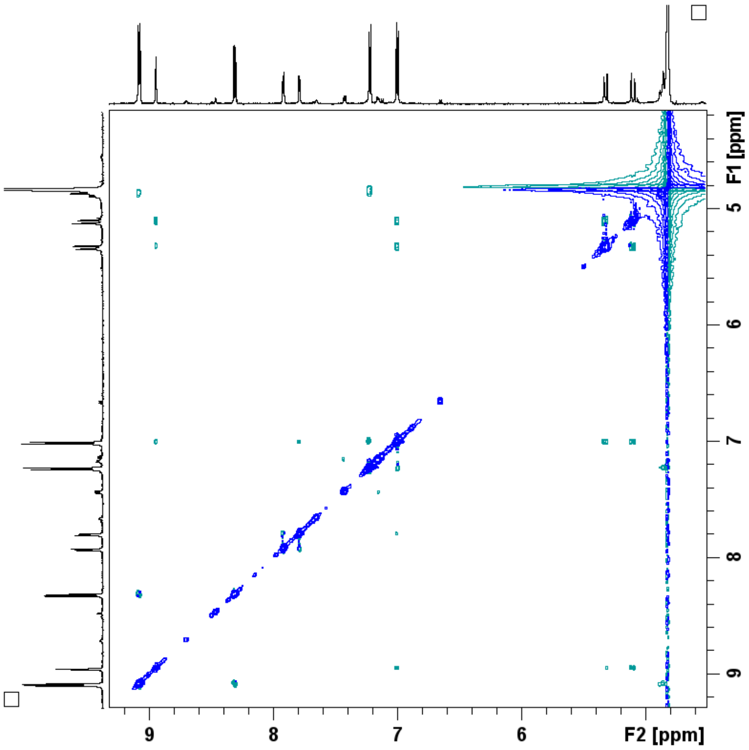

Figure S74. The ^{1}H-^{1}H ROESY spectrum of **7-3Ag$_2$** ([D$_4$]methanol, 300 K, 600 MHz).

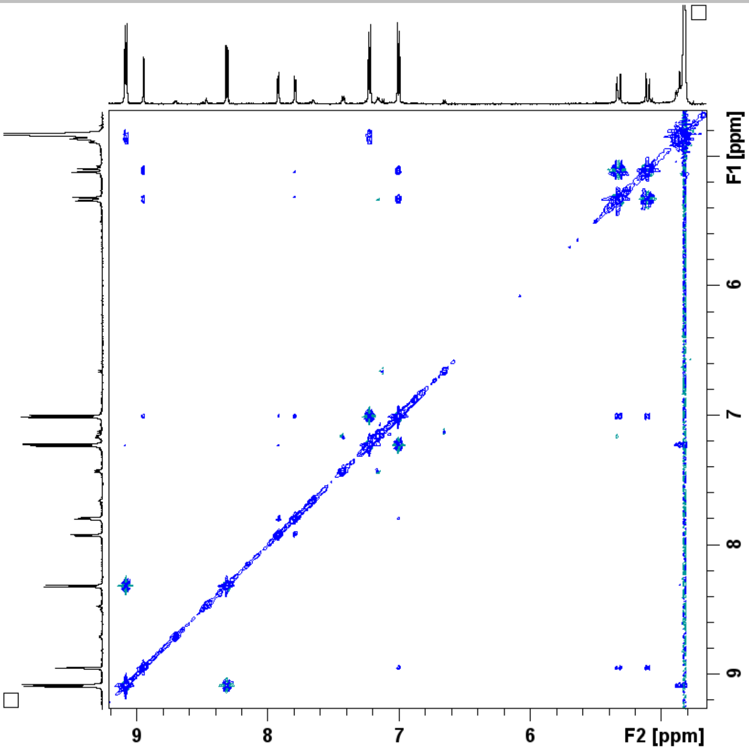

Figure S75. The ^{1}H-^{1}H NOESY spectrum of **7-3Ag₂** ([D₄]methanol, 300 K, 600 MHz).

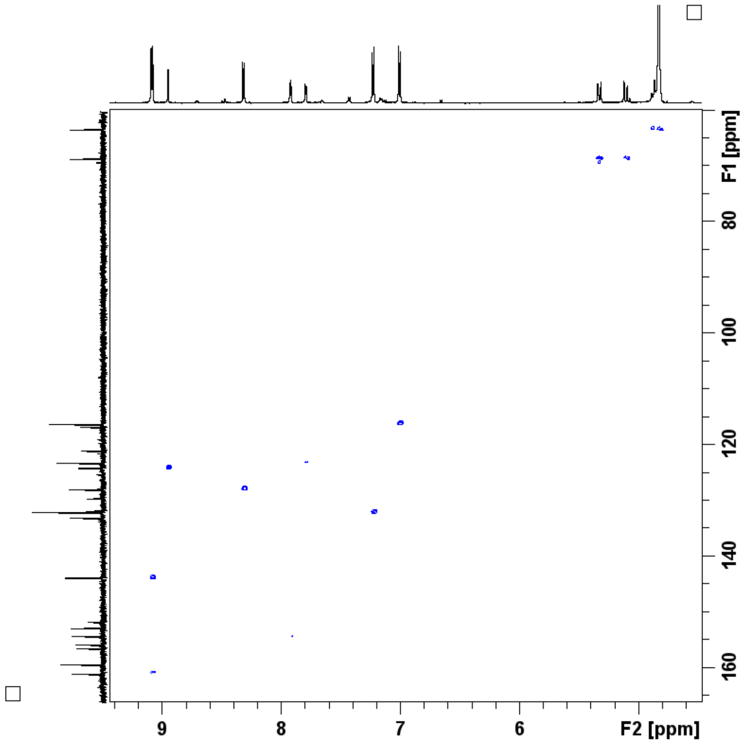

Figure S76. The ^{1}H-^{13}C HMQC spectrum of **7-3Ag₂** ([D₄]methanol, 300 K, 600 MHz).

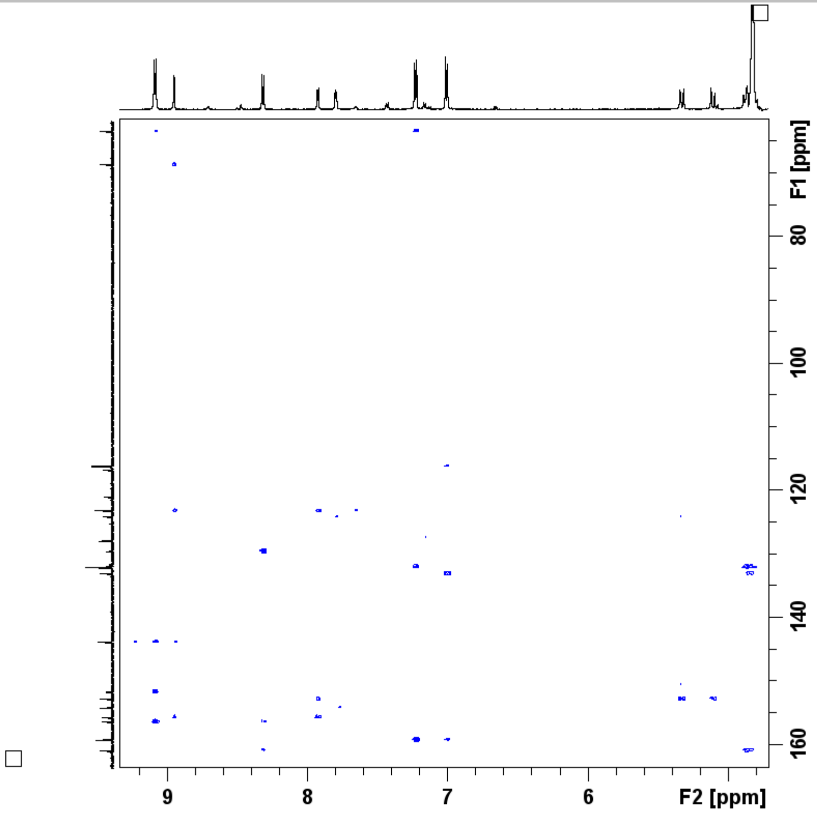

Figure S77. The ^{1}H-^{13}C HMBC spectrum of **7-3Ag$_2$** ([D$_4$]methanol, 300 K, 600 MHz).

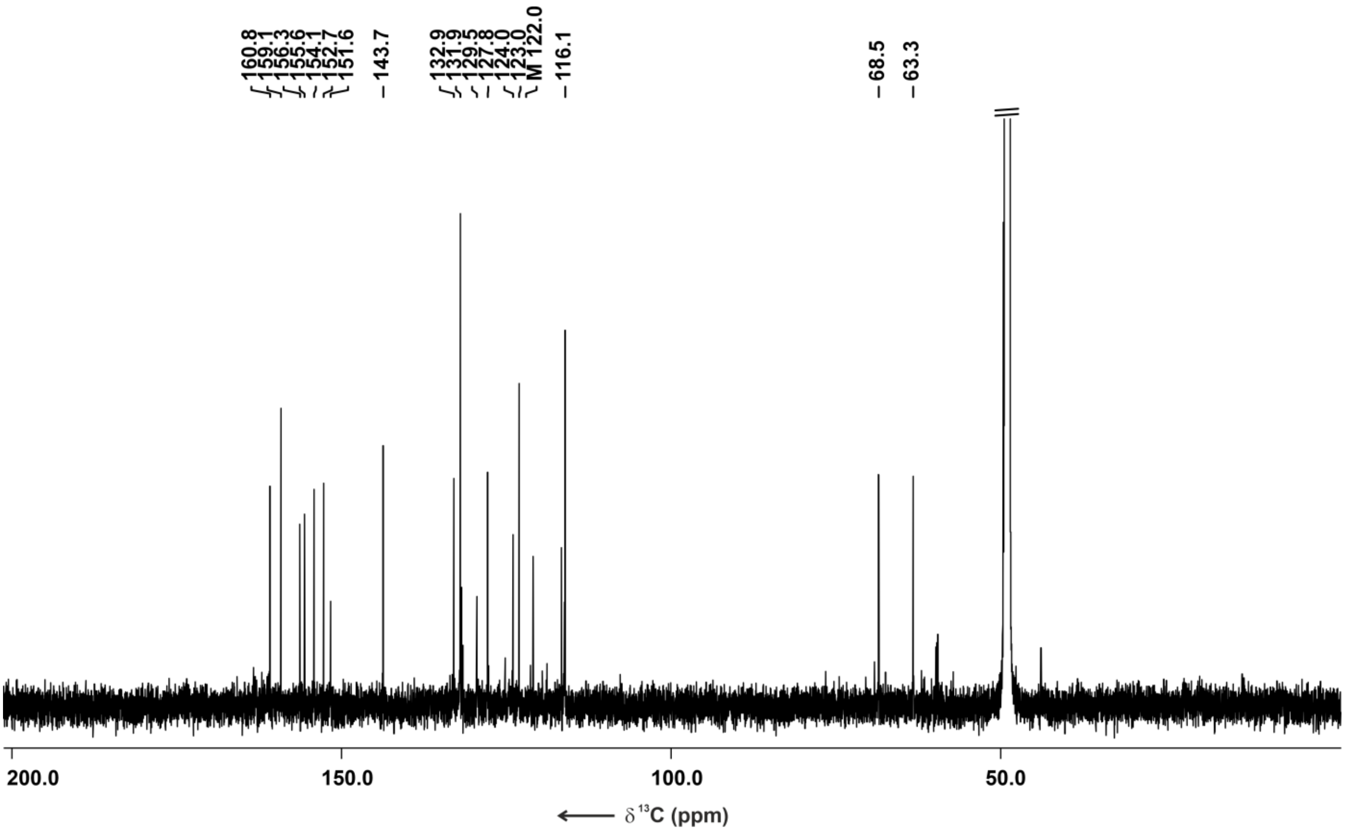

Figure S78. The ^{13}C NMR spectrum of **7-3Ag$_2$** ([D$_4$]methanol, 300 K, 151 MHz).

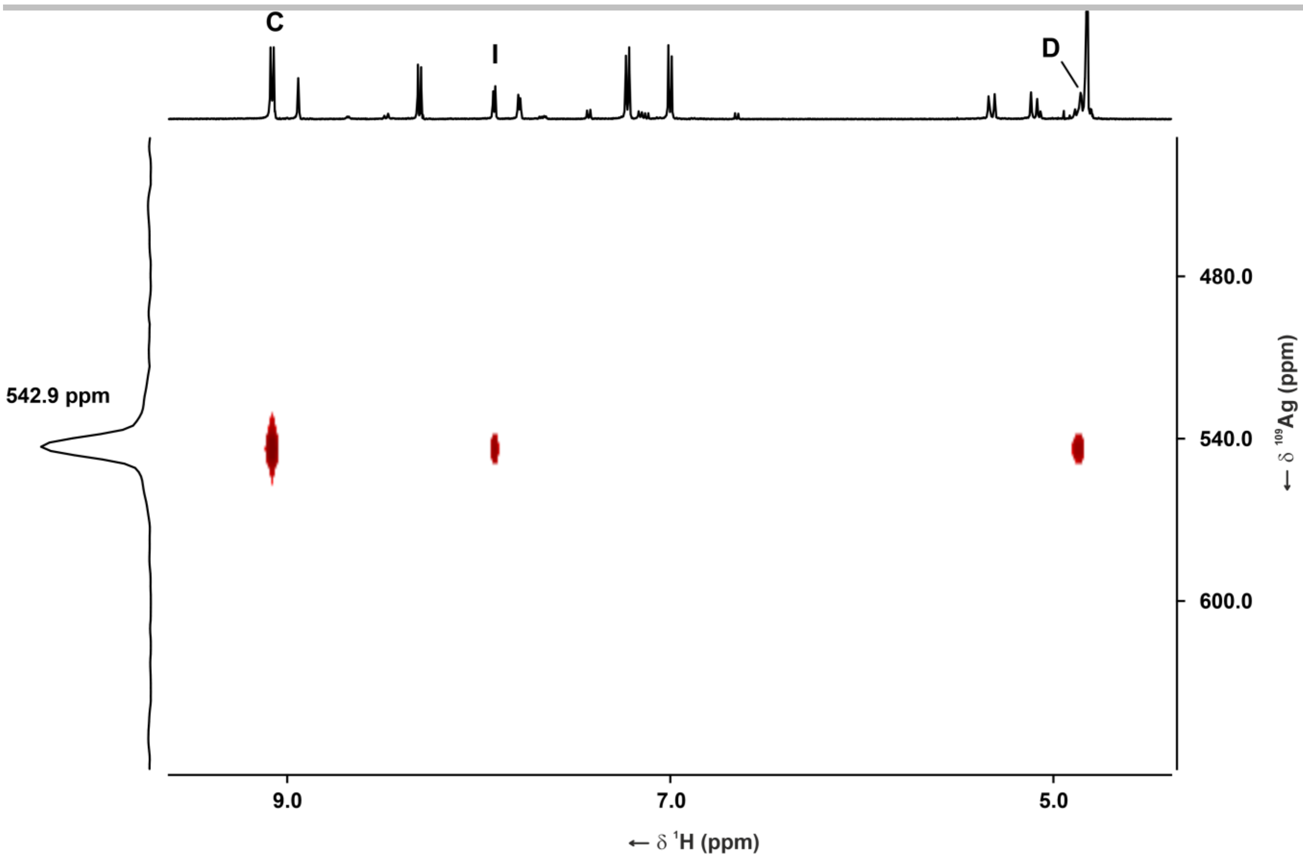

Figure S79. The ^{1}H-^{109}Ag HMBC spectrum of **7-3Ag₂** ([D$_4$]methanol, 300 K, 500 MHz).

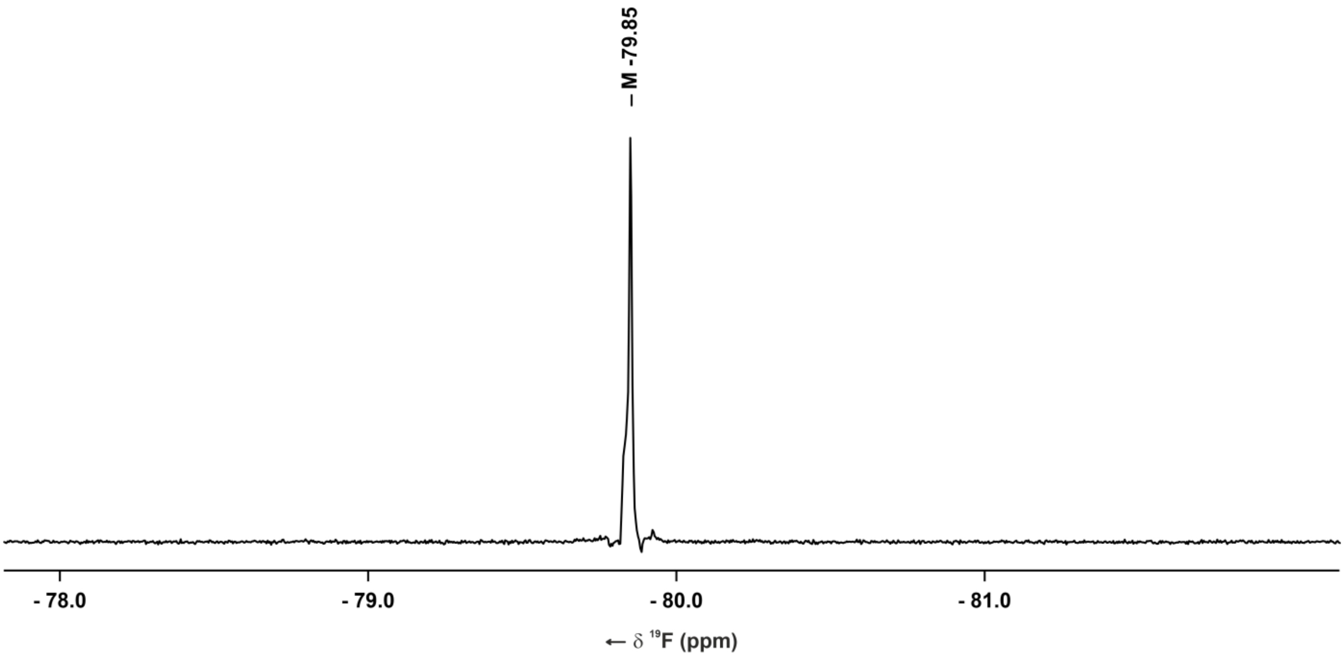

Figure S80. The ^{19}F NMR spectrum of **7-3Ag$_2$** ([D$_4$]methanol, 300 K, 565 MHz).

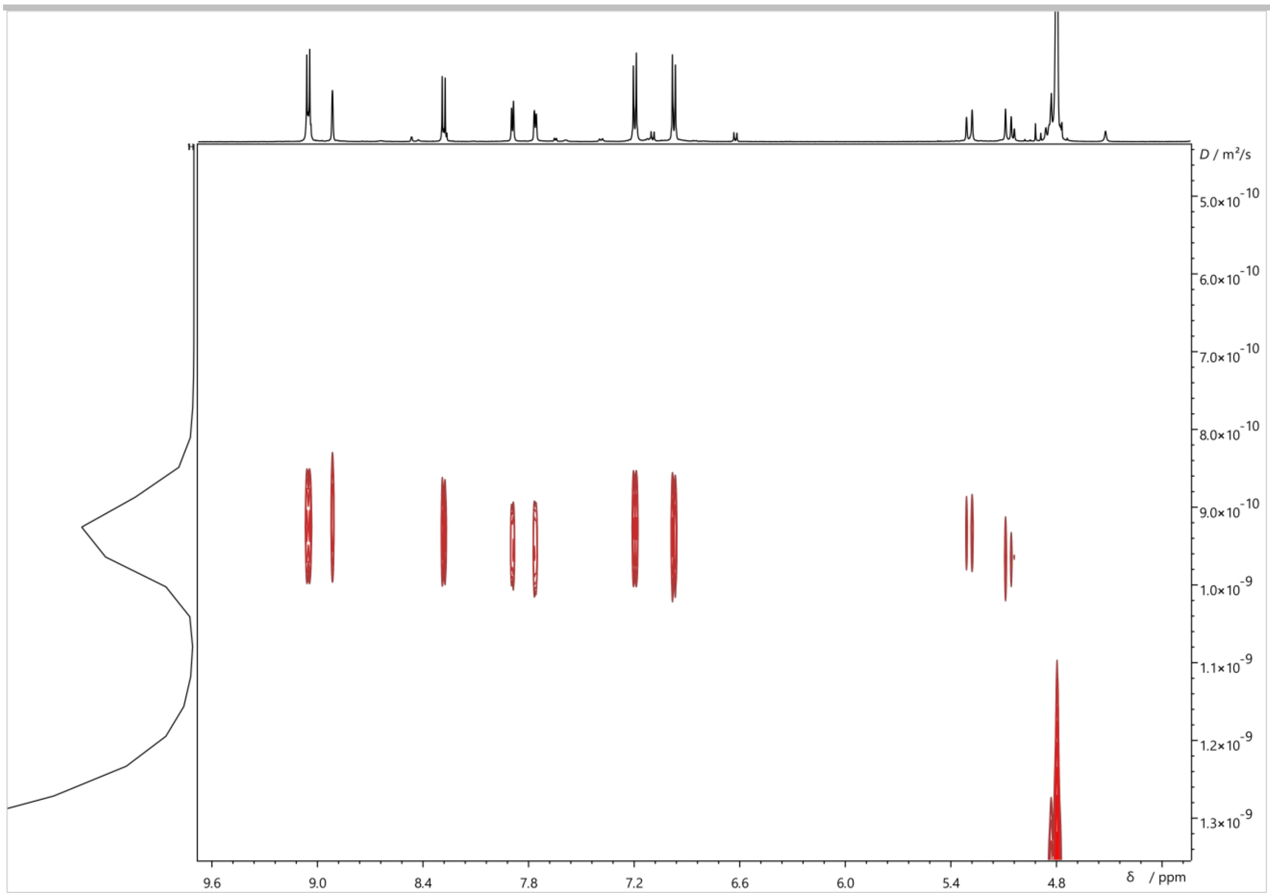

Figure S81. The DOSY spectrum of **7-3Ag$_2$** ([D$_4$]methanol, 300 K, 600 MHz).

NMR spectra of [2]catenane 9-2Ag₂

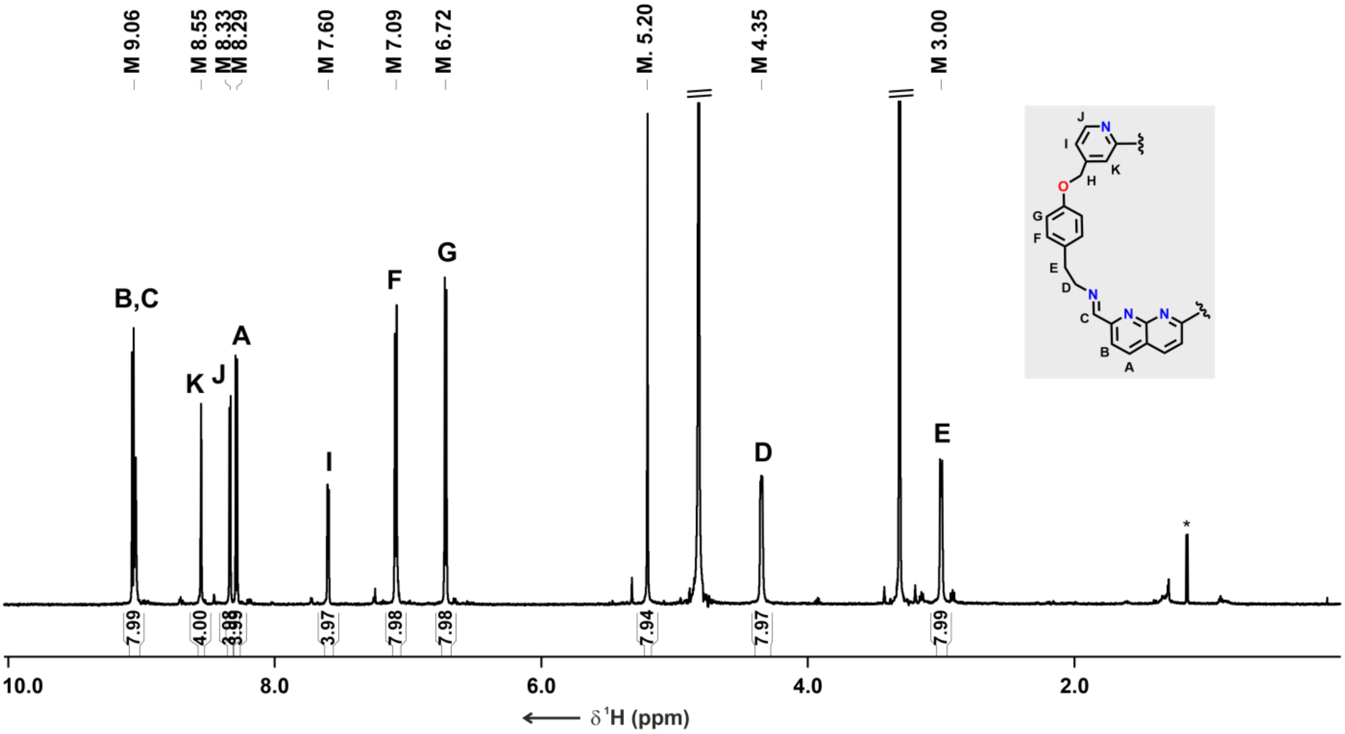

Figure S82. The ¹H NMR spectrum of **9-2Ag₂** ([D₄]methanol, 300 K, 500 MHz). The signal corresponding to impurities was marked with asterisks.

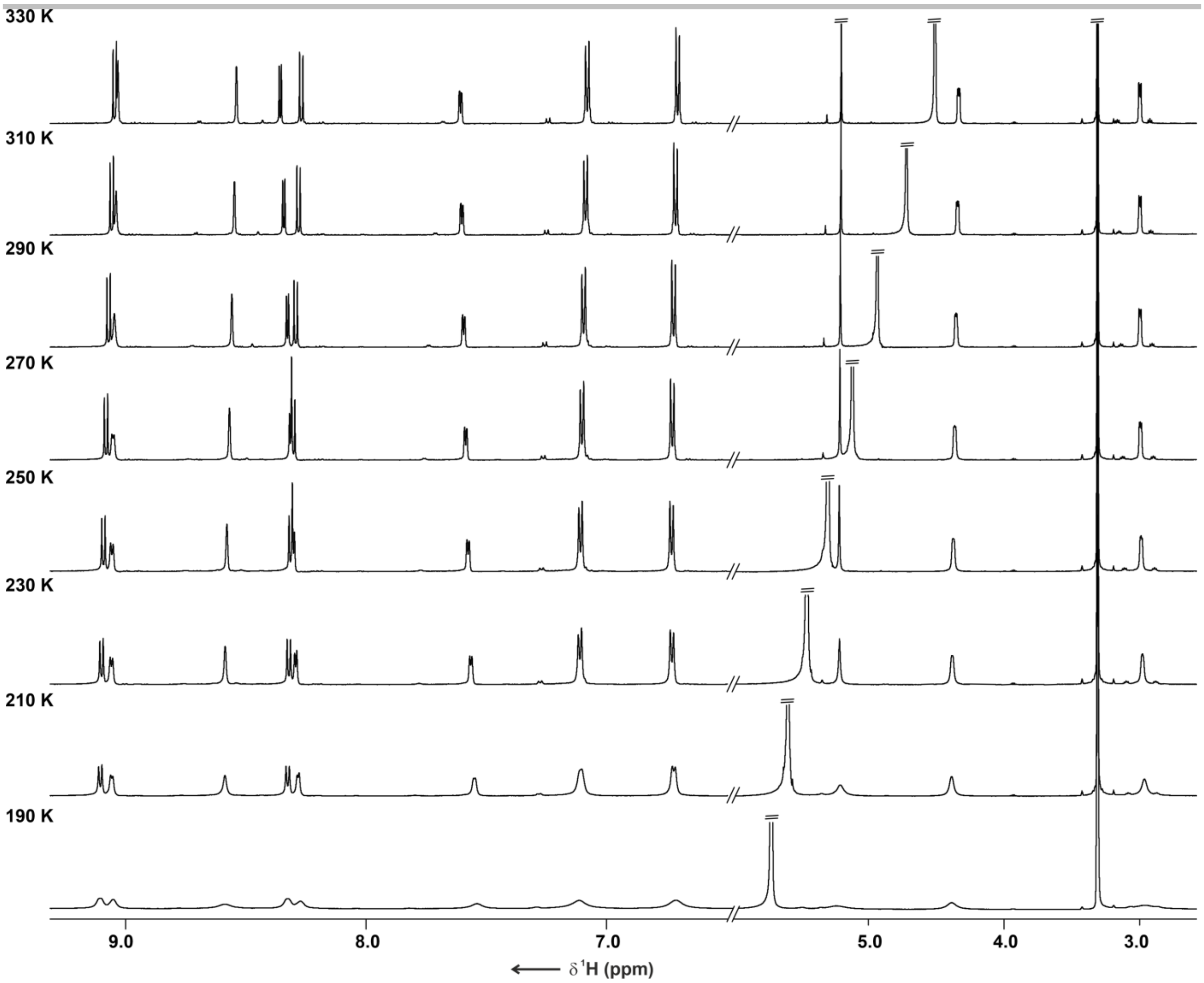

Figure S83. The ^{1}H NMR spectra of **9-2Ag$_2$** recorded in the 330 K – 190 K temperature range ([D$_4$]methanol, 600 MHz).

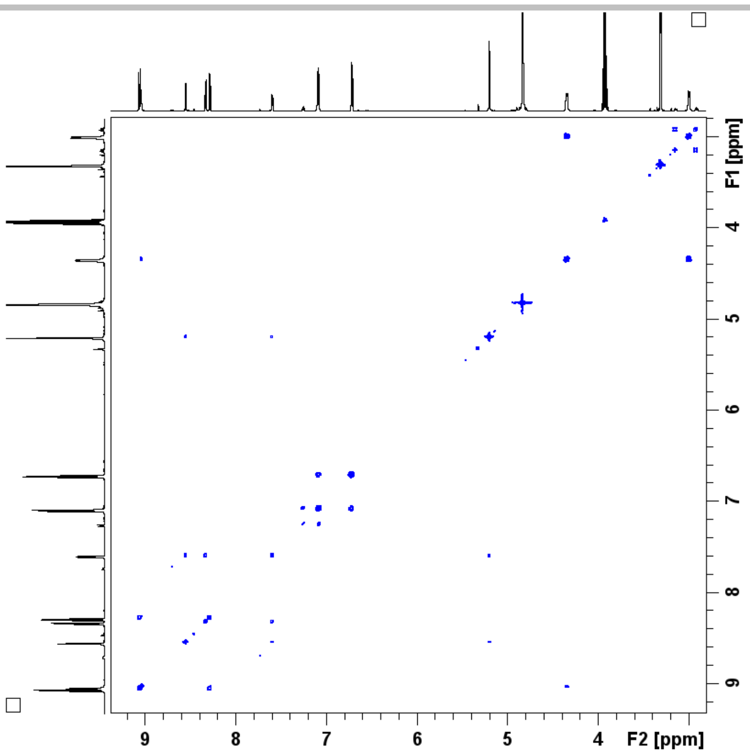

Figure S84. The ^{1}H-^{1}H COSY spectrum of **9-2Ag$_2$** ([D$_4$]methanol, 300 K, 600 MHz).

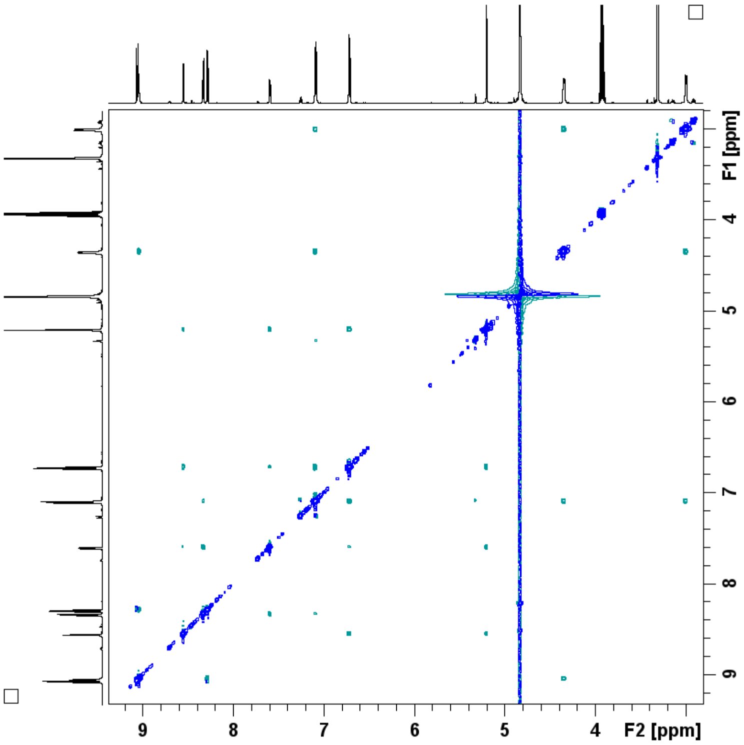

Figure S85. The ^{1}H-^{1}H ROESY spectrum of **9-2Ag$_2$** ([D$_4$]methanol, 300 K, 600 MHz).

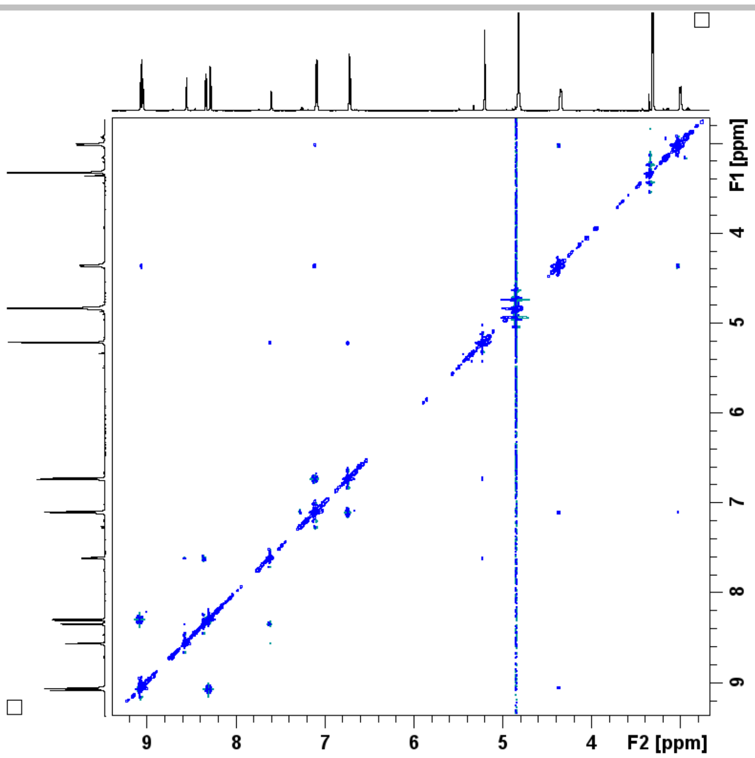

Figure S86. The ^{1}H-^{1}H NOESY spectrum of **9-2Ag₂** ([D₄]methanol, 300 K, 600 MHz).

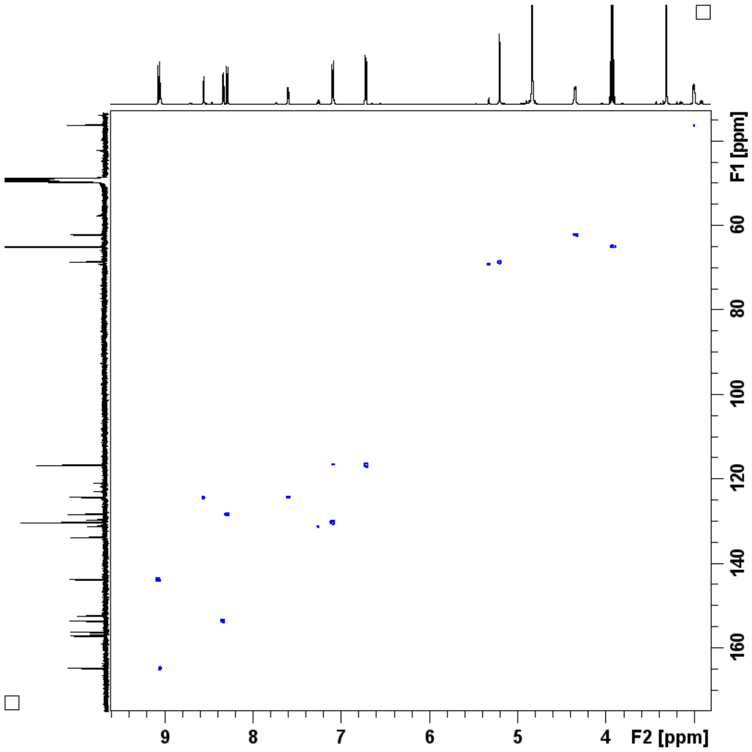

Figure S87. The ^{1}H-^{13}C HSQC spectrum of **9-2Ag$_2$** ([D$_4$]methanol, 300 K, 600 MHz).

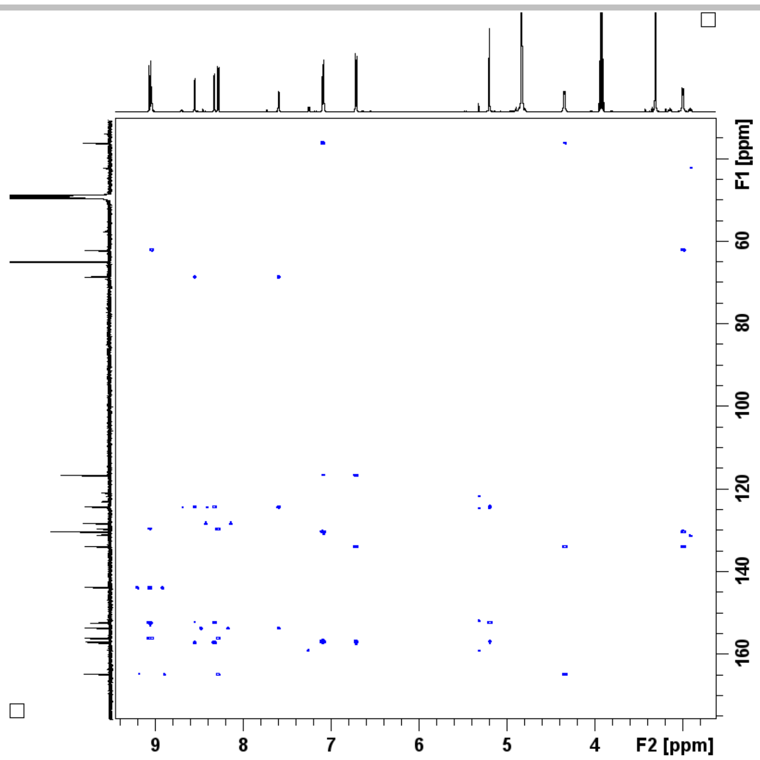

Figure S88. The ^{1}H-^{13}C HMBC spectrum of **9-2Ag₂** ([D₄]methanol, 300 K, 600 MHz).

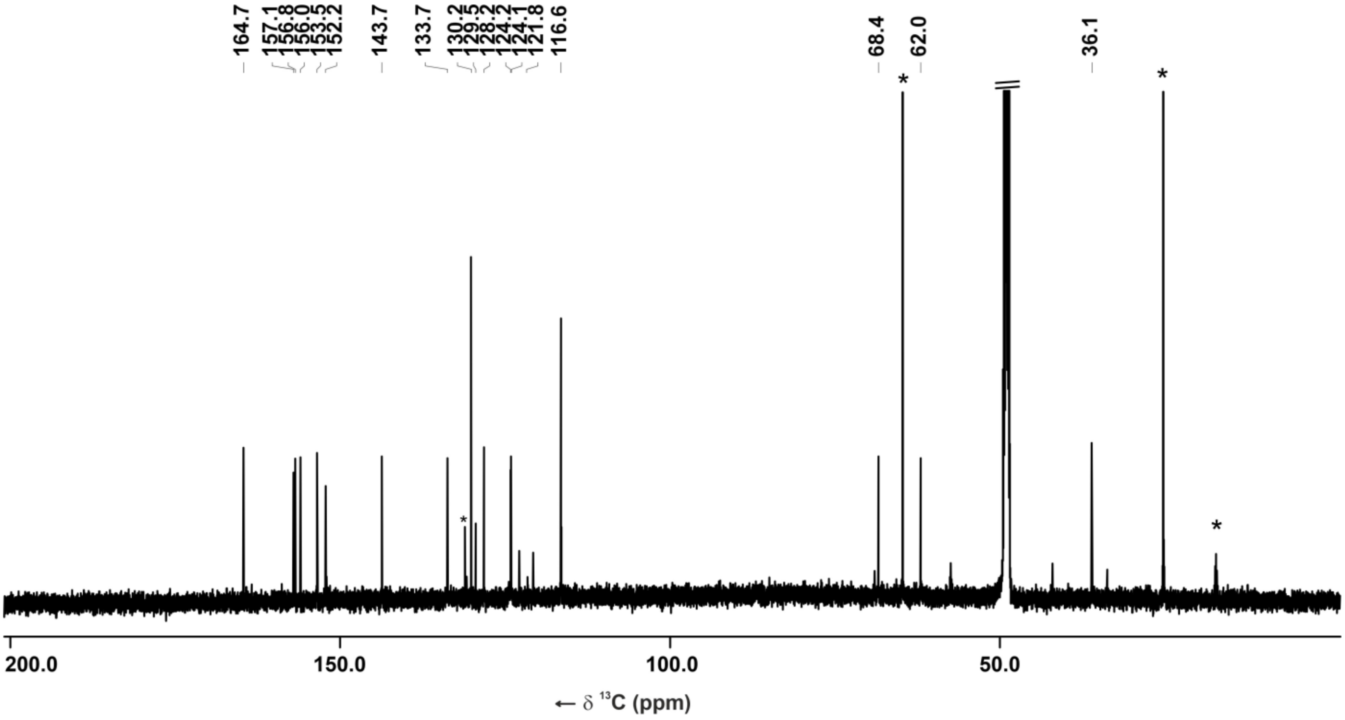

Figure S89. The ^{13}C NMR spectrum of **9-2Ag₂** ([D₄]methanol, 300 K, 151 MHz). The signals corresponding to impurities were marked with asterisks.

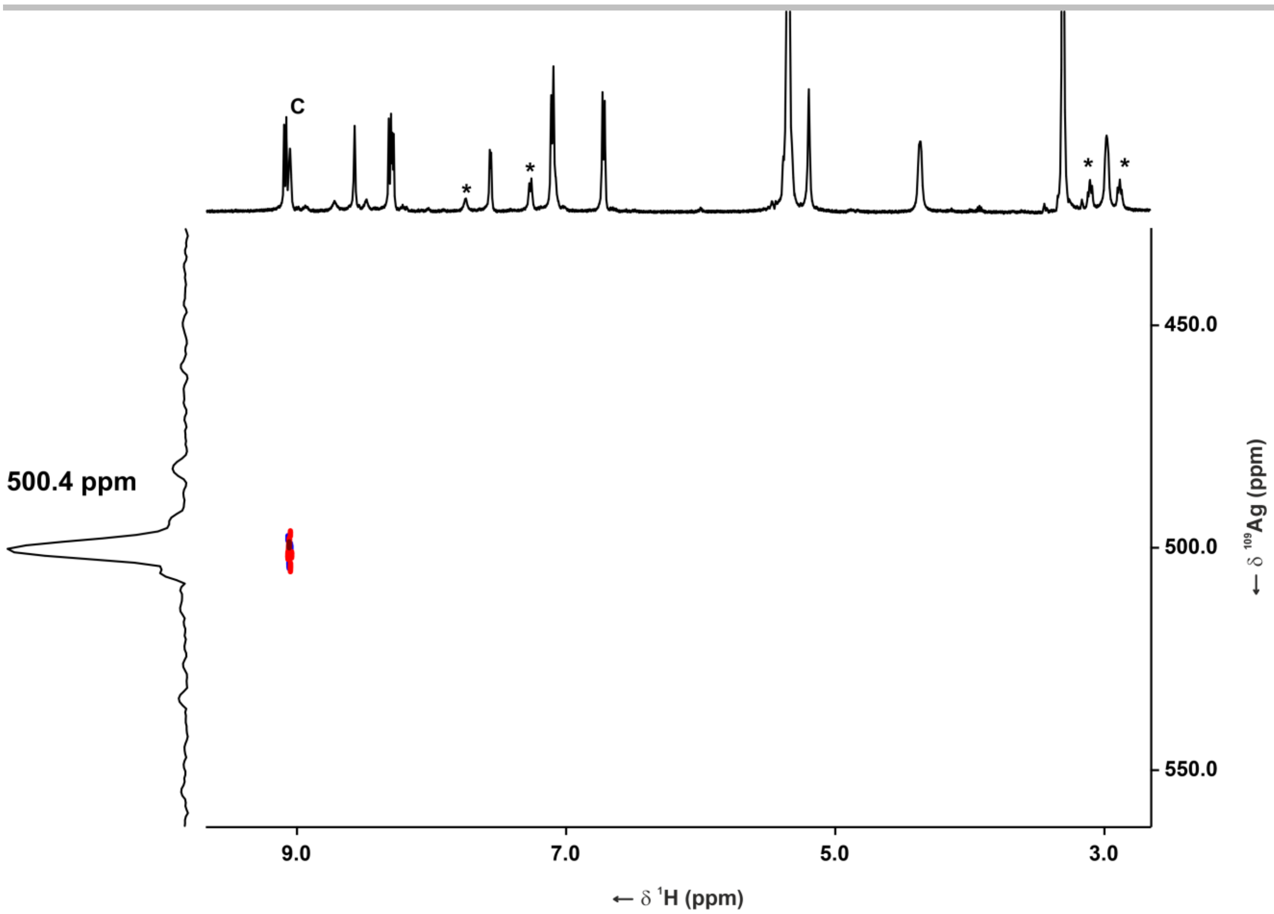

Figure S90. The ^{1}H-^{109}Ag HMBC spectrum of **9-2Ag₂** ([D₄]methanol, 240 K, 500 MHz).

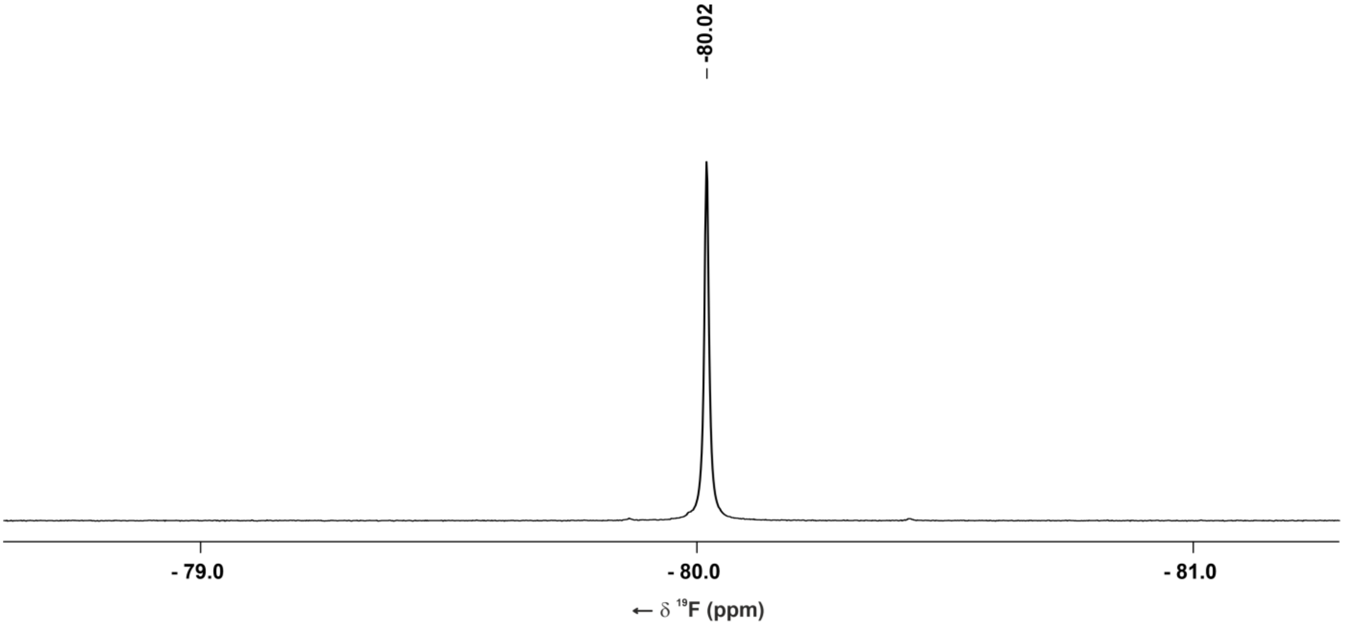

Figure S91. The ^{19}F NMR spectrum of **9-2Ag₂** ([D₄]methanol, 300 K, 565 MHz).

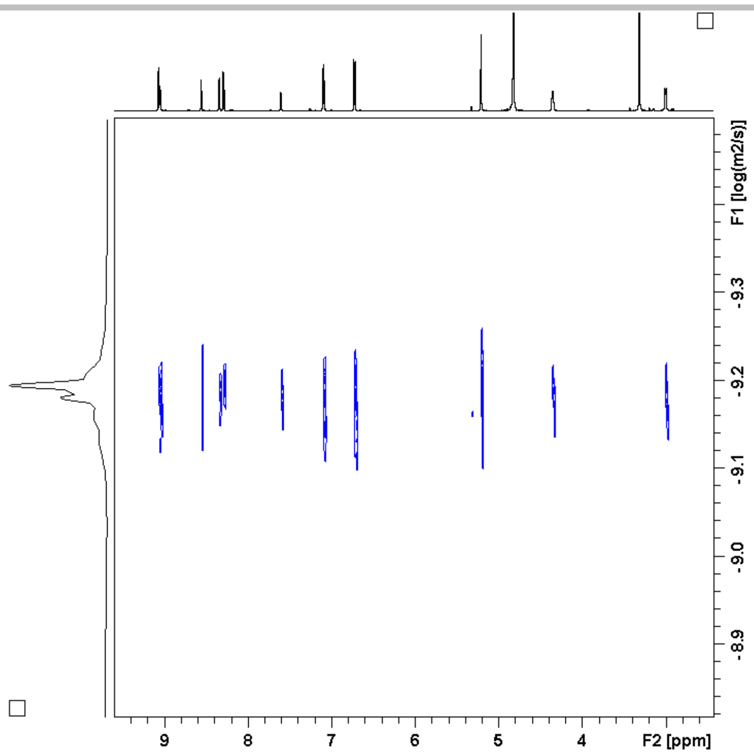

Figure S92. The DOSY spectrum of **9-2Ag$_2$** ([D$_4$]methanol, 300 K, 600 MHz).

NMR spectra of Solomon link 10-4Ag₂

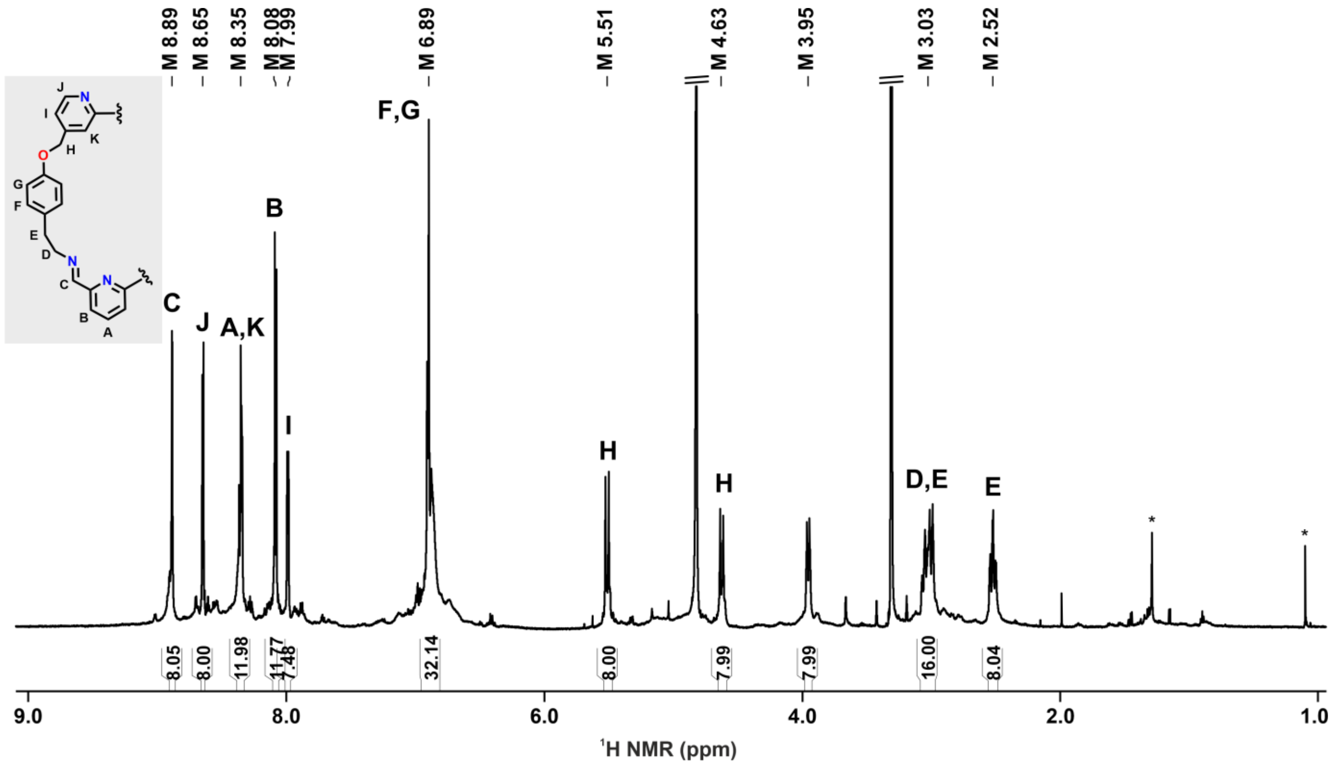

Figure S93. The ¹H NMR spectrum of **10-4Ag$_2$** ([D$_4$]methanol, 300 K, 600 MHz). The signals corresponding to impurities were marked with asterisks.

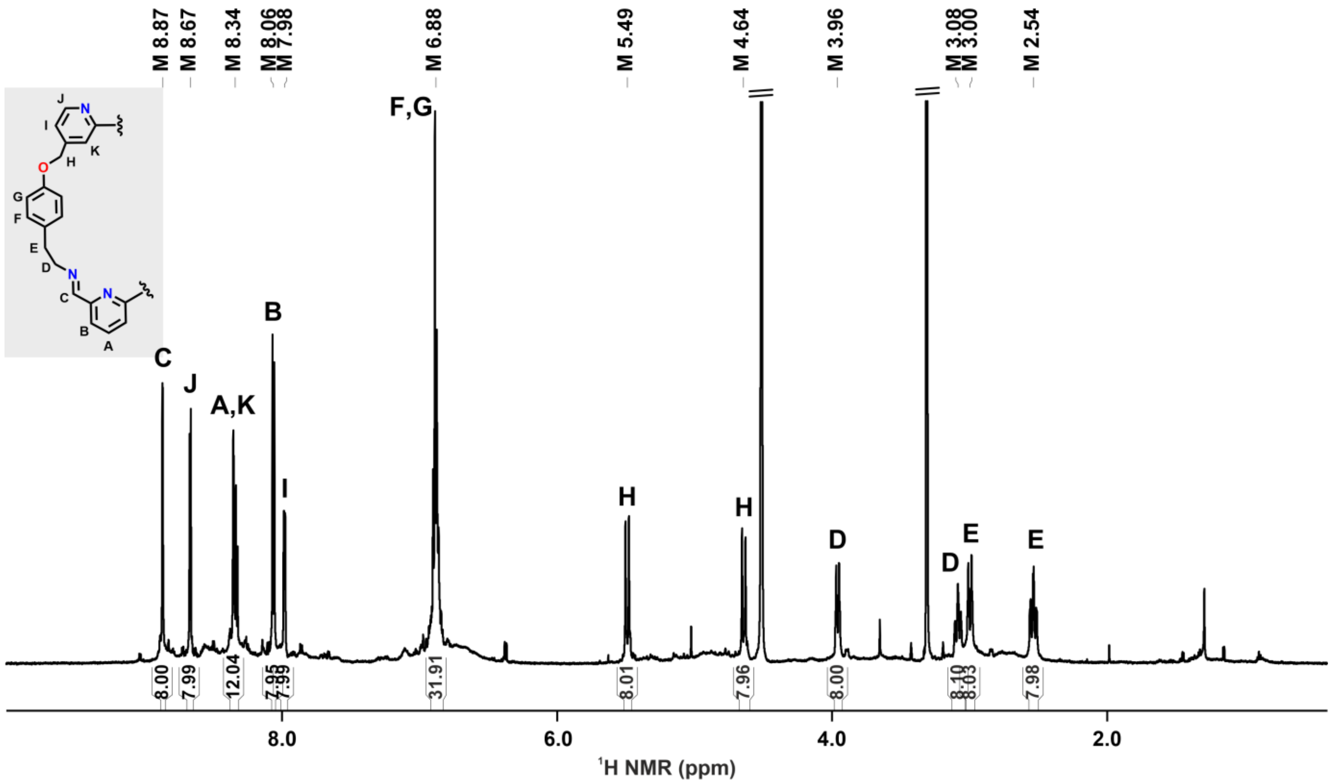

Figure S94. The ¹H NMR spectrum of **10-4Ag$_2$** ([D$_4$]methanol, 330 K, 600 MHz).

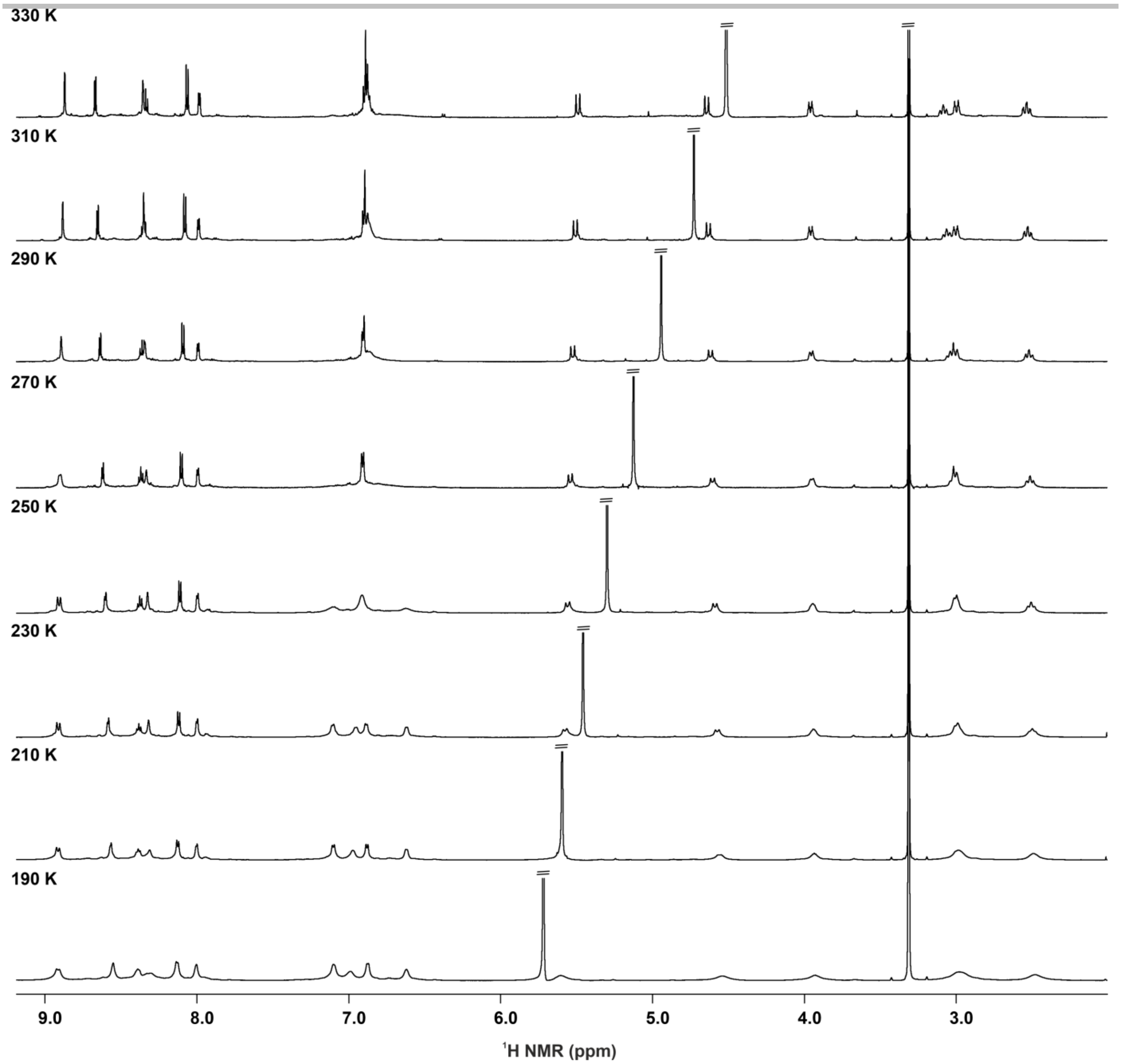

Figure S95. The ^{1}H NMR spectra of **10-4Ag₂** recorded in the 330 K – 190 K temperature range ([D$_4$]methanol, 600 MHz).

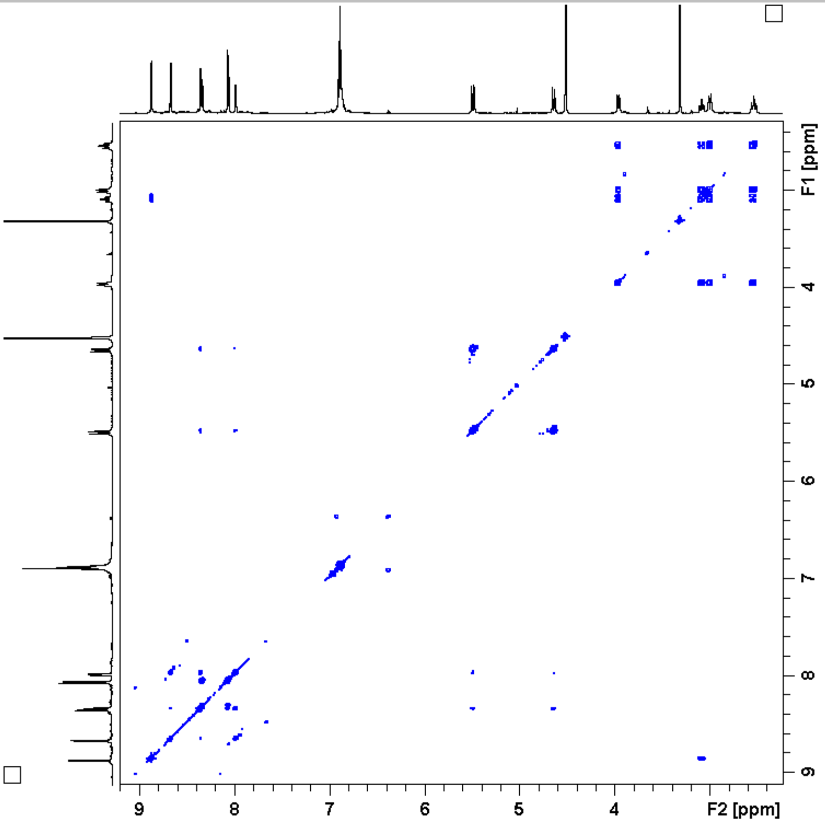

Figure S96. The ^{1}H-^{1}H COSY spectrum of **10-4Ag₂** ([D₄]methanol, 330 K, 600 MHz).

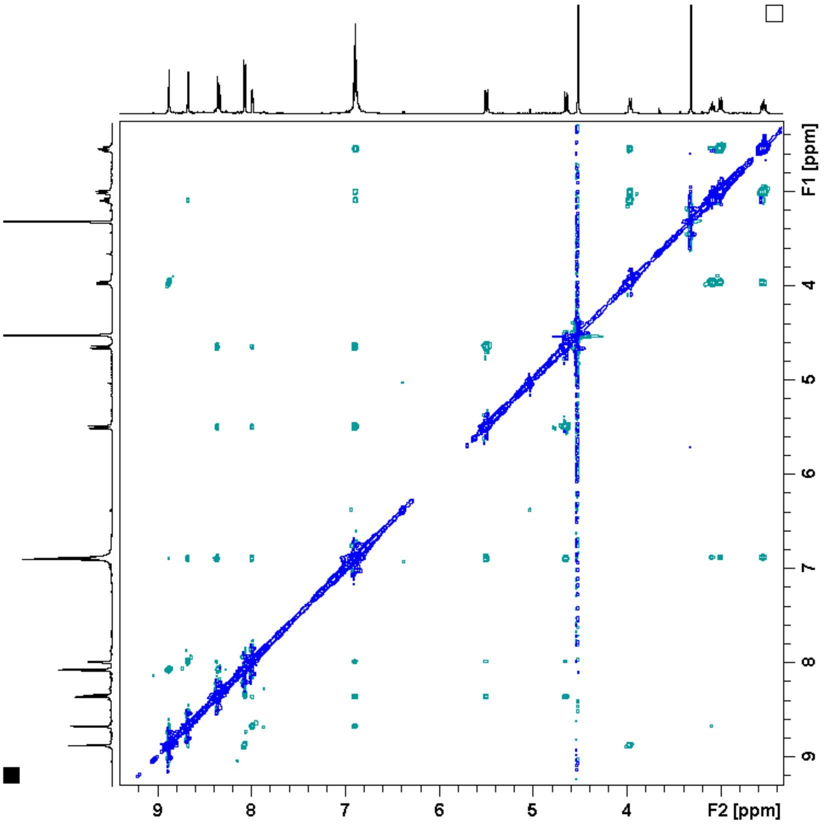

Figure S97. The ^{1}H-^{1}H ROESY spectrum of **10-4Ag₂** ([D₄]methanol, 330 K, 600 MHz).

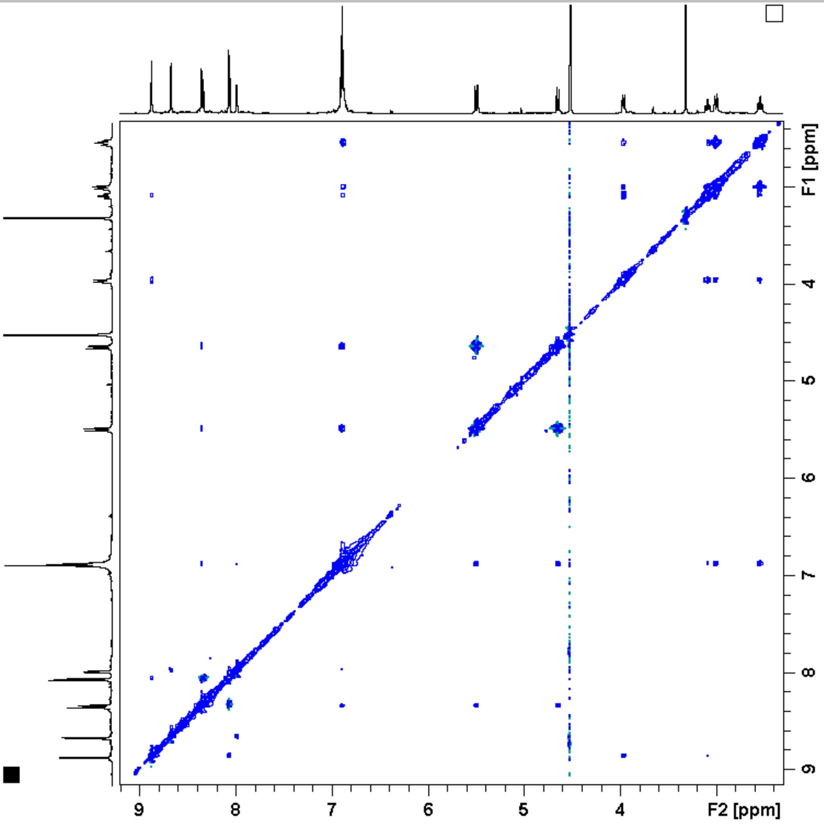

Figure S98. The ^{1}H-^{1}H NOESY spectrum of **10-4Ag$_2$** ([D$_4$]methanol, 330 K, 600 MHz).

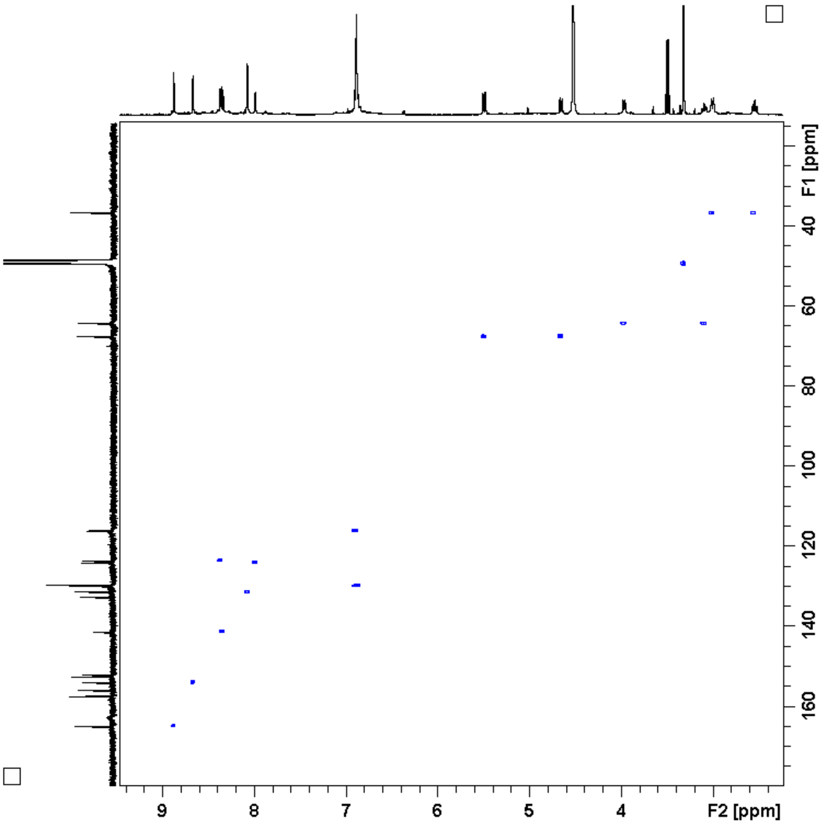

Figure S99. The ^{1}H-^{13}C HSQC spectrum of **10-4Ag$_2$** ([D$_4$]methanol, 330 K, 600 MHz).

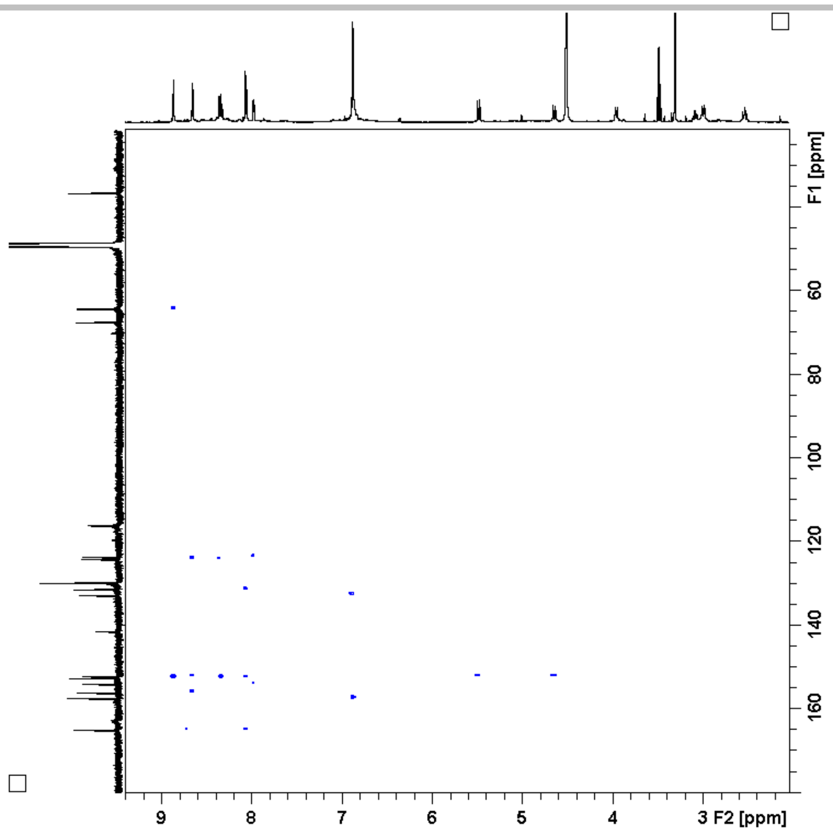

Figure S100. The ^{1}H-^{13}C HMBC spectrum of **10-4Ag₂** ([D₄]methanol, 330 K, 600 MHz).

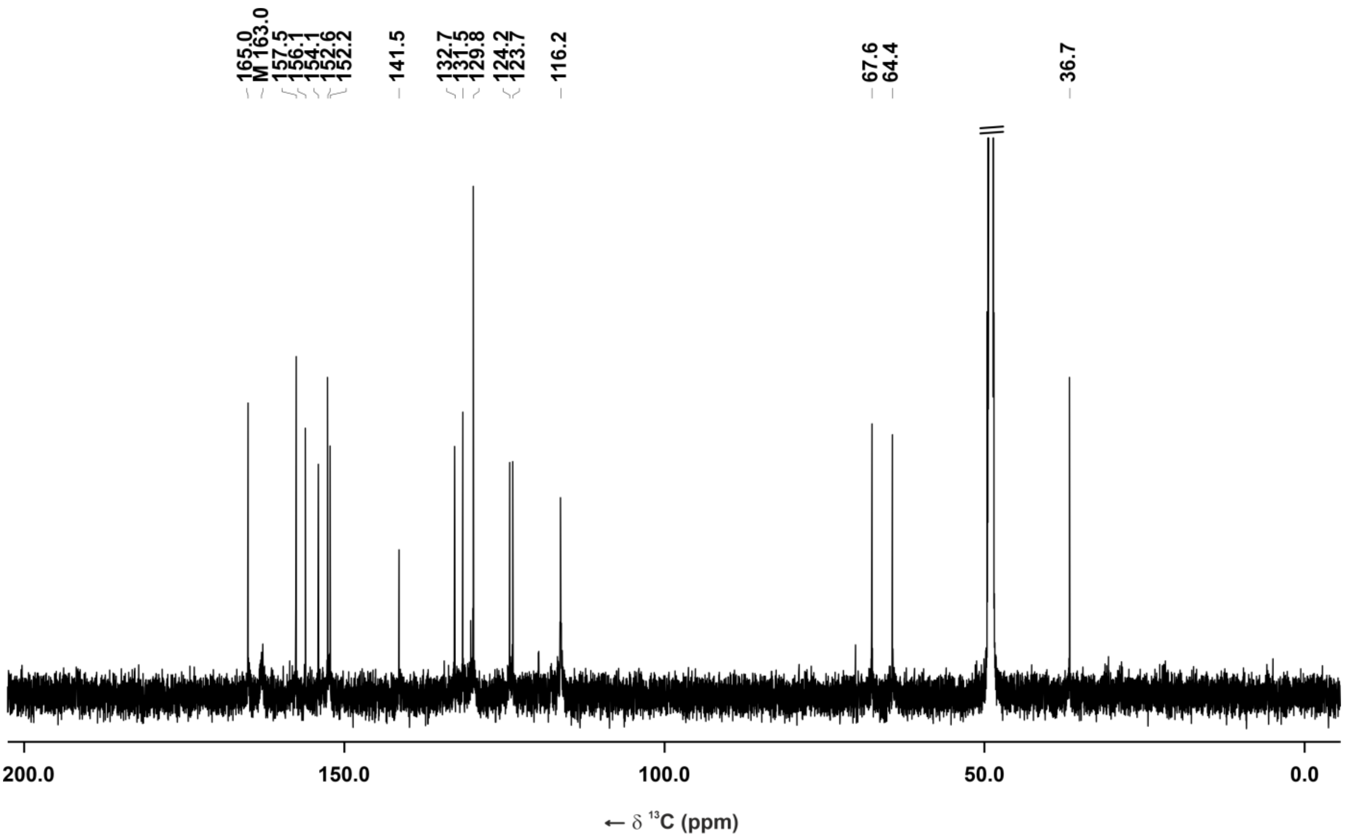

Figure S101. The ^{13}C NMR spectrum of **10-4Ag₂** ([D₄]methanol, 330 K, 151 MHz).

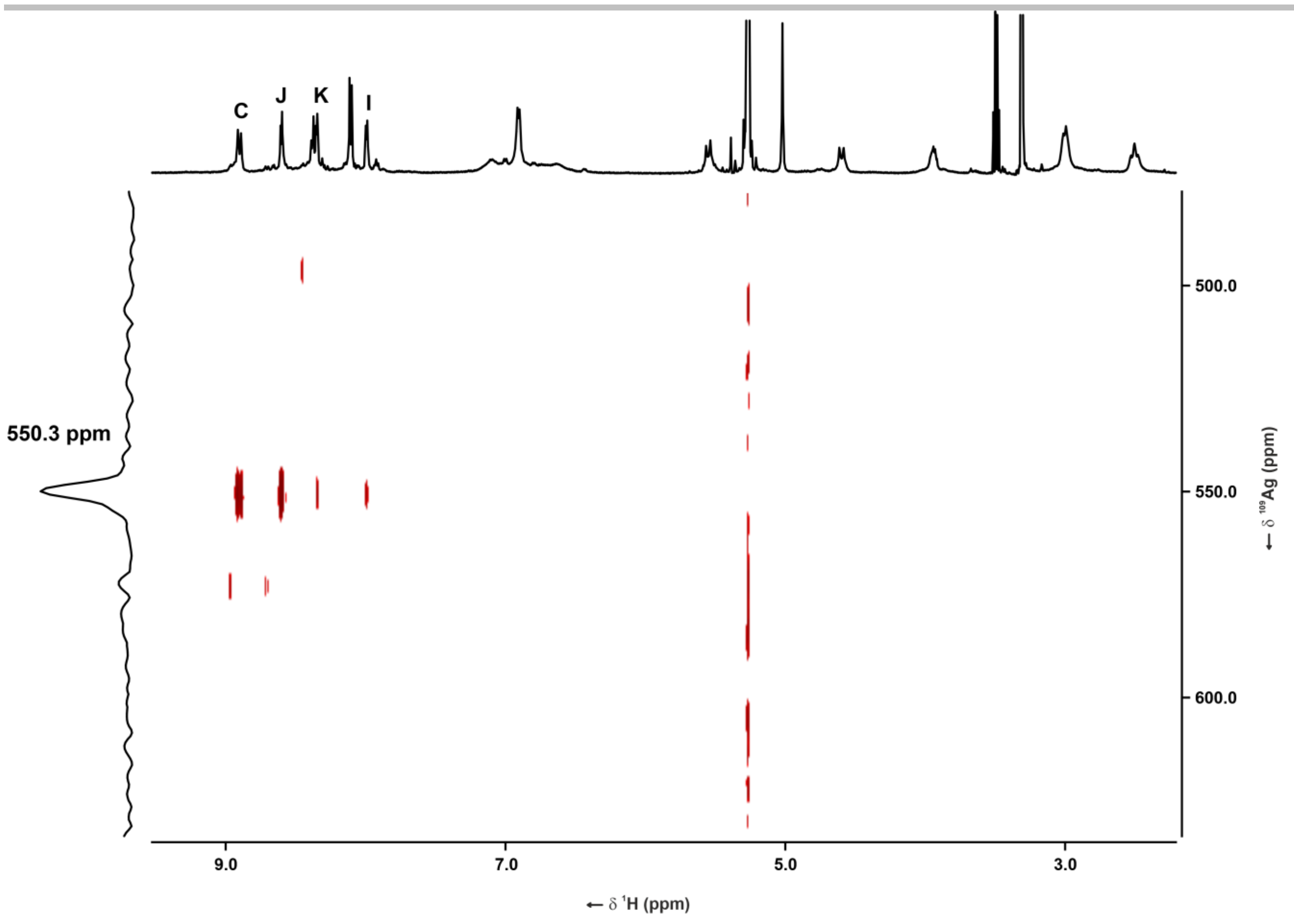

Figure S102. The ^{1}H-^{109}Ag HMBC spectrum of **10-4Ag₂** ([D₄]methanol, 250 K, 600 MHz).

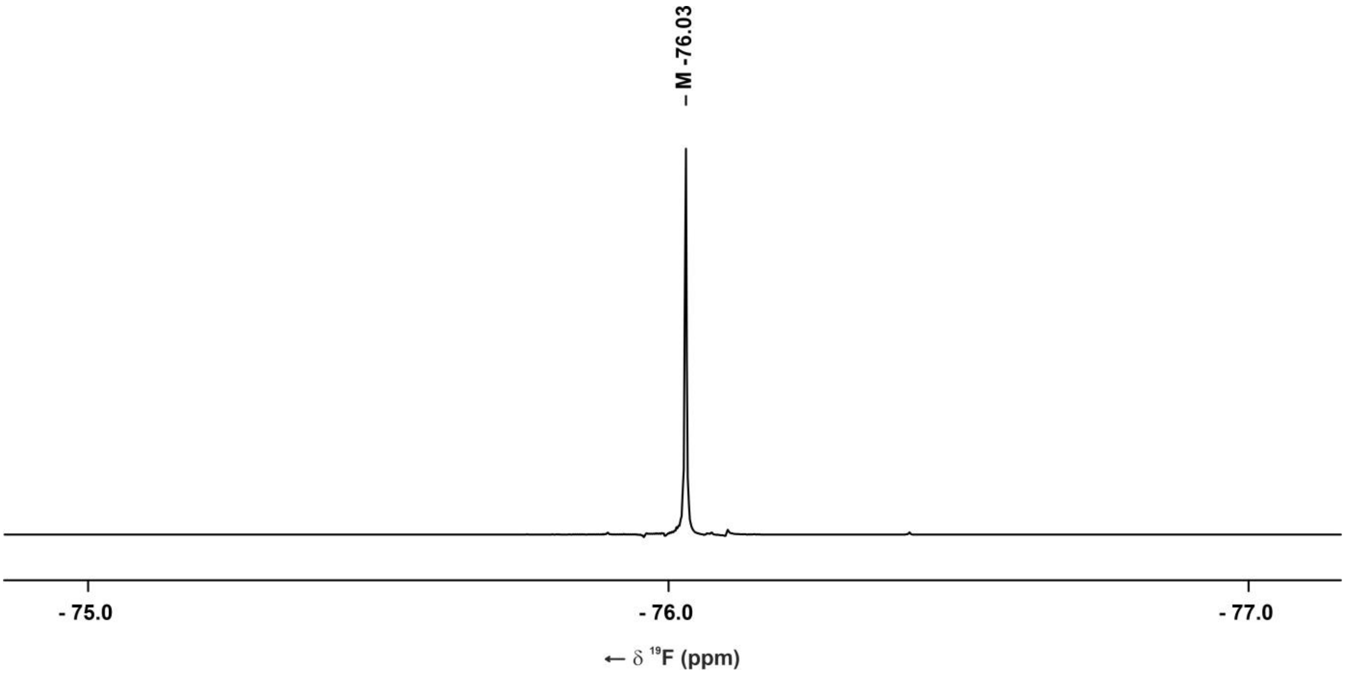

Figure S103. The ^{19}F NMR spectrum of **10-4Ag$_2$** ([D$_4$]methanol, 300 K, 565 MHz).

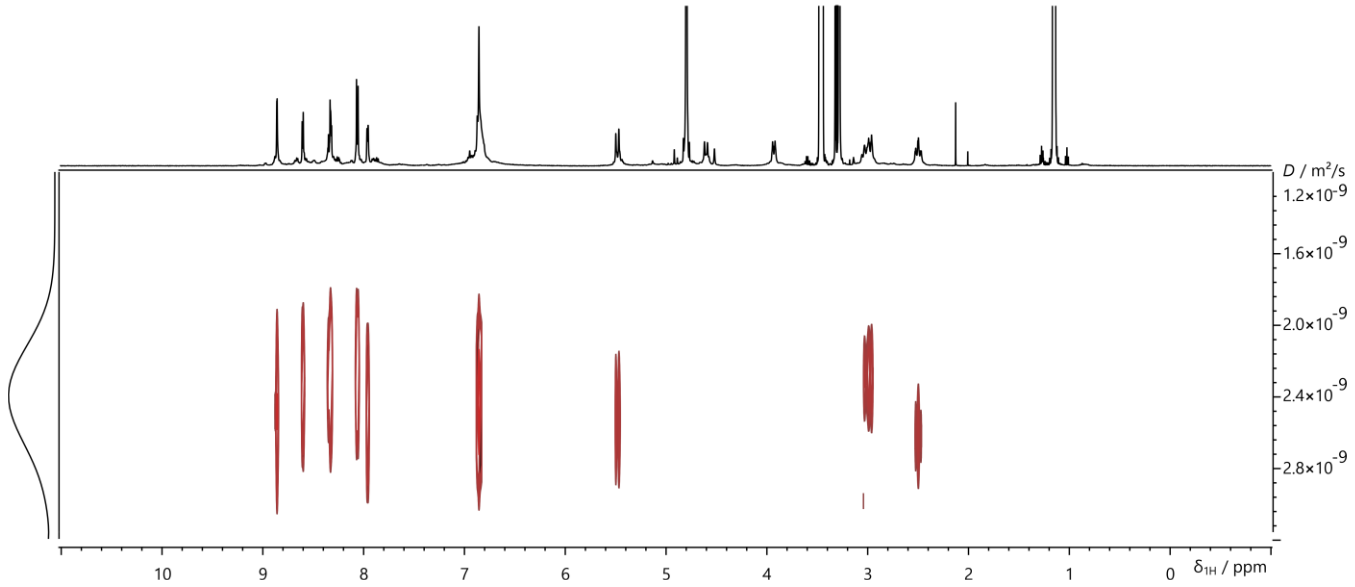

Figure S104. The DOSY spectrum of **10-4Ag2** ([D4]methanol, 300 K, 600 MHz).

Titration of the Solomon Link 10-4Ag₂ with KPF₆.

The 0.5 mL solution of **10-4Ag$_2$** (1.56 mM) in [D$_4$]methanol was titrated with a solution of KPF_6 in [D$_4$]methanol. After each addition, the tube was shaken vigorously for about a minute, and the ^{1}H NMR spectrum was recorded at 300 K.

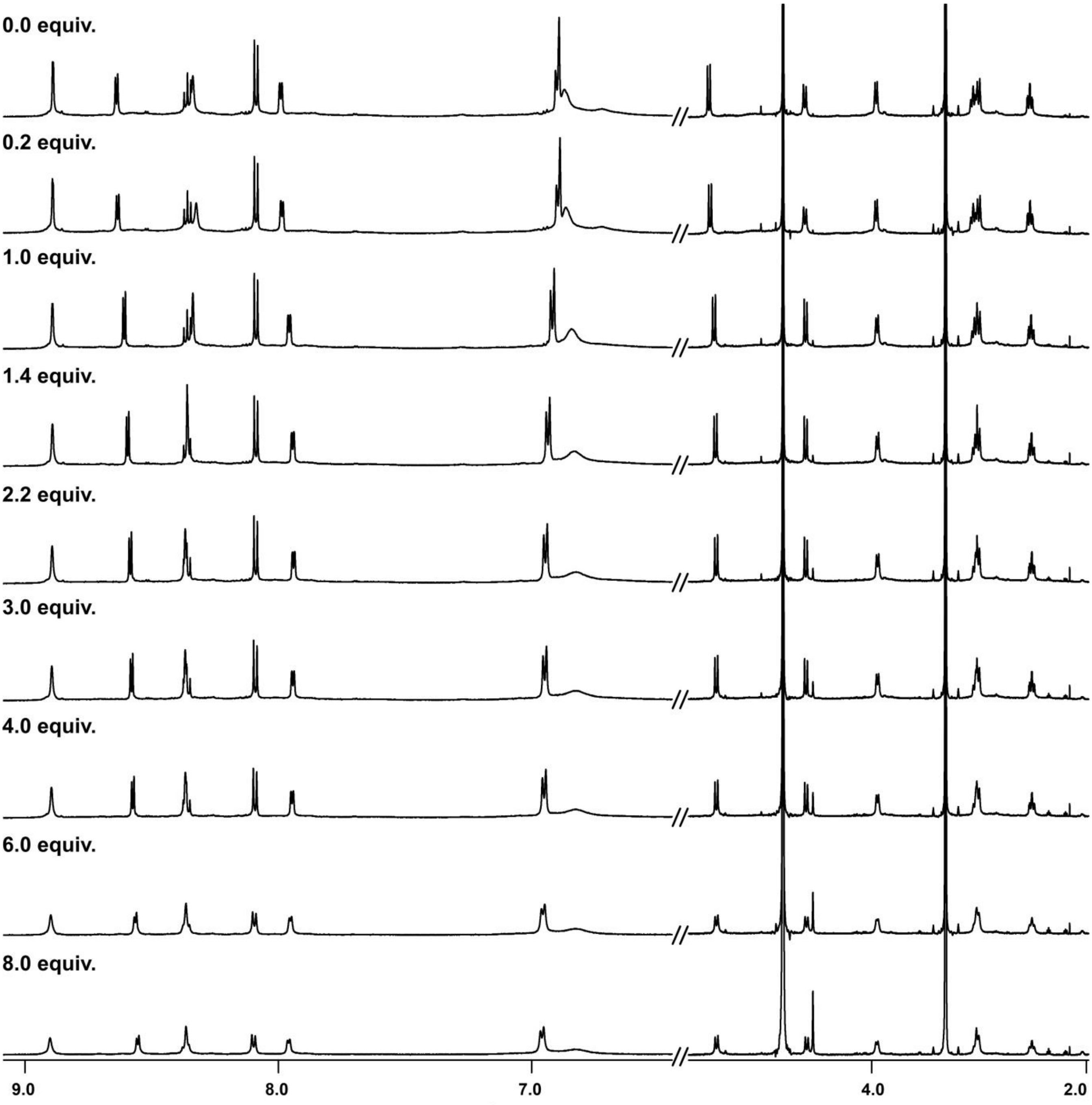

Figure S105. Partial ^{1}H NMR spectra (600 MHz, [D$_4$]methanol, 300 K) recorded upon the titration of methanolic solution of KPF_6 into a solution of **10-4Ag$_2$**.

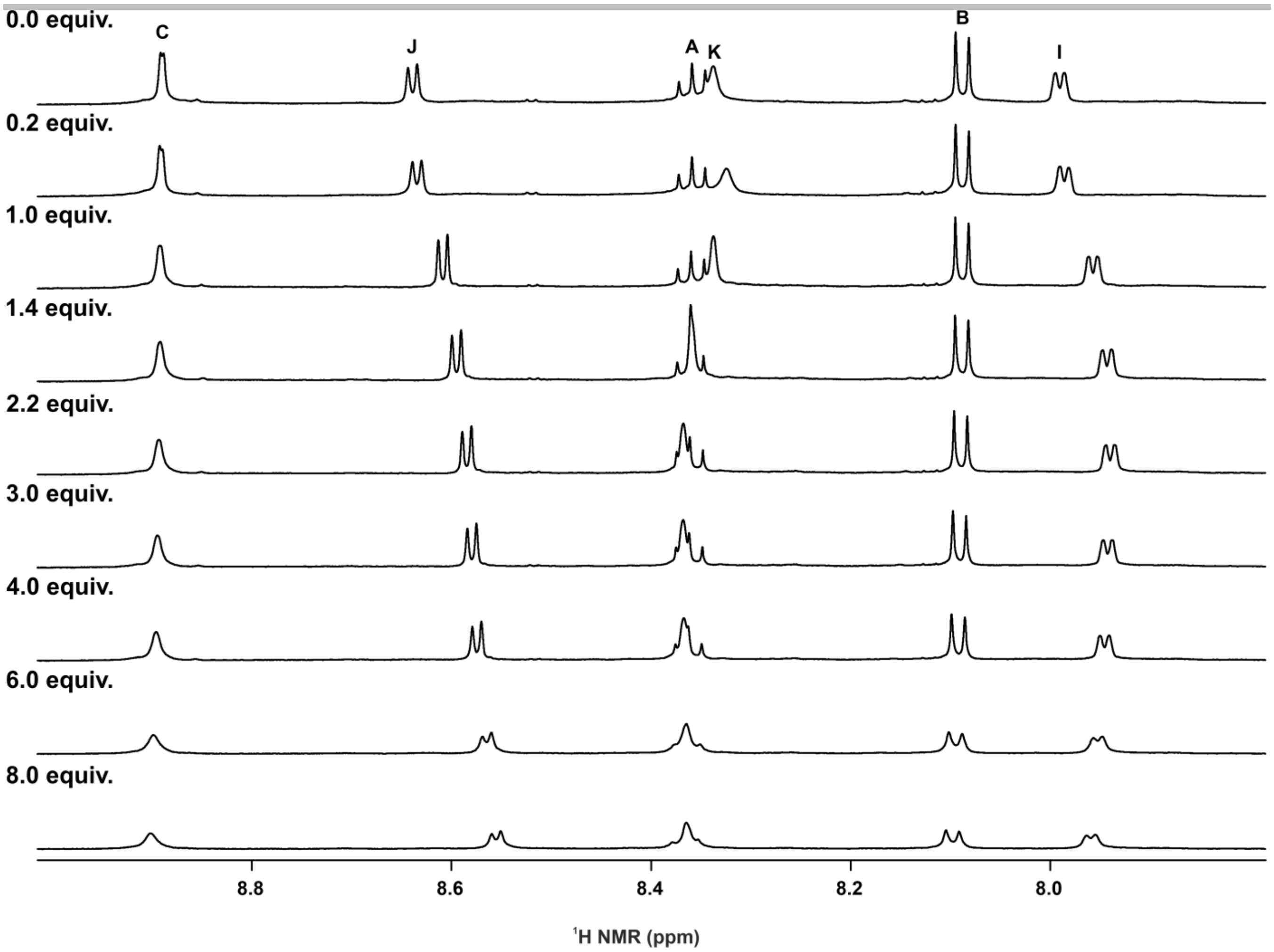

Figure S106. Partial ^{1}H NMR spectra (600 MHz, [D$_4$]methanol, 300 K) recorded upon the titration of methanolic solution of KPF_6 into a solution of **10-4Ag$_2$**.

NMR spectra of Solomon link $(PF_6)_2 \subset$ 10-4Ag$_2$

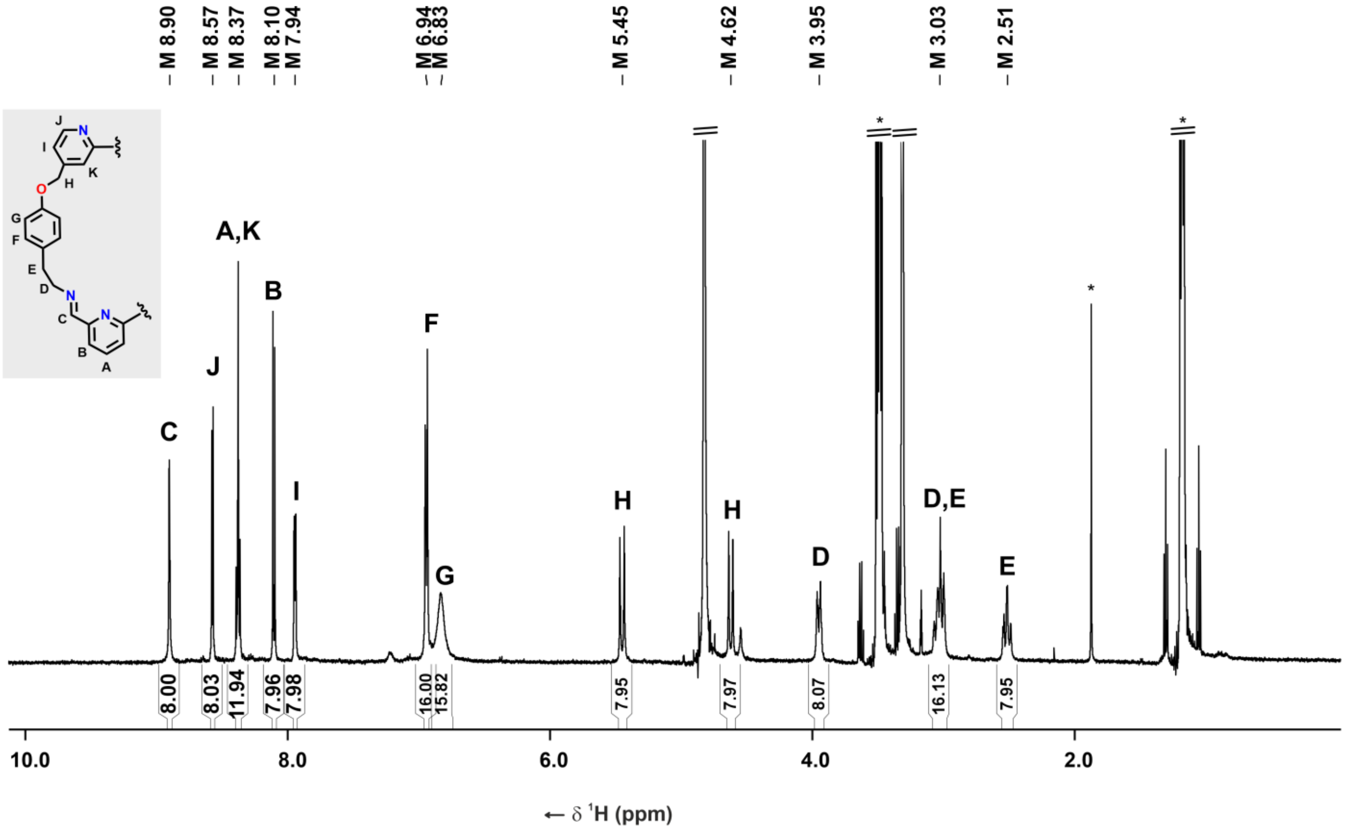

Figure S107. The ^{1}H NMR spectrum of **$(PF_6)_2 \subset$ 10-4Ag$_2$** ([D$_4$]methanol, 300 K, 500 MHz). The signals corresponding to impurities were marked with asterisks.

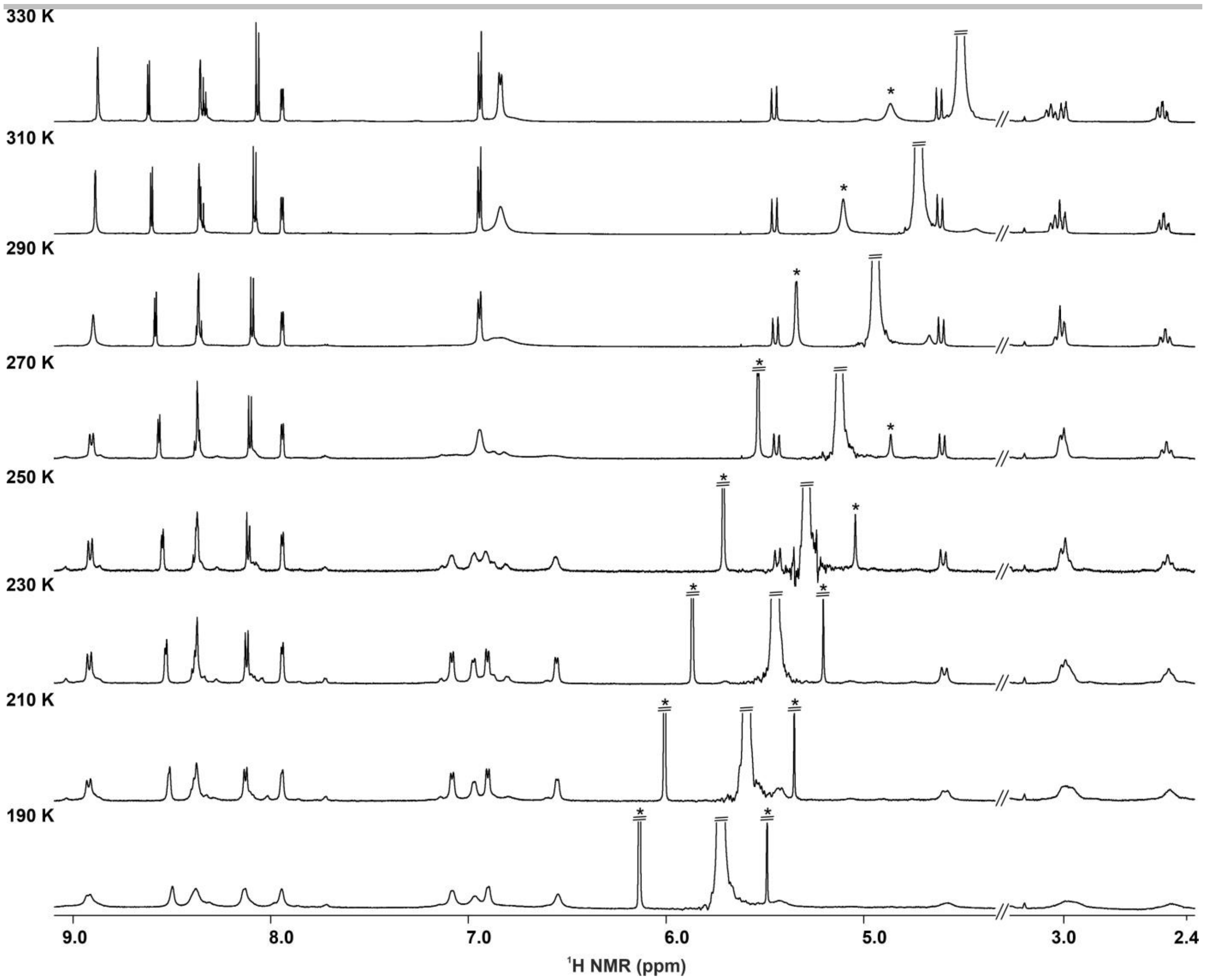

Figure S108. The ^{1}H NMR spectra of **$(PF_6)_2 \subset$ 10-4Ag$_2$** recorded in the 330 K – 190 K temperature range ([D$_4$]methanol, 600 MHz).

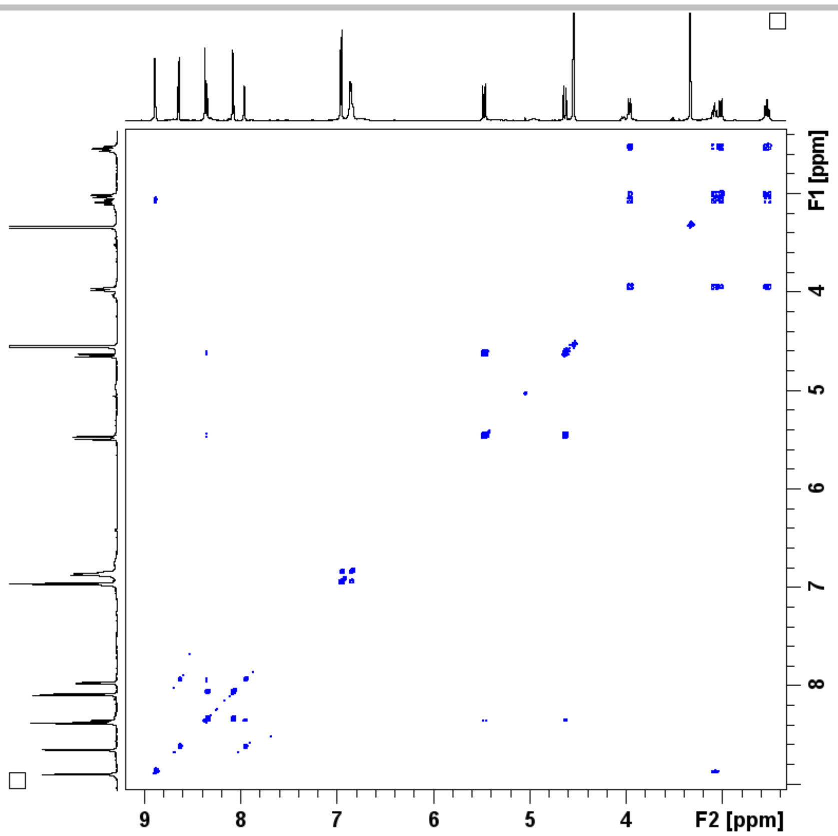

Figure S109. The ^{1}H-^{1}H COSY spectrum of **$(PF_6)_2 \subset$ 10-4Ag$_2$** ([D$_4$]methanol, 330 K, 600 MHz).

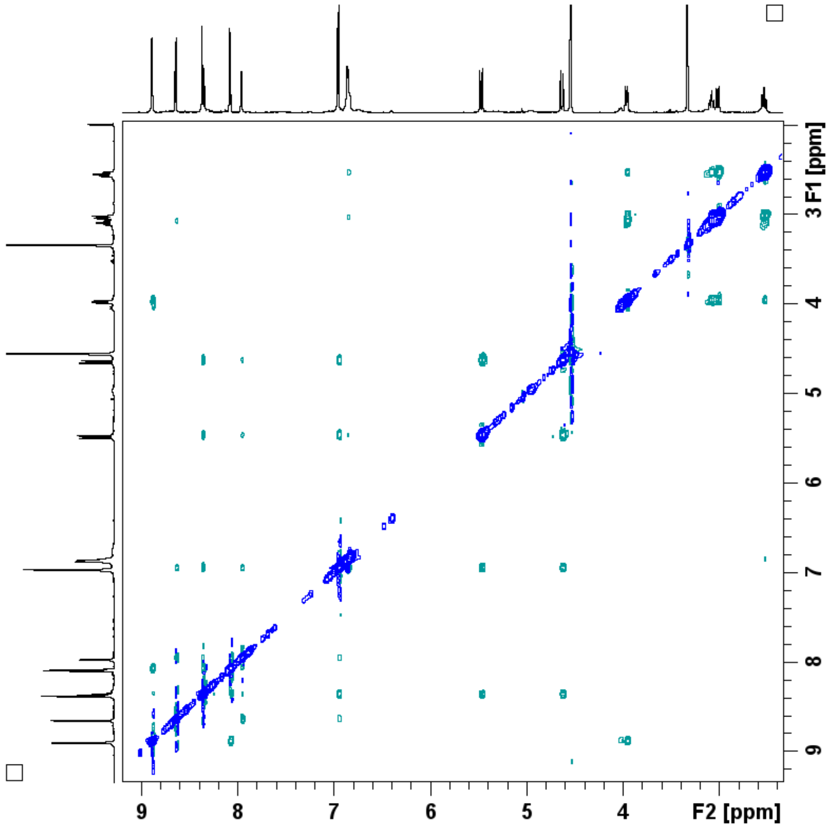

Figure S110. The ^{1}H-^{1}H ROESY spectrum of **$(PF_6)_2 \subset$ 10-4Ag$_2$** ([D$_4$]methanol, 330 K, 600 MHz).

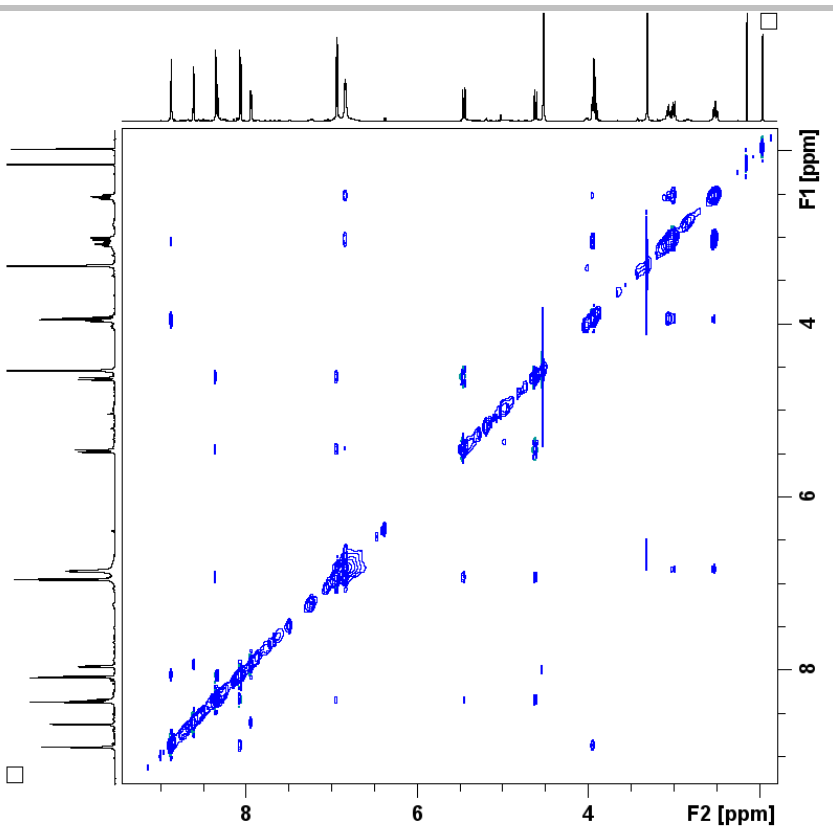

Figure S111. The ^{1}H-^{1}H NOESY spectrum of **$(PF_6)_2 \subset$ 10-4Ag$_2$** ([D$_4$]methanol, 330 K, 600 MHz).

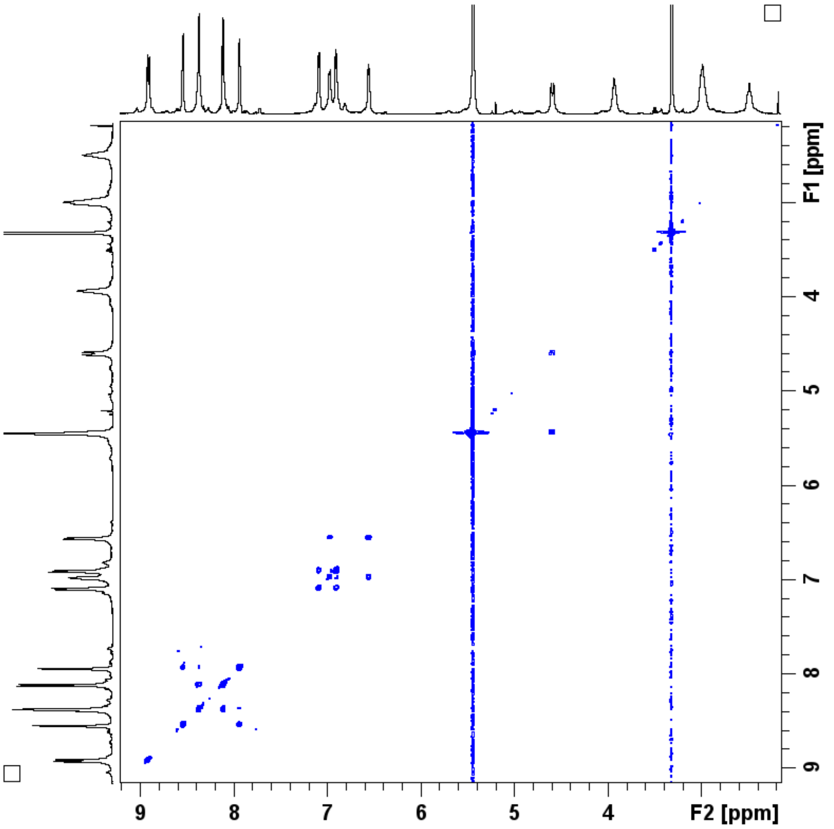

Figure S112. The ^{1}H-^{1}H COSY spectrum of **$(PF_6)_2 \subset$ 10-4Ag$_2$** ([D$_4$]methanol, 230 K, 600 MHz).

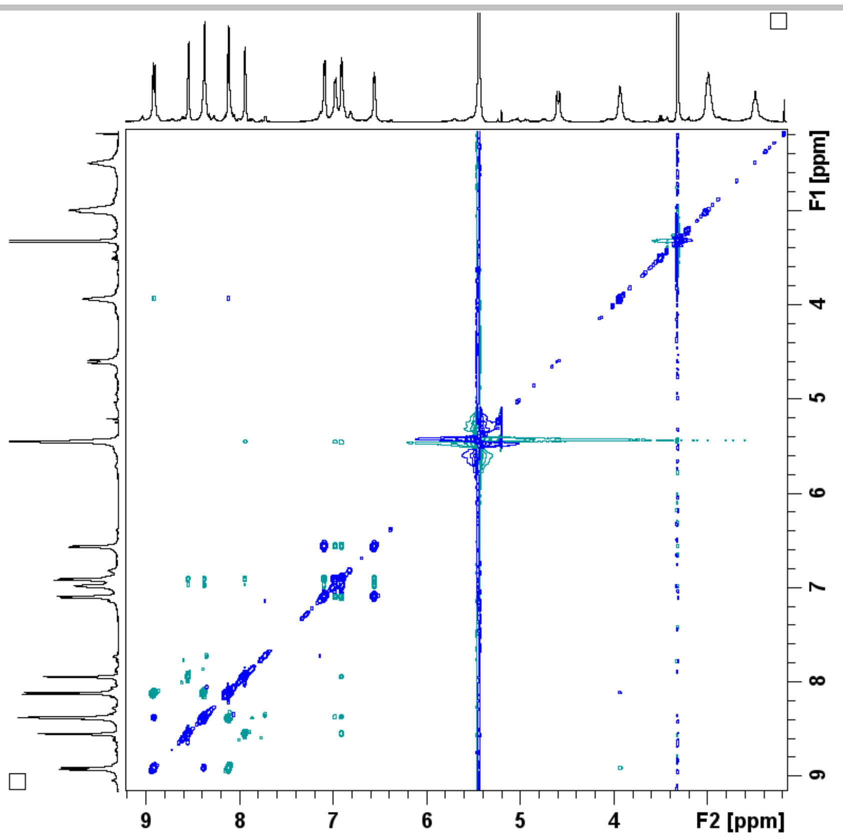

Figure S113. The ^{1}H-^{1}H ROESY spectrum of **$(PF_6)_2 \subset$ 10-4Ag$_2$** ([D$_4$]methanol, 230 K, 600 MHz).

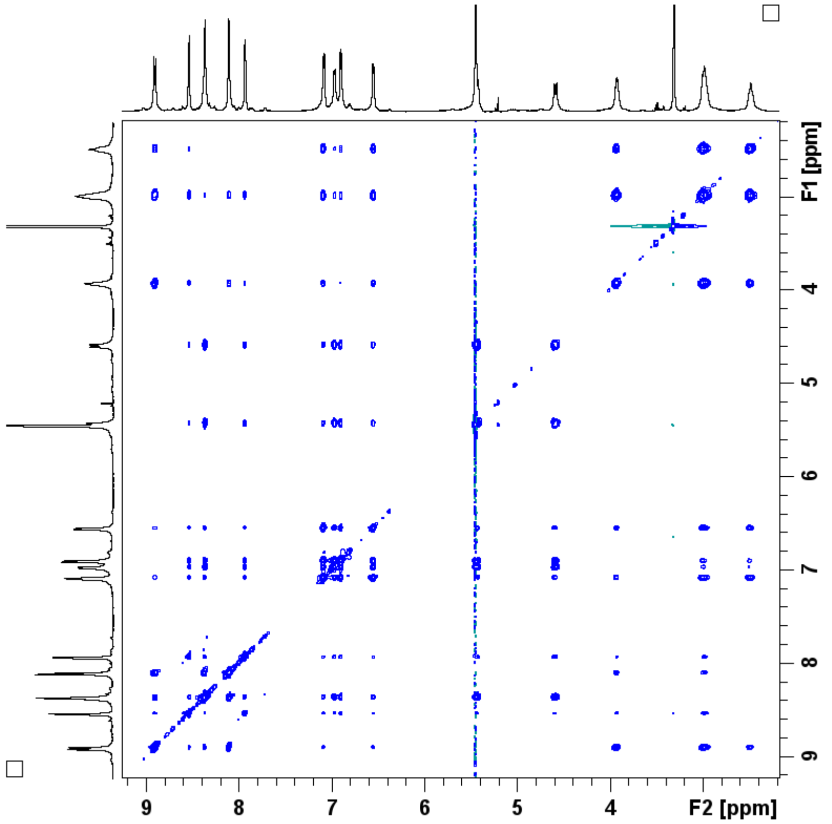

Figure S114. The ^{1}H-^{1}H NOESY spectrum of **$(PF_6)_2 \subset$ 10-4Ag$_2$** ([D$_4$]methanol, 230 K, 600 MHz).

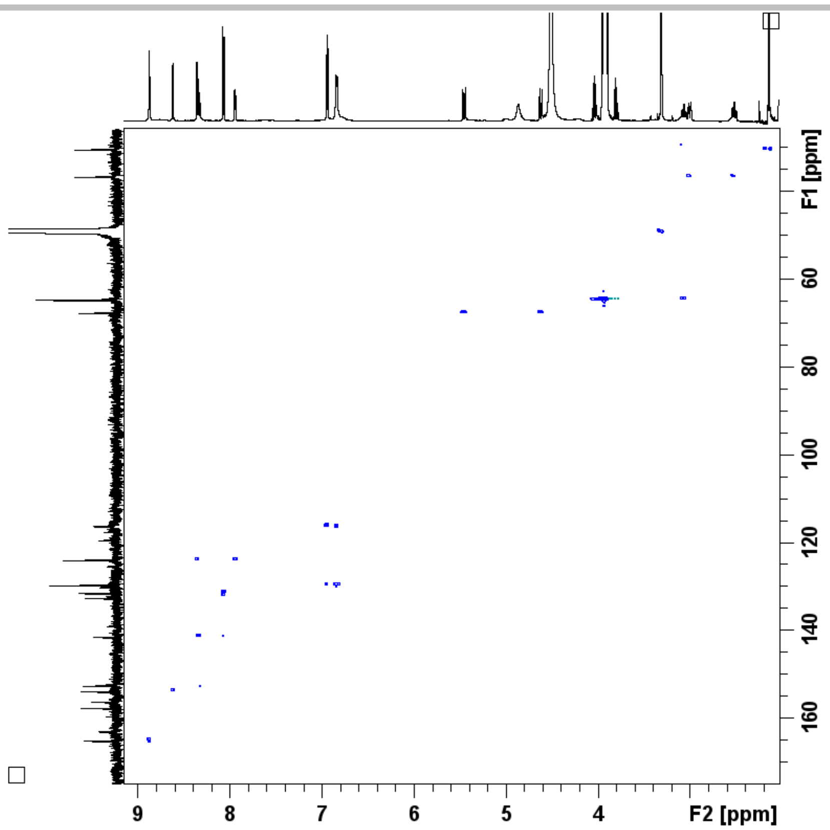

Figure S115. The ^{1}H-^{13}C HSQC spectrum of **$(PF_6)_2 \subset$ 10-4Ag$_2$** ([D$_4$]methanol, 330 K, 600 MHz).

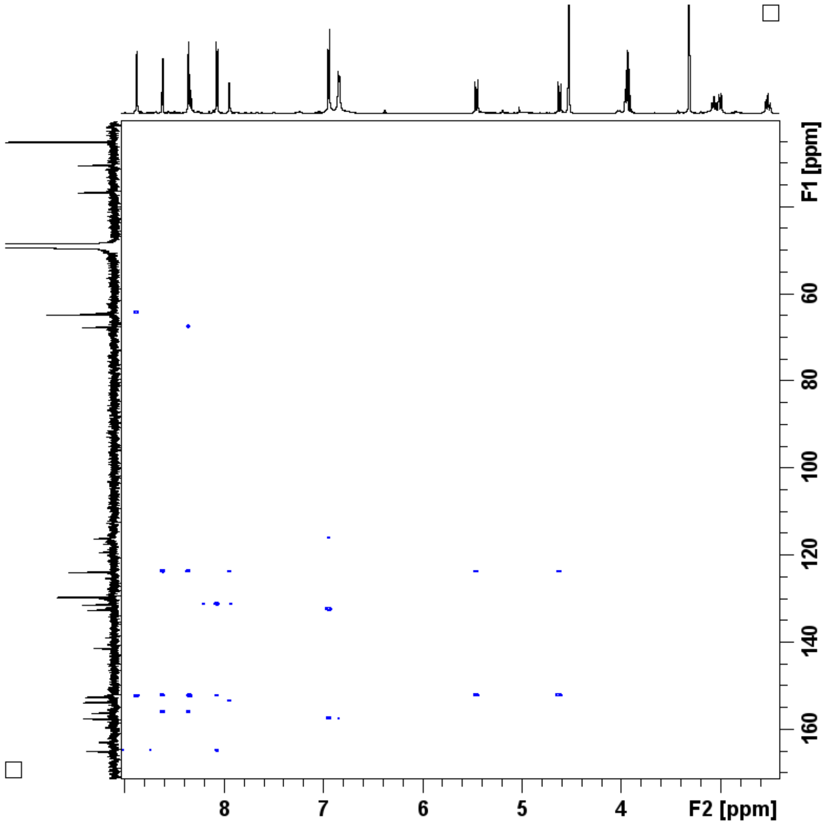

Figure S116. The ^{1}H-^{13}C HMBC spectrum of **$(PF_6)_2 \subset$ 10-4Ag$_2$** ([D$_4$]methanol, 330 K, 600 MHz).

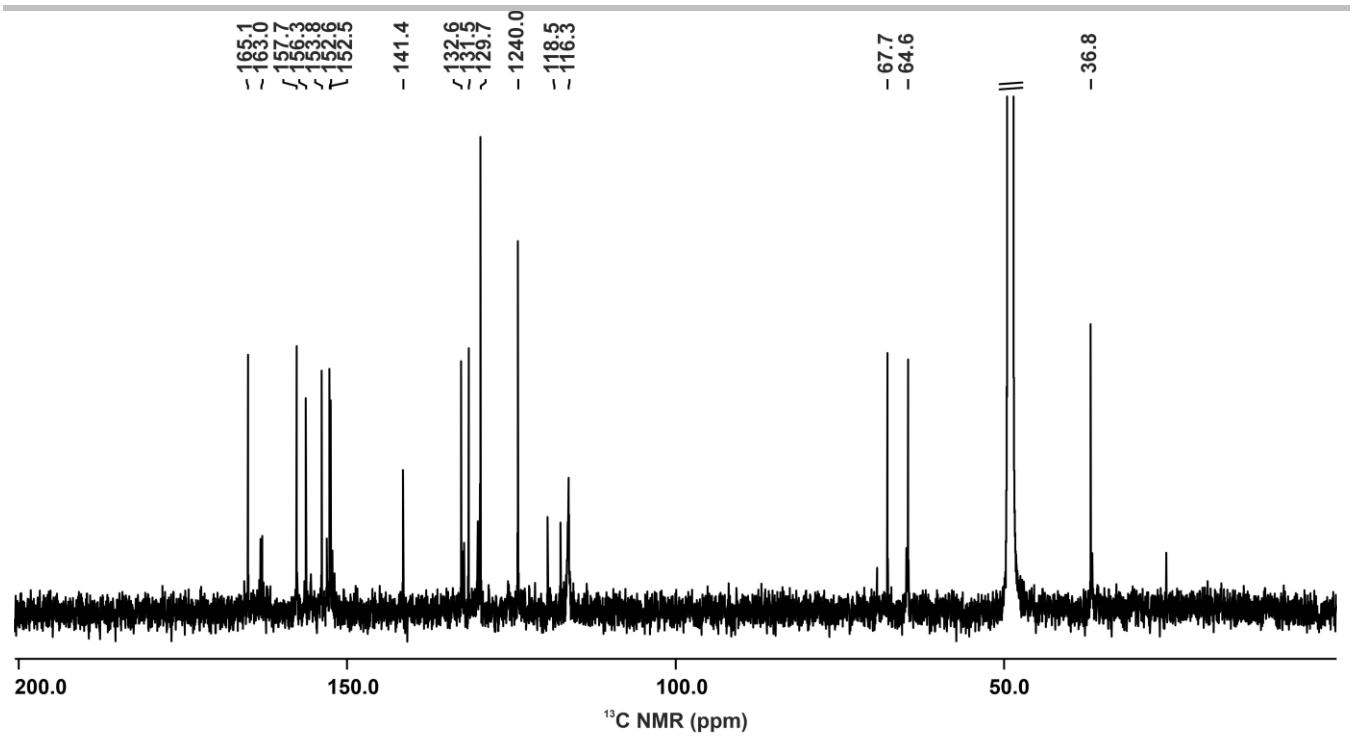

Figure S117. The ^{13}C NMR spectrum of **$(PF_6)_2 \subset$ 10-4Ag$_2$** ([D$_4$]methanol, 330 K, 151 MHz).

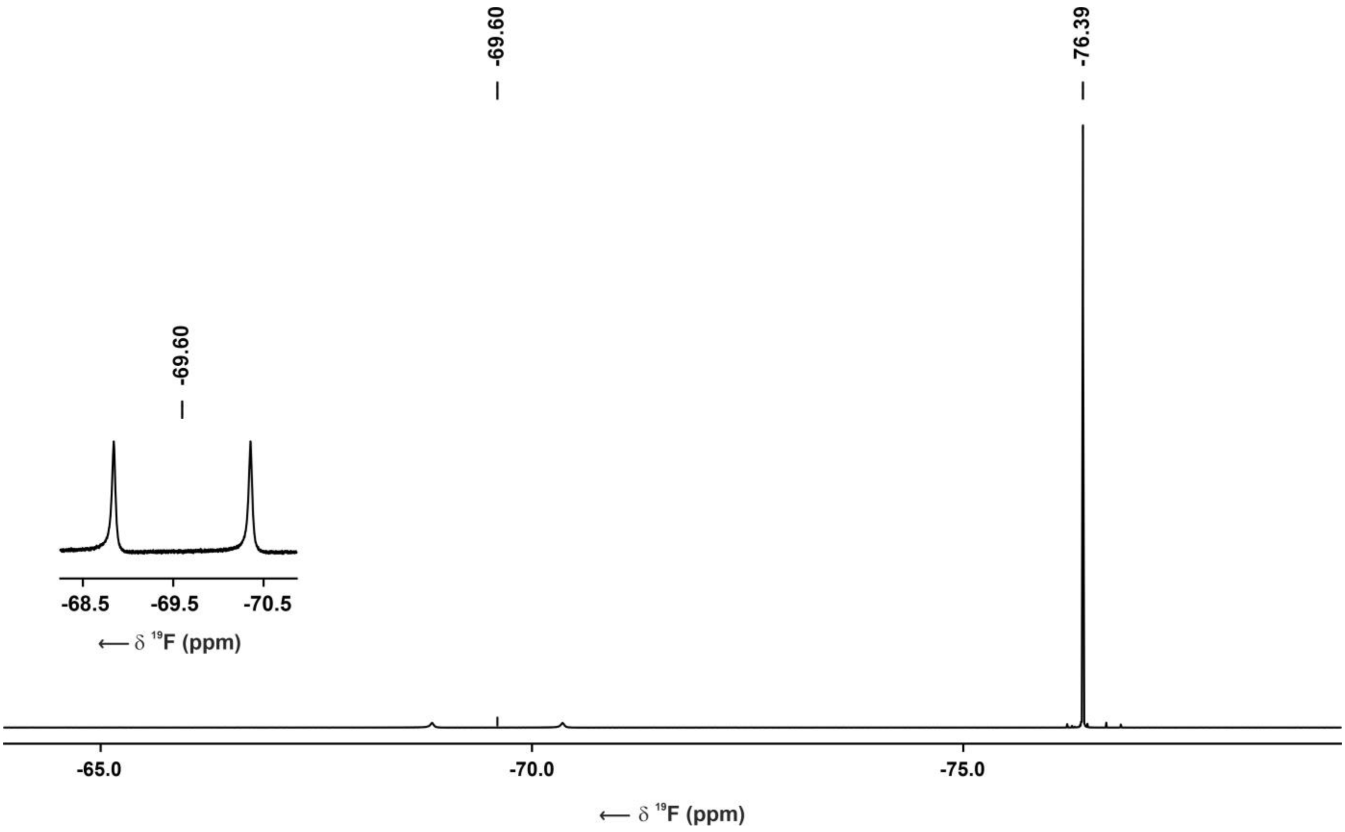

Figure S118. The ^{19}F NMR spectrum of **$(PF_6)_2 \subset$ 10-4Ag$_2$** ([D$_4$]methanol, 230 K, 471 MHz).

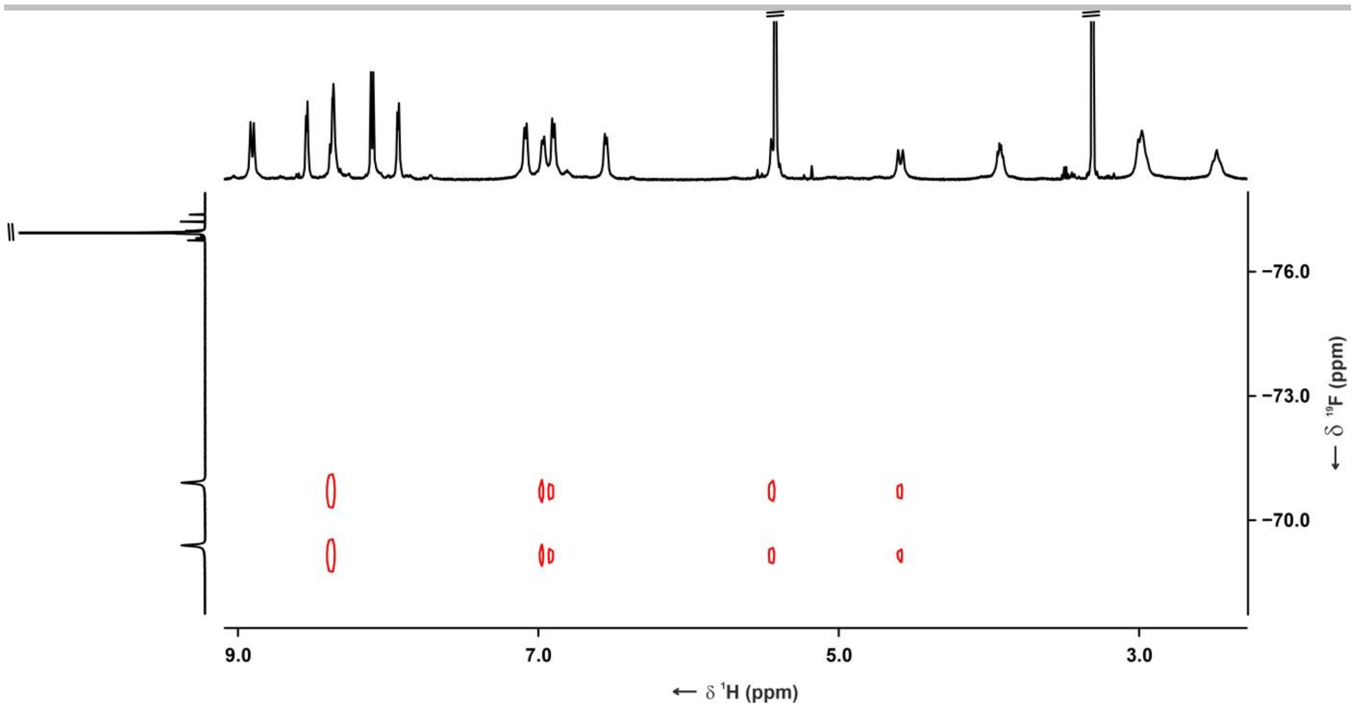

Figure S119. The ^{1}H-^{19}F HOESY spectrum of **$(PF_6)_2$ ⊂ 10-4Ag$_2$** ([D$_4$]methanol, 230 K, 500 MHz).

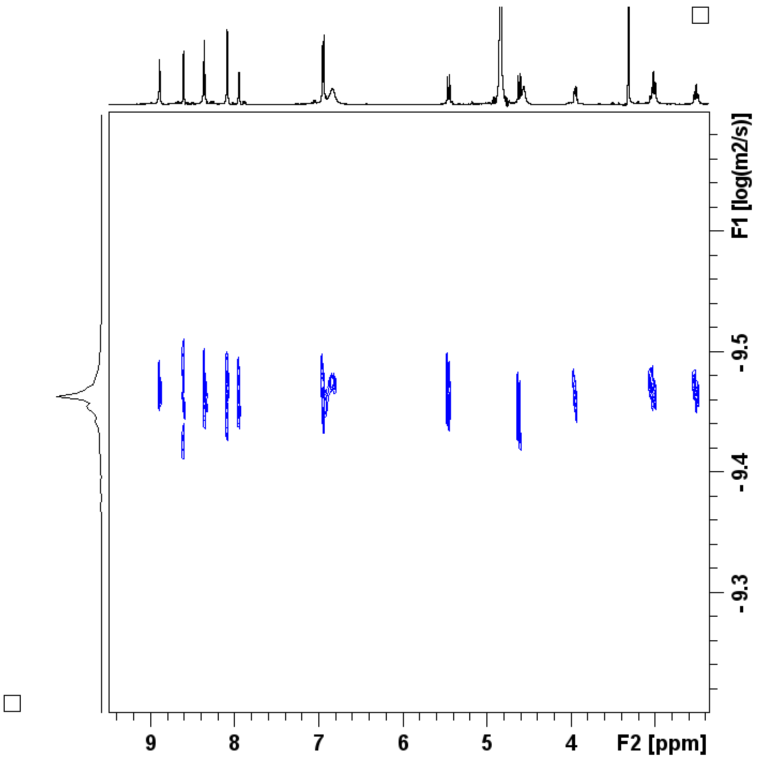

Figure S120. The DOSY spectrum of **$(PF_6)_2$ ⊂ 10-4Ag$_2$** ([D$_4$]methanol, 300 K, 600 MHz).

NMR spectra of Solomon link $(BF_4)_n \subset$ 10-4Ag$_2$

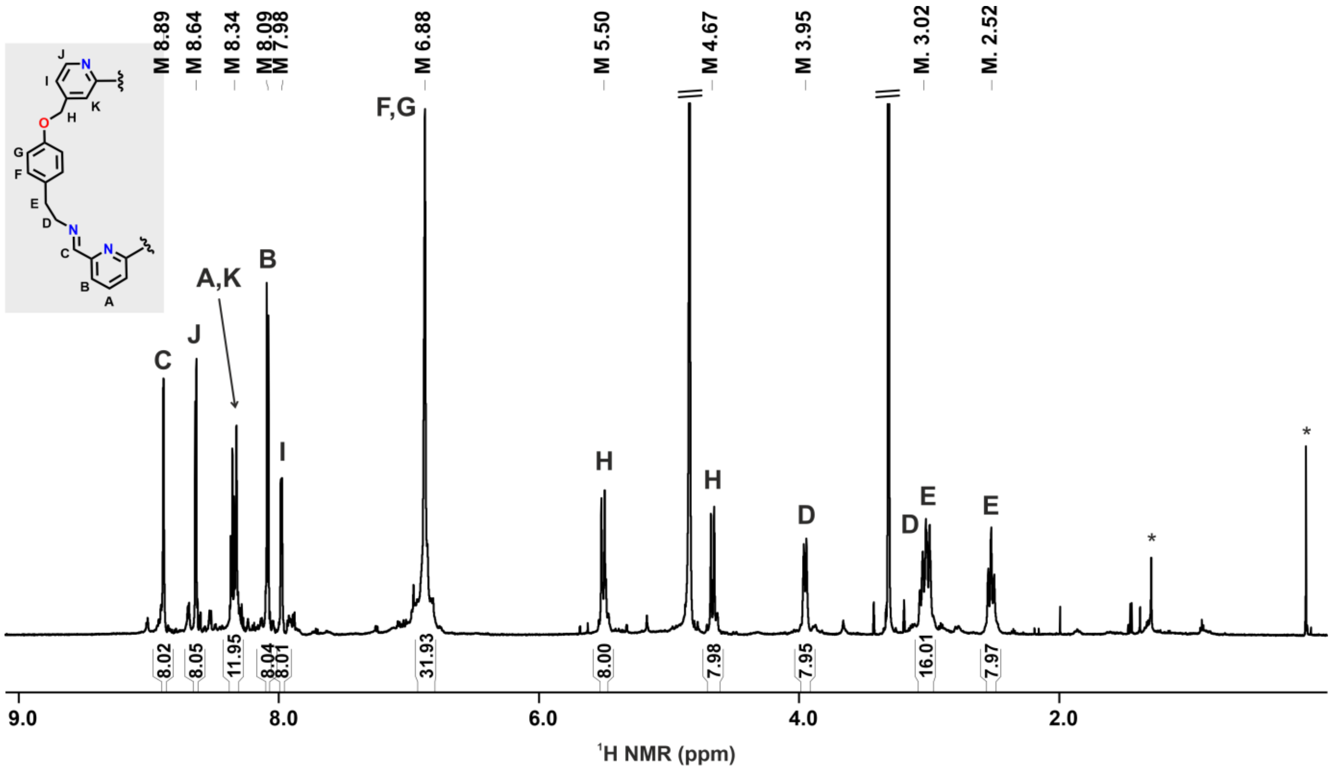

Figure S121. The ^{1}H NMR spectrum of **$(BF_4)_n \subset$ 10-4Ag$_2$** ([D$_4$]methanol, 300 K, 600 MHz). The signals corresponding to impurities were marked with asterisks.

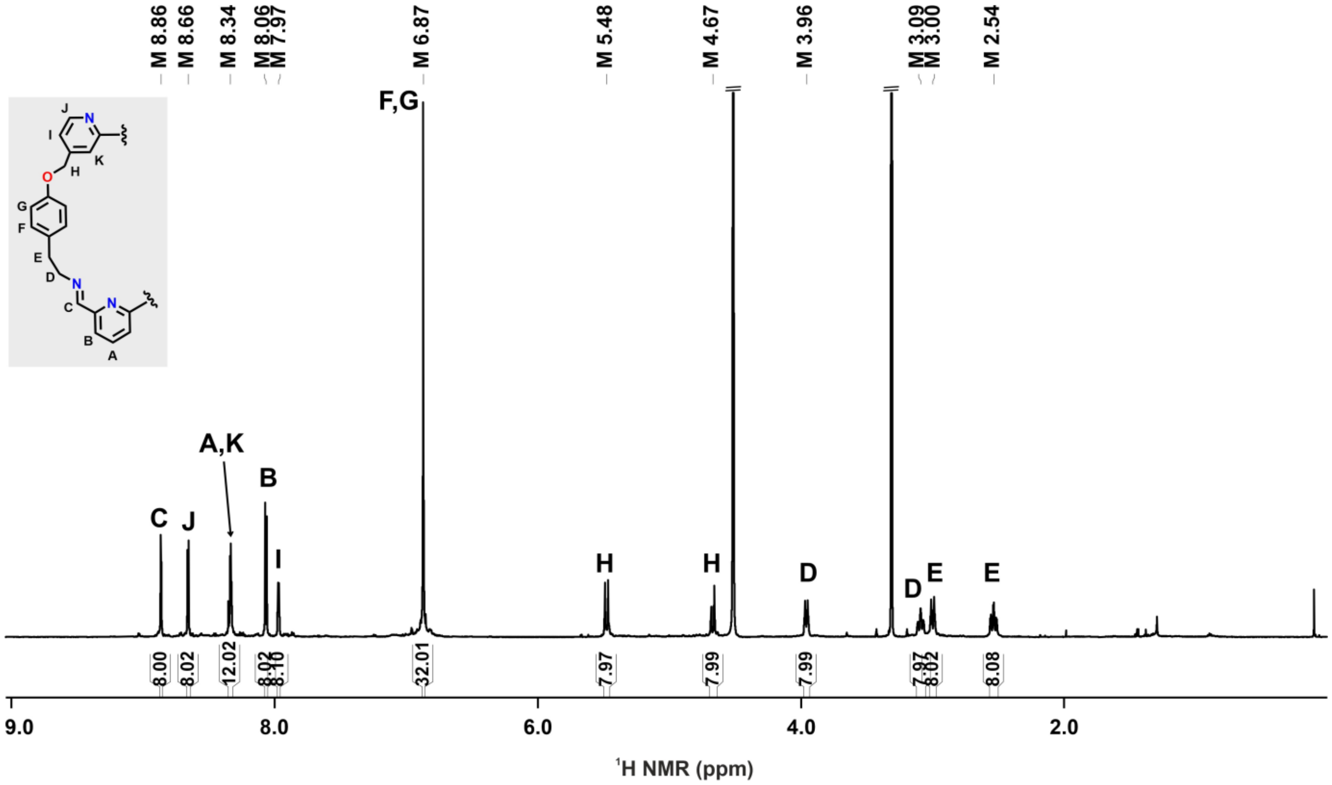

Figure S122. The ^{1}H NMR spectrum of **$(BF_4)_n \subset$ 10-4Ag$_2$** ([D$_4$]methanol, 330 K, 600 MHz).

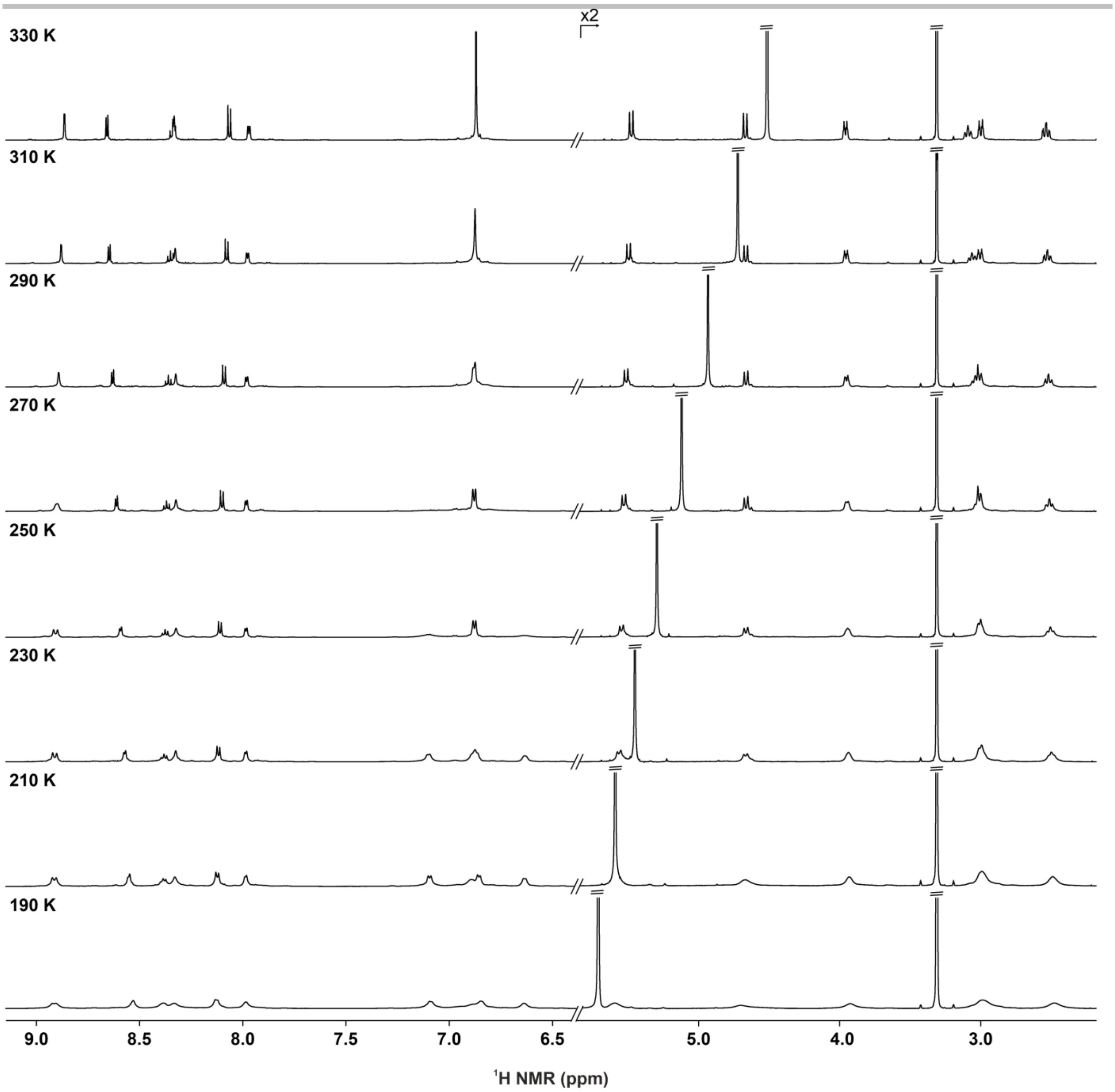

Figure S123. The ^{1}H NMR spectra of **$(BF_4)_n \subset$ 10-4Ag$_2$** recorded in the 330 K – 190 K temperature range ([D$_4$]methanol, 600 MHz).

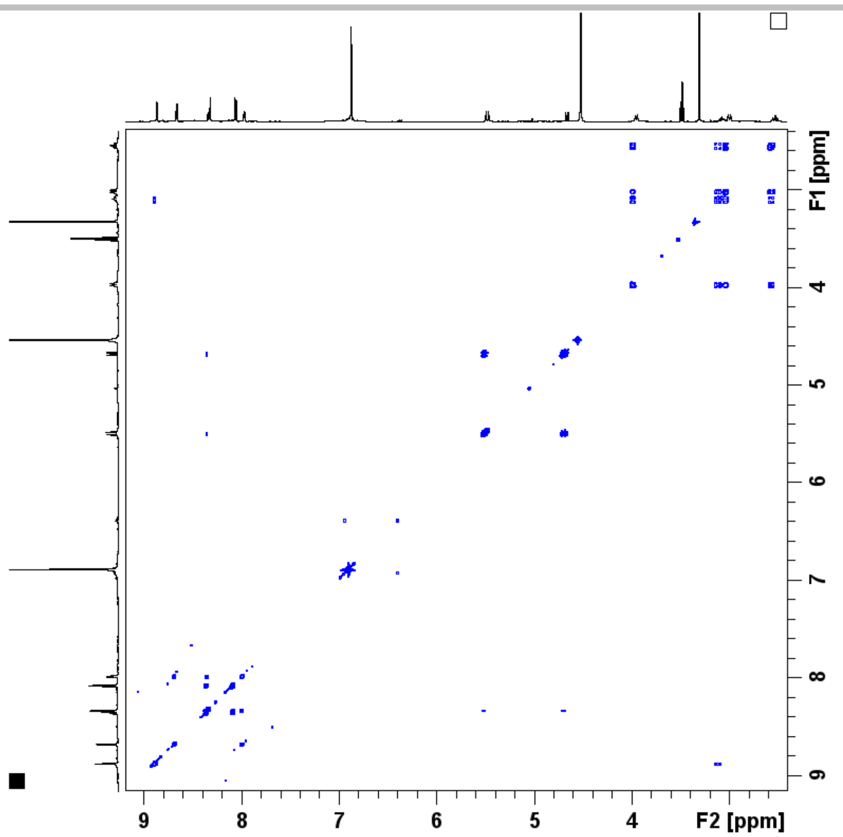

Figure S124. The ^{1}H-^{1}H COSY spectrum of **$(BF_4)_n \subset$ 10-4Ag$_2$** ([D$_4$]methanol, 330 K, 600 MHz).

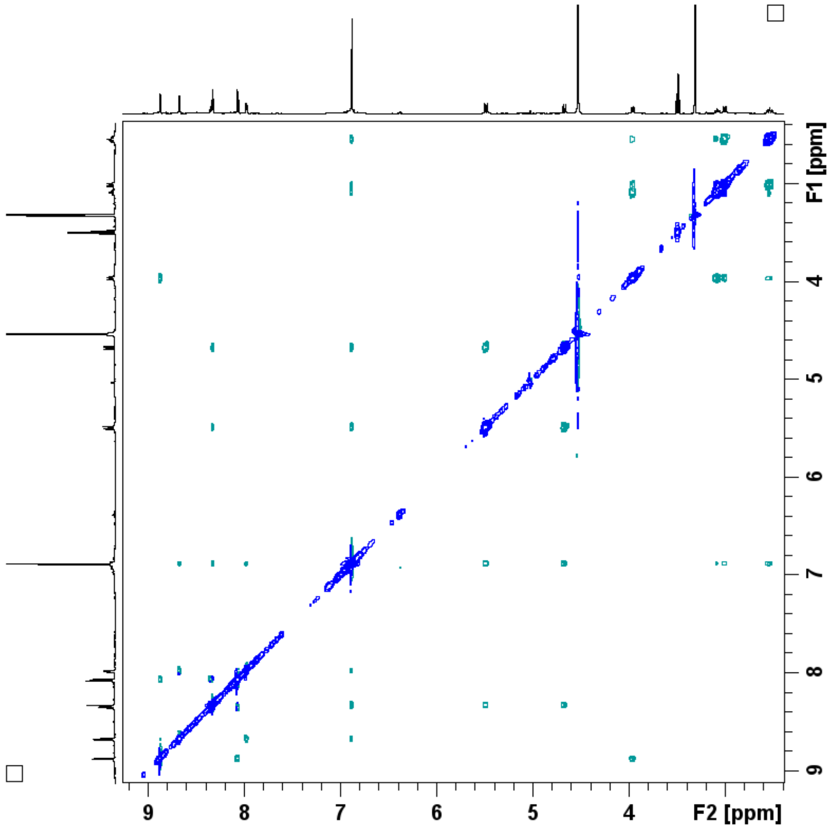

Figure S125. The ^{1}H-^{1}H ROESY spectrum of **$(BF_4)_n \subset$ 10-4Ag$_2$** ([D$_4$]methanol, 330 K, 600 MHz).

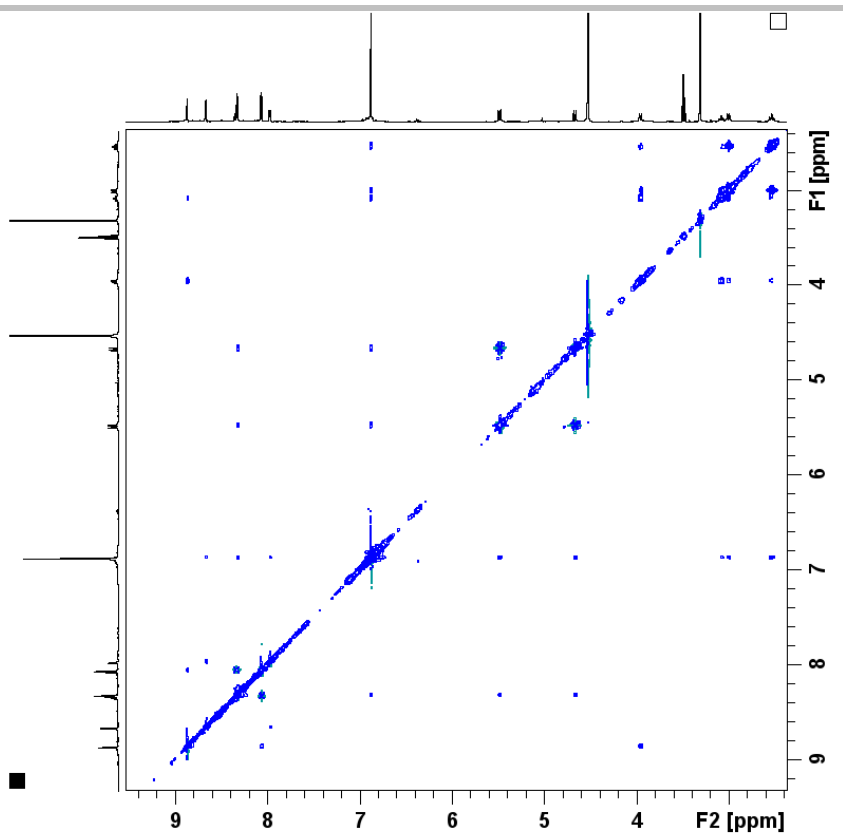

Figure S126. The ¹H-¹H NOESY spectrum of **$(BF_4)_n$ ⊂ 10-4Ag₂** ([D₄]methanol, 330 K, 600 MHz).

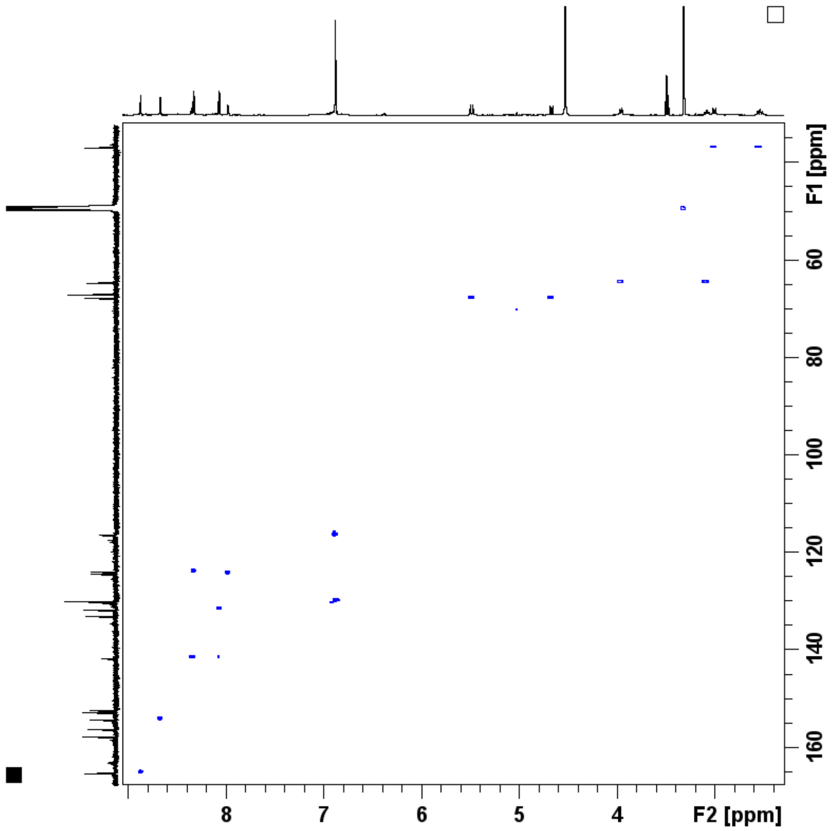

Figure S127. The ¹H-¹³C HSQC spectrum of **$(BF_4)_n$ ⊂ 10-4Ag₂** ([D₄]methanol, 330 K, 600 MHz).

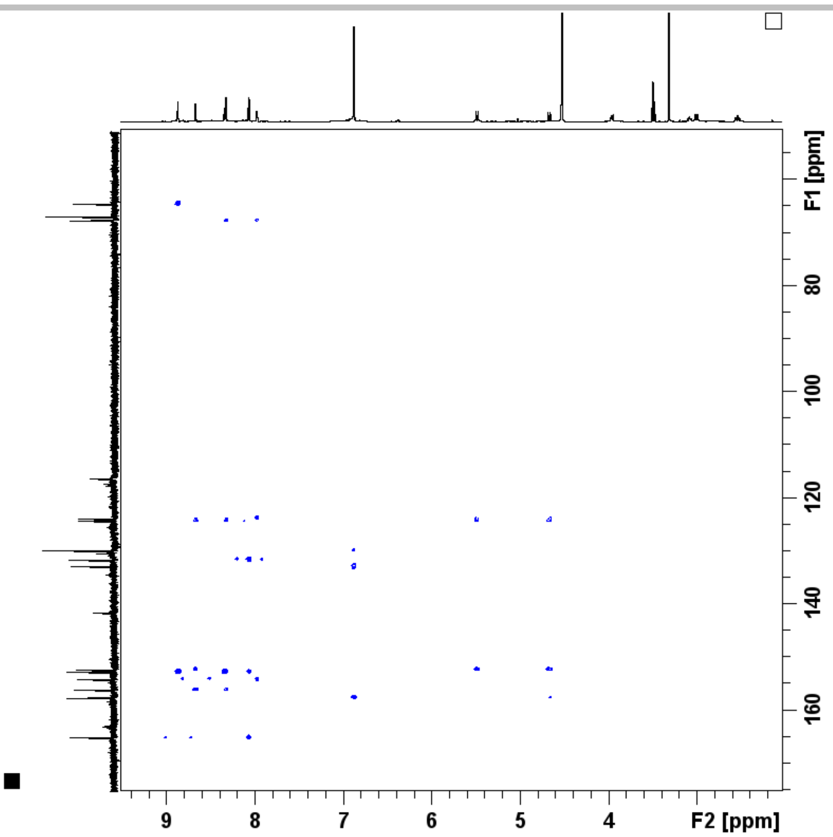

Figure S128. The ^{1}H-^{13}C HMBC spectrum of **$(BF_4)_n \subset$ 10-4Ag$_2$** ([D$_4$]methanol, 330 K, 600 MHz).

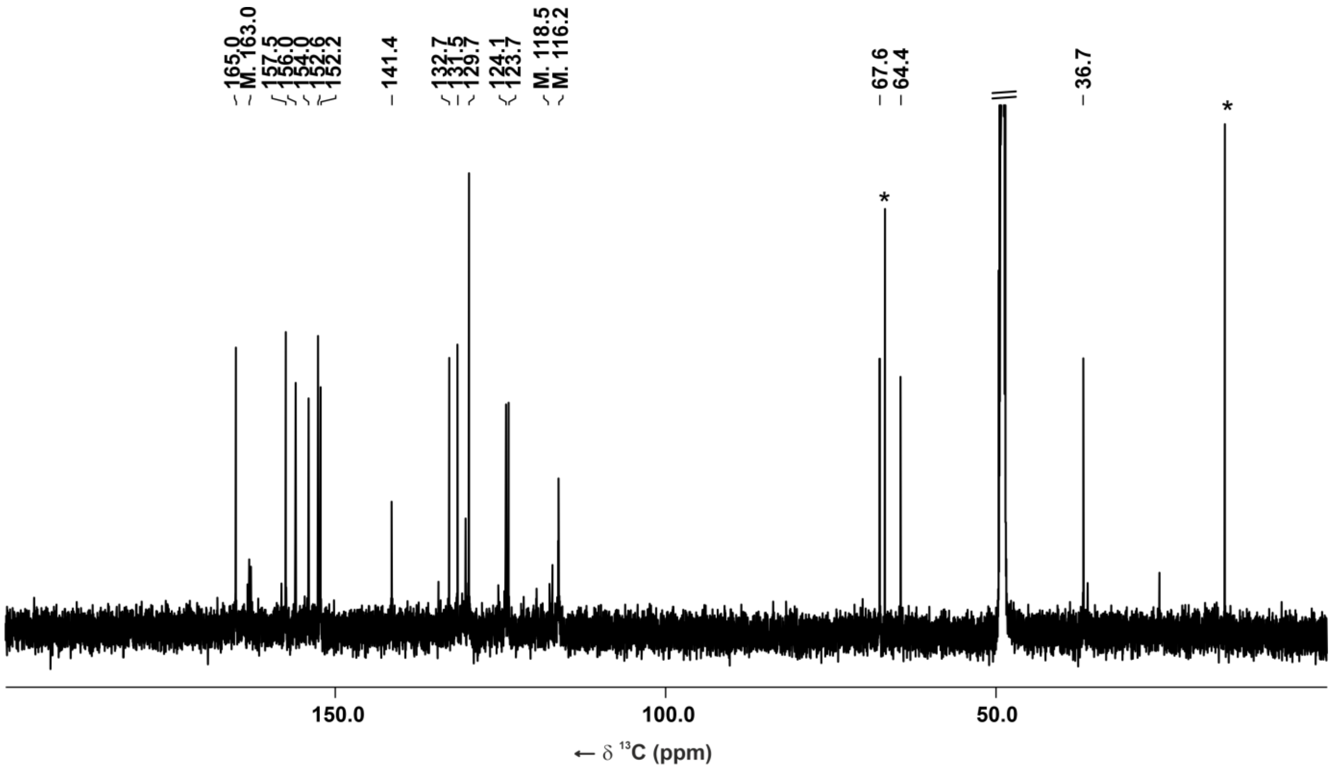

Figure S129. The ^{13}C NMR spectrum of **$(BF_4)_n \subset$ 10-4Ag$_2$** ([D$_4$]methanol, 330 K, 151 MHz). The signals corresponding to impurities were marked with asterisks.

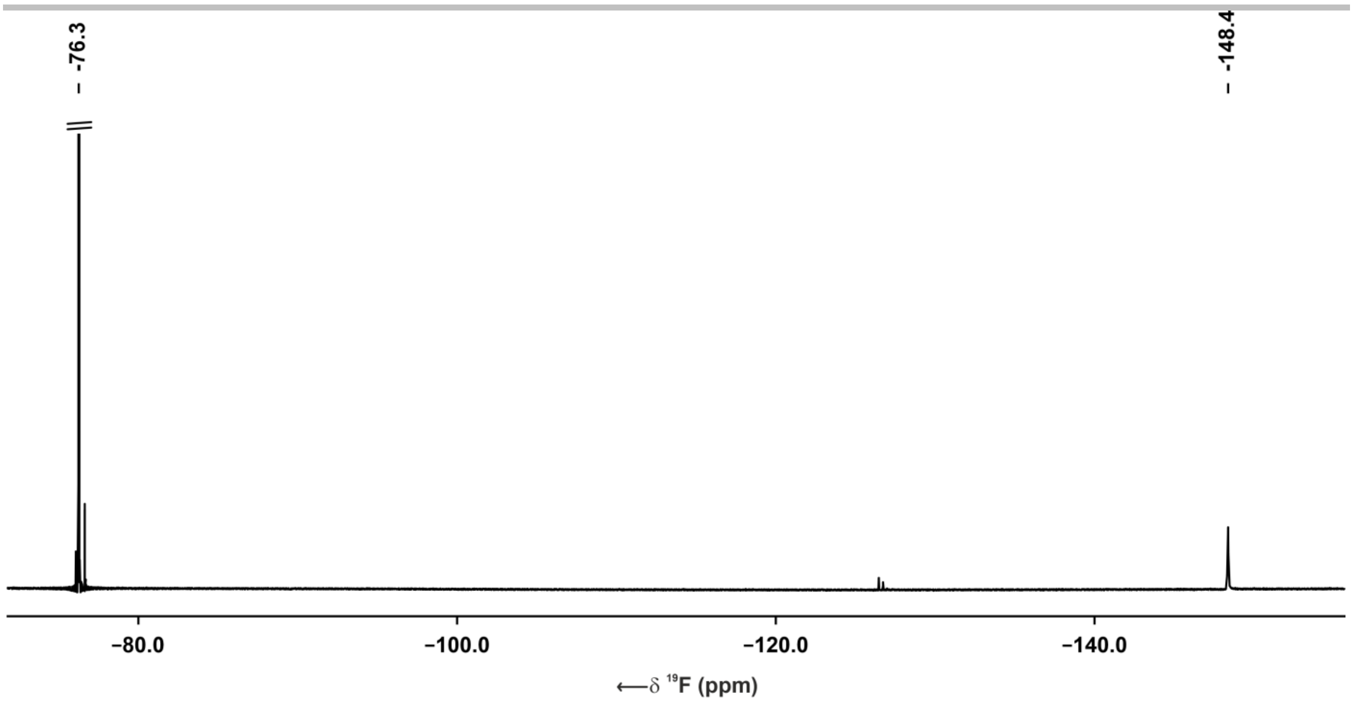

Figure S130. The ^{19}F NMR spectrum of **$(BF_4)_n \subset$ 10-4Ag$_2$** ([D$_4$]methanol, 230 K, 471 MHz).

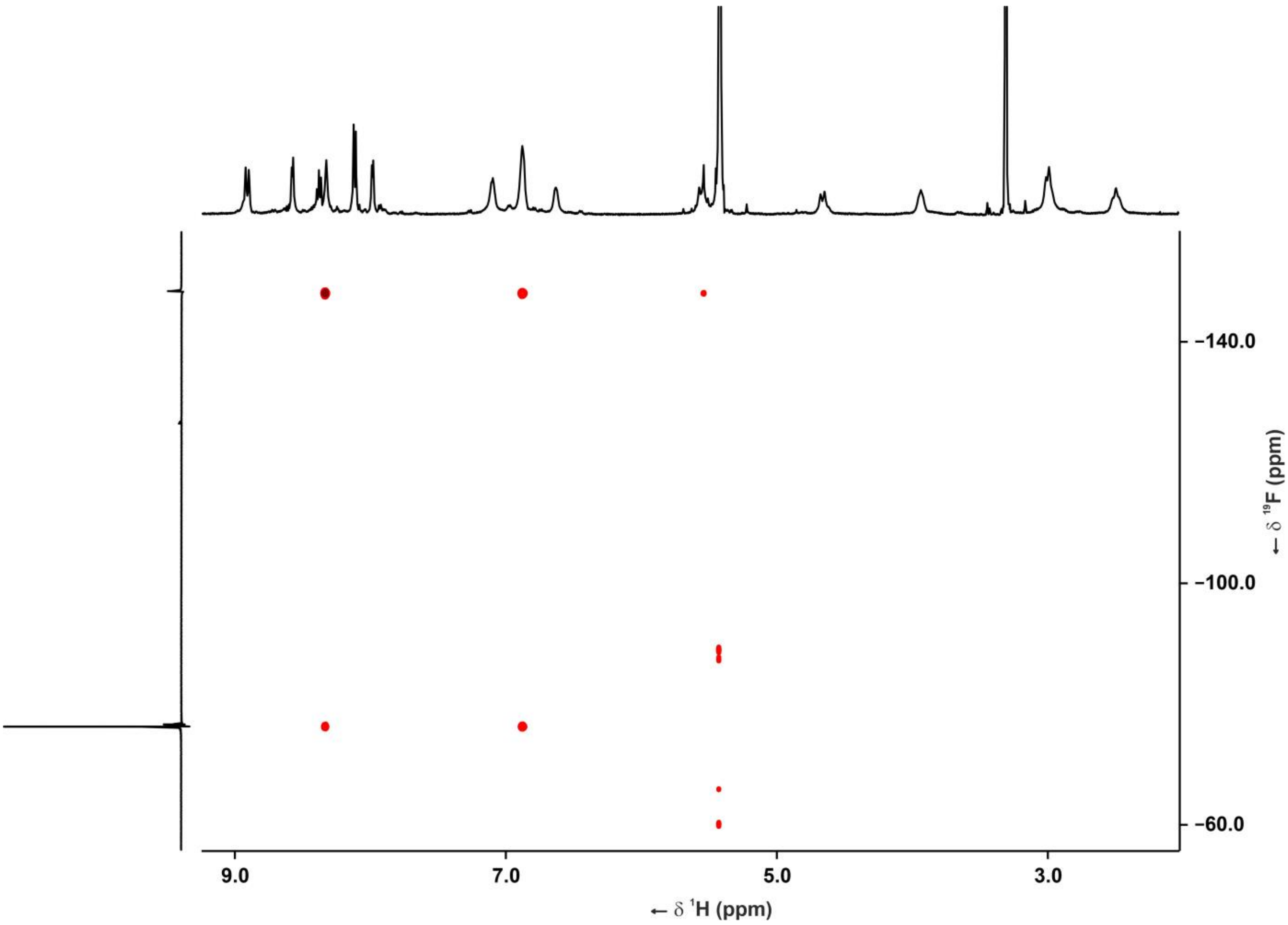

Figure S131. The ^{1}H-^{19}F HOESY spectrum of **$(BF_4)_n \subset$ 10-4Ag$_2$** ([D$_4$]methanol, 230 K, 500 MHz).

The structural model of 4-3Ag based on poor-quality X-ray data

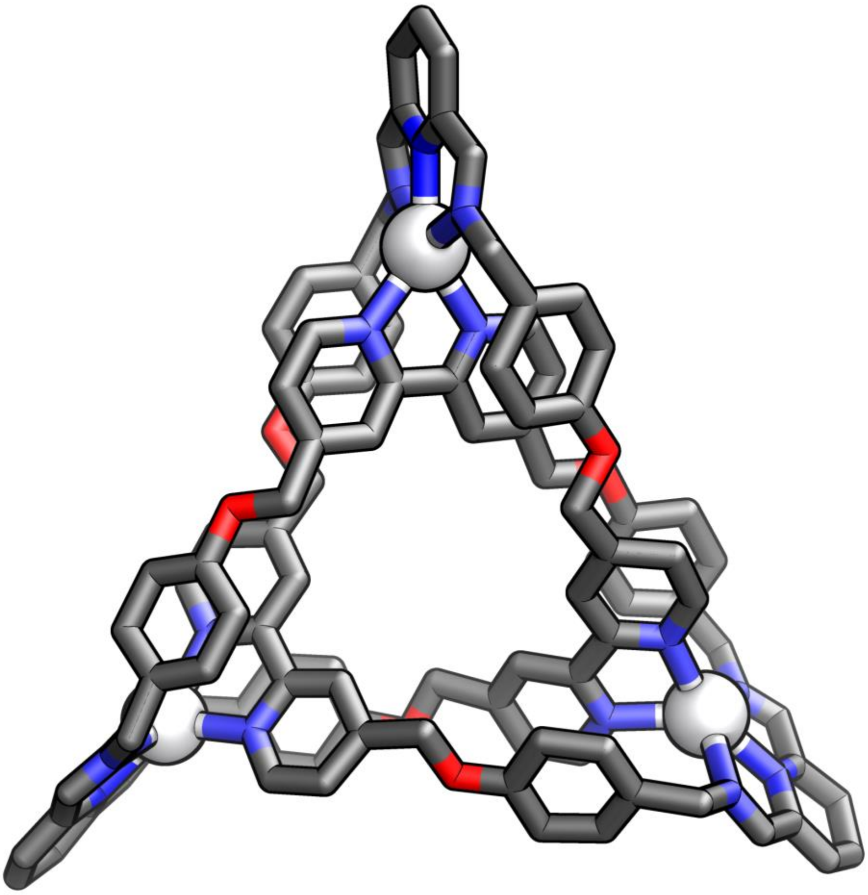

Figure S132. The structural model of **4-3Ag** based on poor-quality X-ray data.

Luminescence studies

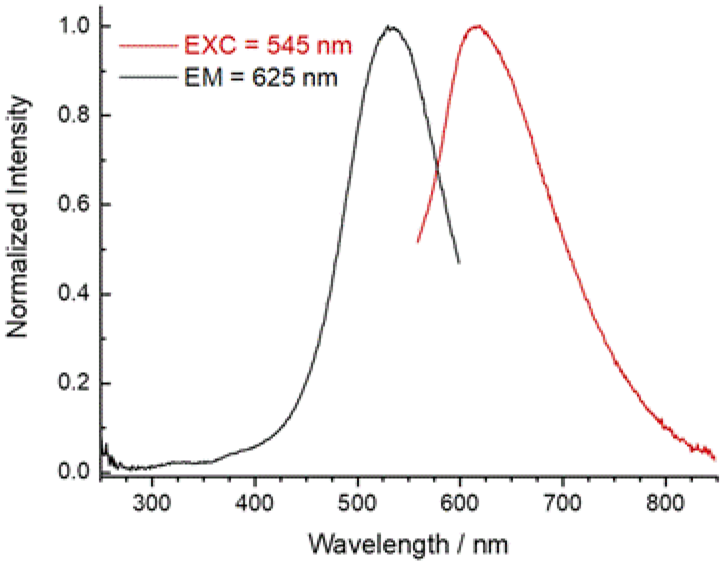

Figure S133. Room temperature photoluminescence upon excitation at the wavelength of 545 nm (red) and excitation monitoring 625 nm emission (black) spectra of the **9-2Ag₂**. Both spectra were taken with a 2 nm resolution.

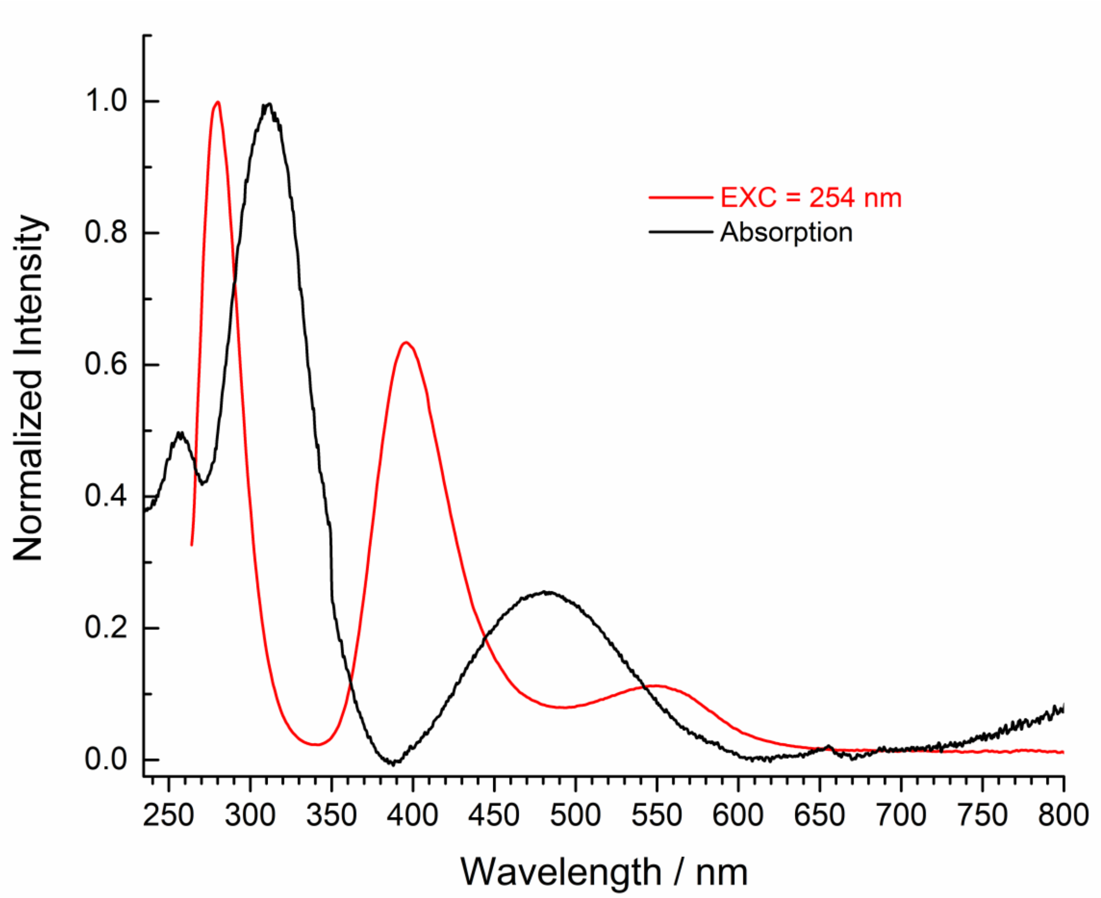

Figure S134. Room temperature photoluminescence upon excitation at the wavelength of 254 nm (red) and absorption (black) spectra of **$(PF_6)_2 \subset$ 10-4Ag$_2$**.

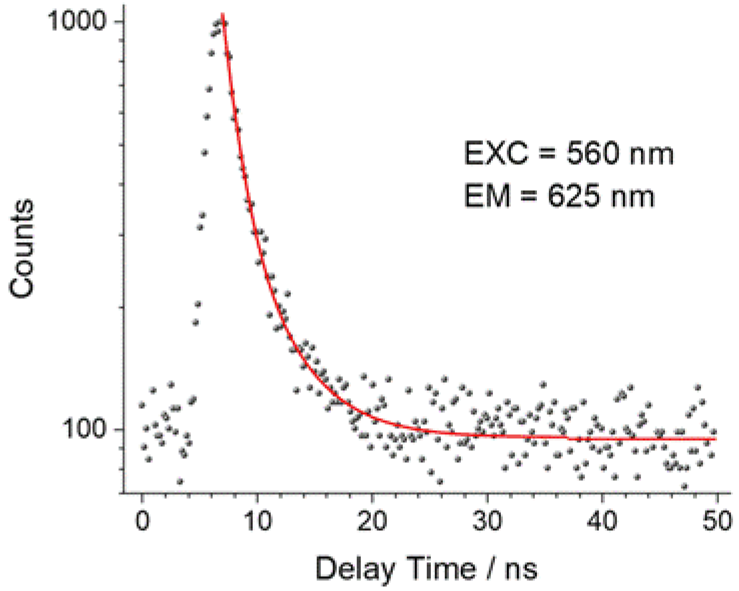

Figure S135. Room temperature decay kinetics of the 625 nm photoluminescence upon 560 nm excitation of the **9-2Ag$_2$**. The fit gave a decay time of 2.2 ns.

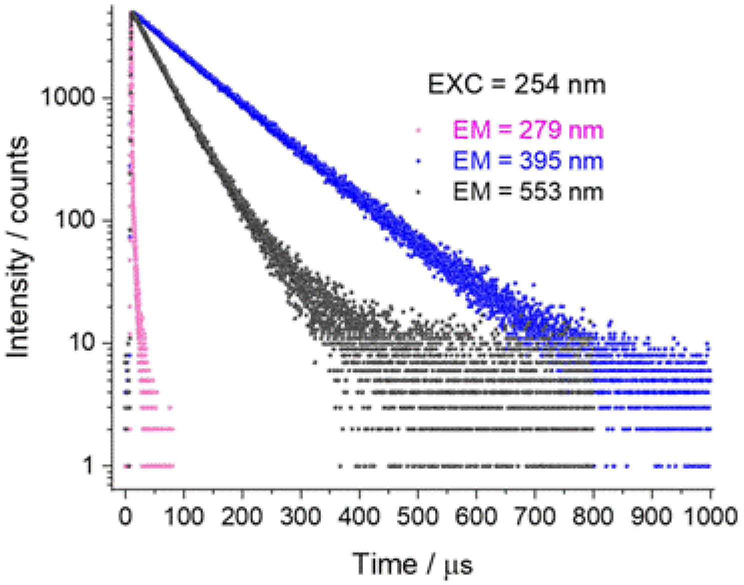

Figure S136. Room temperature decay kinetics of the 279, 395, and 553 nm photoluminescence upon 254 nm excitation of the **(PF$_6$)$_2$ ⊂ 10-4Ag$_2$**. The fits gave decay times of 115.9 ns and 110 and 47.5 µs, respectively.

Computational results

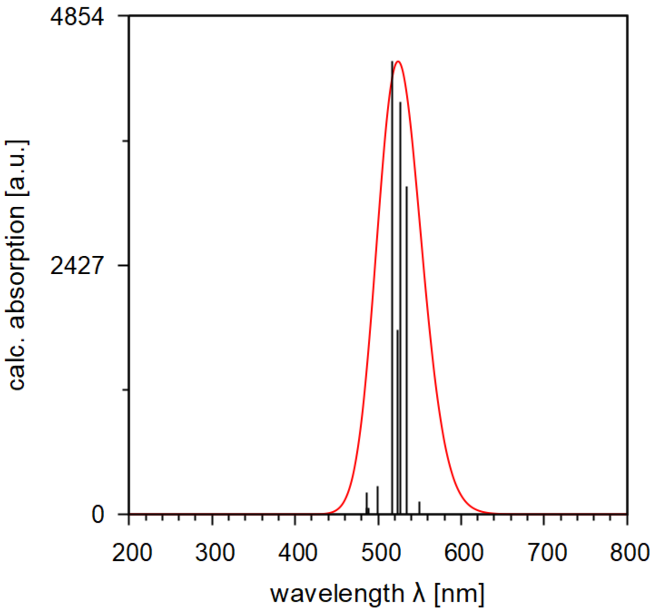

Figure S137. Simulated UV-VIS spectrum of **9-2Ag₂**.

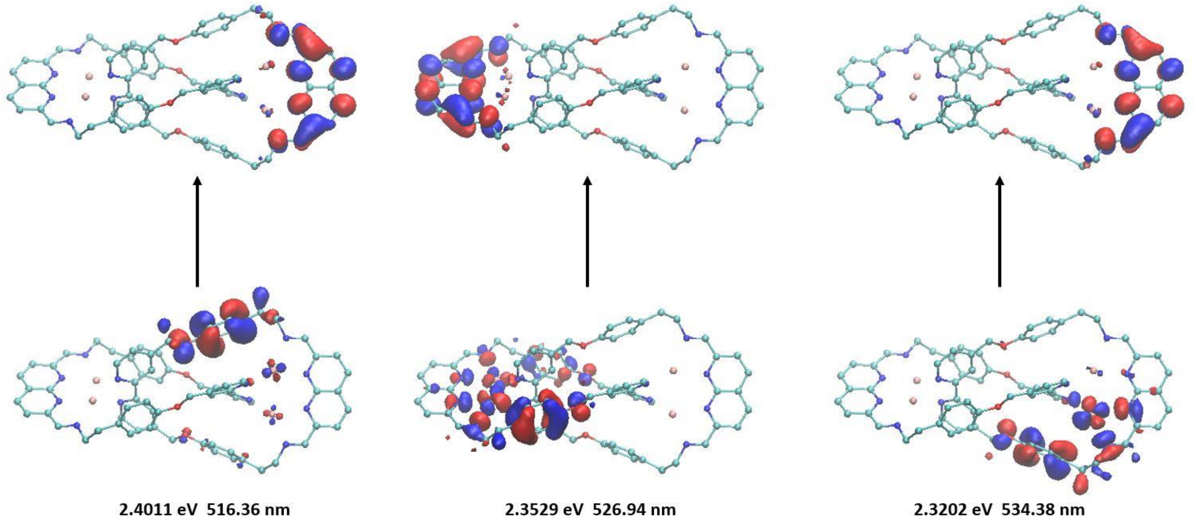

Figure S138. Major transitions contributing to the UV-VIS spectrum of **9-2Ag₂** based on the analysis of Natural Transition Orbitals.

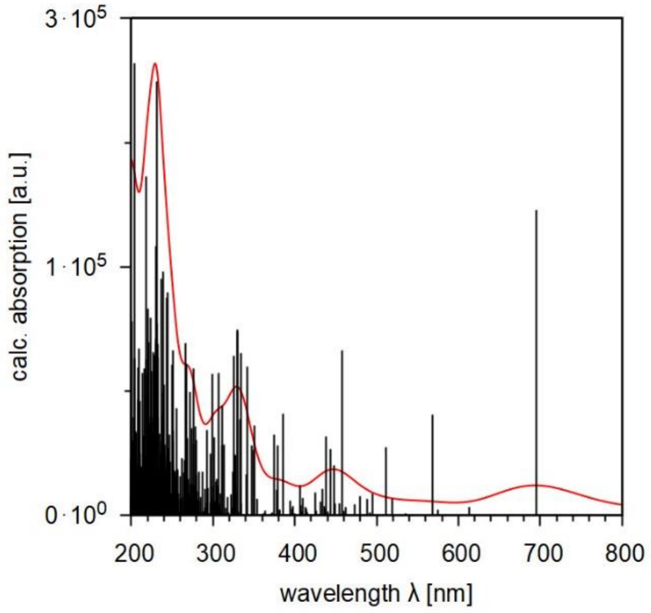

Figure S139. Simulated UV-VIS spectrum of **$(PF_6)_2 \subset$10-4Ag$_2$**.

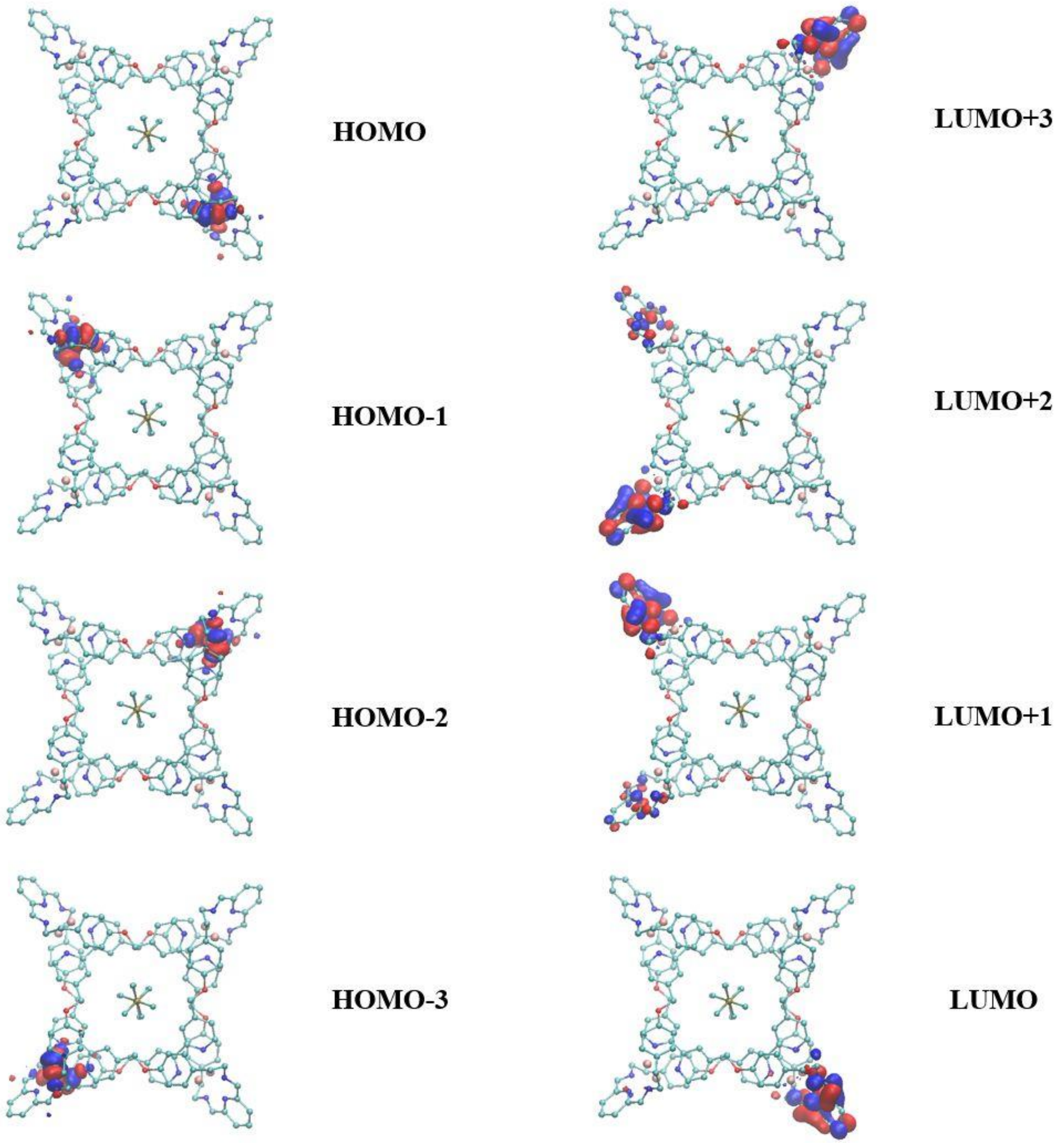

Figure S140. Frontier molecular orbitals of **$(PF_6)_2 \subset$10-4Ag$_2$**.

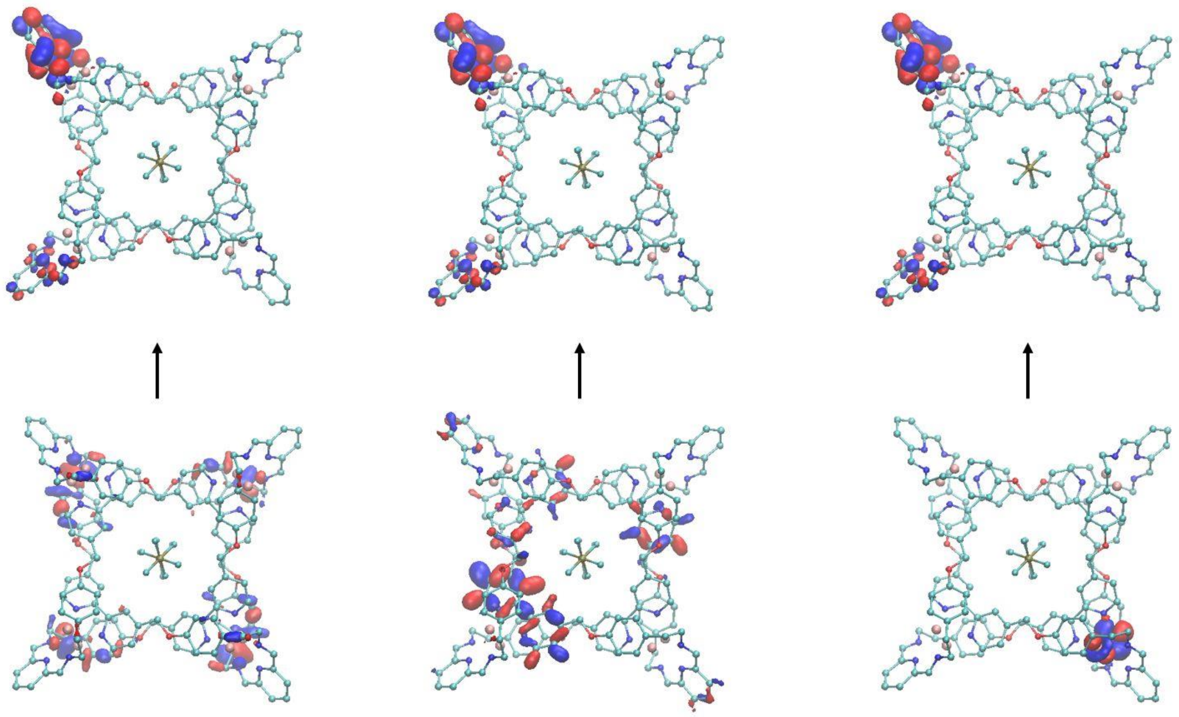

Figure S141. Major transitions contributing to the UV-VIS spectrum of **$(PF_6)_2$⊂10-4Ag$_2$** based on the analysis of GFN2-xTB molecular orbitals.

Cartesian coordinates

Table S2. Tight-binding-optimised Cartesian coordinates of **9-2Ag₂**.

Ag	-6.04960	1.20660	-0.98660
Ag	-6.04910	-1.22660	0.93850
Ag	6.14200	1.06000	1.07270
Ag	6.04940	-1.33590	-0.76670
O	-0.13790	-2.22380	2.14610
O	0.08650	-2.41550	-1.88910
O	-0.00780	2.55860	1.75630
O	-0.04040	2.19050	-2.05220
N	-6.92540	3.33010	-1.46510
N	-8.35560	1.11880	-0.46600
N	-8.37740	-1.04860	0.42310
N	-6.99620	-3.31030	1.38110
N	-4.35910	0.16440	1.43910
N	-4.36620	-0.19250	-1.47660
N	7.00600	3.09490	1.94040
N	8.36860	1.12110	0.45850
N	8.39590	-1.00620	-0.52450
N	7.03200	-3.34580	-1.27950
N	4.37430	0.00360	-1.34480
N	4.34950	-0.16700	1.57510
C	-8.20940	3.37710	-1.33220
H	-8.77500	4.28940	-1.54680
C	-9.00710	2.22180	-0.86990
C	-10.42690	2.32830	-0.85780
H	-10.90200	3.24610	-1.18850
C	-11.17900	1.24800	-0.43220
H	-12.26420	1.29230	-0.42470
C	-10.51450	0.06730	0.00160
C	-9.07480	0.04580	-0.01270
C	-11.20490	-1.09420	0.44660
H	-12.29090	-1.10620	0.46180
C	-10.47660	-2.19850	0.85090
H	-10.97160	-3.10390	1.18640
C	-9.05420	-2.13420	0.83310
C	-8.28270	-3.31850	1.26530
H	-8.87000	-4.21910	1.47120
C	-6.32190	-4.57450	1.75700
H	-5.76790	-4.91280	0.87340
H	-7.07200	-5.34760	1.97710
C	-5.37790	-4.43990	2.96410
H	-5.19810	-5.46450	3.32200
H	-5.90580	-3.93040	3.77950
C	-4.02890	-3.78280	2.72290
C	-3.31170	-3.95110	1.51940
H	-3.73750	-4.51840	0.69610
C	-2.01980	-3.43830	1.36500
H	-1.46130	-3.59060	0.44710
C	-1.41500	-2.74310	2.42250
C	-2.09650	-2.57010	3.63720
H	-1.64130	-2.06450	4.48340
C	-3.39600	-3.08740	3.77060
H	-3.91010	-2.96850	4.72190
C	0.76980	-1.94420	3.23690
H	0.99080	-2.86580	3.79390
H	0.33000	-1.22230	3.94240
C	2.02790	-1.34900	2.65000
C	2.06520	-0.89230	1.32450
H	1.19640	-1.03120	0.69580
C	3.23060	-0.30450	0.80660
C	4.32420	-0.63130	2.85380
H	5.23460	-0.51880	3.43170
C	3.19420	-1.22070	3.42430
H	3.23950	-1.57030	4.45120
C	8.25530	3.23760	1.64450
H	8.83940	4.09080	2.00510
C	9.00780	2.23370	0.85770
C	10.39420	2.44950	0.61370
H	10.86060	3.37310	0.94000
C	11.13070	1.46340	-0.01890
H	12.19290	1.59470	-0.20390
C	10.48640	0.25590	-0.40850
C	9.07340	0.12740	-0.16890
C	11.17340	-0.84140	-1.00000
H	12.24040	-0.77170	-1.19060
C	10.47180	-1.99450	-1.30740
H	10.97220	-2.85600	-1.73740
C	9.07090	-2.03920	-1.05680
C	8.32010	-3.28050	-1.35600
H	8.92430	-4.15270	-1.62530
C	6.36110	-4.64360	-1.51660
H	5.86420	-4.91880	-0.57900
H	7.11040	-5.42110	-1.72310
C	5.34680	-4.61990	-2.67740
H	5.82690	-4.18460	-3.56250
H	5.16310	-5.67490	-2.92980
C	3.99760	-3.95690	-2.44130
C	3.32620	-4.00810	-1.20090
H	3.79300	-4.47830	-0.33910
C	2.02800	-3.50470	-1.05390
H	1.50700	-3.57100	-0.10420
C	1.36740	-2.93720	-2.15470
C	2.00520	-2.88200	-3.40430
H	1.51170	-2.48000	-4.28380
C	3.31070	-3.38640	-3.53050
H	3.78680	-3.35910	-4.50820
C	-0.85580	-2.24670	-2.97560
H	-1.10590	-3.22310	-3.41430
H	-0.42880	-1.62070	-3.77400
C	-2.09110	-1.56210	-2.43470
C	-3.25740	-1.46110	-3.21510
H	-3.32000	-1.90120	-4.20590
C	-4.36300	-0.77380	-2.70580
H	-5.26710	-0.67360	-3.29550
C	-3.25150	-0.31050	-0.69990
C	-2.10800	-0.98460	-1.15670
H	-1.24350	-1.09000	-0.51540
C	-3.25420	0.30860	0.65200
C	-2.12590	1.01510	1.09530
H	-1.27280	1.14530	0.44330
C	-2.11220	1.60080	2.36990
C	-3.26440	1.46460	3.16490
H	-3.32970	1.90590	4.15500
C	-4.35610	0.74460	2.66860
H	-5.24990	0.61970	3.26900

C	-0.89160	2.33690	2.87890
H	-1.17320	3.29470	3.33940
H	-0.39900	1.72440	3.65050
C	1.26420	3.12550	1.95580
C	1.90050	3.24950	3.20160
H	1.40870	2.96620	4.12730
C	3.19500	3.79450	3.25620
H	3.65940	3.92200	4.23210
C	3.87270	4.21360	2.09410
C	3.19980	4.09110	0.86140
H	3.67750	4.44800	-0.04820
C	1.90770	3.55930	0.78650
H	1.38050	3.49250	-0.15920
C	5.22720	4.89380	2.17210
H	5.54660	5.21920	1.17520
H	5.12260	5.81050	2.76900
C	6.37370	4.09270	2.83400
H	7.13270	4.79940	3.20050
H	5.98820	3.54250	3.70040
C	-6.22240	4.56860	-1.87400
H	-5.67650	4.92700	-0.99330
H	-6.95550	5.34680	-2.13100
C	-5.26160	4.37760	-3.06020
H	-5.77800	3.83040	-3.85830
H	-5.07590	5.38420	-3.46340
C	-3.91600	3.73350	-2.76880
C	-3.21600	3.95990	-1.56490
H	-3.65240	4.56700	-0.77650
C	-1.92830	3.45250	-1.36490
H	-1.38620	3.64400	-0.44470
C	-1.30950	2.70420	-2.37710
C	-1.97150	2.47600	-3.59350
H	-1.50350	1.93270	-4.40890
C	-3.26750	2.98880	-3.77230
H	-3.76640	2.82640	-4.72540
C	0.88200	1.84340	-3.11070
H	1.13790	2.73570	-3.69920
H	0.43600	1.10570	-3.79570
C	2.11280	1.23620	-2.47890
C	2.10760	0.79200	-1.14820
H	1.22760	0.95470	-0.54130
C	3.24730	0.18160	-0.60060
C	4.39430	0.45970	-2.62460
H	5.31300	0.31270	-3.18090
C	3.29430	1.07740	-3.22330
H	3.37150	1.41940	-4.25080

Table S3. Tight-binding-optimised Cartesian coordinates of **$(PF_6)_2$⊂10-4Ag_2**.

Ag	22.89960	2.46430	8.24750
Ag	24.46360	0.18370	8.38030
Ag	14.61550	-2.03490	13.55560
Ag	12.56610	-0.82900	12.13570
Ag	11.72620	-8.99780	5.20330
Ag	11.12780	-7.46940	2.97290
Ag	21.34070	-4.03960	-1.03860
Ag	21.45960	-6.64340	-0.10450
O	13.45170	-6.82930	8.48440
O	18.58740	1.69550	9.08440
N	24.04240	3.76900	6.72090
N	15.18150	-0.71460	11.70800
N	21.96580	0.48200	9.08020
O	18.49770	-1.94650	11.59690
N	13.52230	-2.80280	11.30530
O	11.92850	-2.59650	8.17160
O	23.38700	-3.00610	5.69190
C	15.28290	-1.37620	10.54490
C	19.40400	2.39760	8.25030
N	11.80060	-6.66480	5.39370
C	13.56940	-8.01310	7.82260
C	21.08020	4.14170	8.11700
C	20.02260	-1.32730	9.90380
C	16.68040	0.95050	10.88420
H	17.23910	1.85450	11.05670
C	16.04330	-0.87870	9.49440
H	16.07470	-1.39630	8.55240
N	22.98270	0.40740	6.57480
N	25.17090	0.43720	10.57820
C	13.09240	-4.96760	10.40460
H	12.49050	-5.86000	10.39670
C	23.42970	0.53210	5.32780
H	24.41820	0.95800	5.22040
C	20.64110	-0.48030	10.81570
H	20.37360	-0.51690	11.85780
C	20.97330	-0.53610	5.71250
H	19.99390	-0.94490	5.88890
C	14.18140	-4.85190	9.54880
C	20.37550	-1.21570	8.56340
H	19.90400	-1.85900	7.84050
C	16.76450	0.29930	9.65960
C	20.76230	-1.29150	0.92520
C	21.14120	3.99340	6.72010
C	12.43610	-8.83440	7.86950
H	11.60370	-8.52640	8.48440
N	13.35270	-3.55970	14.75590
C	22.70130	0.14360	4.21780
H	23.10030	0.25210	3.22370
N	8.93020	-6.86590	3.34410
C	15.32380	-6.75270	4.40270
H	15.78210	-6.04390	5.07410
C	13.34550	-4.87980	5.70950
H	14.30770	-4.44220	5.50840
N	12.47510	-0.84720	14.87720
N	12.60720	1.31110	13.00850
C	17.69400	-2.78090	12.31230
C	22.03370	4.87590	5.89050
H	22.26290	5.79070	6.43740
H	21.53140	5.14830	4.96260
N	13.37470	-7.82870	3.55400
C	18.91180	-1.84550	-0.50810
H	17.85100	-2.00820	-0.62420
C	12.75450	-2.83800	7.05380
H	13.81600	-2.79390	7.32500
H	12.57980	-2.08030	6.27520
C	14.57100	-5.98740	8.64620
H	14.92900	-5.60130	7.68370
H	15.40920	-6.53340	9.10540
C	14.54980	-2.65550	10.45560
C	26.14890	2.56690	10.10000
C	15.86830	0.41350	11.86640
H	15.76840	0.89740	12.82860

C	21.43340	-0.39180	4.40770
C	23.79400	-2.78170	6.97170
C	14.14680	-8.55210	2.74990
H	13.63460	-9.24490	2.09610
N	25.52620	2.73800	8.92710
C	14.90800	-3.66490	9.56690
H	15.75440	-3.55190	8.90860
C	21.60090	0.40260	10.35640
H	22.09810	1.08250	11.03460
C	22.05090	-3.92890	3.96960
O	21.36160	-0.98170	2.10850
N	19.48420	-5.43820	0.23540
C	13.56750	-11.83910	5.82630
H	12.82470	-12.49460	6.28200
H	14.54510	-12.31160	5.92260
C	19.01600	-2.34890	10.34930
H	18.22540	-2.45180	9.59470
H	19.51570	-3.32680	10.42720
C	24.88560	-0.64990	11.47260
H	23.79820	-0.73130	11.56580
H	25.31990	-0.48670	12.46630
C	25.42200	-1.97150	10.88530
H	26.50970	-1.91850	10.82860
H	25.15420	-2.77650	11.56970
C	12.89020	-0.37610	8.51600
H	13.62730	-0.49300	7.73720
C	25.99010	3.68620	8.10360
N	23.75610	-6.92080	-0.21550
C	20.56200	-0.78770	3.25310
H	20.00930	-1.69960	3.50730
H	19.82690	0.01290	3.07890
C	13.38400	-5.13430	1.65130
C	17.00770	-4.81670	1.33630
C	14.67930	-9.67320	6.48480
H	15.57470	-10.00140	5.97820
C	15.18350	-5.17320	14.88050
H	15.38950	-4.93200	15.92390
H	15.39820	-6.23060	14.72570
C	21.77440	-0.14120	6.77620
C	22.58320	-6.19150	-4.98840
H	22.06810	-6.08270	-5.92930
C	20.22260	3.34670	8.87230
H	20.10720	3.50820	9.93350
N	21.76420	-4.39720	1.24540
C	16.13370	-7.53050	3.57980
C	23.58810	-2.80070	9.36700
H	23.01460	-3.06940	10.24150
C	14.69690	-8.44650	7.13580
H	15.60080	-7.85740	7.12100
C	12.42610	-4.19130	6.49520
N	20.22510	-4.34190	-3.04780
N	11.99360	-11.03700	4.13850
C	17.17820	-3.98940	11.86050
H	17.42900	-4.36670	10.88060
C	16.35650	-4.74180	12.68960
H	15.98670	-5.69010	12.32920
C	23.06070	-3.08200	8.11360
H	22.09360	-3.55490	8.04260
C	19.40700	-1.55170	0.75510
H	18.72230	-1.50710	1.58750
C	27.73550	4.30640	9.63050
H	28.58750	4.90940	9.90140
C	10.92690	-6.01430	6.15760
H	9.97780	-6.50830	6.31660
C	13.94310	-6.91680	4.35790
C	20.69900	-4.47380	2.05760
C	12.80470	-3.92090	11.26010
H	11.96200	-3.97190	11.93600
C	25.74590	1.48310	10.99750
H	26.03620	1.60650	12.04440
C	18.18790	-4.46680	1.98470
H	18.14560	-3.93180	2.91900
C	23.13470	-3.81030	3.10860
H	24.10190	-3.53080	3.48960
O	18.17930	-7.94140	2.45500
C	27.11250	4.46400	8.40500
H	27.46680	5.19840	7.69960
O	14.70570	-5.33960	1.39480
C	25.28040	3.99090	6.86000
H	25.87340	4.48950	6.08880
C	12.37350	-3.20240	15.47300
H	11.74140	-3.92540	15.99560
C	17.59890	0.84930	8.54030
H	18.04870	0.02460	7.97420
H	16.94830	1.40960	7.85210
C	11.19270	-4.78540	6.73260
H	10.46450	-4.29190	7.35320
C	20.81400	-4.26130	3.42640
H	19.96290	-4.36230	4.07740
C	19.17360	-2.14330	-2.99700
H	18.20460	-1.65730	-3.10870
H	19.84540	-1.74800	-3.75910
C	19.46420	2.24500	6.86970
H	18.82950	1.53700	6.36080
C	11.81970	2.35220	10.94470
H	12.04440	3.17460	10.26590
H	10.81810	2.50430	11.34730
C	22.19600	-3.71910	5.44800
H	21.31970	-3.18200	5.83300
H	22.21720	-4.70070	5.94590
C	23.35400	4.16900	5.52550
H	23.11630	3.26280	4.95880
H	23.96870	4.82880	4.90220
C	25.59300	-1.95030	8.36350
H	26.63070	-1.64900	8.45160
C	21.61180	-1.32770	-0.18660
H	22.64470	-1.04360	-0.05270
C	20.33210	3.03170	6.12520
H	20.33850	2.92330	5.05120
C	19.73490	-1.86950	-1.62840
C	17.39350	-2.34810	13.60930
H	17.87370	-1.45330	13.97580
C	13.25530	-11.69820	4.32210
H	14.03030	-11.07300	3.86730
H	13.27090	-12.68790	3.84960
C	12.43760	-10.07300	7.23600
H	11.61610	-10.75890	7.40950
C	15.67090	-4.45060	1.91220
H	15.71180	-4.49350	3.00800
H	15.43720	-3.41350	1.62900
C	22.28360	-8.34890	1.84070
C	12.84060	0.81540	9.22690

H	13.53680	1.60050	8.97350
C	18.35560	-5.78780	-0.37640
H	18.46900	-6.32270	-1.30970
C	13.01010	-6.12180	5.18370
C	20.46390	-7.53850	3.22790
H	20.15310	-7.06470	4.14640
C	19.41120	-4.80150	1.41420
C	27.23320	3.35360	10.50010
H	27.68850	3.19670	11.46480
C	24.85210	-2.24320	9.52020
C	17.62720	-7.40140	3.63450
H	17.90690	-6.34860	3.75450
H	17.99500	-7.93830	4.52280
C	19.53110	-8.02340	2.31850
C	11.97850	-1.38760	8.79570
C	23.75580	-8.54660	1.60370
H	23.91200	-9.38280	0.92130
H	24.25780	-8.77470	2.54410
C	15.52410	-8.43720	2.72180
H	16.12020	-9.03470	2.05370
C	12.83670	2.40210	12.10270
H	13.84160	2.28110	11.68490
H	12.77530	3.37260	12.60870
C	7.42800	-11.33480	2.90390
H	6.60050	-11.98610	2.67130
C	11.88240	1.04180	10.20840
C	10.95590	0.02020	10.48210
H	10.10810	0.22160	11.12880
C	11.38930	-1.49800	16.91790
H	11.05580	-2.29440	17.56370
C	16.04980	-4.33350	13.98220
C	18.97860	-3.65400	-3.23690
H	18.27200	-4.03350	-2.49210
H	18.56750	-3.82040	-4.23940
C	11.63830	0.80660	16.40420
H	11.49720	1.85010	16.63590
C	21.34890	-0.29920	8.18100
C	21.95870	-5.80600	-3.79810
C	13.68290	-4.94900	14.60270
H	13.48030	-5.22840	13.56400
H	13.08740	-5.58630	15.26750
C	16.58860	-3.11990	14.44040
H	16.49450	-2.85040	15.48660
C	25.06420	-2.20790	7.10300
H	25.65540	-2.05970	6.21190
C	13.56670	-10.50510	6.52090
C	11.18650	-0.17450	17.26920
H	10.68990	0.08440	18.19080
C	11.00970	-1.18420	9.78540
H	10.25440	-1.94240	9.92880
C	9.12150	-4.72320	2.18530
H	8.87050	-3.66880	2.29870
H	8.60200	-5.10620	1.30690
C	11.50090	-4.08020	2.71110
H	11.12190	-3.31930	3.37620
C	12.57420	1.50230	14.25880
H	12.71500	2.49210	14.70110
C	22.94170	-4.06070	1.76190
H	23.76180	-3.97990	1.06160
C	12.50290	-5.95820	0.94220
H	12.91030	-6.62670	0.19890
C	17.10180	-5.50480	0.13220
H	16.20960	-5.80980	-0.38740
C	12.24670	0.43130	15.20230
C	12.06610	-1.79460	15.73110
C	21.82050	-7.69520	2.97660
H	22.52540	-7.34010	3.71350
C	10.60730	-4.88390	2.01270
C	19.97660	-8.66940	1.15900
H	19.24150	-9.11100	0.50320
C	11.10480	-11.53640	3.38970
H	11.25960	-12.47520	2.85100
C	24.41370	-7.28540	1.00790
H	24.28450	-6.46110	1.71660
H	25.48520	-7.46530	0.85860
C	12.87300	-4.18850	2.53210
H	13.52910	-3.51860	3.06530
N	9.57750	-9.64650	3.50930
C	9.77100	-10.94730	3.25690
N	22.53830	-5.94710	-2.59980
C	21.10490	-1.60800	-1.45230
H	21.74450	-1.48120	-2.31920
C	8.60890	-5.46830	3.43360
H	9.12330	-5.06460	4.31180
H	7.53080	-5.30510	3.54610
C	8.06460	-7.74620	3.62260
H	7.04920	-7.48590	3.93290
C	11.12720	-5.83360	1.11560
H	10.45840	-6.36310	0.44420
C	20.64540	-5.16850	-3.90850
H	20.08510	-5.39800	-4.81870
C	24.42200	-6.71710	-1.27160
H	25.51200	-6.79900	-1.29630
C	21.33720	-8.84000	0.92620
H	21.65920	-9.47210	0.10640
C	8.32410	-9.17790	3.45920
C	8.72690	-11.81300	2.91730
H	8.93280	-12.84830	2.69760
C	24.49300	-6.78990	-3.71120
H	25.50040	-7.16590	-3.63180
C	23.77970	-6.44700	-2.55870
C	23.87850	-6.67670	-4.94650
H	24.39520	-6.95780	-5.85030
C	7.22240	-9.99790	3.19580
H	6.22760	-9.58210	3.19150
P	18.84660	-5.57620	7.35280
F	17.42710	-4.92040	7.76890
F	20.26300	-6.23610	6.93290
F	18.12350	-7.02110	7.25460
F	19.17270	-5.80940	8.91720
F	19.56380	-4.12740	7.43020
F	18.52500	-5.33420	5.78520
P	17.04800	-2.19640	5.51430
F	17.50370	-2.36540	7.05570
F	18.49560	-2.72510	5.02840
F	16.45820	-3.69980	5.48510
F	15.60640	-1.66230	6.01730
F	17.62030	-0.68350	5.53360
F	16.60590	-2.01820	3.97100
H	21.58460	4.97690	8.59250

References

[48] G. H. Penner, W. Li, *Inorg. Chem.* **2004**, *43*, 5588–5597.
[49] A. D. Becke, *J. Chem. Phys.***1993**, *98*, 5648–5652.
[50] J. P. Perdew, K. Burke, Y. Wang, *Phys. Rev.* **1996**, *54*, 16533–16539.
[51] P. J. Hay, W. R. Wadt, *J. Chem. Phys.* **1985**, *82*, 270–283.
[52] C. Bannwarth, S. Ehlert, S. Grimme, *J. Chem. Theory Comput.* **2019**, *15*, 1652–1671.
[53] S. Grimme, *J. Chem. Phys.* **2013**, *138*, 244104.
[54] B. Trzaskowski, J. P. Martínez, A. Sarwa, B. Szyszko, W. A. Goddard III, *J. Phys. Chem. A* **2024**, *128*, 3339–3350.
[55] Rigaku Oxford Diffraction, (2022), CrysAlisPro Software system, version 1.171.42.74a, Rigaku Corporation, Wroclaw, Poland.
[56] G. M. Sheldrick, Acta Crystallogr., Sect. C: Struct. Chem. **2015**, *71*, 3−8.
[57] G. M. Sheldrick, *Acta Crystallogr., Sect. A: Found. Adv.* **2015**, *71*, 3−8.
[58] O. V. Dolomanov, L. J. Bourhis, R. J. Gildea, J. A. K. Howard, J. Puschmann, *Appl. Crystallogr.* **2009**, *42*, 339−341.
[59] F. Hamon, E. Largy, A. Guédin-Beaurepaire, M. Rouchon-Dagois, A. Sidibe, D. Monchaud, J.-L. Mergny, J.-F. Riou, C.-H. Nguyen, M.-P. Teulade-Fichou, *Angew. Chem. Int. Ed.* **2011**, *50*, 8745–8749.
[60] J. Wang, Y. Zhang, Y. Li, E. Li, W. Ye, J. Pan, *Inorg. Chem.* **2022**, *61*, 8267–8282.
[61] M. Hirose, N. Tanaka, T. Usuki, *Bioorg. Med. Chem. Lett*. **2021**, *46*, 128165.
[62] S. A. Archer, T. Keane, M. Delor, E. Bevon, A. J. Auty, D. Chekulaev, I. V. Sazanovich, M. Towrie, A. J. H. M. Meijer, J. A. Weinstein, *Chem. Eur. J.* **2017**, *23*, 18239–18251.
